# Association of *Cutibacterium acnes* with human thyroid cancer

**DOI:** 10.3389/fendo.2023.1152514

**Published:** 2023-11-10

**Authors:** Vaishakhi Trivedi, Vanita Noronha, Peddagangannagari Sreekanthreddy, Sanket Desai, Disha Poojary, Linu Varghese, Pooja Gowda, Ashwin Butle, Rohit Mishra, Munita Bal, Neha Mittal, Swapnil Rane, Shubhada Kane, Sandip Basu, Vijay Patil, Nandini Menon, Ajay Kumar Singh, Pankaj Chaturvedi, Pratik Chandrani, Anuradha Choughule, Vidya Veldore, Kumar Prabhash, Amit Dutt

**Affiliations:** ^1^ Department of Medical Oncology, Tata Memorial Hospital, Mumbai, Maharashtra, India; ^2^ Homi Bhabha National Institute, Mumbai, Maharashtra, India; ^3^ 4baseCare Oncosolutions Pvt ltd, Institute of Bioinformatics and Applied Biotechnology, Bengaluru, Karnataka, India; ^4^ Integrated Cancer Genomics Laboratory, Advanced Centre for Treatment, Research, and Education in Cancer, Navi Mumbai, Maharashtra, India; ^5^ Department of Pathology, Tata Memorial Hospital, Mumbai, Maharashtra, India; ^6^ Consultant Onco-pathologist, Jaslok Hospital, Mumbai, Maharashtra, India; ^7^ Radiation Medicine Centre, Bhabha Atomic Research Centre, Tata Memorial Hospital, Mumbai, Maharashtra, India; ^8^ Department of Head and Neck Oncology, Tata Memorial Centre, Mumbai, Maharashtra, India; ^9^ Medical oncology molecular laboratory, Tata Memorial Hospital, Mumbai, Maharashtra, India; ^10^ Centre for Computational Biology, Bioinformatics and Crosstalk Lab, Advanced Centre for Treatment, Research, and Education in Cancer, Navi Mumbai, Maharashtra, India

**Keywords:** *Cutibacterium* acnes, immunosuppression, microbes, pathogen, RNAsequencing, thyroid cancer

## Abstract

**Introduction:**

The diverse subtypes of thyroid carcinoma have distinct clinical outcomes despite a comparable spectrum of underlying genetic alterations. Beyond genetic alterations, sparse efforts have been made to characterize the microbes associated with thyroid cancer. In this study, we examine the microbial profile of thyroid cancer.

**Methods:**

We sequenced the whole transcriptome of 70 thyroid cancers (40 papillary and 30 anaplastic). Using Infectious Pathogen Detector IPD 2.0, we analysed the relative abundance of 1060 microbes across 70 tumours from patients with thyroid cancer against 118 tumour samples from patients with breast, cervical, colorectal, and tongue cancer.

**Results:**

Our analysis reveals a significant prevalence of *Cutibacterium acnes* in 58.6% thyroid cancer samples compared to other cancer types (*p=0.00038*). Immune cell fraction analysis between thyroid cancer samples with high and low *Cutibacterium* loads identify enrichment of immunosuppressive cells, including Tregs (*p=0.015*), and other anti-inflammatory cytokines in the tumour microenvironment, suggesting an immune evasion/immunosuppression *milieu* is associated with the infection. A higher burden of *Cutibacterium acnes* was also found to be associated with poor survival defining a distinct sub-group of thyroid cancer.

**Conclusion:**

*Cutibacterium acnes* is associated with immune suppression and poor prognosis in a subpopulation of thyroid cancer. This study may help design novel therapeutic measures involving appropriate antibiotics to manage the disease better.

## Introduction

Thyroid cancer is the most frequent endocrine malignancy (1). The four main types are papillary thyroid cancer, follicular thyroid cancer, medullary thyroid cancer, and anaplastic thyroid cancer (2). Papillary thyroid cancer (PTC), a well-differentiated thyroid carcinoma with an incidence rate of 85%, tends to be relatively less aggressive and has a generally favourable prognosis (3). In contrast, 2% of thyroid cancer cases are comprised of anaplastic thyroid cancer (ATC) and undifferentiated thyroid carcinoma (2). Unlike PTC, ATC is highly aggressive and has a dismal prognosis (4). Despite of efforts to describe the mutation spectrum, fusion genes, copy-number modifications, and transcriptome alterations, the biology underlying thyroid cancer remains largely unknown.

Recently, microbes have been described as a characteristic hallmark of cancer (5). As commensal organisms, they are part of the human body’s normal flora. According to Globocan 2020, 19.7% of the countries in South Asia have malignancies infected with various bacteria (6). For instance, 60–90% of stomach cancer cases are linked to *Helicobacter pylori* (7); cervical cancer and head and neck cancers with *Human Papillomaviruses* (HPV) (8, 9); nasopharyngeal cancer with *Epstein-Barr virus* (EBV) (10); colorectal and head and neck cancer with *Fusobacterium nucleatum* (8, 11); *Cutibacterium acnes* with prostate cancer (12, 13). Pathogens, in general, play an immunomodulatory role in cancer, promoting tumour growth and generating chronic inflammation upon prolonged contact. However, the association between pathogenic microorganisms and thyroid cancer lacks systematic analysis, with sparse information about the functional association between microorganisms and their ability to control immunosuppressive behaviour.

Here, we investigate the microorganisms linked to thyroid cancer. We performed whole transcriptome sequencing on papillary thyroid cancer (n = 40) and anaplastic thyroid carcinoma (n = 30), in addition to reanalysing other malignancies with similar datasets, as reported earlier (14). Using Infectious Pathogen Detector (IPD 2.0) (14), we compared the microbial abundance of 70 thyroid cancer samples with five separate in-house cancer samples: breast cancer (n=49), tongue squamous cell carcinoma (TSCC) (n=15), cervical adenocarcinoma (n=21), and colorectal cancers (n=33). *Cutibacterium acnes* (or *propionibacterium*), a gram-positive facultative anaerobe, was significantly enriched in thyroid cancer tissue samples. Differential gene expression between thyroid cancer samples with high and low *Cutibacterium* loads suggests immune-modulatory pathways. In addition to analysing the immune cell fractions of both groups, we discovered a significant concentration of immunosuppressive cells in thyroid cancer samples with a higher *Cutibacterium* load. In the *C. acnes* high group, we observed an increase in immunosuppressive cytokines and a decrease in pro-inflammatory cytokines, validating our hypothesis. *Cutibacterium acnes* is related to late-stage illness, poor prognosis, and the formation of an immunosuppressive milieu in thyroid cancer, according to our findings.

## Methods

### Sample collection

From 2011 to 2020, we collected forty FFPE orphan tumour samples from patients with papillary thyroid cancer and thirty FFPE orphan tumour samples from patients with anaplastic thyroid cancer who were registered at the Tata Memorial Hospital in Mumbai, India. The FFPE samples were retrospectively acquired from the Department of Pathology at Tata Memorial Hospital in Mumbai, India. The Institutional Ethics Committee (IEC) of Tata Memorial Centre approved the study with wavier of consent (IEC number 900744). The research was conducted in accordance with institutional ethics committee as well as the declaration of Helsinki as revised in 2013 (15).

### Patient cohort

Forty patients with papillary thyroid cancer (PTC) who were treated with radioactive iodine (RAI) therapy following thyroidectomy were selected from a radiation medicine centre (RMC). The average age at diagnosis for the 40 patients is 39.2 years, with 37.5% male and 62.5% female patients (range 20 – 76 years). All patients have MACIS and AMES risk stratification scores determined using American Thyroid Association (ATAtechnique)’s based on AJCC 8th edition (https://www.thyroid.org/professionals/calculators/thyroid-cancer-staging-calculator/). The pathology department at Tata Memorial Hospital selected 30 patients diagnosed with anaplastic thyroid carcinoma. The average age of diagnosis for the 30 patients is 54 years, with 46.6% of the patients being male and 53.2% female (range 37 – 74 years). The complete clinical and histological characteristics of each patient are listed in **Table 1**.

**Table 1:** Clinicopathological details of thyroid cancer patients.

	Anaplastic thyroid cancer (n=30)	Papillary thyroid cancer (n=40)
Pathological subtypes		
Classical	NA	22
FVPTC	NA	17
others	NA	1
Age (years)		
>=45	25	14
<45	5	26
Gender		
Male	14	15
Female	16	25
Stage		
I	0	21
II	0	6
III	6	0
IV	21	0
Recurrence		
present	22	13
absent	8	27
Metastasis		
nodal metastasis	NA	16
distant metastasis	8	4
ETE		
present	23	17
absent	7	17
Thyroid abnormality		
present	4	11
absent	26	29
Performance status		
0	4	16
1	16	3
2	4	0
3	1	0
4	0	0

NA, Not Applicable.

### Preparation of RNA from FFPE samples

70 FFPE orphan tumour samples of papillary thyroid cancer and anaplastic thyroid carcinoma have been subjected to microtome sectioning, from which 5-6 10 um sections have been extracted for RNA extraction. The RNA was extracted from the FFPE sections using Qiagen’s FFPE RNA extraction kit according to the kit’s standard methodology (cat. No. 73504). A Nanodrop 2000c spectrophotometer was used to measure the RNA concentration (Thermo Fischer Scientific Inc).

### Whole transcriptome sequencing

Using the Illumina RNA Prep with enrichment Kit, formerly known as the TruSeq RNA Access Library Prep Kit, whole transcriptome sequencing was done on 40 FFPE papillary thyroid cancer samples and 30 FFPE samples of anaplastic thyroid cancer as detailed in **Supplementary Table S1**. Seventy samples’ whole transcriptome libraries were sequenced using Novaseq 6000 platform at 4basecare Pvt. Ltd., Bangalore, for 2 X 150 bp paired-end sequencing.

### Microbiome quantification from whole transcriptome sequencing data

IPD 2.0 was used to analyse and quantify pathogens using the full transcriptome data of 70 FFPE samples (14). IPD 2.0 quantifies pathogens from single- and paired-end reads data against a database of 1060 cancer-associated pathogens by removing low-quality, and ambiguous alignment reads and producing normalized read counts as FPM (fragments per million) and FPKM (fragments per kilobase per million). > 0 FPM was judged positive for the bacterial pathogen, and > 1 FPM was declared positive for the viral pathogen in thyroid carcinoma samples. As detailed by Desai et al.^11^, we have re-analysed pathogen quantification data from 118 entire transcriptomes of in-house samples representing four cancer types (breast cancer, cervical adenocarcinoma, colorectal cancer, and tongue squamous cell carcinoma). We applied stringent cut-off to reduce the noise in thyroid cancer (20 FPM) and similarly for other cancer types to resolve *Cutibacterium* abundance compared to earlier study (11). The Broad institute’s Morpheus toolkit (16) was used to build heatmap representations. We assess the overall microbial burden, which includes archaea, bacteria, plasmids, viruses, fungi, and protozoa, utilizing Kraken2 (17). The read counts from Kraken2 were normalized by translating them to reads per million for each sample and are referred to as the total microbiome burden (TMiB).

### Analysis of differential mRNA expression, immune cell fraction, and cytokine expression using whole-transcriptome sequencing data

Salmon (18) was utilized to do mRNA quantification or expression analysis and immune cell fraction on 70 FFPE samples’ whole transcriptome data. We evaluated raw data quality (fastq file) utilizing the FastQC (19) and MultiQC (20) tools. The low-quality reads with a Phred score of 20 were removed from the raw fastq files using trimmomatic v0.36 (21). In addition, Salmon was used to collecting high-quality readings for RNA quantitation. The DESeq2 package (22) was used for differential gene expression analysis. The false discovery rate of 0.05 was regarded as statistically significant, and log fold change >=1.5 and <1.5 are termed upregulated and downregulated genes, respectively. Using the t-test, the number of pathogens was compared between cancer types; factors with P-values of 0.05 were regarded as statistically different. CIBERSORTx (23) and CytoSig (24) were used to assess immune cell fraction and cytokine levels derived from the entire transcriptome.

### Analysis of qPCR validation of in-house cancer samples

We performed qPCR-based quantification of *Cutibacterium acnes* in thyroid cancer (n=44), tongue squamous cell carcinoma (n=8), and cervical adenocarcinoma (n=15) samples available in our laboratory. A total of 500 ng of RNA were taken for cDNA synthesis using high-capacity cDNA reverse transcription kit (Applied Biosystems, cat. no. 4368814). Further, quantitative real-time PCR was performed on Roche LC-480-II Real-Time PCR instrument using Powerup SYBR master mix (cat. No. A25742) on positive and negative samples as per the availability. A log-transformed delta Ct value was used to reflect the quantitation and relative change in expression of *Cutibacterium acnes* using *16s rRNA* gene primers (25); *beta-actin* was used as an internal control as detailed in **Supplementary Table S2**. We further accessed melt curve analysis to ensure amplification of desired products as shown in **Supplementary Figure S4**.

### Statistical analysis and correlation of clinical data

Using the chi-square test in Microsoft Excel, a correlation between samples with a high and low load of Cutibacterium and clinicopathological data was determined. A correlation with a p-value <= 0.05 was regarded as significant. Using the R tool survminer package, survival analysis was performed (26).

## Results

### Genomic landscape of microbes identifies *Cutibacterium acnes* abundance in thyroid cancer

**Figure 1:**
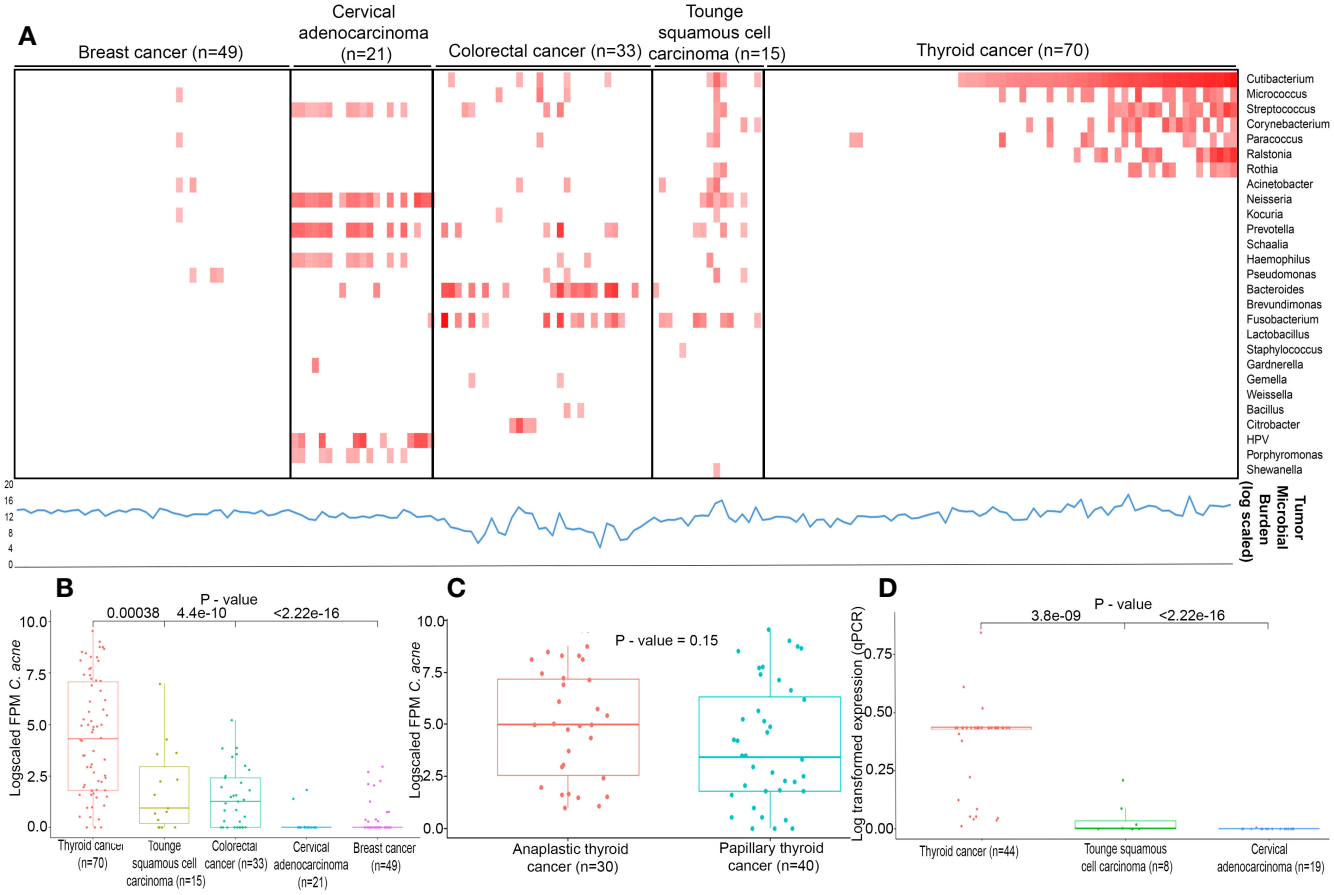
Landscape of infectious pathogens **(A)** Heatmap describes infectious pathogen burden in different cancer types identified from RNA-seq using IPD tool. Red colour square box indicates the log fold FPM in ascending order (0 to 12) as generated by IPD tool. Lower line (blue in colour) graph denotes Tumor Microbial burden (log scaled) quantified by Kraken2 **(B)** Expression levels (log fold FPM) of *Cutibacterium acnes* in different cancer types of Indian cohorts. **(C)** Expression levels (log fold FPM) of *Cutibacterium acnes* in Anaplastic and Papillary thyroid cancers in Indian cohort. **(D)** Expression levels (log transformed) of *Cutibacterium acnes* validated by qPCR in different cancer types of Indian cohorts.

We performed whole transcriptome sequencing of forty papillary and thirty anaplastic thyroid cancer to generate an average of 22.69 and 5.69 million paired end reads with an average alignment of 64% and 46% on human genome hg19, respectively (**Supplementary Table S3**). The raw reads with Phred score < 20 were trimmed to get high quality reads as described in methodology. Further, samples wherein transcripts show >10 counts were processed for downstream analysis. To identify microbial profile, normalized counts >= 1 FPM for bacteria and >1 FPM for viruses were considered to be present in a sample. Next, as a first step, we assessed the total microbial burden of 118 samples using kraken2 which indicates the proportion of captured pathogen reads among the reads sequenced per sample. We found the microbial load was consistent across all cancer types depicted as line graph in lower panel beneath the heatmap (**Figure 1A**). Next, using IPD 2.0 we analysed 70 whole transcriptome sequencing data of thyroid cancer samples along with 118 transcriptome data from in-house samples, including breast cancer (n=49), cervical adenocarcinoma (n=21), colorectal cancer (n=33), tongue squamous cell carcinoma (n=15) for 1060 microbes. With stringent thresholds applied in this study, as described in the materials and methods, we identified higher *HPV* expression in cervical cancer samples and *Fusobacterium* in colorectal and tongue cancer, as reported earlier^11^. In this study, we identified a greater prevalence of *Cutibacterium acnes* in 58.57% of thyroid cancer samples (range: 0 – 1527 FPM) (**Figure 1B**), followed by the presence of Bacteroides in 17.3% (range = 0 – 573 FPM), Micrococcus in 24.28% (range 0 to 275 FPM), Streptococcus in 22.8% (0 to 412 FPM), Corynebacterium in 21.4% of samples (0 – 195 FPM), Ralstonia in 18.57% (range 0 – 698 FPM), Acinetobacter in 11% (0 – 1107 FPM), and Neisseria in 10% (0 – 2992 FPM) in thyroid microflora. Within thyroid cancer subtypes, an enrichment of *Cutibacterium acnes* in anaplastic thyroid cancer relative to papillary thyroid cancer (**Figure 1C**) were observed. The expression of *Cutibacterium acnes* genes were validated in an in-house set of 44 thyroid cancer samples, 8 tongue squamous cell carcinoma, and 19 cervical carcinomas. Our real-time PCR validated higher levels of *Cutibacterium* in thyroid cancer compared to tongue squamous cell carcinoma (p=3.8e-09) and cervical adenocarcinoma (p=<2.22e-16) (**Figure 1D**).

### *Cutibacterium acnes* is associated with an immunosuppressive microenvironment in thyroid cancer

**Figure 2:**
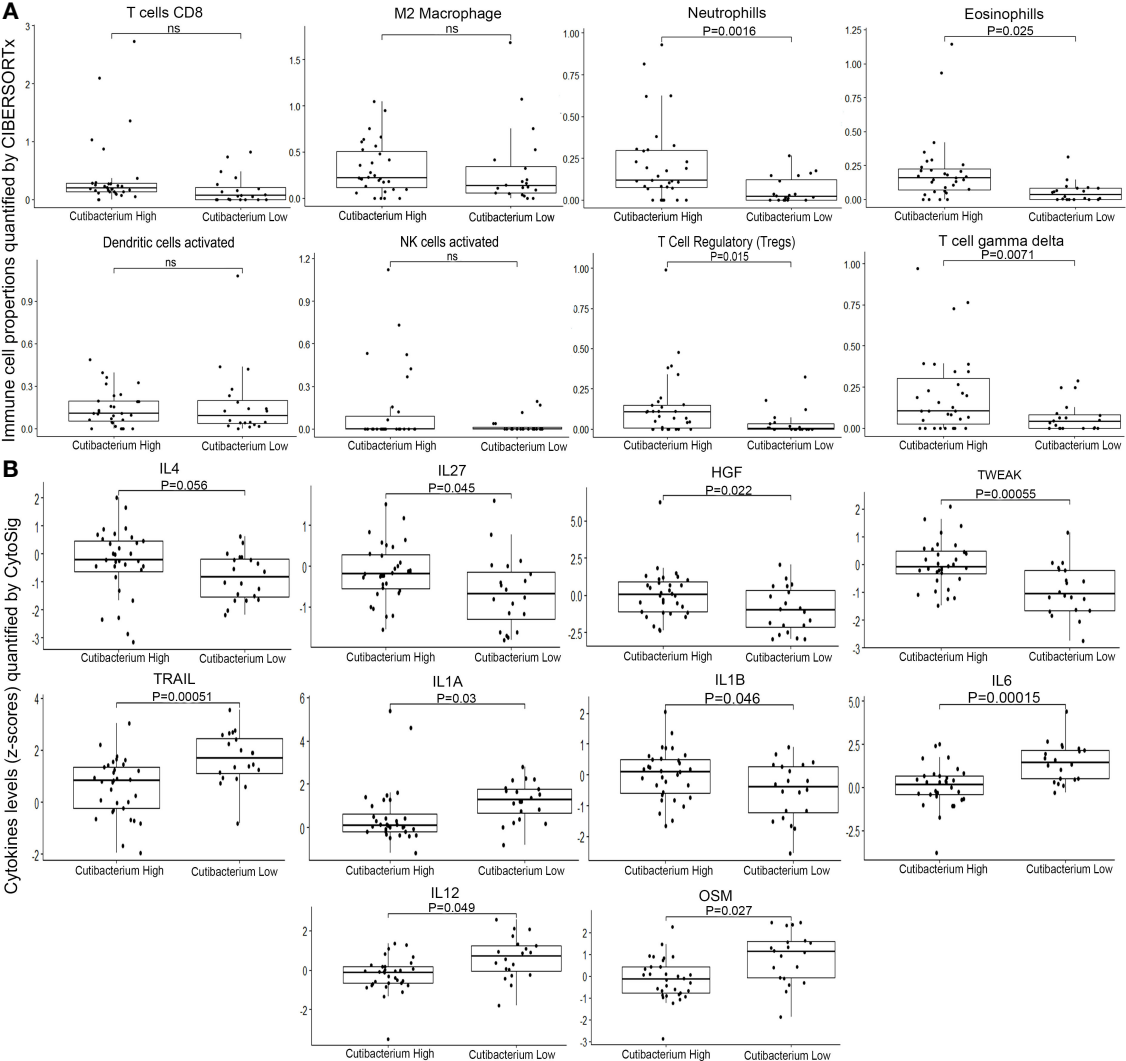
Composition of immune cells and cytokines in Tumor microenvironment of *Cutibacterium* high (n=31) and low (n=20) burden thyroid cancer **(A)** Immune cell fractions quantified by CIBERSHOTx from RNA-seq data **(B)** Cytokine levels (z-cores) quantified by CytoSig from RNA-seq data. ns, not significant.

To assess the role of *Cutibacterium acnes* in thyroid cancer, we divided the samples into two groups based on the *Cutibacterium* load gradient. The top 10% of quantile samples (n=32) were categorized as *Cutibacterium* high, while the remaining 90% (n=20) were *Cutibacterium* low. We performed differential gene expression analysis using DEseq2 between *Cutibacterium* high and low groups to examine differential pathway (**Supplementary Tables S4**–**S7**), differential immune cell fraction and differential cytokines. Further, using CIBERSORTx, we performed immune cell fraction analysis to compare the quantiles. We found high Neutrophils (p=0.0016) and Eosinophils (p=0.0025) in the *Cutibacterium* high-burden group, which is suggestive of pathogenic infection. Further, majority of immune response generating (adaptive or humoral) cells does not show significant difference between *Cutibacterium* high and low group, which may indicate insufficient immune response or immunological fatigue (**Figure 2A**; **Supplementary Figure S1**). In contrast, T regulatory cells (Tregs), characteristic immunosuppressive cells, were significantly higher in the *Cutibacterium* high group (p = 0.015). Other immunosuppressive cells, such as the T cell gamma delta (p=0.0017), were also considerably altered in the *Cutibacterium* high-burden group (**Figure 2A**). Also, M2 Macrophages (p = 0.8), act as anti-inflammatory, exhibits immune suppressive effect by lowering the antigen presenting capacity (27), tends to be higher in *Cutibacterium* high burden group is suggestive of reducing the immune response generation. Interestingly, the NK cells with anti-tumour activity had a lower load among the *Cutibacterium* high group (p=0.049), indicating a decline in anti-tumour efficacy. Additionally, we used CytoSig to analyse cytokine expression to comprehend this phenomenon. We observed a significantly higher level of anti-inflammatory cytokines, including IL4 (p=0.056), IL27 (p=0.045), HGF (p=0.022), and TWEAK (p=0.00055) in the *Cutibacterium* high group. In contrast, pro-inflammatory cytokines including IL1A (p=0.03), IL1B (0.046), IL6 (p=0.00015), IL12(p=0.049), and OSM (p=0.027) were downregulated or stably expressed (**Figure 2B**). We also observed that the apoptosis-inducing cytokine TRAIL (p=0.00051) was considerably downregulated in the *Cutibacterium* high group (**Figure 2B**). Higher levels of IL4 and lower levels of IL12 in *Cutibacterium* high burden group rationalize the mechanism of M2 macrophages exerting immune suppressive effect as reported (27). We also observed that IFN alpha beta and gamma signalling pathways were upregulated (p=0.05) in the *Cutibacterium* high group, whereas Interleukin-1 signalling, Interleukin-1 family signalling, RUNX1 and FOXP3 regulate the development of regulatory T lymphocytes (Tregs) – Treg inhibiting pathway was downregulated (p=0.05) in the *Cutibacterium* high group. Taken together, the findings suggest that the significant presence of *Cutibacterium acnes* in thyroid cancer samples contributes to the creation of an immunosuppressed environment around immune-responsive cells, thereby possibly promoting tumour growth and viability. However, the data indicates a correlation rather than a direct causal relationship between *Cutibacterium acnes* and the development of an immunosuppressive environment in thyroid cancer.

### *Cutibacterium acnes* infection is associated with poor survival in anaplastic and papillary thyroid cancer

**Figure 3:**
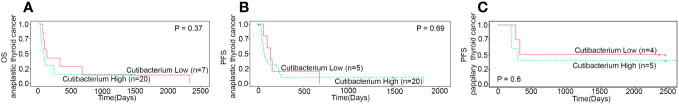
Survival analysis between *Cutibacterium* High and low burden subgroups **(A)** Overall survival (n=27) in anaplastic thyroid cancer **(B)** Progression free survival (n=25) in anaplastic thyroid cancer **(C)** Progression free survival (n=9) in papillary thyroid cancer.

Next, we analysed the relationship between *Cutibacterium acnes*, clinicopathological features, and patient survival in thyroid cancer. The R package survminor (Kaplan-Meier) was used to calculate overall survival and progression-free survival of *Cutibacterium* high and low burden groups. We noticed a downward trend in overall and progression-free survival in the *Cutibacterium*-rich group in both the subtypes where tumour stage is different (**Figure 3**; **Supplementary Figure S2**). We could not examine overall survival for papillary thyroid cancer due to insufficient data. Its association with the late stage of the disease (p=0.05) may correlate with the aggressive condition of the cells, such as distant metastasis and recurrence (**Table 2**). Overall, we observed that *Cutibacterium* infection is associated with poor patient outcomes.

**Table 2:** Correlation of *Cutibacterium* with clinicopathological characteristics of thyroid cancer.

Clinicopathological features	*Cutibacterium* high burden (n=32)	*Cutibacterium* low burden (n=20)
Age (years)		
<=45	11	11
>45	21	8
Gender		
Male	18	7
Female	14	13
Stage		
Early stage	8	8
Late stage	19	5
Distant metastasis		
Present	10	1
Absent	22	6
Recurrence/progression		
Present	21	9
Absent	11	10
Extra-thyroidal extension		
Present	17	10
Absent	8	7
Background papillary thyroid cancer		
Present	11	1
Absent	9	6

## Discussion

*Cutibacterium acnes* is the most prevalent commensal on healthy human skin, subserous glands, mucosal surfaces, and head and neck (28, 29). These microbes maintain balanced symbiotic/commensal relationships or a ‘eubiosis’ under normal conditions. A shift in the eubiotic balance or a ‘dysbiosis’ can lead to disease, as reported in multiple cancers (30–32). Under certain conditions, *Cutibacterium acnes* is known to cause deep tissue infections (33–36) and promote prostate cancer tumorigenesis through interferon-1 signalling and the cGAS-STING pathway (12, 28, 37, 38). While there are no reports of *Cutibacterium acnes* in the thyroid gland, a dysbiosis of the gut microbiome has been linked to thyroid cancer (39, 40). In this study, we systematically analysed whole transcriptome datasets of 70 thyroid cancers along with 118 tumour samples of breast, cervical, and other cancer types. Using Infectious Pathogen Detector, IPD 2.0, we standardized the quantification of distinct cancer-associated pathogens in several tumour types. In our analysis, we observed that *Cutibacterium acnes*-high tumours formed a distinct subgroup of thyroid cancer. We found a 5.35-log_2_ fold enrichment of the bacterium with 1.66891E-09 p value in the papillary and anaplastic thyroid tumour tissues as compared to other tumour types (**Figure 1B**; **Supplementary Figure S3**). A similar enrichment of *Cutibacterium acnes* was also reported earlier in prostate cancer (13). Significant enrichment of the bacterium in papillary and anaplastic thyroid carcinoma thus does underscore the association of the bacterium with the disease.

To elucidate the molecular and clinical characteristics associated with *Cutibacterium acnes* burden in thyroid cancer, we analysed the differential gene expression between *Cutibacterium*-high and -low tumours. The highly elevated genes varied dramatically between groups. Multiple inflammatory pathway genes were upregulated in *Cutibacterium*-rich tumours, indicating an inflamed tumour microenvironment. In addition, CytoSig analysis revealed that the *Cutibacterium*-high group had significantly higher levels of anti-inflammatory cytokines, including IL4, IL27, HGF, and TWEAK, while lower levels of pro-inflammatory cytokines, including IL1A, IL1B, IL6, IL12, and OSM. The apoptosis-inducing cytokine TRAIL was also discovered to be downregulated in the *Cutibacterium* high group. This shows that *Cutibacterium* acnes may play a role in thyroid cancers by inducing immunosuppression under an inflamed environment. The immunological profile of malignancies has been associated with microbial infection and bacterial dysbiosis. This suggests a possible role of *Cutibacterium acnes* in thyroid tumours by induction of chronic inflammation. We analysed the immune cell composition of *Cutibacterium*-high versus -low tumours using CIBERSORTx and detected a rise in T regulatory, or Tregs, cells. Immune cell enrichment study and gene expression analyses reveal that *Cutibacterium* load promotes an immunosuppressive environment, as reported in prostate cancer (41). However, Th17 and Tregs interplay and role in immunosuppression is emerging (42, 43). Th17 cells composition in combination with Tregs composition could give us more insight in immnosuppressive effect under *C. acnes* infection. However, CIBERSORTx analysis does not give output for Th17 cells and thus we could not determine the dual effect of Th17/Tregs in immunosuppressive environment. In addition, immunological suppression was shown by the downregulation of the Tregs inhibitory pathway and the pro-inflammatory signalling pathways, such as interleukin-1 signalling. In addition, we found that a larger load of the microorganism is associated with shorter overall survival in patients with thyroid cancer treated in-house.

There are several limitations of our study. Firstly, based on our findings, we are though unable to discern if this association (enrichment) is causal to the disease or a consequence. The establishment of causality requires additional co-culture experiments. Such experiments have the potential to provide a deeper understanding of the interactions between *Cutibacterium acnes* and thyroid cancer cells, shedding light on whether the bacterium actively contributes to the initiation and progression of cancer or whether its presence is a consequence of the cancerous environment. Such findings may have significant implications for thyroid cancer research and may lead to more precise diagnostic tools and individualized treatment strategies for affected individuals.

Secondly, we found that procedures like sample collection, DNA/RNA extraction, missing sampling blanks, samples/biopsies where the skin surface is traversed during biopsy collection in the FFPE production and sequencing could introduce microbial contamination, potentially affecting results. To ensure accuracy, we took strict precautions. The measures aimed to minimise contamination and enhance the reliability of our research, we used sterilised equipment, worked in a controlled environment, and followed rigorous lab protocols, including wearing sterile gloves and maintaining clean workspaces. Furthermore, in cases where tumours undergo biopsy procedures, particularly those involving percutaneous techniques, it is important the potential for microbial contamination originating from commensal bacteria that are frequently present on the skin or that predominantly inhabit sebaceous glands could not be thoroughly ruled out as described earlier (44, 45).

Thirdly, relating to computational microbial quantification protocol, a recent work by Gihavi et al. underscores the false positive detection of microbes from the metagenomic studies due to misclassification and batch effects (46). In our current study, we used two computational approaches (IPD 2.0 and Kraken2), working on distinct approaches, to quantify microbes from samples. IPD 2.0 uses stringent two step approach to negate human-microbe misalignment in the analysed samples. In the primary alignment step the sequence traces are first aligned to human genome and later un-aligned sequences are considered further. In the secondary alignment, IPD 2.0 further confirms the sequence uniqueness towards genera by ruling out any sequences which align significantly to multiple organisms (including human contigs) using a conventional alignment method Blast+ (47). We thus implement rigorous steps to address false positive detection of microbial traces in our dataset. Further, in the PCR validation step as described in methodology, we have QC’ed our primers and we ensure the identification of *C. acnes* based on melt curve analysis, negative samples and non-template controls where we did not see the amplification. Further, we classified thyroid tumours having the top 10% pathogen load as *Cutibacterium* -high and with no expression as *Cutibacterium* -negative.

Overall, the research identifies a correlation between the presence of *Cutibacterium acnes* and an immunosuppressive environment in cases of thyroid cancer, without establishing a causal relationship between these factors. Of note, the disparities in *Cutibacterium acnes* concentrations observed between thyroid cancer and other types of cancer may be attributed to inherent fluctuations in the microbiome, rather than a direct influence on the progression of thyroid cancer. In the same way, the increase in *Cutibacterium acnes* presence in thyroid cancer may be attributed to alterations in the tumour microenvironment. It is worth noting that the existence of microorganisms as well as the severity of cancer may as well be impacted by patient-related factors such as overall health and genetic predisposition. Additionally, it is crucial to emphasise that the study is based on retrospective data obtained from a singular region, which may introduce inherent biases and restrict the extent to which the findings may be generalised. Similarly, the potential association between *Cutibacterium acnes* and a more unfavourable outcome may be fortuitous, possibly attributable to unidentified confounding factors. Conversely, it is plausible that the microorganism could exert an impact on the response to treatment, rather than directly facilitating the progression of aggressive tumours. Taken together, these aforementioned limitations highlight the necessity for additional experiments in order to elucidate the intricate connections between *Cutibacterium acnes*, the tumour microenvironment, and the advancement of thyroid cancer.

## Conclusion

Our study presents a comprehensive landscape of microorganisms found from the transcriptome dataset of Indian origin across six distinct tumour types. We identified a subpopulation of thyroid cancers with a poor prognosis that may be driven predominantly by inflammation caused by *Cutibacterium* and other microorganisms. We further demonstrate that *Cutibacterium* is associated with advanced disease and poor overall and progression-free survival in thyroid cancer patients. This study could be a basis for designing more studies to understand the diversity in treatment response, disease recurrence and resistance, and adoption of additional measures such as using antibiotics for similar cancers of specific origin.

## Data availability statement

The datasets presented in this study can be found in online repositories. The names of the repository/repositories and accession number(s) can be found below: The genomic datasets can be accessed from the Array Express database under the accession numbers E-MTAB-12479, E-MTAB-12476, E-MTAB-9766, E-MTAB-6619, E-MTAB-4653, E-MTAB-8801, E-MTAB-11404, E-MTAB-9281, and E-MTAB-11407. The colorectal cancer data have been deposited at the European Genome-phenome Archive, under accession number EGAS00001005970.

## Ethics statement

The studies involving humans were approved by The Institutional Ethics Committee (IEC) of Tata Memorial Centre approved the study with wavier of consent (IEC number 900744). The studies were conducted in accordance with the local legislation and institutional requirements. The ethics committee/institutional review board waived the requirement of written informed consent for participation from the participants or the participants’ legal guardians/next of kin because The study was conducted on retrospective samples.

## Author contributions

VT, AD, and KP each participated in the conceptualization and design of the study. Additionally, AD and KP participated in Project administration, Supervision, Writing (review & editing), and fund acquisition. VT participated in Data curation, Formal analysis (performing experiments and data analysis), Investigation, Software (programming codes), Validation (qPCR-based validation), Visualization, and Writing-original draft. SD, DP, VN, RM, VP, AB, and AC participated in the formal analysis. PS, LV, PG, VV participated in methodology (transcriptome library preparation and data sharing), MB, NMi, SR, SK, SB, PaC, PrC, NMe, and AS provided the resources for the study. All authors contributed to manuscript revision, read, and approved the submitted version.

## Supplementary material

Supplementary Figure 1 Immune cell fractions quantified by CIBERSHOTx from RNA-seq data in *Cutibacterium* high (n=31) and low (n=20) burden thyroid cancer.

Supplementary Figure 2 Survival analysis between *Cutibacterium* High and low burden subgroups **(A)** Overall survival (n=51) **(B)** Progression free survival (n=34) of thyroid cancer patients.

Supplementary Figure 3 Average raw reads (FPM) across different tumour types.

Supplementary Figure 4 Melting curve analysis of *C. acnes* and β-actin. **(A)** Melt curve of *C. acnes* 16s rRNA. **(B)** Melt curve of β-actin used as internal control.
