# Supplementary material for: Association of Cutibacterium acnes with human thyroid cancer
Source: Front Endocrinol (Lausanne). 2023 Nov 10;14:1152514. doi: 10.3389/fendo.2023.1152514 (PMC10668118; doi:10.3389/fendo.2023.1152514)
Supplement: Supplementary Figure 1 — Immune cell fractions quantified by CIBERSHOTx from RNA-seq data in Cutibacterium high (n=31) and low (n=20) burden thyroid cancer. [file DataSheet_1.pdf]

Association of *Cutibacterium acne* with human thyroid cancer

Vaishakhi Trivedi[1,2], Vanita Noronha[1,2], Peddagangannagari Sreekanthreddy[3], Sanket Desai[2,4], Disha Poojary[1], Linu Varughese[3], Pooja Gowda[3,] Ashwin Butle[4], Rohit Mishra[4], Munita Bal[5], Neha Mittal[5], Swapnil Rane[5], Shubhada Kane[6], Sandip Basu[7], Vijay Patil[1], Nandini Menon[1], Ajay Kumar Singh[1], Pankaj Chaturvedi[2,8], Pratik Chandrani[2,9,10], Anuradha Choughule[1,2], Vidya Veldore[3], Kumar Prabhash[1,2#], Amit Dutt[2,4]*

1. Department of medical oncology, Tata Memorial Hospital, Ernest Borges Marg, Parel, Mumbai 400012, Maharashtra, India.
2. Homi Bhabha National Institute, Training School Complex, Anushakti Nagar, Mumbai 400094, Maharashtra, India
3. 4baseCare Oncosolutions Pvt ltd, Institute of Bioinformatics and Applied Biotechnology, Biotech Park Electronic City Phase I, Bengaluru 560100, Karnataka, India
4. Integrated Cancer Genomics Laboratory, Advanced Centre for Treatment, Research, and Education in Cancer, Kharghar, Navi Mumbai 410210, Maharashtra, India
5. Department of Pathology, Tata Memorial Hospital, Ernest Borges Marg, Parel, Mumbai 400012, Maharashtra, India.
6. Consultant onco-pathologist, Jaslok Hospital, Pedder Rd, IT Colony, Tardeo, Mumbai 400026, Maharashtra, India.
7. Radiation Medicine Centre, Bhabha Atomic Research Centre, Tata Memorial Hospital Ernest Borges Marg, Parel, Mumbai 400012, Maharashtra, India.
8. Department of Head and Neck Oncology, Tata Memorial Centre, Ernest Borges Marg, Parel, Mumbai 400012, Maharashtra, India
9. Medical oncology molecular laboratory, Tata Memorial Hospital, Ernest Borges Marg, Parel, Mumbai 400012, Maharashtra, India
10. Centre for computational biology, bioinformatics and crosstalk lab, Advanced Centre for Treatment, Research, and Education in Cancer, Kharghar, Navi Mumbai 410210, Maharashtra, India

# Corresponding author: Dr. Kumar Prabhash
Email address: kprabhash1@gmail.com
Correspondence address: Department of medical oncology, Tata Memorial Hospital, Ernest Borges Marg, Parel, Mumbai 400012, Maharashtra, India

* Co-Corresponding author: Dr. Amit Dutt
Email address: adutt@actrec.gov.in
Correspondence address: ACTREC-Tata Memorial Centre, Sector-22, Kharghar, Navi Mumbai 410210, Maharashtra, India

Supplementary Figure S1

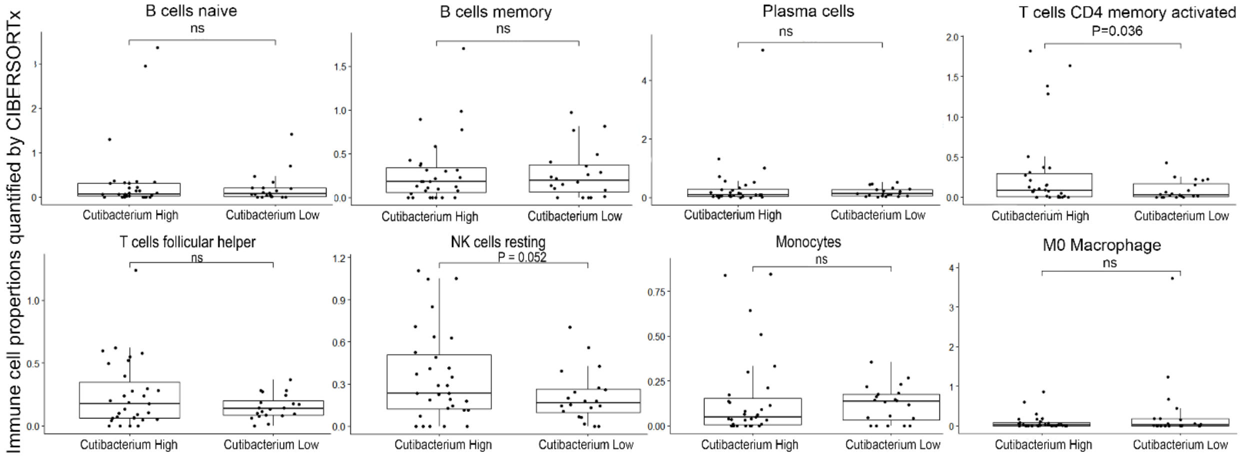

Supplementary Figure S2

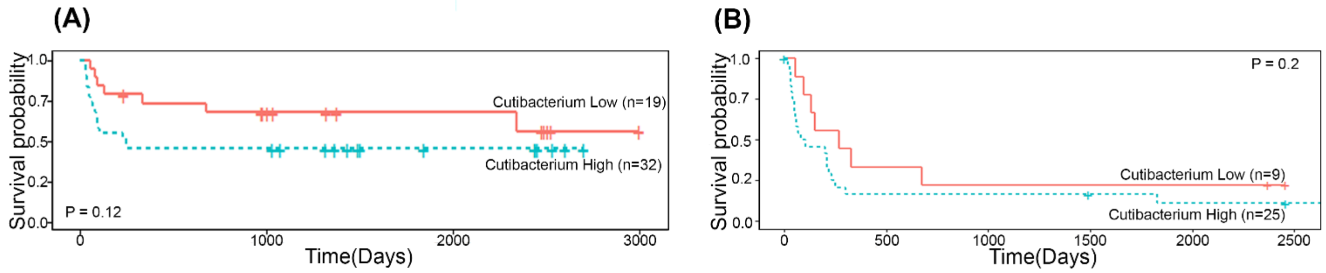

Supplementary Figure S3

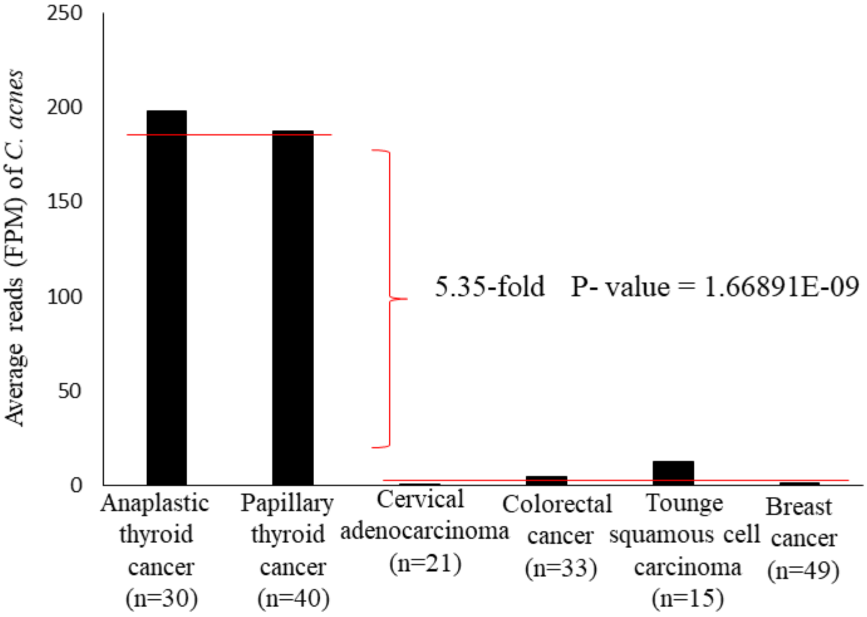

Supplementary Figure S4

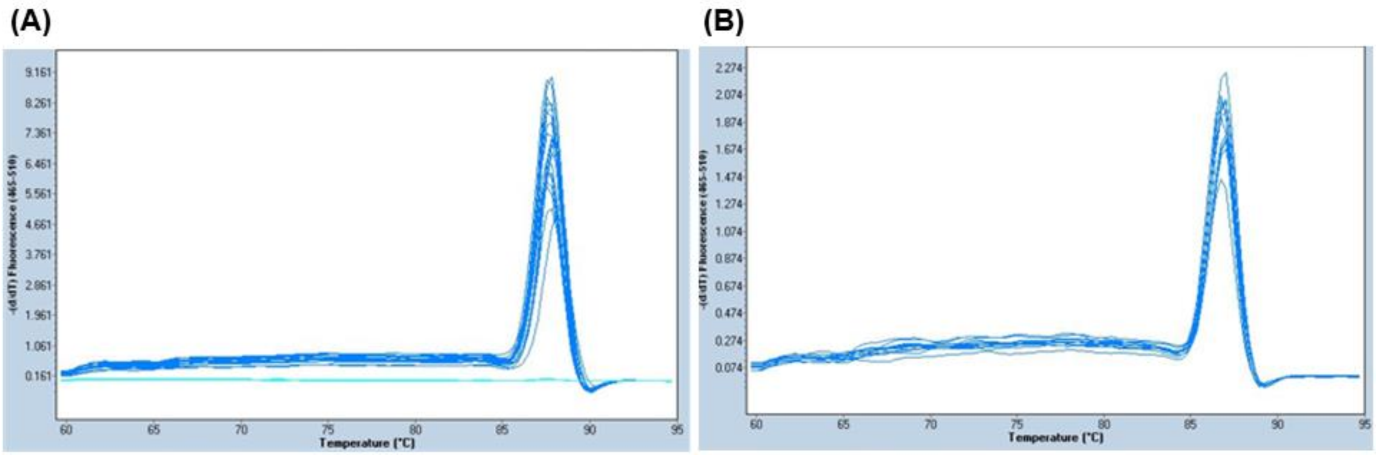

Supplementary Table S1: In-house whole transcriptome samples analysed using IPD.

Sr. No.	Tumor type	Data type	ADLABID	Tumor_Normal	Public ID
1	Anaplastic thyroid cancer	Transcriptome	AD3387	Tumor	E-MTAB-12479
2	Anaplastic thyroid cancer	Transcriptome	AD3388	Tumor	E-MTAB-12479
3	Anaplastic thyroid cancer	Transcriptome	AD3389	Tumor	E-MTAB-12479
4	Anaplastic thyroid cancer	Transcriptome	AD3390	Tumor	E-MTAB-12479
5	Anaplastic thyroid cancer	Transcriptome	AD3391	Tumor	E-MTAB-12479
6	Anaplastic thyroid cancer	Transcriptome	AD3392	Tumor	E-MTAB-12479
7	Anaplastic thyroid cancer	Transcriptome	AD3393	Tumor	E-MTAB-12479
8	Anaplastic thyroid cancer	Transcriptome	AD3394	Tumor	E-MTAB-12479
9	Anaplastic thyroid cancer	Transcriptome	AD3397	Tumor	E-MTAB-12479
10	Anaplastic thyroid cancer	Transcriptome	AD3399	Tumor	E-MTAB-12479
11	Anaplastic thyroid cancer	Transcriptome	AD3400	Tumor	E-MTAB-12479
12	Anaplastic thyroid cancer	Transcriptome	AD3403	Tumor	E-MTAB-12479
13	Anaplastic thyroid cancer	Transcriptome	AD3405	Tumor	E-MTAB-12479
14	Anaplastic thyroid cancer	Transcriptome	AD3406	Tumor	E-MTAB-12479
15	Anaplastic thyroid cancer	Transcriptome	AD3407	Tumor	E-MTAB-12479
16	Anaplastic thyroid cancer	Transcriptome	AD3408	Tumor	E-MTAB-12479
17	Anaplastic thyroid cancer	Transcriptome	AD3409	Tumor	E-MTAB-12479
18	Anaplastic thyroid cancer	Transcriptome	AD3410	Tumor	E-MTAB-12479
19	Anaplastic thyroid cancer	Transcriptome	AD3411	Tumor	E-MTAB-12479
20	Anaplastic thyroid cancer	Transcriptome	AD3412	Tumor	E-MTAB-12479
21	Anaplastic thyroid cancer	Transcriptome	AD3413	Tumor	E-MTAB-12479
22	Anaplastic thyroid cancer	Transcriptome	AD3414	Tumor	E-MTAB-12479
23	Anaplastic thyroid cancer	Transcriptome	AD3415	Tumor	E-MTAB-12479
24	Anaplastic thyroid cancer	Transcriptome	AD3416	Tumor	E-MTAB-12479
25	Anaplastic thyroid cancer	Transcriptome	AD3417	Tumor	E-MTAB-12479
26	Anaplastic thyroid cancer	Transcriptome	AD3418	Tumor	E-MTAB-12479
27	Anaplastic thyroid cancer	Transcriptome	AD3420	Tumor	E-MTAB-12479

28	Anaplastic thyroid cancer	Transcriptome	AD3421	Tumor	E-MTAB-12479
29	Anaplastic thyroid cancer	Transcriptome	AD3422	Tumor	E-MTAB-12479
30	Anaplastic thyroid cancer	Transcriptome	AD3424	Tumor	E-MTAB-12479
31	Papillary thyroid cancer	Transcriptome	AD3347	Tumor	E-MTAB-12476
32	Papillary thyroid cancer	Transcriptome	AD3348	Tumor	E-MTAB-12476
33	Papillary thyroid cancer	Transcriptome	AD3376	Tumor	E-MTAB-12476
34	Papillary thyroid cancer	Transcriptome	AD3349	Tumor	E-MTAB-12476
35	Papillary thyroid cancer	Transcriptome	AD3350	Tumor	E-MTAB-12476
36	Papillary thyroid cancer	Transcriptome	AD3377	Tumor	E-MTAB-12476
37	Papillary thyroid cancer	Transcriptome	AD3351	Tumor	E-MTAB-12476
38	Papillary thyroid cancer	Transcriptome	AD3352	Tumor	E-MTAB-12476
39	Papillary thyroid cancer	Transcriptome	AD3378	Tumor	E-MTAB-12476
40	Papillary thyroid cancer	Transcriptome	AD3353	Tumor	E-MTAB-12476
41	Papillary thyroid cancer	Transcriptome	AD3354	Tumor	E-MTAB-12476
42	Papillary thyroid cancer	Transcriptome	AD3355	Tumor	E-MTAB-12476
43	Papillary thyroid cancer	Transcriptome	AD3379	Tumor	E-MTAB-12476
44	Papillary thyroid cancer	Transcriptome	AD3380	Tumor	E-MTAB-12476
45	Papillary thyroid cancer	Transcriptome	AD3381	Tumor	E-MTAB-12476
46	Papillary thyroid cancer	Transcriptome	AD3356	Tumor	E-MTAB-12476
47	Papillary thyroid cancer	Transcriptome	AD3357	Tumor	E-MTAB-12476
48	Papillary thyroid cancer	Transcriptome	AD3358	Tumor	E-MTAB-12476
49	Papillary thyroid cancer	Transcriptome	AD3359	Tumor	E-MTAB-12476
50	Papillary thyroid cancer	Transcriptome	AD3360	Tumor	E-MTAB-12476
51	Papillary thyroid cancer	Transcriptome	AD3382	Tumor	E-MTAB-12476
52	Papillary thyroid cancer	Transcriptome	AD3361	Tumor	E-MTAB-12476
53	Papillary thyroid cancer	Transcriptome	AD3362	Tumor	E-MTAB-12476
54	Papillary thyroid cancer	Transcriptome	AD3363	Tumor	E-MTAB-12476
55	Papillary thyroid cancer	Transcriptome	AD3383	Tumor	E-MTAB-12476
56	Papillary thyroid cancer	Transcriptome	AD3384	Tumor	E-MTAB-12476
57	Papillary thyroid cancer	Transcriptome	AD3364	Tumor	E-MTAB-12476
58	Papillary thyroid cancer	Transcriptome	AD3365	Tumor	E-MTAB-12476
59	Papillary thyroid cancer	Transcriptome	AD3366	Tumor	E-MTAB-12476
60	Papillary thyroid cancer	Transcriptome	AD3385	Tumor	E-MTAB-12476
61	Papillary thyroid cancer	Transcriptome	AD3367	Tumor	E-MTAB-12476
62	Papillary thyroid cancer	Transcriptome	AD3368	Tumor	E-MTAB-12476
63	Papillary thyroid cancer	Transcriptome	AD3369	Tumor	E-MTAB-12476
64	Papillary thyroid cancer	Transcriptome	AD3386	Tumor	E-MTAB-12476
65	Papillary thyroid cancer	Transcriptome	AD3370	Tumor	E-MTAB-12476
66	Papillary thyroid cancer	Transcriptome	AD3371	Tumor	E-MTAB-12476
67	Papillary thyroid cancer	Transcriptome	AD3372	Tumor	E-MTAB-12476
68	Papillary thyroid cancer	Transcriptome	AD3373	Tumor	E-MTAB-12476
69	Papillary thyroid cancer	Transcriptome	AD3374	Tumor	E-MTAB-12476
70	Papillary thyroid cancer	Transcriptome	AD3375	Tumor	E-MTAB-12476

Supplementary Table S2: List of primers used for *Cutibacterium acne* validation by qPCR

Name	Primer	Sequence
Cutibacterium_16s	Forward	AGATGGGTGTGCCTCTTTTG
	Reverse	AAGGGGCATGATGACTTGAC
Beta-actin	Forward	CCATCTACGAGGGGTATGC
	Reverse	TGGTGGTGAAGCTGTAGCC

Supplementary Table S3: Transcriptome sequencing data statistics of papillary thyroid cancer (n=40) and anaplastic thyroid cancer (n=30)

LabID	Total reads (Millions)	Total Mapped reads on human genome (hg19) (Million)	% alignment on human genome hg19	Length (bp)	% GC
AD3347_R1	12.1	6.04	50.15%	151 bp	51%
AD3347_R2	12.1	6.04	50.15%	150 bp	51%
AD3348_R1	1	0.62	65.38%	151 bp	36%
AD3348_R2	1	0.62	65.38%	151 bp	37%
AD3376_R1	15.6	5.47	35.03%	151 bp	37%
AD3376_R2	15.6	5.47	35.03%	151 bp	36%
AD3349_R1	34.3	18.10	52.71%	151 bp	45%
AD3349_R2	34.3	18.10	52.71%	151 bp	45%
AD3350_R1	9.7	4.01	41.36%	150 bp	45%
AD3350_R2	9.7	4.01	41.36%	151 bp	45%
AD3377_R1	14	6.06	43.31%	151 bp	42%
AD3377_R2	14	6.06	43.31%	151 bp	42%
AD3351_R1	3.8	1.71	45.45%	151 bp	50%
AD3351_R2	3.8	1.71	45.45%	151 bp	48%
AD3352_R1	1.6	0.13	8.17%	151 bp	12%
AD3352_R2	1.6	0.13	8.17%	151 bp	13%
AD3378_R1	17.7	5.22	29.47%	151 bp	35%
AD3378_R2	17.7	5.22	29.47%	151 bp	35%
AD3353_R1	78.9	59.36	75.24%	150 bp	49%
AD3353_R2	78.9	59.36	75.24%	151 bp	49%
AD3354_R1	41.3	28.84	69.83%	150 bp	49%
AD3354_R2	41.3	28.84	69.83%	151 bp	50%
AD3355_R1	42.3	31.53	74.60%	150 bp	48%
AD3355_R2	42.3	31.53	74.60%	151 bp	48%
AD3379_R1	40.3	24.14	59.90%	151 bp	46%
AD3379_R2	40.3	24.14	59.90%	151 bp	46%
AD3380_R1	11.6	4.85	41.80%	151 bp	43%
AD3380_R2	11.6	4.85	41.80%	151 bp	42%
AD3381_R1	8.3	0.85	10.28%	151 bp	16%
AD3381_R2	8.3	0.85	10.28%	151 bp	16%
AD3356_R1	1.6	0.42	26.84%	151 bp	20%
AD3356_R2	1.6	0.42	26.84%	151 bp	21%

AD3357_R1	35.7	22.34	62.62%	150 bp	48%
AD3357_R2	35.7	22.34	62.62%	151 bp	48%
AD3358_R1	30.7	14.15	46.09%	151 bp	43%
AD3358_R2	30.7	14.15	46.09%	151 bp	43%
AD3359_R1	45.3	27.24	60.17%	150 bp	50%
AD3359_R2	45.3	27.24	60.17%	151 bp	51%
AD3360_R1	39.1	26.90	68.84%	151 bp	47%
AD3360_R2	39.1	26.90	68.84%	151 bp	47%
AD3382_R1	40.8	25.11	61.57%	151 bp	46%
AD3382_R2	40.8	25.11	61.57%	151 bp	47%
AD3361_R1	1.9	0.07	3.33%	151 bp	5%
AD3361_R2	1.9	0.07	3.33%	151 bp	5%
AD3362_R1	29.1	16.33	56.17%	150 bp	48%
AD3362_R2	29.1	16.33	56.17%	151 bp	48%
AD3363_R1	2.3	0.72	30.91%	151 bp	41%
AD3363_R2	2.3	0.72	30.91%	151 bp	41%
AD3383_R1	44.4	26.53	59.70%	150 bp	45%
AD3383_R2	44.4	26.53	59.70%	151 bp	46%
AD3384_R1	1.2	0.38	30.27%	151 bp	24%
AD3384_R2	1.2	0.38	30.27%	151 bp	24%
AD3364_R1	1.5	0.28	18.17%	151 bp	22%
AD3364_R2	1.5	0.28	18.17%	151 bp	23%
AD3365_R1	44.8	24.96	55.66%	150 bp	45%
AD3365_R2	44.8	24.96	55.66%	151 bp	44%
AD3366_R1	7.9	3.17	39.87%	151 bp	47%
AD3366_R2	7.9	3.17	39.87%	151 bp	47%
AD3385_R1	37.8	25.45	67.29%	151 bp	44%
AD3385_R2	37.8	25.45	67.29%	151 bp	44%
AD3367_R1	9.1	0.33	3.69%	151 bp	6%
AD3367_R2	9.1	0.33	3.69%	151 bp	6%
AD3368_R1	1.8	0.07	3.70%	151 bp	5%
AD3368_R2	1.8	0.07	3.70%	151 bp	5%
AD3369_R1	5.4	0.15	2.73%	151 bp	5%
AD3369_R2	5.4	0.15	2.73%	151 bp	5%
AD3386_R1	10.9	1.17	10.71%	151 bp	13%
AD3386_R2	10.9	1.17	10.71%	151 bp	13%
AD3370_R1	45.6	31.32	68.62%	150 bp	50%
AD3370_R2	45.6	31.32	68.62%	151 bp	51%
AD3371_R1	46.4	24.24	52.26%	150 bp	50%
AD3371_R2	46.4	24.24	52.26%	150 bp	49%
AD3372_R1	9.6	5.65	58.65%	151 bp	47%
AD3372_R2	9.6	5.65	58.65%	151 bp	47%
AD3373_R1	37.7	27.44	72.83%	150 bp	49%
AD3373_R2	37.7	27.44	72.83%	151 bp	49%

AD3374_R1	1.6	0.06	3.95%	151 bp	6%
AD3374_R2	1.6	0.06	3.95%	151 bp	6%
AD3375_R1	42.9	24.24	56.49%	150 bp	49%
AD3375_R2	42.9	24.24	56.49%	151 bp	48%
AD3400_R1	10.8	1.37	95.84%	151 bp	56%
AD3400_R2	10.8	1.37	95.84%	151 bp	55%
AD3387_R1	0.6	0.33	83.87%	101 bp	39%
AD3387_R2	0.6	0.33	83.87%	107 bp	38%
AD3388_R1	1.7	1.12	91.35%	101 bp	46%
AD3388_R2	1.7	1.12	91.35%	107 bp	42%
AD3389_R1	0.2	0.07	56.37%	142 bp	30%
AD3389_R2	0.2	0.07	56.37%	144 bp	31%
AD3390_R1	10.1	7.36	93.45%	103 bp	48%
AD3390_R2	10.1	7.36	93.45%	109 bp	45%
AD3391_R1	1.4	0.60	53.65%	140 bp	13%
AD3391_R2	1.4	0.60	53.65%	142 bp	17%
AD3392_R1	5.3	0.93	21.36%	146 bp	8%
AD3392_R2	5.3	0.93	21.36%	147 bp	14%
AD3393_R1	1.1	0.39	50.98%	134 bp	15%
AD3393_R2	1.1	0.39	50.98%	135 bp	20%
AD3394_R1	1.9	1.17	76.66%	127 bp	29%
AD3394_R2	1.9	1.17	76.66%	130 bp	31%
AD3397_R1	1	0.76	95.23%	101 bp	48%
AD3397_R2	1	0.76	95.23%	105 bp	46%
AD3399_R1	10.7	8.14	92.83%	106 bp	50%
AD3399_R2	10.7	8.14	92.83%	111 bp	48%
AD3403_R1	0.2	0.13	70.04%	138 bp	30%
AD3403_R2	0.2	0.13	70.04%	139 bp	32%
AD3405_R1	2.2	1.22	72.13%	103 bp	51%
AD3405_R2	2.2	1.22	72.13%	107 bp	49%
AD3406_R1	23.8	19.24	94.47%	112 bp	50%
AD3406_R2	23.8	19.24	94.47%	115 bp	48%
AD3407_R1	10.8	7.54	92.12%	105 bp	51%
AD3407_R2	10.8	7.54	92.12%	112 bp	49%
AD3408_R1	0.3	0.14	70.73%	133 bp	34%
AD3408_R2	0.3	0.14	70.73%	137 bp	36%
AD3409_R1	1	0.18	20.88%	127 bp	43%
AD3409_R2	1	0.18	20.88%	129 bp	42%
AD3410_R1	5.2	3.45	92.22%	98 bp	45%
AD3410_R2	5.2	3.45	92.22%	104 bp	45%
AD3411_R1	22.3	17.10	93.97%	105 bp	48%
AD3411_R2	22.3	17.10	93.97%	110 bp	46%
AD3412_R1	2	1.36	91.49%	100 bp	43%
AD3412_R2	2	1.36	91.49%	106 bp	41%

AD3413_R1	1.8	0.62	44.35%	148 bp	7%
AD3413_R2	1.8	0.62	44.35%	149 bp	14%
AD3414_R1	8.3	6.10	91.96%	105 bp	42%
AD3414_R2	8.3	6.10	91.96%	105 bp	42%
AD3415_R1	1.7	0.74	53.35%	142 bp	17%
AD3415_R2	1.7	0.74	53.35%	143 bp	22%
AD3416_R1	1.4	0.55	46.59%	122 bp	49%
AD3416_R2	1.4	0.55	46.59%	125 bp	48%
AD3417_R1	0.5	0.23	68.66%	104 bp	44%
AD3417_R2	0.5	0.23	68.66%	110 bp	42%
AD3418_R1	0.3	0.12	54.48%	146 bp	13%
AD3418_R2	0.3	0.12	54.48%	147 bp	19%
AD3420_R1	4.8	3.66	95.16%	99 bp	50%
AD3420_R2	4.8	3.66	95.16%	105 bp	47%
AD3421_R1	3.6	2.78	86.18%	130 bp	43%
AD3421_R2	3.6	2.78	86.18%	131 bp	42%
AD3422_R1	22.7	17.15	88.97%	109 bp	52%
AD3422_R2	22.7	17.15	88.97%	112 bp	50%
AD3424_R1	18.4	14.80	96.58%	104 bp	49%
AD3424_R2	18.4	14.80	96.58%	107 bp	47%

Supplementary Table-S4: Differentially expressed genes in *Cutibacterium acne* high versus low sub-group of papillary thyroid cancer samples (n=40). Padj is p-values, adjusted for multiple testing with the Benjamini-Hochberg procedure.

Gene name	log2FoldChange	pvalue	padj
REXO1L6P	27.47924344	9.95E-21	1.85E-18
EFHD2	26.9683621	5.84E-31	7.07E-28
HSPA1A	26.4241844	2.81E-19	3.86E-17
GOLGA8T	25.86987939	1.55E-18	2.06E-16
ENSG00000254701	25.61870751	3.34E-18	4.30E-16
MSH5	25.42672298	5.96E-18	7.52E-16
DYRK1A	25.06620054	1.69E-17	2.07E-15
NAIPP4	24.818869	3.67E-17	4.35E-15
ABCF1	24.81586989	3.70E-17	4.35E-15
POMP	24.66486458	5.79E-17	6.68E-15
ENSG00000255292	24.36997461	1.37E-16	1.43E-14
SNRPCP6	24.35957267	1.42E-16	1.46E-14
SLC35A1	24.09572515	3.08E-16	2.72E-14
ENSG00000259665	23.36779515	2.54E-15	2.01E-13
PRAMEF31P	23.13686985	4.90E-15	3.70E-13
ENSG00000185641	22.50003188	2.90E-14	2.12E-12
BCLAF1P2	22.35291423	4.38E-14	3.12E-12
IL5RA	22.0578668	8.83E-14	5.97E-12

ACOXL	21.78287539	1.82E-13	1.16E-11
PDXP	20.47932803	4.62E-12	2.22E-10
SH3D21	12.47837555	2.27E-05	0.000337783
USP17L13	10.73079739	0.000267414	0.002257166
GRIK3	9.979736414	0.000684268	0.004614137
FAT3	9.294784691	0.001607744	0.008862329
EFCAB5	9.037334287	0.001861726	0.009940153
ENSG00000238015	8.491292417	0.003996379	0.017989865
ACO2	8.260610762	0.005183753	0.022147597
IGBP1	8.225187046	0.005322532	0.022589037
CD302	7.996648075	0.00647356	0.026141569
SLC2A10	7.839562687	0.008141394	0.031204378
SPATA21	7.76931594	0.008550452	0.032362313
CLEC18A	7.743362168	0.007853607	0.030360926
H2BC19P	7.45451145	0.010522953	0.037958708
ENSG00000278931	7.331779327	0.009597865	0.035440074
TUBBP1	7.265563319	0.006603274	0.026567238
ENSG00000235018	7.122554823	0.0160641	0.052360437
ENSG00000226945	6.964342354	0.018628695	0.05856699
HLA-F	6.937131977	0.019100134	0.059661426
RPS27P23	6.937131977	0.019100134	0.059661426
SSC5D	6.692972442	0.022634828	0.06787948
CCNJ	6.602829123	0.015785975	0.05178142
NRIP2	6.516867042	0.007596027	0.029583055
TRIM53BP	6.513863021	0.027959567	NA
EIF4A1P10	6.496599574	0.028392053	NA
ENSG00000235371	6.471440077	0.029014435	0.08201558
ABCB11	6.43029106	0.030071581	NA
SLC14A1	6.402465334	0.030700139	0.085522321
RPL9P31	6.355558174	0.032076299	NA
FAM189A2	6.337008958	0.002410696	0.012132666
ACTR2	6.319949094	0.030566519	0.085307158
MAVS	6.248618401	0.000532601	0.003787665
CDS1	6.244346695	0.035274181	NA
LRPPRC	6.229097288	0.035562263	NA
TATDN2P3	6.190569112	0.036915194	NA
ERVW-1	6.149666063	0.000845898	0.0054116
DUX4L26	6.061456374	0.001438813	0.008183927
HLA-DRA	6.028465581	0.03806212	0.099741048
PCDHA7	5.994089737	0.009842346	0.036172153
TM2D2	5.988727727	0.01613467	0.052512516
ENSG00000270870	5.931393887	0.045787104	NA
ZC3H7B	5.917458363	0.000159222	0.001517501
OR4A9P	5.868345642	0.048193196	NA

PLCB4	5.849259172	0.012831853	0.044275016
RPL29P14	5.838799007	0.03590953	0.09563266
MGAT3	5.816434552	0.050256307	NA
SLC22A18	5.801773383	0.048536897	0.119651853
CDH12	5.7967074	0.005245679	0.022349067
LIN7C	5.627302739	0.043060913	NA
USP12P2	5.518246614	0.012644201	NA
RAB1A	5.303685298	0.012028251	0.042102356
CMBL	5.300439077	0.031062281	0.086202157
SKINT1L	5.283529575	0.000447767	0.003302725
KMO	5.193512995	0.030944297	0.085984795
CEP162	5.118235087	0.002524774	0.012576075
ZFP30	4.806963691	0.019865276	0.061495983
PGM5	4.699858101	0.027100368	0.077989265
ZNF169	4.627437637	0.034201436	0.092300603
CYSLTR2	4.602749651	0.011406282	0.040403907
ENSG00000225813	4.563984966	0.048725266	NA
MUC4	4.555215441	0.044172018	0.111363904
TAC4	4.510753788	0.042953021	0.109136198
SDF4	4.467182006	0.001906845	0.010138374
C1orf216	4.3412239	0.043016876	NA
COL1A2	4.29080216	0.000981662	0.006084019
ARHGEF5	4.276158529	0.046360621	0.115629292
RPS18	4.24837748	5.54E-05	0.000682265
S100A1	4.22672452	0.043503011	0.110182139
RIPPLY3	4.14945536	0.049623407	NA
PHIP	4.113907809	0.024939291	0.073037789
VGLL4	4.113030042	0.003877014	0.017556073
PSMA5	4.099835581	0.028707774	0.081434005
TESC	4.06060311	0.041188248	0.105668198
POLR3H	3.987727928	0.008282647	0.031643076
HEATR5A	3.937274191	0.001129407	0.006778782
PDE4DIPP6	3.92117272	0.022436113	NA
PNN	3.908956384	0.023191001	0.069138884
ZNF236	3.89847878	0.039931829	0.103365024
BAG6	3.892900318	0.049219891	0.120882398
GGT1	3.889344271	0.012127283	0.042326597
UBAC2	3.846096974	0.006980463	0.027747628
SPP2	3.8376444	0.032005898	NA
CALM2	3.834135891	0.009919457	0.036359931
MUC6	3.823357152	0.017732725	0.056430939
NFAT5	3.792031922	0.030431085	0.085066479
POLR2A	3.778165268	0.032756627	0.089520481
SLC2A4RG	3.77585411	0.008403565	0.031956252

ABHD17A	3.769504434	0.038887475	0.101333477
SUPT16H	3.689532309	0.016378745	0.053127941
PDAP1	3.674477248	0.033590541	0.091120552
GIGYF2	3.661806479	0.002254108	0.011508055
ZNF580	3.570886466	0.039764562	0.103127378
STAM2	3.509914886	0.016434039	0.05327199
AQP6	3.506137416	0.037902524	NA
CROT	3.484460308	0.021432373	0.065131169
INGX	3.454390293	0.021259201	NA
FARSA	3.445674554	0.022065679	0.066537863
RPL5	3.437019635	0.026264839	0.076054932
ENSG00000254503	3.43286737	0.043550823	NA
NONO	3.42922947	0.006120034	0.025009078
AP3D1	3.423492269	0.007400814	0.02898722
MED16	3.412283488	0.018119972	0.057459823
HLA-V	3.385775048	0.000221835	0.001955639
PKD1P1	3.380942299	0.043589504	0.110331944
RIN1	3.380032375	0.048853883	0.120188496
RPL26	3.330984128	0.023461196	0.069714462
COL16A1	3.313864233	0.006866736	0.02738247
IL17RA	3.304823771	0.048602095	0.119763795
ARHGAP44	3.298852622	0.022492477	0.067589112
EPS8L1	3.293746919	0.012143822	0.042359892
RDH13	3.291026852	0.016447257	0.053291115
RPS16	3.284264908	0.016919025	0.054435908
TP53BP1	3.282337124	0.003636314	0.016703585
ATXN7L3	3.224861993	0.028980368	0.081976717
TENM4	3.209962467	0.038335048	0.100304241
SBNO2	3.205213783	0.005826545	0.024102701
HERC1	3.196980867	0.002517475	0.012560393
TSC2	3.191456609	0.021707324	0.065765563
TLL2	3.190262015	0.040574267	NA
LINC00672	3.180861844	0.025932151	NA
MAGI1	3.17016109	0.005664361	0.023650027
UQCRC1	3.142729675	0.014273942	0.047872485
QTRT1	3.126729319	0.04200491	0.107349304
P2RX5-TAX1BP3	3.111728063	0.010970791	0.039182784
SNF8	3.09338707	0.020735147	0.063570978
PIGQ	3.078041015	0.013365794	0.045635987
GABBR1	3.069991899	0.021892219	0.06616315
GTDC1	3.069698577	0.028535096	0.081058155
FRMPD1	3.06015746	0.000536837	0.00380884
TCIRG1	3.054525462	0.01525178	0.050453006
SPECC1	3.032013626	0.010094322	0.036812797

RNF227	3.031227658	0.003026611	0.014508553
PLXNB2	3.028342966	0.001681513	0.009162369
MYCBP2	3.027660375	0.033244406	0.090445108
NSFL1C	3.016652675	0.046988308	0.11685771
NEPRO	3.007721223	0.049298363	0.121012489
HMG20B	2.966969185	0.017770006	0.056512914
POLR3E	2.956838177	0.046381661	0.115657936
IRS3P	2.954259971	0.007085548	0.028045608
LTBP3	2.911804964	0.00763008	0.02966816
DGLUCY	2.904487472	0.032274921	0.088584047
SNX25	2.893537129	0.027638981	0.079256627
ATP2B4	2.892619246	0.010741722	0.038489582
CD63	2.887579849	0.003562544	0.01644978
MYO1C	2.85257506	0.030878885	0.085881898
CEP170	2.851755184	0.015536391	0.051087334
WASH6P	2.851337611	0.024616803	0.072426296
ADCY6	2.845763403	0.042687431	0.108589503
SRRT	2.834959012	0.023182114	0.069129418
SRRM2	2.822566185	3.18E-05	0.000438339
KIAA0232	2.81831806	0.0324567	0.088872185
PAXBP1	2.81724523	0.045869894	0.114783792
SCRN2	2.781961893	0.025307875	0.073820468
RHOC	2.779517604	0.013763863	0.046574727
DMXL1	2.772328002	0.004224497	0.018840572
GON4L	2.765738623	0.034558812	0.092996855
RSRP1	2.714517266	0.008775927	0.032999013
PGGHG	2.705466	0.033542067	0.091050275
DACT3	2.699303655	0.007571014	0.029513543
SHFL	2.695062582	0.040354267	0.104137771
RPL6	2.644074365	0.01859499	0.058521518
ATRN	2.629358873	0.041980832	0.107314887
EPOR	2.627216137	0.027208125	0.078224976
SDHAP1	2.595213041	0.029256436	0.082487747
CHFR	2.590920725	0.03726897	0.098140998
SLC4A8	2.587538986	0.00091319	0.005750908
TMTC2	2.559210415	0.036444161	0.096546316
LDHB	2.538840051	0.032588675	0.089121854
GNAS	2.538128088	0.003744329	0.017070189
GRINA	2.530024593	0.042694766	0.108589503
HDLBP	2.529214575	1.96E-06	4.11E-05
ITGA3	2.509310068	0.013878918	0.046833125
LMO7	2.494455091	0.007885809	0.030456233
C12orf43	2.493610898	0.047298223	0.117459518
BCAM	2.463631273	0.049298793	0.121012489

TNKS1BP1	2.425360687	0.024807666	0.072687484
SEMA6B	2.417227031	0.021076608	0.064296544
ATP11A	2.407359847	0.012718252	0.043970785
EEF1A1P5	2.405638245	0.01092843	0.039089158
RACK1	2.402897675	0.00189543	0.010088958
USP34	2.400551222	0.040072477	0.103551935
PLD3	2.398792269	0.023594071	0.069984025
LRP1	2.370839623	0.047236255	0.117391932
PML	2.357156472	0.048312918	0.119237273
MDN1	2.331514233	0.010680383	0.038326521
SPG11	2.331451026	0.00997216	0.036477192
SYNE2	2.307786759	0.001580864	0.008769375
ZMYM2	2.278378422	0.017932969	0.056926476
ENSG00000271519	2.251291516	0.00934827	0.034698395
BRWD1	2.234596375	0.044159022	0.111359162
MAPK12	2.220345907	0.019231467	0.060025187
DNM1P47	2.219575046	0.049808332	0.121966427
ATXN2L	2.202903509	0.014483439	0.048387397
INTS10	2.199614474	0.035177524	0.094368073
TMEM138	2.182298867	0.01006114	0.036766669
ZNF556	2.173517047	0.025546239	0.074365481
LRIG3	2.154297948	0.036393569	0.096451928
NFE2L1	2.15146912	0.042821059	0.108842104
FN1	2.127964561	0.039799314	0.103127378
DDX39B	2.087672009	0.015728129	0.051633652
RASEF	2.06556088	0.000202948	0.001822316
LAIR1	2.054219328	0.021859386	0.06608042
CABIN1	2.050431627	0.014196686	0.04770591
HNRNPH1	2.042470567	0.038705046	0.1010539
ARHGAP5	1.998790445	0.0038752	0.017554424
ATP2A2	1.97432198	0.050531382	0.123412398
ALDOA	1.968292173	0.042073668	0.107461423
KBTBD12	1.962371529	0.006664065	0.026752753
MYO15B	1.935881243	0.000818773	0.005274307
RGL3	1.9214744	0.015442234	0.050834182
NPIPB5	1.908013202	0.020598425	0.063199833
PCDH11Y	1.897765016	0.012120212	0.042314118
LTBP4	1.885702431	0.040602802	0.104520695
ENSG00000283809	1.880300892	0.004519652	0.019856939
SPINK9	1.847193145	0.039165591	0.101882724
GAPDH	1.795861437	0.042274924	0.10783892
MLLT6	1.738391026	0.048845613	0.120188496
ACSL6	1.712934983	0.024493954	0.072134992
RPL13A	1.674511167	0.029029996	0.082040409

EXD1	1.614707665	0.033996585	0.091897726
RPL10	1.536863376	0.030624681	0.085371058
TMEM212	1.522315789	0.045388243	0.113813816
SLC25A29	-1.377893363	0.017205028	0.05507793
NPIPA1	-1.464495679	0.050517314	0.123402939
ANKRD12	-1.820896423	0.023481639	0.069714462
MORC4	-1.822545196	0.01109092	0.039553476
MAP4	-1.846480576	0.04266902	0.108569648
SUCO	-1.892122437	0.041769537	0.10691023
DCBLD2	-1.913788869	0.032608263	0.089155277
ACLY	-1.936342679	0.043573854	0.110331944
KDM3B	-1.947358986	0.047852874	0.118448095
ACAA1	-1.972453702	0.045669446	0.114353119
SLC38A9	-2.040482262	0.045234285	0.113498298
GGA2	-2.063640518	0.040591693	0.104514327
MIPOL1	-2.125022708	0.028469589	0.080929052
HPCAL1	-2.133856727	0.0479127	0.118547694
CHD2	-2.146178324	0.034135138	0.09216411
TOR1AIP2	-2.160459213	0.00900867	0.033736425
DYNC2I1	-2.166406101	0.04941965	0.121210831
ADAMTSL3	-2.172882839	0.03234096	0.088704959
ZNF2	-2.183508613	0.040033473	0.103495336
CAMSAP2	-2.184851073	0.049680725	0.121727833
NXN	-2.204648422	0.046854254	0.116572227
RAD50	-2.208300218	0.048407054	0.119380397
ZNF148	-2.21234607	0.041955567	0.107272959
PPP2R5C	-2.214265194	0.014959445	0.049730602
S100PBP	-2.218113618	0.024527085	0.072197432
ATP1B1	-2.218278156	0.050292005	0.122976653
ZNF655	-2.220753123	0.014817367	0.049326021
FAM120A	-2.23416475	0.046324297	0.115562509
JUP	-2.250855475	0.039490937	0.102486772
ABCC10	-2.258392979	0.008628767	0.032567069
LARS1	-2.289633374	0.04408369	0.111233892
KANK2	-2.316512648	0.016379232	0.053127941
USF2	-2.318877909	0.035791995	0.095466352
C2CD5	-2.322448232	0.022435543	0.067485043
ACAP2	-2.32329344	0.036021577	0.095783209
EEF1A1P6	-2.323906231	0.023697661	0.070148323
MCM3AP	-2.327049579	0.018594762	0.058521518
TNS3	-2.339700387	0.027805659	0.079576548
NELFCD	-2.35174111	0.041178151	0.105664689
PIDD1	-2.366368106	0.022206842	0.066913521
ZNF268	-2.370968664	0.04339855	0.110009644

SORBS3	-2.371622706	0.015360047	0.050672667
PPM1F	-2.371765457	0.038787725	0.101182461
G3BP2	-2.372420344	0.016965	0.054498714
TNFRSF12A	-2.37877945	0.011860058	0.041658195
MTMR12	-2.396589366	0.042728254	0.108651845
DROSHA	-2.399234399	0.038348407	0.100317511
ARF1	-2.410205243	0.039426045	0.102384219
CARNMT1	-2.411932972	0.028339379	0.080710551
HNRNPH3	-2.414672277	0.024775602	0.072646289
PI4KB	-2.420809832	0.006271813	0.025508743
LIMK1	-2.420955628	0.044539102	0.112055974
TIPARP	-2.443306908	0.043700236	0.11047484
DOCK7	-2.444660273	0.044079782	0.111233892
BTBD7	-2.450540697	0.035572677	0.095090919
SUCLG1	-2.455218654	0.018621098	0.05856699
GRHL2	-2.457623819	0.018771362	0.058882338
ULK1	-2.461490518	0.048235858	0.119103799
VDAC1	-2.46225261	0.048775748	0.120069484
LRPAP1	-2.462816032	0.021813526	0.065969493
NUB1	-2.464390418	0.014439065	0.048265794
PRMT1	-2.466071955	0.039315297	0.102139811
IKZF2	-2.4671861	0.043614946	0.11037326
ZMYND8	-2.47037944	0.045822783	0.114689612
EYA3	-2.47038719	0.047267683	0.117407765
NBR1	-2.470537891	0.027745674	0.079506069
HEATR1	-2.476554485	0.023686314	0.070131884
ISG20L2	-2.47698193	0.020617113	0.06324114
NRBP2	-2.479885519	0.016313389	0.052979961
NEMF	-2.483672528	0.016093482	0.052406646
ATP5MC2	-2.488030884	0.024706016	0.072570554
RAPGEF2	-2.492847419	0.025911164	0.075246817
TRMU	-2.497574357	0.048875026	0.120191652
RPL21P135	-2.499761983	0.029675676	0.083397814
LUC7L2	-2.507501244	0.039961746	0.1033917
LRRC37A3	-2.511383535	0.040359409	0.104137771
TSPAN3	-2.525223151	0.016220618	0.052749694
ZNF587	-2.525878947	0.020505956	0.062948034
BLNK	-2.527408295	0.042533999	0.108322832
HBP1	-2.536845828	0.048485119	0.11954856
DNAJA2	-2.53808788	0.030535187	0.085247604
WSB2	-2.538120892	0.050989259	0.124304933
EHBP1	-2.540971298	0.046666257	0.116200035
CLK4	-2.542443847	0.043380571	0.110009644
RUFY2	-2.543900406	0.050831958	0.123996375

ASAP1	-2.555995872	0.024720522	0.072580512
ZNF334	-2.566339419	0.019833721	0.061461179
UBXN1	-2.567974061	0.008385873	0.031929099
UBN1	-2.573166866	0.043051968	0.109337184
CRYZL1	-2.574837945	0.025559238	0.074385434
TMEM175	-2.579581584	0.03925249	0.102042985
USP25	-2.581771325	0.022966027	0.0686145
NIN	-2.586041741	0.031476334	0.087063426
ZC3H14	-2.587965777	0.021153722	0.064494874
BTG2	-2.591808227	0.029655724	0.083361097
MED14	-2.598516592	0.03158314	0.08721933
ZNF519	-2.599283058	0.033773343	0.091458681
ACAP1	-2.600880196	0.037533191	0.098654015
MCFD2	-2.600881851	0.016733404	0.053996566
UBXN4	-2.603387552	0.014968684	0.049747654
SUZ12	-2.605263785	0.023642254	0.070018556
ANKRD13C	-2.608653229	0.023877114	0.070558737
SIPA1L3	-2.609940174	0.027882564	0.079726426
PDXK	-2.610913247	0.041345608	0.105986983
TTC39B	-2.613205455	0.021311222	0.064828105
FNDC3A	-2.619450866	0.00924914	0.034426698
SUZ12P1	-2.620707178	0.029902865	0.083943059
RECQL5	-2.621169014	0.039065579	0.101666259
PHF20	-2.624509218	0.042200626	0.107717498
PRR14L	-2.624679367	0.013460001	0.045868002
LTB4R	-2.626996229	0.048003558	0.118691889
TMEM98	-2.630382898	0.045204888	0.113448053
MXI1	-2.630582512	0.043638857	0.110387613
LIN54	-2.632137693	0.047765004	0.11828686
RANBP1	-2.632494565	0.038043712	0.099714398
MNT	-2.6396329	0.046265253	0.115439006
NOL8	-2.639760168	0.035000125	0.093996342
TEP1	-2.644706077	0.015890331	0.052010993
DOCK10	-2.645632482	0.048319466	0.119237273
CNOT4	-2.647903935	0.025088778	0.073404535
XBP1	-2.649645429	0.024337151	0.071708102
NSUN4	-2.650753204	0.047251611	0.117391932
CTDSP2	-2.651264024	0.034177374	0.092257567
BFAR	-2.652728948	0.049225821	0.120882398
NDRG2	-2.653188727	0.037106116	0.097850202
MAN1A2	-2.656401316	0.009088573	0.033932169
VWA8	-2.661906247	0.027460419	0.078837978
NUP155	-2.662033695	0.023100946	0.068955326
PSME1	-2.663245395	0.050291761	0.122976653

RBM10	-2.667752671	0.044456343	0.111894276
NOLC1	-2.671598074	0.029438404	0.082904243
ENSG00000225840	-2.671911731	0.000166687	0.001568876
DEGS1	-2.67658779	0.033288123	0.090543695
CYP3A5	-2.677012792	0.003156551	0.014971353
PBRM1	-2.677898586	0.010158468	0.03697988
ARRDC1	-2.683256374	0.038588357	0.100792722
MEF2D	-2.686342811	0.015329147	0.050598308
NAV3	-2.688621626	0.01343213	0.045823705
CLN3	-2.698799075	0.022468013	0.067532877
ZCCHC8	-2.702478396	0.02195835	0.066280267
ZNF783	-2.702824721	0.016795299	0.054181851
MINK1	-2.703417785	0.017621476	0.056217802
VPS45	-2.705774916	0.04716311	0.117244257
PBX2	-2.70815427	0.005236148	0.022316314
CARS2	-2.713089831	0.040697398	0.104700417
PRR13	-2.714985542	0.033008757	0.08994552
ITPRID2	-2.716946686	0.01500762	0.049836003
MBD2	-2.717279798	0.022441803	0.067487102
PACSIN2	-2.718614869	0.02977347	0.083614404
FAM172A	-2.719776352	0.025187873	0.0735829
NCOA7	-2.724110094	0.024647089	0.072470354
ANP32A	-2.728773768	0.022346302	0.067283493
NBPF8	-2.730621764	0.03677785	0.09719631
CTSD	-2.734855668	0.023444421	0.069714462
TEX9	-2.734979903	0.045646963	0.11434413
SHARPIN	-2.738244471	0.039690279	0.102982022
LINC02210	-2.739469652	0.038004702	0.099633726
ZNF394	-2.744679137	0.04766594	0.118130331
USP36	-2.747593729	0.047348788	0.11753686
PRKD3	-2.750706816	0.04023874	0.103892856
IQCN	-2.75330542	0.030111487	0.08438746
ATP6V1D	-2.759592747	0.034452373	0.092813603
ZNF264	-2.771998907	0.017136597	0.054924826
HLA-DQA1	-2.774133498	0.012231464	0.042628746
ARHGEF26	-2.774703473	0.02582465	0.075031579
CDK5RAP2	-2.776050034	0.024990628	0.073152734
PPHLN1	-2.776441894	0.027888441	0.079726426
COQ8B	-2.777534136	0.032962933	0.089901608
TMF1	-2.781383316	0.034731839	0.093400173
MYO1D	-2.781485802	0.032334361	0.088704959
CEP70	-2.782745358	0.01358472	0.046110334
ZNF117	-2.78335495	0.020306496	0.06243074
FAM133B	-2.783521409	0.02791547	0.079784852

ACACA	-2.78473949	0.004697773	0.020483372
REXO2	-2.785928683	0.04610793	0.115165165
ZMAT3	-2.788057979	0.012070443	0.042225607
DAP3	-2.789569841	0.03604269	0.095801874
CEP128	-2.792295567	0.047996826	0.118691889
CD164	-2.793100424	0.018655144	0.058588962
PPOX	-2.795942943	0.046811898	0.116490791
IPO11	-2.797757345	0.041199055	0.105673525
PNPT1	-2.797867248	0.000377568	0.002890625
PPP2CA	-2.802255567	0.035027086	0.094027024
HSD17B12	-2.806988794	0.016912903	0.054435908
DNM1	-2.808716845	0.030997351	0.086092688
TIMM44	-2.808944132	0.036559492	0.096774178
DIP2B	-2.809971359	0.011394873	0.040399396
CCDC28B	-2.811397772	0.037085784	0.097822135
CTDNEP1	-2.816483402	0.03795157	0.099537551
PLD2	-2.816935184	0.034481742	0.092830739
APPL2	-2.821513109	0.046194686	0.115320315
CAPN10	-2.824174775	0.030355509	0.084910501
IGFBP5	-2.825777774	0.035546313	0.095065438
CLCN3	-2.827878917	0.050248898	0.12292091
CBX5	-2.828967705	0.035969225	0.095707078
DYSF	-2.833104677	0.033643744	0.091203557
KXD1	-2.835126116	0.007040764	0.027904847
SYNE3	-2.836624435	0.048764465	0.120066127
PSMG2	-2.839848158	0.045889638	0.114809463
ENSG00000250575	-2.841804842	0.013892742	0.04685365
GRB2	-2.8447483	0.042425218	0.108108388
MCL1	-2.846390921	0.016714759	0.053950786
WDR86	-2.848525621	0.006842285	0.027310733
BCAS3	-2.855940175	0.027314151	0.078455263
RBFA	-2.856072051	0.018102062	0.057433121
SH3GLB1	-2.85879942	0.021728205	0.06578244
PGD	-2.859184011	0.039772449	0.103127378
PIP4K2A	-2.860866827	0.021237801	0.064702327
SMC6	-2.863280851	0.021050441	0.064260919
CNTRL	-2.863331973	0.04030031	0.104029634
ENSG00000259948	-2.865115931	0.049215696	0.120882398
ZNF780B	-2.866151734	0.032455239	0.088872185
R3HDM2	-2.8673257	0.0170072	0.054596838
PPP1R21	-2.868981491	0.019750982	0.061220457
SYNJ2	-2.869012622	0.010327603	0.037415535
RNPC3	-2.874177326	0.005402642	0.022856894
KCTD15	-2.875938822	0.034503698	0.092869192

ARHGAP45	-2.877659132	0.010088723	0.036812797
RRP12	-2.879376593	0.000840413	0.005379357
YWHAB	-2.881503804	0.025651111	0.074599002
GALNT1	-2.881829895	0.008304889	0.03170053
PCED1A	-2.88295868	0.044894268	0.112808847
ZNF189	-2.886546599	0.02793608	0.079811075
ZNF841	-2.891296528	0.002685861	0.013172474
EDC4	-2.894622208	0.034611387	0.093117632
NAPB	-2.895956494	0.015271278	0.050489906
ZSCAN18	-2.897976654	0.001518168	0.00849164
USP8	-2.898965772	0.005373123	0.022773715
SSU72	-2.899169426	0.03412302	0.092151948
ZHX1	-2.903998128	0.011583342	0.040887947
SLMAP	-2.907938698	0.01724218	0.055182271
CSPG4P12	-2.908481323	0.008041109	0.03091791
PPP1R13L	-2.909470917	0.047411167	0.11761934
PLEKHB2	-2.909668684	0.003608508	0.016601057
PDCD2	-2.911004007	0.048027338	0.118691889
RIC1	-2.911679249	0.009414028	0.03491103
RPAP3	-2.912287051	0.044827288	0.112687329
DDAH2	-2.918590083	0.019641378	0.060943151
CLK3	-2.91986306	0.012581067	0.043571169
WBP2	-2.923554743	0.027617031	0.079212451
CORO7	-2.924849104	0.045960049	0.114937642
TCOF1	-2.927502641	0.050729292	0.123770884
PRRT3	-2.928982908	0.029546631	0.08310424
LAMA1	-2.929013689	0.018761838	0.058878219
EEF1A1P3	-2.929452395	0.04498231	NA
BCAR1	-2.930296565	0.014870372	0.049448071
FHIP2A	-2.930635498	0.015425198	0.050818346
TM9SF1	-2.935440558	0.030040363	0.084246653
TAP2	-2.938090414	0.039824074	0.103152277
MARVELD2	-2.940234761	0.040141958	0.103687208
FCHSD2	-2.941082191	0.010396351	0.0375999
ALKBH1	-2.941442689	0.041754586	NA
SEC23A	-2.94868241	0.006113038	0.025005817
KIAA1614	-2.950564334	0.02913542	0.082242332
FGF7P3	-2.951913842	0.032556478	0.089053924
ENSG00000233836	-2.952084493	0.024960328	0.073081715
ARHGEF15	-2.952445009	0.016242237	0.052791633
ABR	-2.955043979	0.017656287	0.056294016
RGS12	-2.957172425	0.024447406	0.072015431
MEF2C	-2.960183981	0.022773389	0.068162488
HSDL2	-2.965397282	0.039472201	0.102460116

MAP1S	-2.966329041	0.020148011	0.062053824
RPL7AP10	-2.96641228	0.043635232	0.110387613
CBFA2T2	-2.967504628	0.017688986	0.056329252
ICE2	-2.968824364	0.020840525	0.063780143
ENTPD1	-2.970203007	0.008068637	0.030994369
USP3	-2.970647063	0.038372647	0.100359232
CUL4A	-2.972956238	0.039288123	0.102091765
OAZ1	-2.975918581	0.002594263	0.012853444
ZNF766	-2.977495332	0.035911865	0.09563266
RAC1	-2.981228584	0.047459595	0.117715355
LAD1	-2.982354635	0.046403148	0.115663861
ATF5	-2.983113233	0.01661504	0.053671855
ARVCF	-2.985520525	0.044419501	0.111840186
FKBP2	-2.987863719	0.040437004	0.104249095
LONRF2	-2.989980565	0.003226798	0.015232903
SEMA4D	-2.993133352	0.007119655	0.028152989
CCDC148	-2.995150214	0.029618141	0.08327479
EEF1A1P11	-2.997423734	0.05048827	NA
DCAF6	-2.999980245	0.008653667	0.032620365
RPS15AP29	-3.003221184	0.014776599	0.049217378
IRF7	-3.00663449	0.033998514	0.091897726
KATNIP	-3.008624943	0.019967172	0.06168521
EXOSC3	-3.013914471	0.029190201	0.082358554
ZNF778	-3.028228119	0.010495774	0.03788871
UCP2	-3.028261873	0.008031876	0.030892222
DIO2	-3.029023374	0.022555912	0.06772268
BRPF3	-3.032924591	0.033119625	0.090207006
PRR14	-3.033005385	0.049876078	0.122107615
IP6K1	-3.033295485	0.02292432	0.068512586
CNOT7	-3.033918808	0.009935484	0.036398032
ZBTB14	-3.036994388	0.015602566	0.051263154
GOLIM4	-3.038504017	0.027944426	0.079811075
MPZL2	-3.041700336	0.021932197	0.066238423
PCDHGC3	-3.042018186	0.029224904	0.082418041
RNF149	-3.043202353	0.032413091	0.088822289
SNRNP35	-3.045856132	0.031242691	0.086575441
DPM2	-3.053140852	0.034407222	0.092733247
SULT1A1	-3.055147608	0.005904489	0.024370004
GNPTG	-3.056861832	0.007773389	0.030127792
PXDN	-3.057481257	0.017009639	0.054596838
MRPL43	-3.059055582	0.029411403	0.082847479
ARHGAP6	-3.061050592	0.014685931	0.04898278
KDM1B	-3.062135758	0.01924181	0.060039279
RNF146	-3.062263533	0.009853548	0.036196463

EZR	-3.063237431	0.035550897	0.095065438
STARD9	-3.06333572	0.026163184	0.075887654
ZNF83	-3.0637863	0.023175334	0.069126229
CD74	-3.066299637	0.00503058	0.021600264
ACCS	-3.067149631	0.033586459	0.091120552
CSPP1	-3.067742266	0.038812566	0.101225447
APEX1	-3.069019329	0.018942258	0.059321369
SAV1	-3.069894082	0.041831649	0.107028734
SRGAP3	-3.074681456	0.031867432	0.087764141
YWHAQ	-3.078105473	0.014698055	0.048996215
PDXDC1	-3.081511706	0.010675343	0.038326521
SELENOF	-3.08438558	0.011894326	0.041766443
DCAF1	-3.087862498	0.019496129	0.060616785
CFAP410	-3.089576503	0.048700619	0.119957732
SLC25A36	-3.08993326	0.039003023	0.101568974
RETSAT	-3.090888364	0.021658838	0.065687441
BET1	-3.094636814	0.038577411	0.100785881
PIN1	-3.095702746	0.033460106	0.090909342
RCSD1	-3.096446171	0.038856771	0.101297082
SPATA13	-3.097011845	0.043280833	0.109780218
CTSO	-3.098321998	0.044679776	0.112362164
SEPTIN10	-3.099546035	0.003265033	0.015359487
FZR1	-3.099729422	0.011409497	0.040403907
ENSG00000260404	-3.102464594	0.006437884	0.026035466
DLST	-3.107005232	0.026577162	0.07677565
KLF12	-3.107549782	0.033007002	0.08994552
FBH1	-3.108317279	0.015409721	0.050781176
UNC5B	-3.114304935	0.028462776	0.080928691
DENND6B	-3.116526541	0.032362703	0.088724384
RTTN	-3.123047822	0.048203889	0.119049148
ARHGEF3	-3.123612468	0.037680384	0.09895495
STXBP1	-3.125532205	0.046654909	0.116195684
POC1B	-3.126955232	0.020840613	0.063780143
UNC45A	-3.128262104	0.014245793	0.047791319
TBC1D10C	-3.130308039	0.013965228	0.04708499
CHID1	-3.132657166	0.042384893	0.108028372
CAMK2B	-3.133078156	0.011765007	0.041396409
PRMT1P1	-3.133625824	0.030487849	0.085150913
ATAD3B	-3.138614663	0.028601161	0.08118866
TRIM24	-3.14450223	0.01205164	0.04217203
CREB3L2	-3.147040607	0.035123976	0.094266209
PDZD2	-3.147241591	0.036250151	0.096242997
COL9A3	-3.151588421	0.03932349	0.102139811
ATXN7	-3.15254449	0.016385451	0.053128717

TRAPPC6B	-3.155010735	0.04900036	0.120450926
ANXA7	-3.156581402	0.016569737	0.053568403
SHROOM1	-3.158887227	0.009345963	0.034698395
MNAT1	-3.159123164	0.034473813	0.092830042
URGCP	-3.159213307	0.005494827	0.023148714
RAB11B	-3.159681628	0.035404155	0.09480794
TCF7L2	-3.15979564	0.034106264	0.092151948
RDH11	-3.160105932	0.036240862	0.096239446
RIC8A	-3.160611528	0.033424386	0.090853081
ZNF606	-3.162681766	0.048373079	0.11934524
ANKRD11	-3.163138718	0.012186558	0.042496716
PRR3	-3.165217857	0.021068998	0.064296544
MICOS13	-3.168099374	0.04578797	0.114626181
ATP6V1A	-3.168499629	0.00338749	0.015830955
EBF2	-3.16952246	0.041426516	0.106122021
SNUPN	-3.169727366	0.033854524	0.091610812
KDSR	-3.173318963	0.011167397	0.039725707
ACOT8	-3.174337221	0.019898306	0.061580151
DDAH2	-3.175670836	0.049530194	0.121432746
SUPV3L1	-3.17665161	0.045035122	0.11306889
FLOT1	-3.177345849	0.029075743	0.082112178
AMPD3	-3.178404608	0.026426849	0.076450904
MAT2B	-3.179894291	0.008530319	0.03229621
SMIM5	-3.181462495	0.036629741	0.096896775
SGSH	-3.184075229	0.00996234	0.03646331
SEMA3F	-3.185940444	0.017659317	0.056294016
PHLPP2	-3.185997879	0.010643555	0.038239711
LRRC37A2	-3.189216829	0.039797219	0.103127378
UBE2J2	-3.191412797	0.013528013	0.046008169
STRN	-3.196876775	0.040702371	0.104700417
NUP133	-3.197146988	0.025466516	0.074206638
TTYH3	-3.198835076	0.031556768	0.087205095
NECTIN2	-3.198924696	0.014708832	0.04901864
CTBS	-3.199924297	0.032926959	0.089864241
SLC33A1	-3.200228604	0.005156944	0.022048625
SLC35E1P1	-3.201698583	0.044354232	0.111753461
TMUB1	-3.206351408	0.035447514	0.094903056
TRABD	-3.207872814	0.048395656	0.119376609
ARAP2	-3.208420839	0.005512869	0.023193525
RNF207	-3.208560046	0.01024659	0.037199977
MYEF2	-3.208963981	0.001027086	0.006297798
SEC11A	-3.209192061	9.14E-05	0.000999243
ITGB8	-3.210296891	0.001701586	0.009249341
KPNA1	-3.210661113	0.020078973	0.061904199

FAM168B	-3.213930264	0.034800356	0.093522095
EPPK1	-3.220977448	0.044373915	0.111779784
NT5C3B	-3.223704265	0.019288318	0.060125111
CCDC125	-3.224261587	0.002363298	0.011973779
DOCK4	-3.224542799	0.016087773	0.052402153
SEL1L	-3.230266994	0.028408078	0.080830129
ARHGAP1	-3.230368625	0.012431302	0.043176035
MAP3K4	-3.237331997	0.009380247	0.034806411
UBA5	-3.237464876	0.012086044	0.042234888
PSEN2	-3.238859677	0.049086561	0.120638321
BBS9	-3.239736045	0.00170435	0.009249341
DDX52	-3.240533865	0.036044426	0.095801874
ZNF540	-3.243861063	0.012896104	0.044471351
GSTM3	-3.252324189	0.012365498	0.043003831
AURKAIP1	-3.254294747	0.012141814	0.042359892
TRAPPC1	-3.254363716	0.026356888	0.076303222
RIPOR1	-3.256897867	0.000582321	0.004062489
FBLN1	-3.257234523	0.013785504	0.04662189
ENSG00000237719	-3.257612123	0.030648267	0.085417136
PFKM	-3.259369726	3.44E-05	0.000468057
LRP3	-3.262549123	0.019907727	0.061580151
POLR2J3	-3.263282238	0.001592117	0.008802352
ZNF419	-3.265432678	0.047402946	0.11761934
NR4A2	-3.266331947	0.022791439	0.068199649
KDELR1	-3.267607733	0.012020319	0.042086763
ARHGAP12	-3.268318742	0.030868731	0.085873391
RINT1	-3.275023559	0.027602164	0.079188574
TMUB2	-3.275386109	0.037281511	0.098140998
SEC13	-3.276954014	0.001327308	0.00767227
LSG1	-3.278061039	0.041922825	0.107211889
MTMR14	-3.282165384	0.00610166	0.024976156
SEC11C	-3.286015869	0.025531533	0.074358439
ENSG00000259924	-3.286646601	0.021967887	0.066292204
MAPK6P3	-3.287917661	0.014428574	0.048257382
EEF1A1P9	-3.28846006	0.001345687	0.007763677
SLC35A3	-3.290170735	0.001365703	0.007852952
RGPD6	-3.293420618	0.02673216	0.07713433
PDE4D	-3.294760486	0.028854406	0.081677985
USP40	-3.295881379	0.003229937	0.015235836
C1orf52	-3.296043499	0.049578853	0.121502822
TRMT10B	-3.300964772	0.013789662	0.046622923
RPS3AP6	-3.302063163	0.01897381	0.059358749
CCDC30	-3.303629289	0.022566704	0.067728088
ENSG00000249967	-3.30381729	0.016965561	0.054498714

REC8	-3.303964163	0.031488808	0.08707803
FAM177A1	-3.308511338	0.007105574	0.02811568
BAHD1	-3.313346909	0.011280546	0.040064475
PCBP4	-3.313572305	0.008361975	0.031868182
ZCCHC7	-3.313887544	0.042373175	0.108021254
NPIPB13	-3.314804775	0.022559306	0.06772268
ACTR8	-3.319556326	0.03130252	0.086681697
SPDYE16	-3.319710302	0.016282559	0.052894282
NDUFS7	-3.324898259	0.009940755	0.036406323
IQCJ-SCHIP1	-3.326633584	0.01593582	0.052098637
MDM4	-3.326701778	0.000173085	0.00161155
COQ4	-3.327016173	0.00322953	0.015235836
CCDC78	-3.329559406	0.038439366	0.100512008
MMADHCP2	-3.330545834	0.013876687	0.046833125
KLHL22	-3.330667675	0.014224841	0.04775801
CBX6	-3.332278252	0.00054094	0.003826734
TAS2R20	-3.336625279	0.040457984	0.10428097
GATAD2B	-3.336673706	0.01208705	0.042234888
ADPGK	-3.338566327	0.032992592	0.089941966
ZBTB18	-3.339979831	0.007928253	0.030590875
MTHFD1P1	-3.34280174	0.044425612	0.111840186
SLC12A7	-3.343025659	0.001697592	0.0092349
CCNB1IP1	-3.343981788	0.000697254	0.004673703
VEZTP1	-3.344084218	0.042251657	0.107802289
CEP135	-3.344452897	0.010080155	0.036805491
HMGB1	-3.345706044	0.002614539	0.012919084
TBCEL	-3.347469123	0.016317688	0.052979961
PHKA1	-3.347518038	0.026774894	0.077217139
ZNF470	-3.350235664	0.006697464	0.026860868
TBC1D22A	-3.352423281	0.004824501	0.020915385
CDH5	-3.35353562	0.042224902	0.10775674
WDR13	-3.35394143	0.005018811	0.021572927
FAM214B	-3.354340758	0.021738097	0.065795931
NFE2L3	-3.357664641	0.016960792	0.054498714
RFX5	-3.358445841	0.01165442	0.041102885
TBC1D22B	-3.358990456	0.0355975	0.095136263
LANCL1	-3.360282698	0.023481978	0.069714462
NPIPB11	-3.360370127	0.001255004	0.00737406
VTI1A	-3.363084921	0.013313287	0.045482366
ZBTB25	-3.365384627	0.044142448	0.11135894
KMT5A	-3.366027725	0.041044896	0.1054087
MBNL1	-3.366185234	0.009530161	0.035233068
TYW1	-3.366296584	0.023608829	0.069984025
HDGFL2	-3.367822863	0.009251485	0.034426698

UFC1	-3.368495973	0.04687186	0.116592065
PIK3C3	-3.370183239	0.028610433	0.081195939
ACBD5	-3.370381433	0.00727261	0.028617579
RRM1	-3.374115561	0.022468184	0.067532877
SPSB1	-3.37671015	0.035815226	0.095507269
NPLOC4	-3.376911984	0.005055868	0.021700788
RRP8	-3.377246647	0.024735179	0.072580512
MRS2	-3.377703127	0.00130881	0.007597643
TMEM231	-3.380603716	0.01944727	0.060511504
MRPL4	-3.381313155	0.023801058	0.070378735
GSTM2	-3.381690089	0.018130612	0.057463883
HBA2	-3.384206639	0.023350815	0.069495517
RREB1	-3.384642929	0.004759598	0.020685881
MUC5B	-3.386338624	0.01619728	0.052687954
TRIM32	-3.388941129	0.031569292	0.087205095
SEC24D	-3.393032976	0.008970384	0.033625746
ADAMTS10	-3.393550426	0.030368271	0.084910501
GMPS	-3.397337732	0.003573243	0.016482673
CNPY4	-3.397601081	0.045501447	0.114026815
SLC43A3	-3.398185288	0.040047762	0.103510168
TMEM267	-3.398533077	0.044952256	0.112931114
TMPO	-3.399758237	0.01613325	0.052512516
RER1	-3.401203389	0.005672763	0.023676939
RBSN	-3.402338502	0.005030663	0.021600264
WDPCP	-3.404367748	7.71E-06	0.000134969
PARP6	-3.404399864	0.00155996	0.008689259
MGAT1	-3.407502925	0.001363032	0.00784505
MBTD1	-3.40757141	0.002539587	0.01262907
TRIM5	-3.408141586	0.009418417	0.034915933
SH2B3	-3.408731231	0.005386934	0.022814363
PPAN	-3.408967159	0.010473721	0.037831668
ETF1P2	-3.409993476	0.022553979	0.06772268
CAMTA1	-3.413508671	0.028023687	0.079999695
CARS1	-3.415533985	0.001506103	0.008459334
CD58	-3.416969464	0.033792566	0.091484058
TRIQK	-3.421218159	0.023032599	0.068768273
CYP4V2	-3.422109138	0.00075749	0.004974855
RNGTT	-3.422527309	0.026131146	0.075812893
CDCP1	-3.42325046	0.006141831	0.025072758
FBXO3	-3.424620471	0.002417001	0.012154292
PABIR2	-3.427004656	0.010233831	0.037164803
AGK	-3.427296758	0.013745835	0.046539746
PCGF5	-3.431664954	0.027075502	0.077954777
SLC37A2	-3.432794739	0.004688131	0.020448699

GSTCD	-3.433502427	0.005530375	0.023218751
MEAK7	-3.435585692	0.03551404	0.09503912
SPEG	-3.437902424	0.005585786	0.023390255
NUP205	-3.440623855	0.032487205	0.088924725
PTENP1	-3.442126149	0.020011265	0.061783391
MCUR1	-3.442428881	0.002244397	0.011486756
HSPB1	-3.442480498	0.011140449	0.039659999
HSP90AB3P	-3.443244306	0.044160937	0.111359162
ENSG00000228981	-3.44561062	0.047244808	0.117391932
CXADR	-3.445916926	0.013451978	0.04586556
DCTN3	-3.448181298	0.010627498	0.038238774
SCAMP1	-3.448864441	0.011522143	0.040755119
TAS2R3	-3.450391291	0.04183544	0.107028734
TRIR	-3.451353849	0.019467831	0.060559916
KATNA1	-3.451720729	0.037587366	0.098753523
PELP1	-3.451842405	0.00761232	0.029621295
COPB1	-3.454120652	0.006117636	0.025009078
RYK	-3.455746273	0.005604272	0.023438435
NPIPP1	-3.456687638	0.013972589	0.04709669
ITPK1	-3.459738035	0.00671477	0.026921358
LONRF1	-3.45996753	0.001501485	0.008437316
SLC39A11	-3.460308525	0.038186247	0.100001369
UBOX5	-3.462545119	0.031820503	0.087693358
SMURF2	-3.463491022	0.024046034	0.071023229
GSAP	-3.463558381	0.005696783	0.023736624
MTX2	-3.464845823	0.011385876	0.040391163
GLIS2	-3.465785653	0.007570792	0.029513543
DPH7	-3.465836666	0.003412556	0.015929647
CREB3L4	-3.473248213	0.046088509	0.115140415
ABITRAM	-3.473524741	0.027762823	0.079517559
MTHFD1	-3.47367155	0.010107158	0.036848506
GRHL1	-3.475052977	0.009676723	0.035676836
NUDCD3	-3.476662656	0.014839398	0.049372204
DHX57	-3.4789319	0.015446804	0.050834182
ZNF687	-3.479162068	0.000829287	0.005322208
ZNF252P	-3.480666291	0.001694751	0.009223588
LRP5L	-3.482277152	0.008503471	0.032255096
METAP2	-3.482984975	0.014401703	0.04818082
DCLRE1C	-3.483747566	0.037158255	0.097958546
GOSR1	-3.48399152	0.000326684	0.002625618
SLC25A45	-3.484019192	0.011977401	0.04197292
AP3B1	-3.484104647	0.004370063	0.019382646
NENF	-3.485100572	0.043437542	0.110085428
ENSG00000234268	-3.485509914	0.023341761	0.069485656

KREMEN1	-3.485947664	0.045988351	0.114937642
ZNF677	-3.486324505	0.019418424	0.06045283
SSPOP	-3.487740571	0.023985572	0.070861938
SLC16A7	-3.488503928	0.032272336	0.088584047
PHF21A	-3.490446044	0.022424572	0.06747169
NFX1	-3.495566111	0.004638529	0.020242492
TRRAP	-3.496227921	0.013563946	0.046065658
LSM14B	-3.496534999	0.031526517	0.087162395
MMAA	-3.499014716	0.030685271	0.085500581
SUGP1	-3.501043138	0.015444606	0.050834182
PINK1	-3.502139518	0.005809889	0.02405024
DENND10	-3.502965279	0.021331118	0.064858812
CENPV	-3.504977994	0.033196535	0.090355489
MGME1	-3.506452124	0.036293086	0.096272521
TMEM126A	-3.507220327	0.014301963	0.047939896
FAM184A	-3.50723561	0.015128322	0.05014053
SLC7A11	-3.507554214	0.038254251	0.100157788
TBC1D19	-3.507829866	0.029974331	0.08411994
ZCCHC2	-3.508090735	0.0030066	0.014446959
ATR	-3.508659222	0.038524274	0.100707736
ZNF93	-3.509459832	0.010937338	0.039109464
TNFSF12	-3.509830138	0.043495848	0.110182139
MYL12B	-3.511505514	0.015686462	0.051510835
IDH3A	-3.511718041	0.017118373	0.054888381
RHOF	-3.512996021	0.04751464	0.11782774
AXIN1	-3.513164878	0.023506824	0.069771113
MPPED2	-3.513764028	0.042954796	0.109136198
TGFB1I1	-3.51489156	0.005533703	0.023224668
ANKRD37	-3.515107659	0.046484283	0.115794559
COG8	-3.516168308	0.00528394	0.022488328
CRIP1	-3.516529053	0.037673098	0.09895495
THEMIS2	-3.521568351	0.014160839	0.047625116
OTUD4	-3.524076642	0.013422362	0.045803291
RBM45	-3.525174914	0.050409046	0.123213065
AKR1B1	-3.528864995	0.000364656	0.002816715
YY1	-3.529423776	0.006524256	0.026288147
WWC1	-3.530413868	0.000407034	0.003063893
ATXN10	-3.531714785	0.001615911	0.008882372
HSCB	-3.533542211	0.032643112	0.089230404
BUB3	-3.533767865	0.030076682	0.08430944
COMMD9	-3.535648463	0.031827241	0.087693358
CLCF1	-3.535730038	0.028694641	0.081415831
TMEM132A	-3.539039664	0.007965417	0.030695131
PREX2	-3.539968485	0.02242553	0.06747169

PFKP	-3.542049312	0.01854942	0.058408474
TMEM140	-3.542940988	0.030793408	0.085703245
ANKRD42	-3.544333204	0.007605137	0.02960842
TOP1MT	-3.546381483	0.035523716	0.095044
C8orf44	-3.55286974	0.033367751	0.09071951
ERCC6L2	-3.553165952	0.003749176	0.017082356
PDGFC	-3.554539195	0.032361638	0.088724384
BCR	-3.55537219	0.000448798	0.003306297
RNPS1	-3.556146192	0.010817371	0.038714802
ZNF415	-3.557580896	0.001650214	0.009025843
TNFAIP1	-3.557613268	0.030276148	0.084770413
OPTN	-3.559016314	0.005605938	0.023438435
COA5	-3.559546107	0.028533186	0.081058155
MAGED2	-3.559871687	0.004009447	0.018020924
ARHGEF9	-3.560660114	0.02914327	0.082245312
BAD	-3.563871734	0.031289943	0.086666698
USP5	-3.564985164	0.000409654	0.003079781
DDX23	-3.565249036	0.001456111	0.008243578
DHX40	-3.565733527	0.00843617	0.032050033
ZFTA	-3.565825826	0.026629692	0.076890694
INPP5J	-3.566311023	0.001607871	0.008862329
OSTCP8	-3.567096393	0.036819214	0.097284385
TCN2	-3.568248952	0.011849075	0.04163258
DEK	-3.568285637	0.001088302	0.006583109
MIGA1	-3.569261554	0.002895136	0.014000292
ZNF692	-3.576925892	0.013582132	0.046110334
ETV5	-3.577294375	0.017398119	0.055578474
MRPL52	-3.579878101	0.015020663	0.049838296
DPRXP4	-3.579940353	0.022670846	0.067956394
ERMARD	-3.58220136	0.028843443	0.081677985
IFT88	-3.58366033	0.024707299	0.072570554
IMMP1L	-3.58420286	0.040491414	0.104328158
SPNS1	-3.586015736	0.002930729	0.014149799
ISLR	-3.58722273	0.011468262	0.040588259
PBX4	-3.587361996	0.035321181	0.094634283
LCOR	-3.589810313	0.002386543	0.012066296
EIF3EP1	-3.591187133	0.030253379	0.084726262
GALNT11	-3.594746094	0.00769609	0.029866313
TP53INP2	-3.594798256	0.028800633	0.081601794
HLA-A	-3.595513553	0.01778642	0.056550256
CORO1A	-3.599729061	0.024683309	0.072551427
MTRFR	-3.601647337	0.00220671	0.011346651
RNF43	-3.601716624	0.035633713	0.095168465
ACTG1	-3.603497084	0.009897789	0.036336926

DLC1	-3.604559744	0.004088703	0.018309163
RMDN1	-3.605871574	0.001910191	0.010145218
EVI2A	-3.606543674	0.02311571	0.068982387
MVP	-3.606671806	0.013666062	0.046308516
API5	-3.609117572	0.008358761	0.031865968
DAPK1	-3.610493535	0.035193891	0.09439106
MRPL10	-3.611217344	0.0336847	0.091294136
TM2D1	-3.611251395	0.000594143	0.004125936
GAS6	-3.612592441	0.003896486	0.017631054
NPIPB9	-3.612740426	0.005431851	0.022956398
NOXA1	-3.61437446	0.00571957	0.023764564
NAA10	-3.618462071	0.018963361	0.059341398
SPSB3	-3.623829604	0.01853314	0.058373515
TEX261	-3.624439874	0.016630642	0.053707922
EIF4BP6	-3.624448984	0.018204404	0.057606824
PRRT2	-3.624715213	0.028392543	0.080830129
ACAD8	-3.625109153	0.002407967	0.012129342
C1orf112	-3.626255702	0.001791502	0.009606418
NCAPD2	-3.627456774	0.005083743	0.021774107
CAMKK1	-3.629966734	0.001273591	0.007445466
PAPOLA	-3.630900094	0.003376972	0.015787899
TBRG1	-3.631393864	0.000688794	0.004639486
MRPL35P3	-3.634403898	0.025206378	0.07358852
RBFOX2	-3.636353083	0.019350096	0.060302153
LYPD5	-3.638573218	0.03237066	0.088726103
ALDH2	-3.638945046	0.00934088	0.03469224
SPATS2L	-3.638993056	0.00608525	0.024925842
C1orf159	-3.64034023	0.047848304	0.118448095
AZI2	-3.645315879	0.008331618	0.031772496
FLYWCH2	-3.64532712	0.015938636	0.052098637
STEAP2	-3.646770471	0.001790521	0.009606418
EIF2B5	-3.646879227	0.007168424	0.028326794
TPD52L1	-3.650409068	0.00892485	0.033499338
AP1M1	-3.650477317	0.007022762	0.027870004
CDC73	-3.651973262	0.011168753	0.039725707
CSPG4P13	-3.652228647	0.025192295	0.0735829
XRCC6	-3.652302851	0.001001927	0.006174811
RPA2	-3.652444443	0.018409336	0.058118571
B4GALT6	-3.655636417	0.021711764	0.065765563
SCRN3	-3.656221587	0.002460466	0.012342097
CGRRF1	-3.657760436	0.049754546	0.121867786
IAH1	-3.658241784	0.015629985	0.051339304
DGKQ	-3.65853167	0.00818002	0.031322669
CPT2	-3.659570039	0.010609631	0.038185839

FAM102B	-3.661430271	0.050049333	0.122507002
DDX18P5	-3.661962309	0.031313886	0.086693339
SIL1	-3.662459865	0.016531711	0.053459745
ECT2	-3.663207317	0.001681988	0.009162369
TLR1	-3.66408051	0.04447924	0.111928633
DTYMK	-3.664119746	0.033475376	0.090921301
ZNF468	-3.664895602	0.007427273	0.029056145
EGFL7	-3.665551895	0.001516535	0.008486426
PBX2	-3.66580309	0.003563394	0.01644978
PACRGL	-3.666283116	0.015967105	0.052135377
ZNF45	-3.667221386	0.018135495	0.057463883
PHF23	-3.667853327	0.001120367	0.006736644
CHRAC1	-3.668089903	0.031240283	0.086575441
SULT1C2	-3.668304609	0.005221088	0.02227566
MSS51	-3.668475511	0.031862163	0.087764141
TFIP11	-3.669224728	0.046208157	0.115320315
SMARCD1	-3.669719769	0.003970127	0.017910702
ABCC8	-3.670014586	0.045664644	0.114353119
CEP192	-3.670759525	0.003762394	0.017126746
PAFAH1B2	-3.671386071	0.016882492	0.054390653
ZNF266	-3.674456205	0.002038327	0.010625287
SOD2	-3.674663213	0.004458608	0.019674441
HNRNPRP1	-3.675891974	0.005082595	0.021774107
COL7A1	-3.676275489	0.000174558	0.001621527
ENSG00000272101	-3.6772171	0.035640999	0.095168465
ENSG00000240265	-3.677622417	0.023472371	0.069714462
STAG3L4	-3.678031805	0.040707218	0.104700417
RTCA	-3.679006874	0.013052172	0.044919958
NF2	-3.680964632	0.005503054	0.023160282
R3HDM4	-3.681446127	0.027942082	0.079811075
PDGFB	-3.681664709	0.024257475	0.071543002
ZNF19	-3.682758258	0.019955878	0.061681805
HEXB	-3.682817387	0.005502863	0.023160282
PSMD9	-3.683665598	0.008830912	0.033185147
RAN	-3.686785056	0.001003521	0.006180076
RDH5	-3.68707678	0.028112046	0.080192555
ZNF791	-3.6905422	0.003441793	0.016023204
AEBP2	-3.690951082	0.009123291	0.034051285
GPR87	-3.691107044	0.040432073	0.104249095
ETHE1	-3.692290914	0.040663371	0.104654359
FTH1P11	-3.695584468	0.012994556	0.044747112
DUSP5	-3.69568935	0.020197179	0.062173614
RELA	-3.69616377	0.000494754	0.003560344
ZRANB2	-3.698029106	0.001170336	0.006989652

DYM	-3.698338068	0.004905745	0.021237172
GPALPP1	-3.699956994	0.031542538	0.087186773
METTL18	-3.702522058	0.048716988	0.119973635
SLC27A5	-3.703016554	0.00446299	0.0196866
PAXX	-3.704841276	0.007631318	0.02966816
UEVLD	-3.705216792	0.00928863	0.034519365
ZNF483	-3.705455188	0.006244714	0.025424155
LZIC	-3.705629477	0.019245902	0.060039279
GTF2B	-3.70571327	0.04898663	0.120441635
CLDN1	-3.706740203	0.024276184	0.071563305
FAAH	-3.707084514	0.023601438	0.069984025
PCDHA6	-3.707302518	0.029364167	0.082733676
METTL26	-3.708770467	0.000786181	0.005137541
SLC25A27	-3.70945824	0.028194473	0.080373504
GNB5	-3.710596396	0.000311955	0.002538288
DCP1A	-3.712751146	0.006604496	0.026567238
SENP2	-3.713177948	0.007037852	0.027904847
DGUOK	-3.714381052	0.016712538	0.053950786
CCDC142	-3.714890652	0.03628779	0.096272521
MBTPS2	-3.716150967	0.049380005	0.121187262
IL1R1	-3.717244582	0.001232129	0.007271421
CSNK1G1	-3.717689165	0.011551993	0.040801085
TIE1	-3.717699838	0.009915379	0.036357389
PIK3R3	-3.718126525	0.006407887	0.025931481
ZEB2	-3.719056938	0.006813004	0.027216038
ABLIM3	-3.721078882	0.045301701	0.113643895
CRMP1	-3.7222499	0.024634092	0.07245955
UBALD1	-3.722535044	0.032975338	0.089915183
SARNP	-3.722684556	0.030607725	0.085363111
SEMA4C	-3.722831493	0.000248124	0.00213454
PTGR2	-3.723776397	0.006440059	0.026035562
RPS7P1	-3.724547455	0.05043753	0.123257797
ACKR4P1	-3.725005828	0.04049353	0.104328158
ENSG00000232027	-3.725546524	0.033129152	0.090212657
XPO5	-3.725738407	0.000714203	0.004757682
EIF2AK3	-3.727646358	0.013626082	0.046185969
ZNF525	-3.728749606	0.032328646	0.088704959
FADS1	-3.732169822	0.017499543	0.055887723
FANCM	-3.732897043	0.021168185	0.064506474
ANO1	-3.733198528	0.001588179	0.008793645
ENSG00000236583	-3.734779875	0.032906447	0.089838693
MRPL30	-3.735771745	0.01058432	0.038117408
RAP1B	-3.73629812	0.004546869	0.019918677
PAXIP1	-3.738457694	0.003731794	0.017042129

NICN1	-3.741318136	0.02764682	0.079260328
ENSG00000251288	-3.742269157	0.02361903	0.069984025
SUMO2	-3.742411635	0.008518746	0.032272582
RIT1	-3.742607305	0.036616268	0.096896221
RALBP1	-3.74565574	0.023724221	0.07019261
ZWILCH	-3.746095978	0.028854516	0.081677985
B4GALT4	-3.747343502	0.035995389	0.095734606
LCMT2	-3.750783478	0.043968467	0.110989431
IL1RL2	-3.751176728	0.015396676	0.050752006
ARL17B	-3.754560873	0.0106354	0.038238807
NR4A3	-3.756795112	0.018856678	0.05909923
LYPLA2	-3.757444787	0.010340232	0.037438876
SHMT1	-3.75930953	0.001432012	0.008160581
SNAPC4	-3.76092954	0.0045745	0.020032469
TTC9C	-3.762097044	0.002090503	0.010845887
AMT	-3.762398012	0.001681469	0.009162369
IFIT3	-3.763230313	0.035310408	0.094634283
LRRFIP1P1	-3.765883355	0.023219417	0.069189517
DUT	-3.765895386	0.004621328	0.020186414
USP47	-3.766117697	0.01017996	0.037018869
WNT3	-3.766122299	0.033479531	0.090921301
KIAA0895L	-3.766578009	0.007047136	0.02792096
METTL17	-3.767232995	0.010814186	0.038714802
RBMXL1	-3.76727986	0.004526906	0.01986573
PAPOLG	-3.768790381	0.011952382	0.04190951
TPM3P9	-3.768894668	0.033944473	0.091813163
PALM	-3.769573146	0.015314277	0.050563013
GATM	-3.771460718	0.033316254	0.09059985
PDIK1L	-3.771878693	0.035323669	0.094634283
PBX2	-3.773182929	0.025683655	0.074654857
SNPH	-3.773755538	0.017601091	0.056167572
MYO1E	-3.773967936	0.003297999	0.015478474
CDK9	-3.77574088	0.010726433	0.038452781
BNC2	-3.776199498	0.037761997	0.099083288
ZNF512	-3.77749	0.003353263	0.015707207
MCRS1	-3.777548964	0.001259951	0.007385204
GRAMD4	-3.777651804	0.036083946	0.095864811
FAM98C	-3.778311249	0.000335393	0.002672548
CDAN1	-3.778617769	0.015894635	0.052010993
QRSL1P3	-3.782871906	0.010063745	0.036766669
USP21	-3.784145451	0.004395085	0.019457979
POLB	-3.786966624	0.000558145	0.003925167
TBC1D7	-3.786988874	0.030291753	0.084774884
DUSP4	-3.789153128	0.025316322	0.073820468

EPC1	-3.789399675	0.003677432	0.016866859
PLEKHB1	-3.791071135	0.042611182	0.108445278
ATF1	-3.795025135	0.013773707	0.04659501
TRO	-3.796915972	0.00710869	0.028118817
CXXC1	-3.797065705	0.00758962	0.029576549
PDGFRB	-3.799337559	0.000853911	0.005451339
FEZ2	-3.801516568	0.007461291	0.029160951
EIPR1	-3.80259485	0.013214488	0.045272618
ZFYVE16	-3.804048926	0.000144628	0.001416324
OGFOD3	-3.804516138	0.004340661	0.019273425
VPS13B	-3.804553516	0.021255307	0.064739366
ENSG00000146722	-3.805183126	0.037194638	0.097998237
SELENOO	-3.805284634	0.007009301	0.027834835
BEX3	-3.806188352	0.012494654	0.043363942
ATP5F1C	-3.806705028	0.000830204	0.005325274
RAP1A	-3.807698909	0.020762579	0.063622849
CDK20	-3.809709437	0.01020215	0.037071997
ZNF554	-3.809739858	0.001310554	0.007600837
ABHD16B	-3.811128371	0.02370892	0.070164492
PIGX	-3.811204408	0.027092772	0.077985947
CIAO2B	-3.811925004	0.046203457	0.115320315
PPP1R11	-3.812338611	0.050983367	0.124304933
TPRN	-3.812915135	0.03655124	0.096774178
ADORA1	-3.812949582	0.036066834	0.095840386
DTNBP1	-3.814231698	0.014362695	0.048076896
TRIM3	-3.814484085	0.003427488	0.01598702
TTC1	-3.81511569	0.003993207	0.017989865
HACE1	-3.816099659	0.004070181	0.018241795
ELK4	-3.816750161	0.001224654	0.007237894
RIPK1	-3.816915429	0.007423085	0.02904915
CIAO1	-3.817900603	0.004976349	0.021458401
RPSAP21	-3.819313832	0.04547593	0.113986468
LEMD2	-3.821667462	0.005474013	0.023097508
REEP4	-3.82322788	0.041383458	0.10605661
SLC6A12	-3.823328765	0.03193239	0.0878761
UBE2Q1	-3.823987243	0.00844682	0.032080423
SLC22A23	-3.824792454	0.013154659	0.045195569
PAG1	-3.825946684	0.007469202	0.029173029
ICMT	-3.826394635	0.009902184	0.036342035
SLC24A5	-3.827172708	0.034239428	0.0923597
ZNF850	-3.827255333	0.015812179	0.051839277
ALDH1A3	-3.827485239	0.025224937	0.073607194
TBC1D31	-3.831949935	0.034796306	0.093522095
HNRNPA1P76	-3.833013155	0.013112527	0.045071215

PIK3R1	-3.835181038	0.003767288	0.017142575
GUCD1	-3.83569329	0.003217212	0.015206009
CREB5	-3.839569755	0.05065157	0.123680978
HMG20A	-3.840861773	0.037716646	0.099007217
ENSG00000259232	-3.841633846	0.048152166	0.118945678
VAMP2	-3.842317828	0.000131037	0.00131297
ROR2	-3.84373489	0.044970131	0.112952576
EGR3	-3.844652202	0.01699613	0.054582424
ENSG00000179038	-3.845555662	0.037163388	0.097958546
USHBP1	-3.846326468	0.006019623	0.024715577
PCDH18	-3.848762896	0.020014266	0.061783391
ENSG00000275756	-3.848825699	0.034999191	0.093996342
SAMD9L	-3.850303202	0.005170162	0.022097329
SPTBN2	-3.851740769	0.042536221	0.108322832
SMN1	-3.852011442	0.037183706	0.097990764
ZNF250	-3.853168965	0.011786412	0.041459671
C21orf91	-3.855280373	0.043677449	0.11046215
OAF	-3.855798134	0.038747943	0.101100815
CMC2	-3.856031536	0.042012034	0.107349304
PLEKHA3	-3.856093342	0.003932779	0.017768702
ITGB3BP	-3.857146413	0.016225214	0.052750468
AP4B1	-3.860080651	0.024077452	0.071098678
MKI67	-3.86078185	0.035700075	0.095284168
ZNF713	-3.860954115	0.009719165	0.035811498
MDH1B	-3.861752669	0.027828661	0.079611939
PNPLA7	-3.862434708	0.006850373	0.027329238
KCMF1	-3.866576689	0.02128902	0.064793134
PIAS2	-3.867577847	0.000282471	0.002351302
DPH2	-3.869953536	0.014628434	0.04881791
MAEA	-3.873560977	0.001639969	0.008977919
FAM120AOS	-3.873877271	0.018166249	0.057501119
ST3GAL2	-3.874208381	0.005930919	0.024459233
RECK	-3.875550239	0.029904368	0.083943059
WRAP53	-3.875938587	0.039279328	0.102090828
ITPKB	-3.879340073	0.003139541	0.014916279
YEATS4	-3.879771371	0.032890036	0.089838693
TNFRSF21	-3.880712641	0.016066314	0.052360437
FGF11	-3.882324014	0.04374845	0.110572403
U2AF1L5	-3.883176657	0.002068718	0.010746679
UPP1	-3.883881493	0.020176857	0.062126857
CYP2R1	-3.8855143	0.031393701	0.086874567
POGLUT3	-3.886593985	0.00579183	0.024008324
SMPDL3B	-3.886959171	0.026191146	0.075950559
TBC1D4	-3.887495488	0.015141716	0.050171183

KLF13	-3.888890816	0.022762208	0.068162488
RAD17	-3.889439825	0.015469042	0.050893527
CAMK1	-3.894069282	0.027786276	0.079547088
KDM2B	-3.897107555	0.001443181	0.008197213
VPS9D1	-3.897992089	0.031080817	0.086205824
SRP68	-3.90076447	0.001818212	0.009720687
NLRC3	-3.901634443	0.020883709	0.063864681
IP6K3	-3.90271388	0.038748074	0.101100815
ZNF430	-3.902836135	0.031091554	0.086209817
THUMPD2	-3.905435515	0.016514012	0.05343106
NCR3LG1	-3.906107284	0.032535466	0.089016565
FBXL20	-3.908145478	0.00132278	0.007649751
TERF2	-3.909112444	0.007829695	0.030307205
SERPINA1	-3.911683072	0.025160049	0.073541954
TAFA2	-3.912571378	0.032910176	0.089838693
EOLA1	-3.915083319	0.029487284	0.083022583
VARS1	-3.915227065	0.040750308	0.104766721
SNX33	-3.915548426	0.012963864	0.044665882
LSM4	-3.91748462	0.035243864	0.094504149
RNLS	-3.92040746	0.020110299	0.061953438
CWC25	-3.920576252	0.000150877	0.001463316
QSOX2	-3.92212983	0.02655231	0.076722169
AACS	-3.922423148	0.006185823	0.025218322
KNTC1	-3.926707247	0.002289253	0.011652277
HMGB1P10	-3.927004907	0.037527841	0.098654015
CDHR5	-3.928063216	0.014310087	0.047953847
RPL29P11	-3.928849421	0.020820193	0.063767111
IL13RA1	-3.9302738	0.000626353	0.004311584
HGS	-3.930605041	0.0010778	0.006541063
PNMA8A	-3.931091701	0.038079016	0.099742137
NEK6	-3.932380275	0.010294076	0.037327591
PECR	-3.934882641	0.011746031	0.041353681
RANBP10	-3.936792039	0.020975185	0.064079668
MOB4	-3.93819249	0.033835493	0.091579787
NRCAM	-3.938803826	0.007339969	0.028817058
HERC6	-3.938845564	0.029006375	0.082011952
CDK17	-3.939049464	0.004783233	0.020766232
ZNF141	-3.939218486	0.014352085	0.048054671
TIGD6	-3.939735074	0.038741341	0.101100815
ARMC8	-3.940222747	0.00059072	0.004106881
STRN4	-3.941396087	0.001079441	0.006544595
RAD17	-3.941941516	0.007308488	0.028721408
SCD5	-3.945254777	0.001350074	0.007785278
TOMM22	-3.945341654	0.026781027	0.077217139

E2F6	-3.945642783	0.019508898	0.060640908
OXA1L	-3.945842623	0.031622452	0.087268161
EPHB6	-3.945997406	0.031065252	0.086202157
SLC48A1	-3.946339044	0.002046126	0.010661349
RASSF7	-3.94652203	0.030920879	0.085939454
ERO1A	-3.946807505	0.001257299	0.007375924
ENSG00000283145	-3.947358225	0.046702068	0.116265289
IL18	-3.948270504	0.031080267	0.086205824
DNAJC18	-3.948761572	0.037208374	0.098013092
NAV1	-3.949952447	0.00048459	0.003505963
TMEM41A	-3.952733602	0.002669915	0.013115526
TLCD5	-3.952923457	0.01651089	0.05343106
TICRR	-3.952945528	0.022871464	0.068405288
SLC22A5	-3.955838007	0.011932814	0.041853024
FTO	-3.956067325	2.17E-05	0.000322757
NADK2	-3.957003483	0.008498961	0.032248097
PALMD	-3.957815217	0.047899149	0.118538398
MRPL19	-3.958567556	0.010023712	0.036654684
BRD2	-3.961963005	0.019545783	0.060708789
CPM	-3.962073491	0.010572952	0.0380878
SNX5P1	-3.962436784	0.040006904	0.103448743
CCDC12	-3.963142425	0.001426211	0.008131348
PDE3A	-3.965118893	0.045414368	0.11385574
FGF1	-3.966916004	0.039218647	0.101994108
DDX10	-3.966926368	0.021391333	0.065022775
C11orf68	-3.967397484	0.005077849	0.021771976
AGTPBP1	-3.969536591	0.005083136	0.021774107
MRI1	-3.970956193	0.011403969	0.040403907
CNOT10	-3.971006246	0.013870414	0.04683054
ZPR1	-3.974238789	0.006345019	0.025737182
FAM217B	-3.974476645	0.005217506	0.02227566
FAM47E	-3.975946425	0.024314887	0.071659944
PRPF19	-3.976088303	0.035290546	0.094608365
TRIM26	-3.977841478	0.048825329	0.120167098
CYP24A1	-3.977864274	0.038293656	0.100217603
ZW10	-3.979304155	0.013511834	0.045966059
DCAF7	-3.983095146	0.014169809	0.047642045
CS	-3.983661649	0.001176465	0.007018204
TMEM63A	-3.984041314	0.004729316	0.020598646
HTD2	-3.984063775	0.014810891	0.049318025
TRIM37	-3.985464466	0.007981023	0.030735697
SRP9P1	-3.987239913	0.028404446	0.080830129
RALGDS	-3.987804319	0.001883101	0.01002774
YEATS2	-3.98806735	0.027111216	0.078001939

TUBGCP5	-3.98982241	0.020837201	0.063780143
EGFL8	-3.991038824	0.017183969	0.055038217
ZER1	-3.991149444	0.006013288	0.024714715
CHTF8	-3.991893144	0.018132418	0.057463883
PIPSL	-3.992616065	0.0451272	0.113276573
HNRNPA1P59	-3.992664243	0.037705878	0.099000423
EIF4BP7	-3.992774058	0.047313409	0.117473129
OSGEPL1	-3.993780564	0.021165875	0.064506474
PCDHGB7	-3.994884348	0.000337933	0.002680431
ZNF350	-3.996436427	0.044407157	0.111840186
ENSG00000279208	-3.997593779	0.016590157	0.053605782
RNF181	-3.997790112	0.021628227	0.065643947
C1QC	-3.99834176	0.015565563	0.051169357
IMPA2	-3.998460638	0.02419307	0.071387839
FAM186B	-3.999919234	0.021635586	0.065649821
HAAO	-4.000379082	0.030771826	0.085702296
MAPK8IP1	-4.000935518	0.018264557	0.057782069
ZNF701	-4.001528975	0.04023561	0.103892856
NTAN1P2	-4.001669285	0.03374463	0.091436088
NUDT22	-4.001714874	0.002814195	0.013712968
SEZ6	-4.005617799	0.045591179	0.114228034
POLR1E	-4.007707477	0.033615383	0.091160062
YWHAZP5	-4.007990142	0.016012432	0.052255184
SCNN1D	-4.009799718	0.031763554	0.087594421
SLC25A44	-4.011185091	0.008402549	0.031956252
ZC3H12B	-4.011548526	0.003050209	0.014598548
EME2	-4.013798343	0.011393112	0.040399396
EIF1B	-4.015237244	0.029550615	0.08310424
MAML3	-4.016733111	0.000460036	0.00336859
UTP4	-4.018599178	0.00472883	0.020598646
USP33	-4.019179121	4.95E-05	0.000617444
SRP14	-4.020902248	0.003731299	0.017042129
SNRNP48	-4.021094147	0.030238337	0.084703732
RPL7P21	-4.022033594	0.041707782	0.106774744
GLT1D1	-4.022884205	0.049124015	0.120705862
TERF1P7	-4.023309111	0.019008062	0.059435179
CAMKMT	-4.024830991	0.036382479	0.096446239
ID1	-4.026541769	0.029550157	0.08310424
VPS52	-4.026876628	0.001709643	0.00927129
ELAC1	-4.027554686	0.018806096	0.058955965
PPM1K	-4.02924962	0.013884196	0.04683788
ZNF507	-4.030037373	0.015017761	0.049838296
JPT1	-4.030150421	0.023618439	0.069984025
PCDHA14	-4.031987068	0.019537117	0.060697449

EMC4	-4.032385467	0.004118679	0.018395752
DDX50P1	-4.034687011	0.003211268	0.01518921
TMCC1	-4.037274322	0.001797928	0.009629255
ZNF844	-4.037346382	0.012965396	0.044665882
FBLN7	-4.037437003	0.036935563	0.097527934
KRT18P15	-4.037682121	0.010283923	0.03730195
CLBA1	-4.040264075	0.014473638	0.048368007
LHFPL2	-4.040279496	0.026931004	0.07761259
CHTF18	-4.041045672	0.003589174	0.016531098
FAM126B	-4.04151227	0.000327862	0.00262865
HAUS7	-4.044033996	0.019430425	0.060474636
SLC50A1	-4.044888488	0.000156152	0.001496487
SLC35A2	-4.045278178	0.004005324	0.018009078
NAPRT	-4.045746407	0.021573784	0.065495131
GNAL	-4.048316864	0.030486118	0.085150913
ZNF454	-4.049146243	0.013273812	0.045411593
EPM2A	-4.050240708	0.023429581	0.069695661
EPHA2	-4.051350941	0.003076361	0.014688867
HOMER3	-4.05313957	0.003806288	0.017285564
VWA7	-4.056023949	0.00471722	0.020560759
TAF5L	-4.057190386	0.034760051	0.093455276
HNRNPA1P10	-4.057360461	0.018328816	0.057897544
USP51	-4.058118949	0.036968288	0.097571775
RORA	-4.059170349	0.000216644	0.00192248
HVCN1	-4.060671344	0.019689289	0.061060505
ATP5MF	-4.062954003	0.000824372	0.005299094
ZNF708	-4.063854175	0.000255059	0.002174108
ZNF114	-4.063900191	0.01328829	0.045435442
GRAMD1C	-4.0645673	0.011385656	0.040391163
CD101	-4.064600258	0.034682486	0.093288181
ABCA13	-4.065835815	0.015745959	0.051678169
STMP1	-4.066045145	0.01863847	0.05856699
ZCWPW1	-4.066287525	0.013460273	0.045868002
EEF1A1P12	-4.066784543	0.000365258	0.002819566
COMMD7	-4.066846783	0.013449808	0.04586556
CCHCR1	-4.067132905	0.015300287	0.050551228
AGAP14P	-4.06882953	0.020396928	0.062692843
ADGRL2	-4.069736254	0.001566688	0.008714699
LINC01881	-4.071990588	0.020042	0.061837462
CCDC90B	-4.072945626	1.99E-05	0.000301348
KNOP1	-4.074265648	0.002849506	0.013818275
PKNOX1	-4.079494191	0.001673283	0.009135508
FAHD2A	-4.082145862	1.00E-04	0.001058744
ZFP1	-4.082399157	0.031937125	0.0878761

CEP78	-4.083767151	0.006666086	0.026752753
MPC1	-4.083952058	0.032036138	0.088009522
RNF20	-4.084266688	0.033564828	0.091091632
POLN	-4.084417134	0.034465264	0.092827672
PRXL2C	-4.084726949	0.04614646	0.115237621
PDGFA	-4.085562193	0.00992208	0.036359931
B9D1	-4.08576874	0.037750628	0.099074934
ZNF615	-4.086519662	0.008263819	0.0315836
IFI6	-4.087300545	0.005574144	0.023353908
ANKRD20A11P	-4.088160552	0.01728282	0.055268495
HSD11B1L	-4.089538785	0.050858441	0.12403598
PLA2G7	-4.089551998	0.039963797	0.1033917
CCDC32	-4.089662791	0.007056943	0.027941525
FCF1P7	-4.090795713	0.005283911	0.022488328
METTL2A	-4.090816653	0.034415238	0.092734204
UTP15	-4.091089646	0.014021705	0.047249085
ENSG00000214135	-4.091270015	0.013172692	0.045239612
IMMP1LP1	-4.09207711	0.02193019	0.066238423
DZIP3	-4.092127218	0.005283645	0.022488328
CHTOP	-4.092667205	0.000412678	0.003096746
DYNLT1	-4.093605081	0.014694173	0.048996215
CCDC102B	-4.093726641	0.015943312	0.052098929
SLC9C1	-4.094859606	0.016830188	0.054265474
FAM126A	-4.096461499	0.022751476	0.068162488
DDX55	-4.097345411	0.000564686	0.003962292
SHC1	-4.10058894	0.000142155	0.001397759
GTF2H2C	-4.101329231	0.01179089	0.041463373
PWWP2B	-4.102740116	0.050128477	0.12265112
NR6A1	-4.10377898	0.008574965	0.032424671
CYRIB	-4.104099119	0.000744664	0.00491935
OR2A9P	-4.104162378	0.001724633	0.009331675
OXSM	-4.104456668	0.046416842	0.115674174
PIGH	-4.10553918	0.002281118	0.011620644
BBS5	-4.105949464	0.00054864	0.003870865
TUBD1	-4.106500226	0.001032073	0.006315578
NAA38	-4.107406325	0.033775636	0.091458681
PSMD10	-4.107437077	0.029333337	0.082685307
ZNF702P	-4.107534225	0.03597743	0.095707872
BRPF1	-4.108511817	0.039079723	0.101681204
TOMM7	-4.108666827	0.013247072	0.045345746
FOXRED2	-4.110288251	0.03200748	0.088009522
NDFIP1	-4.110990818	0.005522812	0.02320344
SLC9A5	-4.111674405	0.004496384	0.019776246
ATP5MC1	-4.112974207	0.007343421	0.028821261

CCNJL	-4.115581661	0.01410332	0.04747124
ZFPM1	-4.116609105	0.041052265	0.1054087
MARCHF9	-4.11735997	0.037280577	0.098140998
IFI35	-4.11739977	0.023613331	0.069984025
FAM47E-STBD1	-4.117728393	0.022922853	0.068512586
TCEA2	-4.118032392	0.001561039	0.008689603
NUTM2D	-4.119194284	0.018684364	0.05866551
LCMT1	-4.121078156	0.018166047	0.057501119
ST6GALNAC6	-4.1216699	0.00411164	0.018377876
ANAPC11	-4.122848081	0.013605667	0.046155548
MYG1	-4.123703996	0.037087391	0.097822135
ASB8	-4.124873319	0.002396006	0.012098982
SYT17	-4.125939427	0.004227325	0.01884624
MZB1	-4.126642384	0.036496115	0.096662797
ZNF197	-4.127425059	0.011123467	0.039634513
GUCY1A2	-4.127765382	0.013807808	0.04665821
POLR2K	-4.12983404	0.035944733	0.09566294
NOC3L	-4.130385639	7.96E-05	0.00090428
RPUSD1	-4.131253309	0.017672358	0.056305927
COG3	-4.13136745	0.00036145	0.002801482
XRCC4	-4.132219266	0.0362743	0.096272521
SLC1A3	-4.133152503	0.030320755	0.084836436
RIOK1	-4.134325177	0.00847364	0.03217219
BSPRY	-4.13449807	0.03502284	0.094027024
ENSG00000253330	-4.135564467	0.018329758	0.057897544
DR1	-4.136254047	0.004107156	0.018364615
CYP20A1	-4.136771366	0.013605279	0.046155548
NCF1	-4.13781138	0.01862192	0.05856699
ITGB1BP1	-4.138034183	0.000754477	0.004960448
ZNF620	-4.138237661	0.010954406	0.039147365
TTI2	-4.138447104	0.013862714	0.046817603
SHF	-4.138707309	0.004013272	0.018031417
PWWP2A	-4.139199551	0.003061454	0.014640789
CSF3R	-4.141792445	0.004747331	0.020654813
MRPS31	-4.142686397	0.006653886	0.026730379
DUSP8	-4.143468065	0.013183923	0.04525743
RTEL1P1	-4.143678049	0.030471059	0.085150913
MAPK3	-4.143878561	0.001704831	0.009249341
FBXW8	-4.145339907	0.028793232	0.081599925
VEZF1	-4.145379736	0.024541256	0.072221581
CHRNB1	-4.145845286	4.31E-05	0.000554157
TTC31	-4.146143436	0.000249278	0.002139905
ENSG00000229689	-4.146891177	0.017670102	0.056305927
PI4K2A	-4.14856797	0.030911163	0.085932181

NOL4L	-4.149015611	0.000497405	0.003575172
RABAC1	-4.149565864	0.004315123	0.019188189
MLST8	-4.150315289	0.000703521	0.004704649
ERI3	-4.152677159	0.005883525	0.024296892
PIH1D2	-4.155439156	0.028842873	0.081677985
C1QA	-4.155801112	0.028117747	0.080192555
NKIRAS1	-4.156497095	0.032530655	0.089016565
BAG2	-4.157840526	0.007962854	0.030695027
LIN9	-4.157872589	0.017267253	0.055247907
MATR3	-4.158174921	0.033077905	0.090113653
RHOD	-4.15987163	0.034925431	0.093837384
ENSG00000268279	-4.160311844	0.00611906	0.025009078
SRPX	-4.161231855	0.036955598	0.097559556
CPNE3	-4.161602228	0.00179579	0.009622063
ORC4	-4.162306689	0.000132328	0.001322623
ATP6V0D1	-4.162758397	0.000376807	0.00289029
FBXO4	-4.162832506	0.018635276	0.05856699
TRIM4	-4.163316626	0.006345355	0.025737182
MAPRE3	-4.165010476	0.024732008	0.072580512
TMEM39A	-4.166058219	0.00186445	0.009945924
MSH5	-4.166684932	0.025867975	0.075139423
GMCL1	-4.168268325	0.007596178	0.029583055
HMBS	-4.1688226	0.006677219	0.026788549
SLC25A1	-4.169845466	0.002252263	0.011508055
GATB	-4.172068333	0.000994704	0.006136545
TTC33	-4.172930395	0.017865378	0.056741677
GNRHR	-4.173738313	0.048260892	0.119141309
ABCD3	-4.174331317	0.009517325	0.035207114
SPIRE1	-4.175005537	0.004102711	0.018358303
ENSG00000228897	-4.175014714	0.017583508	0.056126262
TMEM141	-4.176185076	0.002125262	0.010997934
ENSG00000235859	-4.178754437	0.003534803	0.01634897
CSF2RB	-4.180071242	0.022539273	0.067712922
PLA2G12A	-4.180222167	0.01413602	0.047554859
ZNF876P	-4.180979973	0.009707956	0.035781089
GALNTL6	-4.181970085	0.049757981	0.121867786
TCEAL4	-4.182609814	0.003460815	0.016080503
SMAD7	-4.183524261	0.01816242	0.057501119
ZNF304	-4.184545339	0.01172326	0.041298143
AKAP10	-4.187294888	0.000248306	0.002134584
MTHFD1L	-4.189146537	0.001726568	0.00933797
STXBP5	-4.191532371	0.001863547	0.009945489
TMEM86B	-4.193474108	0.028997648	0.082006431
LANCL3	-4.193717467	0.012496153	0.043363942

PCDHGB8P	-4.193872172	0.006488394	0.026176303
OMA1	-4.197523801	0.005522894	0.02320344
RBBP4P2	-4.199831176	0.02600678	0.075506372
C1orf116	-4.200839716	0.012769792	0.044086015
EGFEM1P	-4.20134513	0.036278139	0.096272521
FAM124A	-4.201863083	0.050689464	0.123748542
SAXO2	-4.202749408	0.005693687	0.023736624
EMC9	-4.203197671	0.03517266	0.094368073
HGH1	-4.203339737	0.019852622	0.061472535
C17orf75	-4.204547038	1.37E-05	0.000219865
DNAJC17	-4.204649789	0.016469995	0.053326271
MIF	-4.205140167	0.000355012	0.002765167
IDI1	-4.205382845	0.017022763	0.054624475
EEF1AKMT2	-4.206326939	0.008714004	0.03278654
ENSG00000170089	-4.20640838	0.012324364	0.042927798
MED20	-4.206966499	0.009881453	0.036287957
TAF1A	-4.207416642	0.050709598	0.123770884
TOP3A	-4.207493136	0.00122209	0.007229805
AAMDC	-4.208614395	0.050114401	0.122641471
IRX3	-4.208829666	0.047709924	0.118190936
INHBA	-4.211896825	0.02477103	0.072646289
WNT5A	-4.211971274	0.004628155	0.020208941
LRRC14	-4.212855984	0.011542384	0.040779049
FNTB	-4.214628003	0.02260629	0.067813269
DHX32	-4.21505802	0.030781838	0.085703245
RABL2A	-4.215251477	0.004613526	0.020161938
HMGB3P24	-4.21536779	0.035757982	0.095396652
RBAK	-4.21562941	0.01349845	0.04596102
GVQW3	-4.217068652	0.005390663	0.022822172
GK	-4.21799975	0.031016089	0.086106528
ZNF37BP	-4.218789008	0.001045395	0.006374538
ADAM8	-4.219778124	0.047364869	0.11755267
BORCS5	-4.220013801	0.034245731	0.0923597
AFAP1	-4.220185154	0.027132605	0.078044927
CYREN	-4.220350173	0.002222193	0.011404712
BIRC2	-4.221293552	0.00012445	0.00125948
VPS4A	-4.221410832	0.003318209	0.015561255
POLE4	-4.221937061	0.011384382	0.040391163
BMT2	-4.222044233	0.030830539	0.085786862
AP2B1	-4.222279044	0.001120919	0.006736644
PRPF4B	-4.222792432	0.00270608	0.013260889
FAM228B	-4.225748439	0.000993006	0.006132321
EIF3FP3	-4.226728443	0.031997471	0.088002455
EID3	-4.227838104	0.025224437	0.073607194

CACNB4	-4.228431461	0.025033335	0.07326003
ALDH4A1	-4.229345095	0.006338364	0.025727553
TDP2	-4.230221237	0.000895815	0.00566803
TPTE2P5	-4.232863381	0.014208888	0.04773366
MCTS1	-4.23318502	0.004639183	0.020242492
ZDHHC3	-4.234179281	0.023753186	0.070261136
OTULIN	-4.234339971	0.005829652	0.024107315
FAM104A	-4.234399161	0.008402951	0.031956252
CBFA2T3	-4.235727391	0.010165594	0.036983573
BORCS7	-4.236035779	0.026775169	0.077217139
OSBP	-4.238455432	0.008367175	0.031877961
THG1L	-4.238813717	0.027809716	0.079576548
TRAF3IP1	-4.239567496	0.005629954	0.023522597
TDRKH	-4.239727154	0.002966184	0.014292472
ENSG00000260914	-4.239743605	4.70E-05	0.000596518
CCDC77	-4.241728236	0.033225641	0.09041438
EXTL2	-4.244738398	0.013028905	0.044852635
SUMO3	-4.245297442	0.000743577	0.004915489
SEPTIN7P14	-4.247441794	0.028335821	0.080710551
TPTEP1	-4.248073671	0.011692073	0.041223669
SLC25A40	-4.249359779	0.043774571	0.110615326
MAK16	-4.249861995	0.050479452	0.123335342
RANBP17	-4.250025088	0.004002329	0.018002301
TXN	-4.250594869	0.050722339	0.123770884
TNFRSF10C	-4.251813153	0.030289582	0.084774884
TNIP2	-4.254491959	0.007364523	0.02887599
PRRG2	-4.255072971	0.029121249	0.082221505
CASP2	-4.255835624	0.012082907	0.042234888
ASCC2	-4.256184514	0.002006032	0.010506709
ACP6	-4.25816625	0.00406375	0.018231145
AQP1	-4.259931492	0.014231918	0.04775801
NHLRC3	-4.260583701	0.000142416	0.00139919
IRGQ	-4.261756872	0.043240428	0.109741628
CFH	-4.263199008	0.006010189	0.024714715
PTMS	-4.263591254	0.049417543	0.121210831
C8orf76	-4.264412505	0.013191934	0.04525743
CD34	-4.264574881	0.000156719	0.001498363
BARD1	-4.265441431	0.00892836	0.033499338
RPS3AP26	-4.266164464	0.005691619	0.023736624
TEAD2	-4.267857949	0.008638552	0.032585808
SHLD2P3	-4.268088107	0.043122818	0.10947118
CPNE1	-4.268481822	7.97E-05	0.000904538
RAB4B	-4.269349553	0.000602241	0.004172593
PPP2R5B	-4.269460041	0.035379303	0.094762355

PCDH7	-4.269612077	0.00033992	0.002687386
ITPRIPL2	-4.271011362	0.000727891	0.004832906
RRN3	-4.271362262	0.00387001	0.017545994
NPM2	-4.272192336	0.049566342	0.12149676
SIVA1	-4.275041087	0.002239366	0.011475209
COQ8A	-4.275108174	0.005696597	0.023736624
IL18BP	-4.275453539	0.001994625	0.010478705
MRPL35	-4.275548995	0.04776819	0.11828686
UTP18	-4.275582948	0.012424637	0.043165272
RABGGTA	-4.275742001	0.000329722	0.002637776
KIAA0825	-4.275745399	0.00014736	0.00143611
PHF14	-4.276217945	0.00287935	0.013935087
FBXO44	-4.277328859	0.000701146	0.004691363
POLR3F	-4.27829794	0.01077051	0.038581312
MTFMT	-4.278501613	0.00587141	0.024255136
PDPK2P	-4.280230631	0.000678998	0.004583708
FAM104B	-4.28033369	0.024797435	0.072675098
CDH24	-4.28063245	0.005801687	0.024024502
PSD4	-4.282144731	2.33E-05	0.000343237
DPY30	-4.282791077	0.04000522	0.103448743
LPCAT3	-4.283071848	0.004531897	0.019874667
MRPS28	-4.285446239	0.007319062	0.028753627
LYRM1	-4.285506457	0.008230511	0.031486127
SZRD1	-4.285978321	0.016012326	0.052255184
NOB1	-4.286173509	0.016800939	0.054185601
PLEKHG6	-4.287651694	0.000975599	0.00606505
GAPDHP2	-4.287981747	0.031599391	0.087244303
ARL4AP4	-4.288059097	0.005232087	0.022306862
ZRANB3	-4.289812151	0.03290029	0.089838693
SENP5	-4.290410192	0.000174226	0.001619688
KCNK1	-4.290455642	0.024264362	0.071545879
AGAP6	-4.292015698	0.026217379	0.07600842
CCDC158	-4.292132494	0.016448745	0.053291115
ENSG00000229273	-4.292288677	0.019906325	0.061580151
MAP3K21	-4.292302786	0.013193679	0.04525743
AK4	-4.29237355	0.026248303	0.076047984
SIK2	-4.292861254	0.020075174	0.061904199
PARP16	-4.293041873	0.027353575	0.078549863
POLR3C	-4.293467361	0.017749544	0.056462676
CADPS2	-4.293603195	0.003135653	0.014907286
GJC1	-4.294832957	0.007265172	0.028597607
KLHL3	-4.295229465	0.003475941	0.016138395
PCDHGB6	-4.295690308	0.03996776	0.1033917
DDX52	-4.296195868	0.000988666	0.006111755

WDR35	-4.296393334	0.001308866	0.007597643
TANGO2	-4.296493482	0.000201047	0.001810623
ARF4	-4.300074587	0.005546384	0.023269821
MCPH1	-4.300430084	0.018878967	0.059153771
WWTR1	-4.301490138	0.000152126	0.001471893
SARDH	-4.301992636	0.01422786	0.04775801
CLDN7	-4.302716961	0.001449631	0.008218422
MRPL17	-4.305104869	0.00962411	0.03550449
YIPF6	-4.305324379	1.32E-05	0.000213573
MBP	-4.305801379	0.006915256	0.027533636
NIPSNAP1	-4.306848928	0.04256074	0.108362474
DHX8	-4.309522266	0.000709935	0.004737078
ENSG00000284776	-4.309696363	0.028745347	0.081502385
ZNF275	-4.309819349	0.031899831	0.087813407
RBM41	-4.309850871	0.004791802	0.020795976
HAPLN3	-4.31169173	0.027581211	0.079147222
PIGS	-4.311839447	0.00032793	0.00262865
FBXO6	-4.312195073	0.046479953	0.115794559
DGKI	-4.313119113	0.002070013	0.01074859
RPL36AP13	-4.31555659	0.028971025	0.081969445
PHYH	-4.315874302	0.016916397	0.054435908
CYTIP	-4.315947936	0.041103956	0.105519043
SHANK2	-4.316276146	0.011062739	0.039464603
RPL7P46	-4.318132561	0.048653214	0.119865357
NPM1P26	-4.319628861	0.002329951	0.011829586
ZNF26	-4.320197521	0.001119415	0.006734296
COQ10B	-4.320682596	0.021078047	0.064296544
TBL2	-4.320752409	0.001965502	0.010379769
EMB	-4.320793626	6.97E-05	0.000815037
KHDRBS1	-4.320835622	0.002420913	0.012168906
ABHD17B	-4.321086673	0.021310366	0.064828105
ENSG00000223804	-4.321526709	0.011532732	0.040759346
FABP5P9	-4.321639881	0.041842364	0.107028734
GPX2	-4.321909172	0.032076412	0.088099136
L3MBTL4	-4.323117752	0.007295792	0.028680828
CHADL	-4.32354378	0.027692681	0.079373008
ZSWIM7	-4.326032976	0.013498961	0.04596102
ZNF623	-4.326265458	0.001352795	0.007793539
CDHR3	-4.327631843	0.04484465	0.112707568
KDR	-4.328482096	0.002408031	0.012129342
HELLS	-4.329448688	0.000566338	0.003971433
SAYSD1	-4.329474221	0.006396714	0.02590359
PLAA	-4.329569385	0.001366723	0.007855087
IFT46	-4.331721722	0.007728904	0.029964977

CCSER1	-4.33180827	0.00539982	0.022852945
LACTB2	-4.331888002	0.020908333	0.063923835
FLT3LG	-4.333977797	0.018339243	0.057912391
EDRF1	-4.335228061	0.00256959	0.012757306
ARMC1	-4.338272275	0.019839243	0.061462553
RILP	-4.338586558	0.044264092	0.111572798
TEKT4P2	-4.339699071	0.042302311	0.107863318
PDE2A	-4.339890796	0.0078467	0.030345374
DXO	-4.340438053	0.01755761	0.056058379
PCYOX1L	-4.340510986	0.011236812	0.039932583
ZSCAN30	-4.343213198	9.73E-05	0.001040309
MRPL28	-4.345119043	0.011847576	0.04163258
CXCR6	-4.345859919	0.005698679	0.023736624
AK1	-4.345876411	0.002573522	0.012771592
SLC27A2	-4.346404876	0.044648163	0.112307016
PSKH1	-4.346469607	0.004758513	0.020685881
ENSG00000165121	-4.347041645	0.012931802	0.044581752
AKAP8	-4.348543706	0.023398128	0.06961921
HAVCR2	-4.348656275	0.040390669	0.104179577
CTNNBIP1	-4.35003911	0.019494094	0.060616785
DGCR2	-4.352122901	0.000793668	0.005167592
MTHFD2L	-4.35275705	0.045985066	0.114937642
ZNF226	-4.352810705	0.043473285	0.110152949
CDYL2	-4.353164335	0.040536103	0.10440104
ZNF420	-4.354078456	0.023305548	0.06939492
ESRP2	-4.354112307	0.000300661	0.002458918
POLR2J4	-4.354357168	0.034396367	0.092724637
TRPT1	-4.355165216	0.014071751	0.04739134
CASP3	-4.356307131	0.010351457	0.037468314
MXD1	-4.356898761	0.00609142	0.024942676
MAD2L1BP	-4.358169544	0.047691701	0.118169979
KAT8	-4.359348645	0.002118561	0.01096795
SPINT1	-4.359387966	0.000115496	0.001185718
C6orf47	-4.362309033	0.002734433	0.013378164
CUEDC1	-4.362420093	0.005227504	0.022295175
THAP7	-4.362829037	0.020270174	0.062382454
DDX18P3	-4.362937292	0.002614983	0.012919084
PLEKHM2	-4.363373981	0.000978545	0.006077119
DLSTP1	-4.363756023	0.00594405	0.024502918
CHRM3	-4.364172559	0.010995873	0.03926078
ANOS1	-4.365418	0.041128642	0.105560025
ZNF486	-4.365623795	0.012580094	0.043571169
ATP6AP2	-4.365987672	0.003614525	0.016616105
KIF5A	-4.367182987	0.007868618	0.030399538

TRAPPC14	-4.367542559	0.000697349	0.004673703
RBL1	-4.370978983	0.001146141	0.006867765
ATL1	-4.371254566	0.021795764	0.065937497
MKRN2	-4.372071608	0.009970496	0.036477192
PLAT	-4.374167549	0.000993806	0.006134128
NRSN2	-4.374441863	0.035625878	0.095168465
SFXN2	-4.374447483	0.02520533	0.07358852
L3HYPDH	-4.375084595	0.005698829	0.023736624
BLOC1S5	-4.377501575	0.022639579	0.06787948
ZNF707	-4.378594271	0.004913126	0.021261522
IL33	-4.380333667	0.037986533	0.099607669
CLPX	-4.380397065	0.000130984	0.00131297
ENOX2	-4.380896157	0.037421891	0.098446983
OCRL	-4.381100153	6.96E-05	0.000814723
SHISA6	-4.381320764	0.029757295	0.083588373
GABBR1	-4.381562492	0.016154875	0.052564141
ZNF653	-4.383363223	0.02512371	0.073488976
ZNF665	-4.384075084	0.021261027	0.064740497
HLCS	-4.38516364	0.000264004	0.002239316
ZNF234	-4.38532921	0.018504314	0.058327141
COPG2	-4.385360455	0.000878581	0.005579402
NUBP1	-4.385647511	0.003545775	0.016387196
MMS22L	-4.385768828	2.86E-06	5.70E-05
PPIAP53	-4.386241762	0.01328572	0.045435442
C3orf35	-4.386879102	0.022868407	0.068405288
PIGU	-4.388023446	0.014721182	0.049046294
NDUFA2	-4.389049518	0.004987336	0.021481465
SEC14L1P1	-4.389825917	0.021726661	0.06578244
SNRPD1	-4.390947764	0.003450733	0.016045974
ENSG00000228007	-4.391221473	0.019413069	0.060451708
SMG1P6	-4.39159508	0.005373573	0.022773715
PFKFB3	-4.392721479	0.00192062	0.010182735
FAM174C	-4.392723034	0.042092217	0.107486117
THEM4	-4.393394293	0.005520717	0.02320344
SPI1	-4.393427507	0.028589884	0.081175689
ABCC4	-4.393485268	0.013800977	0.046648151
TMC8	-4.39666161	0.001959941	0.010354923
CCDC9B	-4.397071792	0.01461808	0.048796812
NDUFA11	-4.397110681	0.015847062	0.051899846
SCARA3	-4.397203532	0.000648506	0.004419968
RICTOR	-4.39803733	0.001028434	0.006302868
ZKSCAN3	-4.398262179	0.006347116	0.025737182
CCT5	-4.398693508	0.003757536	0.017111067
ZNF239	-4.398971737	0.037443744	0.098483067

EIF1AD	-4.399125745	0.019264481	0.060081752
RWDD4	-4.39923074	0.002738932	0.013389559
CDH12P2	-4.401361299	0.025143977	0.07352737
SLC19A2	-4.403616122	0.028456198	0.080928691
COMMD2	-4.403706623	0.040392825	0.104179577
KIF18A	-4.404079908	0.018637523	0.05856699
LINC01347	-4.405611296	0.000846673	0.0054137
UBR1	-4.407252879	0.000475902	0.003458587
VPS37B	-4.408016917	0.001170524	0.006989652
COX7A2L	-4.408054485	0.000450603	0.003311422
LRRC37A2	-4.408457355	0.039225262	0.101994108
GOSR2	-4.408480712	0.002200981	0.011326818
MIEN1	-4.410232274	0.010563784	0.038077438
SNHG32	-4.410713775	0.011020018	0.039323792
MAGOH	-4.411156589	0.039805941	0.103127378
HLA-C	-4.411767251	0.00271129	0.01327567
TCEA1P2	-4.411956716	0.000889979	0.005636997
AGO4	-4.412245171	0.040817281	0.104849823
NOP16	-4.414970523	0.024250001	0.071538388
NPM1P27	-4.415024675	0.002004588	0.010506709
TMX1	-4.41519042	0.000119056	0.001216386
RPL13AP5	-4.417549581	0.03699259	0.097614629
EMC3-AS1	-4.418265895	0.011132974	0.039646746
SEC61G	-4.418753583	0.017734905	0.056430939
PIK3CA	-4.420578271	0.042288426	0.107850634
HLA-DMB	-4.420995077	0.005706474	0.023743574
KDM4B	-4.421886878	3.91E-05	0.000513309
PILRA	-4.421917317	0.026049718	0.075612898
TMEM241	-4.422247078	0.01595024	0.052098929
CDK11B	-4.422447114	0.000236773	0.002061802
EEF1AKMT3	-4.422463263	0.01236751	0.043003831
KCNIP3	-4.422682335	0.043916706	0.110905031
GNL3	-4.423352436	0.016460531	0.053315029
CUL5	-4.423724324	0.000661271	0.00449412
CHMP6	-4.424011729	0.016525691	0.053454559
MUS81	-4.424438612	0.002679332	0.013145779
AZIN2	-4.425645378	0.020448673	0.062819983
ENSG00000283427	-4.42586519	0.038566926	0.100780241
HSPD1P11	-4.426013008	0.007017896	0.027859829
MYNN	-4.426236998	0.000800668	0.005196399
FAP	-4.427031335	0.040728394	0.104732628
SEPSECS	-4.428140158	0.035839995	0.095550785
EEF1B2P3	-4.428657633	0.048562018	0.119689406
ALG14	-4.428699498	0.038291857	0.100217603

NAA40	-4.429486699	0.000903383	0.005701013
HLA-DMB	-4.42974302	0.013511319	0.045966059
C12orf29	-4.429932684	0.050331225	0.123047696
CIB1	-4.430173608	0.00558998	0.023395959
CD37	-4.430691774	0.036400562	0.096451928
MARCHF8	-4.433299648	0.015243662	0.050442086
WDR25	-4.434233226	0.007613338	0.029621295
TMEM102	-4.434292221	0.036562154	0.096774178
BCL3	-4.43493367	0.002748258	0.013424096
HMGN5	-4.435281041	0.045923606	0.114870702
RTN4R	-4.435579405	0.01605549	0.052360437
CATIP	-4.435623707	0.022581001	0.0677542
TMEM79	-4.436899478	0.03161614	0.087268161
LRRC1	-4.437644725	0.021973259	0.066292204
ARV1	-4.437875286	0.035847333	0.095550785
SLC25A25	-4.438645779	0.00324782	0.015308259
TSPAN31	-4.439392232	0.00290774	0.014044409
TYRO3	-4.439910202	0.00380728	0.017285564
SFXN1	-4.44177391	0.040769871	0.104783611
HDGFL3	-4.443243602	3.54E-05	0.000478788
DHX35	-4.444016883	0.002054648	0.010696542
RAB8A	-4.444460552	0.010265405	0.037257112
CNOT6L	-4.445618207	0.035923053	0.09563266
CHST4	-4.445629517	0.018307228	0.057870573
DSN1	-4.446112708	0.012338322	0.042951696
FCHO1	-4.448280224	0.046402547	0.115663861
C1orf74	-4.448670049	0.036829226	0.097289601
AK6	-4.449102493	0.003871557	0.017545994
ZBTB45P1	-4.449724667	0.01005688	0.036764868
ZNF785	-4.449885999	0.001677315	0.00915339
ZNF331	-4.450162003	0.000877121	0.005575981
TUBGCP4	-4.450590026	0.000331762	0.002652339
TBL1X	-4.450687997	0.004483886	0.019749984
DPEP2	-4.450743249	0.006906357	0.027507252
AGBL2	-4.451005051	0.03993133	0.103365024
DPCD	-4.451714798	0.015206974	0.050359839
GSTK1	-4.452380475	0.000211425	0.001885844
DHRS11	-4.452401788	0.001246365	0.007333985
DYRK4	-4.453211675	0.006358768	0.025775798
CSMD1	-4.454061066	0.038839192	0.101273068
CNTNAP3	-4.455170644	0.002671221	0.013116614
PTCD2	-4.455880585	0.019116504	0.059681755
NOMO1	-4.456409635	0.00198362	0.010443554
DCST2	-4.456620007	0.042114087	0.107519279

ZMAT1	-4.458623389	3.68E-05	0.000489541
USP35	-4.459804099	0.000265074	0.002244302
RNF166	-4.460122037	0.007186455	0.028370792
ZNF35	-4.461972837	0.025688311	0.074654857
IFT27	-4.463666086	0.004293025	0.019103963
RPL3P6	-4.463752726	0.014047602	0.047323176
RNF135	-4.46409262	0.001501045	0.008437316
TRIM68	-4.465460087	0.007466712	0.029172718
CNTFR	-4.465865659	0.03787795	0.099365995
HSP90AB2P	-4.466704689	0.010677816	0.038326521
PTPN1	-4.467072592	0.021485555	0.065276394
PHACTR4	-4.467977737	4.70E-06	8.80E-05
PROSER2	-4.469906025	0.011202412	0.039822023
SALL4	-4.470183847	0.02998995	0.084144264
PXDC1	-4.472987828	0.037493274	0.098591917
UBALD2	-4.473027691	0.006131733	0.025039979
GRK4	-4.473209314	0.0019854	0.010448384
LARS2	-4.473303311	0.001237201	0.007294241
PPWD1	-4.473311734	3.74E-05	0.00049685
UBE3D	-4.474705603	0.008639116	0.032585808
TULP3	-4.475089277	0.00266897	0.013115526
LEPROT	-4.476215963	0.001042731	0.00636471
GLT8D2	-4.476672859	0.039049985	0.101647532
CHRD	-4.476929306	0.005329736	0.02261168
GEN1	-4.478776742	0.016060749	0.052360437
AATF	-4.478937325	0.009910258	0.036349627
TRMT61B	-4.480085815	0.019955548	0.061681805
COG6	-4.482083734	0.000656571	0.004468577
PLCB2	-4.483210941	0.003723497	0.017026525
RPS29	-4.483794034	0.00161087	0.008874816
CENPP	-4.483818715	0.030018621	0.084205189
OTUD4P1	-4.484514096	0.001208571	0.007163833
ZSCAN23	-4.485166191	0.00941421	0.03491103
TMEM8B	-4.48551289	0.004373305	0.01938992
CCDC110	-4.486082739	0.036767054	0.097188998
KIAA0753	-4.48697745	0.000803094	0.005206564
BBS7	-4.487024065	0.004743322	0.020644794
CTIF	-4.488309221	0.000195718	0.001770535
TMX3	-4.48863177	0.000177478	0.001639845
MED22	-4.490108083	0.009434542	0.034943605
STPG3	-4.490289394	0.024189516	0.071387839
DDX19A	-4.493248043	0.000147153	0.00143611
NPIPB7	-4.49434458	0.023460906	0.069714462
SEZ6L2	-4.494957867	0.006632282	0.026670147

CEACAM1	-4.495300534	0.036745817	0.097154078
APBA3	-4.495479034	0.024748867	0.072603074
BID	-4.496902586	0.019003884	0.059435179
HMBOX1	-4.498690447	0.000818715	0.005274307
LDHAP4	-4.500231447	0.03616604	0.096061828
LYPLAL1	-4.500946247	0.000416839	0.003121732
ZNF565	-4.501619927	0.005304915	0.022537976
TAS2R19	-4.502199828	0.043897294	0.110879142
JAG2	-4.502236734	0.006196561	0.025253596
SLC25A15	-4.502707936	0.021817241	0.065969493
ENSG00000237115	-4.503390073	0.000868711	0.005534148
ZFP37	-4.506409321	0.005719766	0.023764564
TP53I3	-4.507745491	8.02E-05	0.000908315
RWDD3	-4.508130836	0.009080113	0.033911041
SLC9A1	-4.509396771	0.001539777	0.00860455
FBXO32	-4.510258884	0.001770484	0.009528651
EXOSC4	-4.510309893	0.009548842	0.035291354
HNRNPA1P45	-4.5121705	0.040539069	0.10440104
ENSG00000259079	-4.513226466	0.043067414	0.109353467
ANGPTL4	-4.513429239	0.003850063	0.017466701
SYF2	-4.513564387	0.048023768	0.118691889
IGHV4-4	-4.514304694	0.042037055	0.107390569
C9orf64	-4.51501456	0.018442101	0.05817649
CFAP92	-4.516218699	0.002401444	0.012115876
MXD4	-4.51630601	0.006018879	0.024715577
C18orf25	-4.516517677	0.00187303	0.009982895
POM121L14P	-4.518174701	0.028257125	0.08051418
SLC7A1	-4.518719022	0.032460669	0.088872185
ADPRH	-4.518943154	0.017188075	0.055038217
LRP2BP	-4.519241344	0.000621418	0.004283764
FAAP20	-4.520155166	0.000784213	0.005128099
ZNF660	-4.520200615	1.43E-05	0.000227833
RPS9	-4.520235217	0.006757402	0.027047485
MSH5	-4.521244432	0.038886888	0.101333477
MCRIP2	-4.521268186	0.004380203	0.019413393
VPS52	-4.521960847	0.011973339	0.041970836
MCM4	-4.522378542	0.000903383	0.005701013
VPS26C	-4.523438477	0.012696428	0.043920424
TADA2A	-4.523487223	0.033180185	0.090331299
FXYD6	-4.524951093	0.00868801	0.032714817
COL6A1	-4.525399096	0.039468434	0.102460116
KLHL8	-4.527045206	0.000434562	0.003219054
FAM227B	-4.527224878	0.000715733	0.004765256
SPAG16	-4.528421629	0.008647379	0.032606813

PCED1B	-4.529073551	0.010636985	0.038238807
STARD4	-4.52961989	0.041577767	0.106464415
USPL1	-4.531287585	0.009011059	0.033736425
ZNF699	-4.531332617	0.001371413	0.007878302
KCNQ5	-4.531790007	0.003018164	0.014485272
PNKD	-4.531837479	0.001625736	0.008916133
DUS4L	-4.532058313	0.019069145	0.059595385
L2HGDH	-4.532095311	0.007357686	0.028861093
DPY19L1P1	-4.53239801	0.017638351	0.056256813
RNASE1	-4.533466183	0.025545422	0.074365481
ARID5A	-4.533816964	0.000428364	0.003190717
SWI5	-4.53568243	0.024707804	0.072570554
GCSHP3	-4.538178483	0.016279512	0.052894282
FASTKD3	-4.540382262	0.011163596	0.039725707
RABEPK	-4.540865186	0.009255162	0.034426698
SKAP2	-4.543269861	0.034111593	0.092151948
ABCB9	-4.544748693	0.002647815	0.013038713
NAP1L4	-4.545613147	0.001762342	0.009497501
ZCCHC10	-4.549562412	0.042986856	0.109194733
ENSG00000243403	-4.549706434	0.037393555	0.098393823
PPP1R2	-4.551105132	0.000873665	0.005559855
CKS1B	-4.551319045	0.032036509	0.088009522
FCRL5	-4.552863755	0.004816293	0.020894769
EED	-4.552931052	0.006015367	0.02471487
COX18	-4.553258634	0.003522459	0.016316817
R3HCC1L	-4.553592997	0.00281768	0.013718904
ZNF439	-4.554654634	0.000292605	0.002412987
SPDYA	-4.555341675	0.01170254	0.041248557
TRIM27	-4.555595954	0.020098936	0.061940999
EVC	-4.555735622	0.000760877	0.004991684
RPL17P17	-4.556744822	0.020845526	0.063780143
ENSG00000215158	-4.557472235	0.000292118	0.002411867
TMEM255B	-4.557978304	0.036655868	0.096937433
TMEM147	-4.559353376	0.008908186	0.033454757
INO80B-WBP1	-4.559476253	0.017358561	0.055496045
TTC39A	-4.560257805	0.009145162	0.034122393
EIF1AX	-4.560686564	0.009153888	0.03413434
NAA50	-4.56070734	0.003024385	0.014503628
PKIA	-4.561108851	0.031997588	0.088002455
CATSPER2P1	-4.561186391	0.022252755	0.067035178
NR1H2	-4.561895599	0.00018662	0.001709954
ZNF324B	-4.564039657	0.026256157	0.076047984
MED31	-4.564708676	0.032956212	0.089901608
MINPP1	-4.565729566	0.007483957	0.029221231

JAZF1	-4.566107511	0.014231677	0.04775801
TP53RK	-4.56632256	0.02624981	0.076047984
ABHD10	-4.567549856	0.007031689	0.027896283
SPHK1	-4.567851178	0.007946696	0.030642499
FAXDC2	-4.568056353	0.000612402	0.004233304
ODAD4	-4.568548992	0.012663986	0.043833253
ABHD12	-4.569311758	0.000392111	0.002971916
TRAFD1	-4.570251742	0.001974438	0.010417097
CARD19	-4.57084399	0.000293968	0.002417244
CCDC107	-4.571082439	0.03157078	0.087205095
MANSC1	-4.571501272	0.033445419	0.09088984
LRRC37A15P	-4.57160223	0.035925455	0.09563266
RCC1L	-4.572931442	0.00018341	0.001681817
GSTA4	-4.572958612	0.008120815	0.031145228
COQ5	-4.573158024	0.001197185	0.007113761
PLA2G4F	-4.574960031	0.02028244	0.062404334
SLC39A4	-4.574971957	0.00520732	0.022240436
GLOD4	-4.575600325	0.003639162	0.016710324
ABR	-4.576799079	0.002267057	0.011558744
NDRG4	-4.576963889	0.003297485	0.015478474
IFFO2	-4.577533701	0.010184471	0.037018869
ZFP28	-4.578596598	0.009021049	0.033742516
RABL6	-4.578854805	1.81E-05	0.000277786
SERTAD2	-4.57893393	0.001299331	0.00756111
ENSG00000263620	-4.579064964	0.008434227	0.032050033
C16orf87	-4.58061414	0.03853086	0.100707736
TRAPPC2L	-4.580969718	0.028510602	0.081026608
NAA35	-4.581338675	0.013684152	0.046356836
ANGPT2	-4.582570885	0.000163234	0.001542378
LIPH	-4.582682384	0.010414532	0.037629101
PGRMC2	-4.583287248	0.002491828	0.012468412
MAPK14	-4.583338193	0.000992573	0.006132321
ENSG00000277400	-4.586100865	0.043700721	0.11047484
UBE2W	-4.587227532	0.020059678	0.061876234
NUP160	-4.587250906	0.000336866	0.002675478
IMMP2L	-4.588285454	0.001105056	0.006654524
PLXNA1	-4.588988632	0.016835737	0.054268912
IFT22	-4.589630319	0.001702059	0.009249341
LILRB4	-4.589650904	0.038974736	0.101517153
ZNF691	-4.590023333	0.015498332	0.050976037
POLR3D	-4.590313023	0.002037658	0.010625287
RPL10P16	-4.590871875	0.002008669	0.010513099
B3GAT1	-4.590991931	0.000389622	0.002960446
ZFPL1	-4.594538613	0.019014316	0.05943938

TCP11L2	-4.594790109	0.003661814	0.016807962
CREBZF	-4.596537605	1.16E-05	0.000189988
C1orf131	-4.59657164	0.020670408	0.063388554
TTC27	-4.597766904	0.015918691	0.052061561
GRIK1	-4.598897346	0.018288879	0.057828786
SRM	-4.599365546	0.032426533	0.088839012
RPS3AP20	-4.599391303	0.008928301	0.033499338
TMEM200A	-4.599672153	0.02568882	0.074654857
ZNF57	-4.600359214	0.023261354	0.069297423
TIMM9	-4.600473065	0.007632737	0.02966816
SCAMP3	-4.600629086	0.000810856	0.005237249
ZXDC	-4.601312467	9.39E-05	0.00101587
TUBA1C	-4.603411849	0.000100326	0.001060674
PRDM5	-4.60590738	0.002311677	0.011761468
TBXAS1	-4.606028244	0.029058324	0.082090009
DDX39B	-4.608699025	0.000466354	0.003403809
STOX1	-4.608701637	0.032767104	0.089528899
ZNF251	-4.608930898	0.020793251	0.06370071
DHRSX	-4.611132396	0.002248668	0.01150375
AGGF1	-4.611807545	0.015951638	0.052098929
HERC2	-4.611828867	0.007287036	0.028655714
ADAMTSL2	-4.612489235	0.000136155	0.001350833
UMAD1	-4.612634622	0.015445705	0.050834182
YIPF5	-4.615325547	0.003602136	0.016584351
SMIM7	-4.616654765	0.005299155	0.022528397
COPZ2	-4.617586731	0.032939477	0.089878141
ATP8B4	-4.617959042	0.006072554	0.024890687
CXorf38	-4.618365096	0.000646981	0.00441684
MRPS16	-4.618534026	0.002643592	0.013033822
POLDIP2	-4.619761274	0.016575415	0.053572448
ELOB	-4.620398181	0.001908065	0.010138374
ZNF432	-4.621361194	0.006558928	0.026410267
HLA-DRA	-4.624457963	0.003457121	0.016069507
CHML	-4.625174227	0.038072892	0.099742137
EMP2	-4.625305087	0.021775744	0.065893401
IFT57	-4.626003085	0.000242778	0.002103497
RAVER2	-4.626149126	0.009230656	0.034389177
F11R	-4.627557586	2.75E-05	0.000393897
DCP1B	-4.627833202	0.012362088	0.043003831
LRRC28	-4.628213849	0.008080311	0.031019375
ENSG00000230953	-4.628419064	0.009906406	0.036346512
KIF3C	-4.628684781	0.001077757	0.006541063
RAB21	-4.629825693	0.002104798	0.010906026
ZNF283	-4.63010662	0.006750055	0.027035959

TMEM38B	-4.630545027	0.018026712	0.057209052
FSCN1P1	-4.631173606	0.001077847	0.006541063
NOL9	-4.631364378	0.030063729	0.084292653
SLC2A3P4	-4.631785142	0.029061111	0.082090009
CFAP221	-4.632157759	0.014436519	0.048265794
C14orf93	-4.634507755	0.001413603	0.008073283
ATOX1	-4.634627181	0.003361024	0.015731567
ATG16L1	-4.634675764	0.000933779	0.005850136
SOCS3	-4.635814201	0.000479566	0.003478316
RFXANK	-4.636072048	0.002061171	0.01072128
PSMB9	-4.636608472	0.016372338	0.053127941
ARHGEF25	-4.636896656	0.010164398	0.036983573
SLCO2A1	-4.636992515	0.002149951	0.011097233
DNALI1	-4.637238194	0.030979396	0.086062568
VKORC1	-4.638207144	0.004320506	0.019191415
UBE2D1	-4.639202434	0.007798292	0.030195306
NAF1	-4.639289302	0.002849197	0.013818275
RECQL4	-4.639644135	0.015194156	0.050331162
CFAP20	-4.640729445	0.00932676	0.034650431
COL21A1	-4.6413252	0.026607342	0.076844491
STARD3NL	-4.642410754	0.001092309	0.006600753
MFAP2	-4.642720008	0.023213435	0.069188728
JDP2	-4.642959573	0.02731187	0.078455263
ACOT9	-4.643425049	0.003428983	0.015987831
EXT2	-4.646157304	0.000742315	0.004911109
RHOBTB1	-4.647132494	0.027761193	0.079517559
KRT8P3	-4.647354445	0.026076108	0.075671352
AP4M1	-4.650258809	6.88E-05	0.000808191
PEX16	-4.650980248	0.001111549	0.006690299
BTN3A3	-4.651029475	0.004845917	0.021000707
TP53	-4.653314662	0.000512431	0.003659275
PLEKHO2	-4.653351546	0.02878662	0.081599925
DNAJC10	-4.653584485	0.00021023	0.001876568
FAM117A	-4.654436134	9.24E-05	0.001003427
SUGT1P3	-4.654496901	0.002822919	0.013727846
SHC2	-4.655087678	0.013086712	0.045013231
TXNRD1	-4.655567179	0.00038672	0.002945789
ALOX5	-4.655655189	0.033922491	0.091774213
PEA15	-4.656182746	9.77E-05	0.001043187
MVB12B	-4.658020474	0.038972664	0.101517153
GOLPH3L	-4.659632023	0.00374088	0.01706485
OSCP1	-4.660555215	0.012749546	0.044051076
FRMD4A	-4.662230669	0.0054143	0.022890217
MFSD2A	-4.66459423	0.000813369	0.005250677

PTPN7	-4.666172624	0.018511059	0.058333211
RNF216	-4.666653001	7.59E-05	0.000870441
POLR2F	-4.66721873	0.003589204	0.016531098
AFG3L1P	-4.667456246	0.00387189	0.017545994
AMN1	-4.668137773	0.003263139	0.015356545
ENSG00000255339	-4.668147069	0.008661219	0.032638665
PPP1R10	-4.668194936	0.00327529	0.015401751
ZNF610	-4.668323457	0.018156946	0.057501119
PANX1	-4.669233803	0.013616111	0.046178035
N6AMT1	-4.669345118	0.002582716	0.012811965
BDH1	-4.669365709	0.001590073	0.008796272
GLUD1	-4.669913453	0.03375887	0.0914542
PPAN-P2RY11	-4.670685903	0.03048943	0.085150913
ZNF789	-4.671252978	0.000194366	0.001759615
MTFR1	-4.671502666	0.002947386	0.014218876
TMEM243	-4.672181915	0.001569165	0.008724473
DHFR	-4.672318191	0.011402657	0.040403907
DDX27	-4.67246626	6.58E-06	0.000117833
BLCAP	-4.672783102	0.000698839	0.0046811
RCAN2	-4.67316961	0.036321125	0.096325787
NT5E	-4.674213715	0.001495854	0.008417673
ZCCHC17	-4.674383508	0.004343226	0.019277746
STEEP1	-4.675731063	9.98E-06	0.000167662
SMYD3	-4.676522793	0.001228033	0.007254323
GPD1L	-4.677269347	0.00260598	0.012900932
ENSG00000261884	-4.677687275	0.001629176	0.008924495
ZKSCAN8	-4.678913486	0.000336423	0.002675089
DCDC1	-4.679898198	0.013398024	0.04573313
TAS2R43	-4.680744581	0.031881524	0.087782976
RIPK3	-4.682202908	0.00047727	0.003465431
NDOR1	-4.684934563	0.001999392	0.010486583
SLC25A42	-4.685783798	0.008077731	0.031019306
YAF2	-4.686042927	8.47E-05	0.000942384
RABL3	-4.686439741	0.005725753	0.023775135
AIFM2	-4.68684949	0.026999165	0.077771989
ZMYM4	-4.687573736	0.000202314	0.001818666
GPATCH1	-4.68865492	0.003527504	0.016333934
FAM53A	-4.688997246	0.001166444	0.00697561
CNEP1R1	-4.689240199	0.032036513	0.088009522
CDKL1	-4.689394205	0.019979971	0.061709001
VAMP7	-4.689529121	0.015275995	0.05049171
NSMCE1	-4.690395823	0.02776982	0.079518785
UAP1L1	-4.690971808	0.010534754	0.037984112
FRMD8	-4.69178951	0.007262308	0.028597607

TIMP2	-4.692676937	3.56E-05	0.000479237
DDX51	-4.692944647	0.000910954	0.005739815
SCAMP4	-4.692992986	5.26E-05	0.000651884
ABR	-4.693607951	0.022091807	0.066600059
SPRTN	-4.693686538	0.035873243	0.095598795
WDR48	-4.694433234	4.36E-05	0.000559509
HSPA4	-4.697597187	0.000961768	0.005994675
BTF3L4	-4.69783548	0.002238618	0.011475209
GBP1P1	-4.698084671	0.018281307	0.057819947
HMGA1P2	-4.698651236	0.048874673	0.120191652
ODR4	-4.698811601	0.000292652	0.002412987
RAB4A	-4.699315829	0.014116202	0.047501391
BPNT2	-4.699739055	0.014567142	0.04865361
RCBTB2	-4.700027694	0.000996123	0.006142166
MRPL53	-4.701111805	0.008526526	0.032291948
PARP3	-4.702259641	0.015083237	0.0500322
PIK3IP1	-4.702786989	0.007695857	0.029866313
MPDU1	-4.704601666	0.001445963	0.008205312
STX10	-4.705349771	0.002646039	0.013035267
SRGAP1	-4.70624037	6.62E-05	0.000786742
FAM210A	-4.706418614	0.012273511	0.042762975
EBP	-4.708332836	0.036854947	0.097336305
UBN2	-4.709100645	0.005963121	0.024558564
RANGRF	-4.710134529	0.02650723	0.076610198
SLC38A6	-4.711225639	0.003296819	0.015478474
B4GALT5	-4.711327543	7.10E-05	0.000827214
STEAP3	-4.711903428	0.002774373	0.013540732
SDR39U1	-4.713126792	0.000132933	0.001326477
DNAL1	-4.713507158	0.011096067	0.039560174
MMP16	-4.714179147	0.003632996	0.016694677
YBX2	-4.714523556	0.028093567	0.08016137
TAS2R43	-4.716798989	0.031702517	0.087469174
SPAG5	-4.717744797	0.006969832	0.027714469
GRB14	-4.71815561	0.023628047	0.069993609
GRK3	-4.718313434	0.030538137	0.085247604
SWAP70	-4.718667504	0.000230891	0.002020754
AIMP2	-4.718743697	0.027179136	0.078160197
CCM2	-4.72009157	0.000813996	0.005251924
BYSL	-4.720475029	0.030363886	0.084910501
ZNF596	-4.720895007	0.000377275	0.002890625
NDUFA5	-4.722175612	0.002663746	0.013099725
SLC17A5	-4.722487174	0.049499582	0.121382281
GABRB3	-4.724716084	0.018955136	0.059330998
MTSS2	-4.725295184	0.013220358	0.045279914

SLC39A8	-4.725373184	0.033498086	0.090951285
IQSEC1	-4.725836023	0.005300799	0.022528397
POU6F1	-4.727264013	0.007147869	0.028255325
MRPL23	-4.727769087	0.002837428	0.013787328
BTBD9	-4.728093816	0.000507693	0.003629721
CYB561	-4.728339339	0.001597369	0.008824536
SLC3A1	-4.728369425	0.012575808	0.043571169
MORN1	-4.728380375	0.000130436	0.001309946
RFC4	-4.731934042	0.006480274	0.026154464
DNA2	-4.732032339	0.039745557	0.103103349
CD99L2	-4.732953543	0.001396854	0.008005455
AAR2	-4.732994494	0.003065076	0.014646539
MORN2	-4.733291134	0.014979642	0.049756746
NSRP1	-4.733949793	0.000102558	0.001079403
CFAP46	-4.735509104	0.01890563	0.059221984
SSR3	-4.735856998	9.07E-05	0.000993261
SNX16	-4.736664771	0.030617499	0.0853707
MEGF8	-4.737515002	0.00629353	0.025579881
TVP23C-CDRT4	-4.737885936	0.03555529	0.095065438
BAK1P1	-4.739397722	0.020292433	0.062409813
METTL15P1	-4.739605376	0.031459851	0.087037721
TARBP1	-4.739776677	0.000406063	0.003058628
MZT2A	-4.740169809	0.000218122	0.001934172
SPDYE7P	-4.743423636	0.019733973	0.061183403
SERAC1	-4.744229669	0.000291004	0.002404305
TUT1	-4.744373891	0.00024768	0.002132236
RAD21	-4.747607438	0.001703216	0.009249341
STEAP4	-4.748193776	1.37E-06	2.99E-05
DTX1	-4.749710715	0.028581961	0.081172234
EBF1	-4.749713709	0.007256277	0.028597607
SLC39A3	-4.750049043	0.005563776	0.023318541
SLC25A32	-4.750326277	0.00076079	0.004991684
PAIP2	-4.750470473	0.000202058	0.001818371
HSBP1	-4.751391285	0.002321419	0.011796162
TMED1	-4.751515169	0.030133901	0.084430726
CASP10	-4.752143052	0.001600906	0.008836011
RTKN	-4.752669974	0.000731193	0.004846855
PLAU	-4.752767227	0.013465751	0.045873753
DNAI7	-4.752896747	0.028250939	0.08051418
H2AC8	-4.753972527	0.008569868	0.032415527
CYP2A6	-4.754563746	0.047947072	0.118608493
NR5A2	-4.755605389	0.027976927	0.07988505
ANKRD46	-4.755988007	0.015108493	0.050102247
ZNF285	-4.756610673	0.007798032	0.030195306

GPD1	-4.756945799	0.018473229	0.058259499
FTH1P4	-4.758315846	0.004986686	0.021481465
CHMP1A	-4.758347617	0.000809229	0.005232323
TBC1D24	-4.758685783	0.00010381	0.001088798
CLCN2	-4.760382669	0.000233853	0.002042248
FUCA2	-4.760567223	0.027028418	0.077837729
AGER	-4.761251323	0.003067412	0.014651917
ENDOV	-4.761900684	0.004479559	0.019745295
TAGLN2P1	-4.762834832	0.014292852	0.047922628
PORCN	-4.763119089	0.00955322	0.035296758
DENND1A	-4.763259769	8.44E-05	0.000941097
TAF11	-4.763927406	0.035628115	0.095168465
NABP1	-4.765662776	0.002497632	0.012487127
KHDC1	-4.765839047	0.007844002	0.030345374
UGT8	-4.765841757	0.000355312	0.002765719
PSPH	-4.766071163	0.01048308	0.037854177
AK3	-4.767605647	1.75E-07	4.90E-06
FURIN	-4.768243751	1.67E-05	0.000259647
RPP38	-4.768622329	0.006059655	0.024846228
NDUFA9	-4.768644789	0.003994551	0.017989865
RYR3	-4.770972453	0.000307932	0.002508727
KPNA5	-4.7712065	0.000144871	0.001417553
MGST2	-4.771959674	0.012746335	0.044051076
RIOK2	-4.772177638	0.000678014	0.004579622
NUDCP1	-4.773233662	0.013301347	0.045454406
TMEM54	-4.773238054	0.000597536	0.004144743
CRYZ	-4.773714807	0.000203966	0.00182974
SLC2A8	-4.774031506	0.025479859	0.074225804
EXOSC10	-4.7743384	9.44E-06	0.000159978
RASGEF1B	-4.77568664	0.004823222	0.020915385
STK4	-4.775941159	1.10E-05	0.000181766
MRPL42	-4.776990377	0.025625352	0.074559918
SSBP3	-4.77835415	0.005477176	0.023097508
NQO2	-4.779040211	0.015388293	0.050742451
XPOT	-4.780578855	0.000553609	0.003898885
CHST15	-4.780791745	0.000207113	0.001855582
ZNF180	-4.780793988	0.046725969	0.116300869
ANO8	-4.781846906	0.02464974	0.072470354
ENSG00000256664	-4.782360773	0.005729436	0.023782269
LSM5	-4.782439631	0.000295572	0.002427136
ZNF570	-4.782583082	0.01708572	0.054811969
CCDC50	-4.783277115	0.008186793	0.031338693
LLPH	-4.783833246	0.00451588	0.019847572
VWA1	-4.784924225	0.003543758	0.016384128

PIM1	-4.786025711	0.020294533	0.062409813
RCN2	-4.786100273	7.06E-05	0.000823904
FLOT1	-4.786622152	0.048029814	0.118691889
EXT1	-4.788531694	0.002847543	0.013818275
WDR89	-4.78961872	0.003911424	0.017681986
EIF4BP2	-4.790159111	0.012785955	0.044129228
TSPAN32	-4.790165334	0.007685944	0.029855798
CYHR1	-4.790560484	0.004973651	0.02145441
NDUFV2	-4.79077362	0.00960158	0.035442978
MRPL24	-4.790859811	0.001809712	0.009683797
HLA-DMA	-4.791707774	0.032033768	0.088009522
SLC49A3	-4.793445181	0.019386844	0.06038558
CMC1	-4.793673673	0.017382816	0.055554048
HSD17B11	-4.793704828	0.009051116	0.033834066
PCDHGA9	-4.793994423	8.58E-05	0.000951709
FOSL1	-4.797467602	0.005013478	0.021564726
ATP5MKP1	-4.797756966	0.012711179	0.043958889
HNMT	-4.79791504	0.002205679	0.011346169
GFPT2	-4.800396052	0.0160224	0.052273619
TJP3	-4.801682845	0.000573969	0.004015794
COMMD5	-4.803267034	0.012560542	0.043537459
P3H1	-4.804486557	0.001915537	0.01016469
JOSD2	-4.804586509	0.015014409	0.049838296
DGAT1	-4.804676185	0.00031539	0.002557598
RASA2	-4.80490204	0.016380823	0.053127941
SRBD1	-4.805270509	0.004588954	0.020088498
ENSG00000258130	-4.807163169	0.002070862	0.01074859
LPXN	-4.80864398	0.006789673	0.027140754
RPA3	-4.808780952	0.027870124	0.079711715
UBE2Z	-4.809101606	2.57E-05	0.000372677
BHLHE40	-4.809227409	0.009748682	0.035898403
GUCY1A1	-4.809261232	7.69E-05	0.000880955
ZNF821	-4.809407901	0.005610461	0.023449249
SLC16A6P1	-4.80981512	0.012342503	0.042953897
MCOLN3	-4.81050196	0.025394188	0.074029685
CHAD	-4.811331553	0.001773663	0.009541517
IFFO1	-4.811378952	0.004929281	0.021308579
DNAI4	-4.812077476	0.000889324	0.005636997
REX1BD	-4.812588058	0.000111996	0.00115567
SDHAP3	-4.813317471	0.004096192	0.018335912
GBGT1	-4.813855328	0.003720974	0.017026525
MSRB2	-4.814132324	0.047560007	0.117916084
KIF20B	-4.814403117	0.002062328	0.010722688
UBE3C	-4.814899284	3.11E-05	0.000432671

ZNF205	-4.816114977	0.008995302	0.033698278
TRIM39	-4.816704894	0.012459413	0.043261254
WDR12	-4.817147442	0.005467792	0.023084113
MAF1	-4.817517116	0.001878962	0.010010103
VKORC1L1	-4.817712115	0.026467263	0.076512957
C12orf57	-4.818153693	0.025148985	0.07352737
ITIH4	-4.823137056	0.001271646	0.007442944
INSIG2	-4.823392856	0.011760798	0.041393633
EIF4HP1	-4.823430807	0.045975702	0.114937642
CD36	-4.823732587	0.000432063	0.003206431
TSPAN6	-4.825741753	0.033959773	0.091834024
MARK2	-4.826083086	0.000950123	0.005938642
UPRT	-4.827109099	0.008393219	0.03194702
RRM2	-4.827547007	0.025183017	0.0735829
SLC25A17	-4.827845066	0.00642767	0.026002846
SNX18	-4.829490662	0.001305747	0.007587502
ZNF721	-4.829505312	0.008591021	0.03247524
FAM185A	-4.830653233	0.000643876	0.004403096
PPP2R3B	-4.831050943	0.002544688	0.012644048
KLC2	-4.831855712	0.002522409	0.01256947
HSH2D	-4.83215731	0.002532649	0.012606845
MAP3K1	-4.834053947	0.021504318	0.065308976
KLF13	-4.834301595	0.006505682	0.02623077
REEP3	-4.834386542	0.011849326	0.04163258
RAP1GAP2	-4.834942793	0.001358078	0.007820253
ENSG00000219201	-4.835433808	0.043391298	0.110009644
ERCC2	-4.835499971	0.00473578	0.020619381
ISG20	-4.837038103	0.022711892	0.068062576
SLC9A7P1	-4.837305264	0.000723228	0.004807224
HIKESHI	-4.837725646	0.016472813	0.053326271
NSUN5P2	-4.837838187	0.001395539	0.008001708
NHEJ1	-4.83798544	0.017385883	0.055554048
MACIR	-4.838422934	0.046054131	0.115078284
MECOM	-4.839462438	0.006200212	0.025259969
LZTFL1	-4.839922176	0.006250381	0.025438671
THOC1	-4.84026886	0.004070645	0.018241795
RPS11P5	-4.840389835	0.029712216	0.083481119
PMF1-BGLAP	-4.84050988	0.017805788	0.05659697
EEF1A1	-4.840519574	7.95E-05	0.00090428
TIAM2	-4.840608667	0.002533034	0.012606845
LBH	-4.840629393	0.003370426	0.015763385
MCOLN2	-4.840683307	0.043893253	0.110879142
KLHL5	-4.842358024	0.013199479	0.04525743
TXNL4A	-4.842864376	0.000322176	0.002598617

HSF2	-4.843255491	0.044289981	0.11161481
LTN1	-4.843624432	0.001195867	0.007109415
UNC13B	-4.843940677	4.13E-05	0.000536661
TAP2	-4.844881446	0.000423093	0.00315996
POC5	-4.845396514	0.007564705	0.029507957
ZNF562	-4.845905551	0.000141312	0.001390604
DNHD1	-4.84601322	5.87E-06	0.000106984
PSMC3IP	-4.846497782	0.025467149	0.074206638
VSIR	-4.846554084	2.86E-05	0.000406028
SLC25A24	-4.847000354	0.000320865	0.002592622
MAMDC4	-4.84825644	0.000121174	0.001234589
FAM53C	-4.849071645	2.32E-06	4.72E-05
SLC35B2	-4.84996285	8.08E-05	0.000911812
TSHZ2	-4.850945904	0.00067461	0.004564268
BLMH	-4.851157808	0.000347372	0.002731085
BRCA2	-4.851878076	0.012967276	0.044665882
LRRC75B	-4.852103322	0.010091619	0.036812797
EVA1C	-4.852125427	0.026733189	0.07713433
KIAA1143P1	-4.852820445	0.006012394	0.024714715
RPL17P25	-4.852958347	0.010728275	0.038452781
NDUFB5	-4.853024597	0.000273711	0.002295908
TMEM126B	-4.853106623	0.004940899	0.021335939
RPF2	-4.853351366	0.028739025	0.081502385
RUNX1T1	-4.853813155	0.002209171	0.011354484
ADAP2	-4.854779817	0.003814007	0.017309613
RHOG	-4.856276519	0.001679047	0.009158712
NEMP2	-4.856445641	0.01156676	0.040841326
MRPS7	-4.856933231	0.024728861	0.072580512
SMC5	-4.859742893	0.031096502	0.086209817
IQCB1	-4.860035841	0.003939268	0.01779138
SCLY	-4.86017985	0.010642739	0.038239711
ATXN7L1	-4.860582093	0.0067487	0.027035959
SMIM15	-4.863071424	0.043256552	0.109741628
GPR161	-4.863514753	0.002034747	0.010615764
ELMO3	-4.863744966	0.002626681	0.012961007
RHOQ	-4.86611126	0.011593065	0.040910338
SUMO2P8	-4.866274244	0.043254806	0.109741628
CLK2	-4.866351914	5.39E-05	0.000664875
NFATC2IP	-4.866570061	0.000475145	0.003456223
RUVBL1	-4.867975592	4.94E-05	0.000617407
USP42	-4.868467323	0.000350477	0.002743968
RDH10	-4.868556461	0.017864504	0.056741677
THRB	-4.869234897	0.027300454	0.078453156
ESS2	-4.869263958	0.001043406	0.00636562

PPAT	-4.869646031	0.012682662	0.043885346
DBP	-4.86975575	0.001342585	0.007756381
SNRPE	-4.869857182	0.022033278	0.066456715
HMCN1	-4.870602539	0.043588933	0.110331944
RORC	-4.870917708	0.00975212	0.035900138
RNF215	-4.871188634	0.000596599	0.004140614
SCML1	-4.87147631	0.01124944	0.039965724
RHNO1	-4.871710975	0.001284575	0.007489644
RPL36AP16	-4.872137398	0.011133451	0.039646746
ADAP1	-4.872345829	0.00104967	0.006390951
IRAG1	-4.873317766	2.74E-05	0.000393073
DENND2D	-4.874141071	0.000791679	0.005160192
BHMT2	-4.87415564	0.013299186	0.045454406
TXNDC17	-4.874777431	0.015389585	0.050742451
TOM1	-4.875464772	0.00042319	0.00315996
CMTM6	-4.875749038	0.002135761	0.011041933
TMEM30B	-4.875895284	3.13E-05	0.000436021
LRRC57	-4.875896311	0.000286456	0.002376468
POU5F1P3	-4.876806333	0.043943593	0.110949781
C6orf120	-4.877727399	0.010661855	0.038294092
SCO1	-4.87789461	0.002586208	0.012824034
HLA-DRB1	-4.879148444	0.014976663	0.049756746
PDE4A	-4.88065187	0.000537883	0.003814022
FAM107B	-4.88069771	0.00604043	0.024784191
DCUN1D3	-4.880797731	0.000283966	0.002360661
TAS2R15P	-4.881841429	0.001944271	0.010281111
MDK	-4.882538388	0.028415964	0.080833567
JAK2	-4.884555015	0.003093433	0.01474711
HSPA7	-4.88546369	0.000799641	0.00519252
VOPP1	-4.885514004	0.004057532	0.018216754
DCLRE1A	-4.88592526	0.001209737	0.007167234
TRAF7	-4.886083695	0.000153207	0.001481162
EMC7	-4.886147225	0.010634527	0.038238807
CYBRD1	-4.887007663	0.000293667	0.002416415
PAK4	-4.887115538	8.55E-05	0.000949284
ZBTB22	-4.888152767	0.024171631	0.07135937
CYCS	-4.888986233	0.015244312	0.050442086
ZBTB47	-4.88910918	0.012524873	0.043438701
SNAP23	-4.889996418	0.000160664	0.001524042
CHERP	-4.890361544	0.00056048	0.003935256
HLA-H	-4.890980765	0.047630476	0.118066615
CBX1	-4.892365445	0.001924024	0.010196316
MICA	-4.892871825	0.008140803	0.031204378
CYB5D1	-4.893504066	0.006469514	0.026137181

PAK3	-4.893789365	0.049396274	0.121202615
ERI1	-4.894841709	0.006947558	0.027644064
C2CD2L	-4.894996819	0.010338373	0.037438876
ASNSD1	-4.896042285	0.01791071	0.056870733
PSTPIP1	-4.896548582	0.007516495	0.029338811
SPACA9	-4.89793433	0.01459404	0.048730003
DNASE2	-4.89856444	0.012336459	0.042951696
ZNF765	-4.899881314	0.012396031	0.043090627
SIRT2	-4.900422228	9.53E-05	0.001024154
CEP76	-4.901657698	0.010792318	0.038647992
GATA6	-4.903437358	0.013703709	0.0464101
TDRD3	-4.90353433	1.08E-05	0.000179246
PHETA1	-4.90365092	0.000440342	0.003253908
TGFB1	-4.903796412	0.01096649	0.039178983
LEPROTL1	-4.904063612	0.00282031	0.013720671
EPHA1	-4.90587666	0.010893777	0.038976731
MFSD6	-4.906167624	7.57E-05	0.000870441
FAM131A	-4.907134122	0.001078108	0.006541063
SMYD2	-4.908409861	0.010158097	0.03697988
SGCB	-4.908504666	0.004608011	0.02015006
CCNK	-4.908784677	9.74E-06	0.000163904
TSSC4	-4.909154034	0.004939434	0.021335939
ANKH	-4.909361315	2.76E-05	0.00039451
RPS6KA5	-4.90955411	0.002866174	0.013887977
LIPA	-4.909934401	8.46E-05	0.000941619
FERMT3	-4.910284007	0.013081554	0.045008281
SIN3A	-4.910464626	8.51E-07	1.98E-05
C21orf62	-4.911284608	0.04259081	0.108416227
NAPSA	-4.911395661	0.041347533	0.105986983
BCL2L2	-4.91268148	0.044997268	0.112997289
INSYN2A	-4.913838605	0.006805054	0.027193258
SLC7A8	-4.913869908	0.001289687	0.007515826
LINS1	-4.914798567	4.20E-05	0.000543147
SLC25A22	-4.916143991	0.003218239	0.015206009
ATAT1	-4.916995619	0.000604821	0.004188077
SEPTIN6	-4.917358944	0.001097007	0.00661923
CKAP2	-4.917808992	0.002540769	0.012629759
PHYHIP	-4.917974321	0.008498928	0.032248097
CCDC146	-4.918884892	0.001444014	0.008198101
TAB1	-4.919244756	0.011294435	0.040102037
ASF1A	-4.920130067	0.011467	0.040588259
MRPL15	-4.921338055	0.022407993	0.067452461
ZNF517	-4.921340009	0.002644908	0.013035002
SCN1B	-4.921353236	0.020472807	0.062878166

NFYB	-4.921359865	0.002188357	0.011271437
ZNF516	-4.921447037	0.000282646	0.002351302
PGAP2	-4.921824571	0.002361355	0.011968946
ZNF404	-4.921877535	0.005976423	0.024604975
EPHB1	-4.92265291	0.023616258	0.069984025
PIK3CD	-4.922849079	0.021332033	0.064858812
AGBL5	-4.924553197	0.000351081	0.002746923
ZNF776	-4.926095689	0.01576576	0.051729128
PPARGC1A	-4.92694112	0.000187456	0.001713721
DAZAP2	-4.927477331	2.85E-05	0.000403853
ODF3B	-4.928908705	0.000111423	0.001150742
CCL14	-4.929586357	0.041393177	0.106059062
ZNF614	-4.93007382	0.004502684	0.019796763
LIN37	-4.930722406	0.042784064	0.108770912
SERINC5	-4.931358146	0.001600344	0.008836011
CCDC189	-4.931431979	0.011185472	0.039773489
IFI44L	-4.931630556	0.008803556	0.033092622
CREB1	-4.93278329	0.000274939	0.002301426
POP5	-4.93344202	0.012756033	0.044051076
EAPP	-4.934564782	0.023459764	0.069714462
FAM98A	-4.934778881	0.001014712	0.006240893
BCAS2P2	-4.935802256	0.002614731	0.012919084
SEC14L2	-4.936179718	2.66E-05	0.000383262
ZNRD2	-4.936781006	0.009578793	0.035380443
RPL22	-4.937323144	0.000699621	0.004683747
RPL23AP18	-4.937434314	0.045324374	0.11367721
N4BP2L1	-4.937697248	0.000246705	0.002126864
GAB2	-4.937739342	0.000622988	0.004291775
METTL25B	-4.938250071	0.000138752	0.001372104
ZNF174	-4.938880682	0.040135105	0.103687208
TRIM47	-4.939460403	0.022200495	0.066911054
RNASEH2C	-4.939483633	0.039788838	0.103127378
CCDC150	-4.939785371	0.00119913	0.007118241
FSD1L	-4.940646459	0.012093216	0.042244239
STX2	-4.941482781	0.030581309	0.08532876
SLC30A7	-4.942116252	0.001585254	0.008785676
PIK3R2	-4.945446158	0.028401529	0.080830129
PLA2G15	-4.946565284	0.005797947	0.024017233
RPSAP9	-4.946603495	0.003908969	0.017680929
FOXO4	-4.946648766	0.000153825	0.001483582
PGAM5	-4.946993975	0.02167298	0.065713864
RHEB	-4.947242307	0.000806839	0.005219656
PA2G4	-4.947661511	0.00626226	0.02547845
KARS1	-4.949848315	0.00093005	0.005838437

PPT2-EGFL8	-4.950839966	0.010406294	0.037614533
DHRS7	-4.950926808	1.69E-05	0.000262173
PRDM11	-4.951392886	0.001792076	0.009606418
CCDC157	-4.951904931	0.000955602	0.00596832
EDNRA	-4.952080591	0.001572929	0.008733361
PGLS	-4.952182491	0.003483711	0.016162067
TIMM17A	-4.953430644	2.71E-05	0.000390522
RAD51B	-4.953530523	0.000726904	0.004829007
PLEKHG3	-4.95379313	0.004439198	0.019603085
CUEDC2	-4.954069552	0.000343183	0.002709646
IRF4	-4.954660815	0.008931684	0.033501427
LDHAP2	-4.955056985	0.007941455	0.030632051
LDB2	-4.956658019	0.000583421	0.004067813
SLC2A9	-4.957120282	0.024783613	0.072652179
BLOC1S1	-4.957445032	0.021709204	0.065765563
PTTG1	-4.957890124	0.001123821	0.006750733
FAM118B	-4.958803197	0.000332874	0.002657677
PRR4	-4.959487965	0.022969758	0.0686145
AVL9	-4.95973673	1.67E-05	0.000259338
BIRC7	-4.959894964	0.026838548	0.077364561
ARL6IP1	-4.960189044	9.48E-05	0.001020164
PKN3	-4.960314676	0.026453759	0.076492189
CACNA1D	-4.960914113	9.44E-05	0.001016584
TNFRSF10A	-4.96192699	0.02150707	0.065308976
FAAP100	-4.961945125	0.000132475	0.001323003
SVEP1	-4.962244174	0.014186616	0.04768531
CASP7	-4.962348576	0.020490951	0.062917928
ALAS1	-4.963402777	0.000317849	0.002573411
TMCO3	-4.963854658	0.000101768	0.001073091
MUC4	-4.964139394	0.009018006	0.033742516
GRPEL1	-4.965123324	0.018311657	0.057870573
PITPNM3	-4.965610358	0.006039314	0.024784191
CNNM4	-4.965969835	0.021282734	0.064790296
CIAPIN1	-4.968298428	0.006393909	0.025900895
SMPD2	-4.968615719	0.040813416	0.104849823
BOP1	-4.969149918	0.000925077	0.00581367
DNAJC15	-4.970185753	0.009264596	0.034451204
ALKBH8	-4.971892194	0.01039713	0.0375999
ACY1	-4.972124354	0.000273962	0.002296425
ZNF767P	-4.972636149	0.000169564	0.00158978
PDE1C	-4.973034007	0.029521352	0.08309808
ETV6	-4.973514829	0.005110764	0.02187436
XYLB	-4.976285533	0.030902536	0.085927932
CCDC61	-4.976668926	0.004958162	0.021402851

TMEM214	-4.977533815	0.001052192	0.006403083
HINT1	-4.978851879	0.042445186	0.108136504
TGIF2	-4.979108887	0.000621471	0.004283764
IGLV3-25	-4.979178022	0.039916271	0.103365024
ESRRG	-4.980013358	0.00249561	0.012482175
KAT2B	-4.98002666	0.000961803	0.005994675
UTP11	-4.980451099	0.013114741	0.045071215
ACTG1P14	-4.980762418	0.011116569	0.039621599
ERO1B	-4.981053177	0.004988797	0.021481465
FAT2	-4.981171076	0.044688653	0.112362164
APLF	-4.981907209	0.00031824	0.002574847
ZNF859P	-4.982701089	0.041291134	0.105887263
OSBPL6	-4.983395393	0.006289589	0.025572449
SLC25A10	-4.98373132	0.023475777	0.069714462
MSH5	-4.984636696	0.020590215	0.06319066
ZHX2	-4.984835075	0.022900999	0.068476702
PCDH9	-4.98557508	0.000138711	0.001372104
ACSL5	-4.985928163	0.001252412	0.007362408
ZNF706	-4.986655575	0.000344857	0.002719318
COG5	-4.98675913	0.023846229	0.070484678
TCEA3	-4.986820219	0.030601197	0.085363111
GNE	-4.986934211	0.00288319	0.013948094
SMIM14	-4.987213478	2.96E-06	5.86E-05
MYOM1	-4.988773194	0.020980779	0.064080583
PRPSAP1	-4.989613344	0.000295874	0.002427971
KSR2	-4.990777838	0.00408136	0.018283042
DTNB	-4.992034087	0.00431824	0.019191415
ACAT1	-4.992721464	0.000179818	0.001657672
CYP2E1	-4.993445807	0.004045118	0.018167757
PRDM4	-4.994650608	0.000155167	0.001490585
ENSG00000269069	-4.996715398	0.000449743	0.003308543
SGMS2	-4.996850467	0.000112536	0.001160252
KCNJ13	-4.997242835	0.027284353	0.078425505
LZTR1	-4.998143629	0.000169382	0.001589303
CMAS	-4.999032863	0.003037245	0.014542253
TAS2R30	-4.99904919	0.006992431	0.027786073
RPS6KA1	-4.99969007	6.23E-06	0.000112791
NNMT	-5.000014222	0.022766295	0.068162488
INTU	-5.000017849	0.000105484	0.001100669
HAUS4	-5.000255036	0.020915307	0.06392901
PTDSS2	-5.001333037	0.039048388	0.101647532
KCTD5	-5.001454486	0.0249329	0.073036743
CTDSPL	-5.001499411	0.000566645	0.003971433
MS4A14	-5.001566206	0.021141419	0.064473603

ADGRB3	-5.002632848	0.019596112	0.060818293
TRDMT1	-5.002682453	0.000337564	0.002679263
FHIT	-5.003014981	0.015125196	0.05014053
PCGF1	-5.003311786	0.026941572	0.077624564
NUDC	-5.003413781	0.010524572	0.037958708
TRIM39	-5.006772467	0.010571588	0.0380878
LSS	-5.007926726	0.000444906	0.003283621
ITM2A	-5.010054681	0.002448529	0.012297509
WNK4	-5.010237347	0.004894664	0.021196785
TSPAN18	-5.0107972	8.31E-05	0.000931315
WRNIP1	-5.011391374	8.25E-05	0.000926388
FAM221A	-5.013862898	2.00E-06	4.18E-05
TMED7-TICAM2	-5.01448852	0.006374833	0.025832265
GNPNAT1	-5.014573198	0.00147043	0.008312975
IMPDH1P5	-5.014589276	0.01050315	0.037904034
ESR2	-5.016215657	0.000540578	0.003826409
NFE2L3P1	-5.016495258	0.001462753	0.008273438
ZNHIT3	-5.017501272	0.018440322	0.05817649
CBWD1	-5.017821834	0.000188231	0.001718213
MSR1	-5.018253065	0.041504444	0.10629915
AGTRAP	-5.019638749	9.22E-05	0.001003427
FIBP	-5.020392945	9.34E-05	0.001013392
ZNF654	-5.020480914	0.009059207	0.033853858
CCDC66	-5.02148336	6.75E-05	0.000797729
LRRC49	-5.021602848	0.000575612	0.004020314
SELENOW	-5.02185768	3.70E-06	7.10E-05
NAIP	-5.021941642	0.01153344	0.040759346
FCGR1B	-5.022403604	0.040774191	0.104783611
FBXW7	-5.02308871	8.88E-06	0.000152364
MCC	-5.02334216	0.000329041	0.002635813
ADARB1	-5.025705779	0.000491115	0.003542584
G2E3	-5.026337316	0.00210885	0.010922344
ARHGEF6	-5.027473924	0.002170731	0.01119494
UVSSA	-5.02802177	4.93E-05	0.000616757
PER2	-5.028402995	0.000193117	0.001753824
CD83	-5.028708767	0.037555275	0.098690632
HSD17B7P2	-5.028963699	0.023804572	0.070378735
DNAAF9	-5.030129085	0.012419493	0.04315979
IRF6	-5.030288885	0.000393309	0.002979105
PES1	-5.032387666	0.001189982	0.007081387
TEFM	-5.034579922	0.011633869	0.041042364
CEP44	-5.035412128	0.000853798	0.005451339
THYN1	-5.036695566	0.004535549	0.01988348
MFSD13A	-5.037631751	0.00157139	0.008728824

MAP3K10	-5.038708421	0.004254858	0.018955025
KCNN3	-5.039306708	0.019568476	0.060748096
PFDN5	-5.04049655	7.04E-05	0.000822139
NSMCE4A	-5.041416244	0.003422743	0.01597104
SPCS3	-5.041648413	0.000547059	0.003865501
ZNF23	-5.04271126	0.000821704	0.005284756
RPSAP36	-5.043040945	0.011531309	0.040759346
ENSG00000231181	-5.043763107	0.019279275	0.060112403
TMEM69	-5.043837444	7.56E-05	0.000869807
ZBTB12BP	-5.044319277	7.49E-06	0.000131341
SCYL1	-5.044443585	2.19E-06	4.51E-05
GGCT	-5.044514477	8.80E-05	0.000971336
RELN	-5.044842523	0.00027454	0.002299675
PPM1M	-5.045019695	0.001224374	0.007237894
ZNF175	-5.045192924	0.009230869	0.034389177
ARMC10	-5.04546179	1.95E-05	0.000296843
TCP11L1	-5.046042418	0.004382757	0.019417604
ENSG00000256966	-5.047033434	0.003441865	0.016023204
ASL	-5.047171518	0.005586684	0.023390255
MED27	-5.047929158	0.00649001	0.026176303
ENSG00000213976	-5.048912362	0.023028589	0.068768273
AKR1A1	-5.050147928	1.43E-05	0.000227833
CIAO2A	-5.050196522	0.000411897	0.003092801
TTC7B	-5.050319657	0.002009857	0.010513099
ANKMY2	-5.050552853	0.019677309	0.061038992
FRS2	-5.051709479	0.005520739	0.02320344
PFKFB2	-5.051735588	0.001390542	0.007976834
SNU13	-5.052244213	0.001179935	0.007029502
ADSS1	-5.052657558	0.005527662	0.023215414
RAMP2	-5.05369274	0.010151291	0.03697988
ARMCX7P	-5.055769349	0.012073928	0.042225607
GCFC2	-5.056005658	0.000249017	0.002139176
ENSG00000283782	-5.057246333	0.015918586	0.052061561
GPATCH4	-5.057427101	0.012609144	0.043655914
SLC5A9	-5.058725397	0.003258943	0.015348733
MRTFA	-5.060226508	0.004481764	0.019747823
MAGED4	-5.060251693	0.015821968	0.051843286
DNTTIP1	-5.060274786	0.000169157	0.001588422
RRP1	-5.060621915	9.06E-05	0.000993154
EXOSC7	-5.061728664	1.60E-05	0.000249345
FAM3A	-5.062413313	0.000109279	0.001133808
ORAI3	-5.062575896	0.018651889	0.058588962
ENSG00000213985	-5.06277435	0.003732542	0.017042129
NAT9	-5.06279424	0.003950093	0.017833616

EHHADH	-5.063461693	0.009520232	0.035207114
NPIPB14P	-5.063591554	0.002939361	0.014185818
NPM1P7	-5.064130682	0.015312014	0.050563013
PIAS3	-5.065243601	5.32E-07	1.34E-05
ZNF17	-5.065286704	0.001410618	0.008063531
METRNL	-5.06595083	0.008903398	0.033447155
CNOT3	-5.067508595	0.022322319	0.067228003
SLC19A1	-5.067889149	0.004263217	0.018985273
ZCCHC9	-5.068818059	0.007532662	0.029392439
RFK	-5.069326028	0.005478598	0.023097508
SMYD5	-5.071124382	0.018533494	0.058373515
PRKCZ	-5.072076492	3.86E-05	0.000509838
PPARA	-5.072423259	0.000133555	0.001329377
TSPAN7	-5.07371833	0.023283758	0.069347097
PHAF1	-5.074374178	0.00145198	0.008227888
MTERF3	-5.075493427	0.014102145	0.04747124
SF3B4	-5.075780872	0.003706734	0.016981949
HAUS5	-5.077299148	0.000280609	0.002339183
KIF3A	-5.077520963	5.29E-05	0.000653437
TTC39C	-5.077707547	0.000118175	0.001209124
CPPED1	-5.078828797	0.02922268	0.082418041
NDUFA10	-5.078884137	1.30E-06	2.87E-05
ENPP5	-5.079175892	0.001997871	0.010486583
JOSD1	-5.080391534	0.004061709	0.018228746
ZNF529	-5.081148533	0.002556399	0.012697029
SUPT4H1	-5.081460546	0.006965063	0.027704607
NAMPTP1	-5.082505086	0.021049259	0.064260919
WDR24	-5.084283515	0.003242732	0.015290231
MTG2	-5.084667664	0.001409922	0.008063531
AASDHPPT	-5.085171583	0.005140687	0.021986881
GTPBP1	-5.085949672	0.000146114	0.001428564
CCDC82	-5.085958138	0.000251334	0.00215145
IGSF6	-5.089108805	0.0066447	0.026702341
CHD1L	-5.089137684	2.99E-07	8.00E-06
ARNT2	-5.089547938	0.001141887	0.006849059
COPE	-5.089689933	0.000517269	0.003685123
BLZF1	-5.091147239	0.000249917	0.002143865
RABL2B	-5.092322281	0.000171446	0.001601252
ETV3	-5.092972277	0.011913462	0.041809379
PEAR1	-5.093258451	0.006045331	0.024795893
DPY19L2	-5.094205606	0.001592627	0.008802352
ATP7B	-5.095479631	0.009488347	0.035114508
OSGIN2	-5.096096199	0.000121826	0.001238104
POLR2I	-5.096237544	0.006128412	0.025034864

MACC1	-5.097621618	6.90E-05	0.000809238
TPST2	-5.098059531	0.001352485	0.007793539
IL12RB2	-5.098313656	0.036632489	0.096896775
RNF144B	-5.098409845	0.009782987	0.035980939
ROBO3	-5.098624609	0.009429183	0.034934446
TRIM73	-5.099111012	0.00011059	0.00114311
LUM	-5.09962319	0.03589624	0.09563266
TXK	-5.100320969	0.008741587	0.032880101
ZNF253	-5.100841624	0.000312253	0.002538288
GABPA	-5.101497329	0.035487452	0.094988969
L3MBTL2	-5.101629452	0.000455439	0.003340993
BTF3L4P2	-5.102322111	0.003064834	0.014646539
EXOSC1	-5.103209781	0.000656775	0.004468577
BRCC3	-5.104005736	0.035736895	0.095361416
CRELD1	-5.104325667	0.017276258	0.05526211
LRRC47	-5.105095987	0.003080081	0.014695035
KCTD20	-5.105310078	0.00012275	0.001246448
MINDY4	-5.105570735	0.000898193	0.005674182
MRPL58	-5.109464838	0.036364831	0.096420573
SAP30L	-5.110384786	0.000242708	0.002103497
CRYBG1	-5.110547874	0.0119989	0.042023924
IL11RA	-5.111958376	0.000572133	0.00400526
DHX34	-5.112332087	0.01384504	0.046770964
TMEM91	-5.11293624	0.009427696	0.034934446
ZNF66	-5.113650879	0.012553238	0.043524605
SHMT2	-5.114369078	0.000728652	0.00483531
RNF8	-5.114655214	0.001976012	0.010417097
SUFU	-5.114664274	0.018692032	0.058674366
NETO2	-5.114847685	0.020029712	0.061815306
MSRB3	-5.114901569	0.000104597	0.001092356
TMEM219	-5.115548086	0.001920575	0.010182735
SGK3	-5.115710498	0.000128226	0.001292293
GLMN	-5.115799901	0.000121751	0.001238104
TMEM237	-5.115881657	0.008097801	0.031068379
TUBGCP5	-5.11627178	0.002012201	0.010520815
RAB6A	-5.117734851	3.18E-05	0.000438339
MEST	-5.11928392	0.018947402	0.059322129
PHC2	-5.120551701	0.001756443	0.009486832
ZCRB1	-5.120652846	0.034208568	0.092300603
ZNF793	-5.122563321	3.98E-05	0.000521403
OCIAD2	-5.122749795	0.002820049	0.013720671
SUCLA2	-5.122860554	0.004320605	0.019191415
WDR83OS	-5.124152606	0.000223334	0.001967423
ALG1	-5.124550537	0.003254606	0.015334274

LRIF1	-5.126500826	0.00703945	0.027904847
ENSG00000268790	-5.126653441	0.00631691	0.025649071
TCF20	-5.126828683	0.009067632	0.033874882
TMEM176B	-5.12694995	0.021933543	0.066238423
COL4A3	-5.127438558	0.0004959	0.003566473
ZNF396	-5.1275019	0.024524236	0.072197432
HSD17B14	-5.128069388	0.001199704	0.007118241
C19orf54	-5.128124781	0.008154251	0.031243766
PRNP	-5.128631364	0.000352246	0.002754252
ZNF112	-5.128829152	0.00239175	0.012082528
CYB5R2	-5.128923826	0.022769696	0.068162488
C3orf52	-5.130217997	6.34E-05	0.000760291
TRIM6	-5.13089404	0.00138275	0.007939659
ENSG00000240695	-5.131653888	0.003028973	0.014514129
FSTL3	-5.131914864	0.000819962	0.005276355
POLG2	-5.133028256	0.019554687	0.060720866
SLC1A1	-5.134259713	0.034122034	0.092151948
ZFP2	-5.134626531	0.017118609	0.054888381
HLA-DRA	-5.135006418	0.01589131	0.052010993
SLC35F2	-5.135033843	0.008321505	0.031753939
DCUN1D1	-5.135806394	7.46E-05	0.000860223
SLC26A5	-5.136133906	0.009960054	0.03646331
ZFAND5	-5.136942642	2.54E-05	0.000369257
ANKRD20A1	-5.139251976	0.005812397	0.024052395
LYSMD4	-5.141396902	0.009617264	0.035490049
SLC4A3	-5.142362382	0.017683171	0.056325554
F8	-5.142757618	0.00104693	0.006377474
FLACC1	-5.143078667	0.008284609	0.031643076
PPP6C	-5.143482508	3.20E-05	0.000439607
ASB7	-5.144006037	0.01877288	0.058882338
QRICH2	-5.145707072	0.003447162	0.016041696
SENP1	-5.148054149	0.000278298	0.002321512
TGFBR1	-5.149248051	6.67E-05	0.000791876
CEP20	-5.150014515	0.000910935	0.005739815
LSM8	-5.150594845	0.013562519	0.046065658
CACYBP	-5.153109248	0.001711348	0.009276378
TMX4	-5.153149915	0.008018112	0.030849086
PAQR3	-5.153631997	0.00740247	0.02898722
NOTCH4	-5.155342783	7.32E-05	0.000849081
VWA5B1	-5.155663858	0.003033779	0.014531404
HS2ST1	-5.15796543	0.002661078	0.013093369
ENSG00000246596	-5.15813215	0.001408911	0.008063531
SNRPGP15	-5.158132982	0.026395659	0.076397191
ENSG00000235105	-5.158631907	0.000486538	0.003517954

ZNF551	-5.158812775	0.002817235	0.013718904
BTNL9	-5.159049969	0.004411024	0.019507445
RTL5	-5.159421729	7.35E-05	0.000851526
HSPB11	-5.159795494	0.000611135	0.004226961
BBS4	-5.160726931	0.000183173	0.00168091
DECR1	-5.160736711	0.0002259	0.001985687
MPP1	-5.162961091	0.000436957	0.003233023
TCHP	-5.165362826	0.000114533	0.001176828
RAD52	-5.166069345	0.00366522	0.016817218
ENSG00000283201	-5.166644223	0.018804015	0.058955965
AMOT	-5.166657815	0.000683274	0.004610001
EMID1	-5.166886938	0.002487555	0.012457329
FECH	-5.167201761	0.000535137	0.003799004
CHMP7	-5.167459171	9.93E-05	0.001054296
ICA1L	-5.168295927	8.24E-05	0.000926388
TREH	-5.168677969	0.007007442	0.027834835
SULT1A3	-5.168985265	0.002831677	0.013764908
TECR	-5.169319851	0.000168412	0.001582652
AXL	-5.170975234	0.000645513	0.004409307
SANBR	-5.171351052	0.001911434	0.010147367
PITPNA	-5.171362218	0.000186973	0.001711798
AGR2	-5.171484055	0.018113514	0.057454396
MTURN	-5.17222271	0.001280285	0.007470701
LRRC70	-5.17285393	0.002250292	0.011507194
MVK	-5.173425518	0.002664534	0.013099725
GPAT4	-5.17429673	2.10E-05	0.000313777
RPL7P57	-5.174463135	0.006644421	0.026702341
GATA3	-5.175053408	0.011911358	0.041809379
ZMAT4	-5.176341343	0.03568742	0.0952714
KY	-5.176855517	0.000791223	0.005159999
AAGAB	-5.177633795	0.001934365	0.01023752
PLPP5	-5.178584921	1.60E-05	0.000249345
REPS2	-5.178938812	0.006835494	0.027296874
SLC10A7	-5.179062469	0.016943373	0.054498714
SSBP1	-5.180484697	0.00797724	0.030730909
DUSP22	-5.180574305	0.000326543	0.002625618
KDM7A	-5.18113065	0.038628284	0.100875243
TCEAL1	-5.181554551	0.00357969	0.016499834
CCDC149	-5.182881499	0.000438592	0.003242956
TRPC1	-5.182936427	0.008068676	0.030994369
ZNRF3	-5.182941351	0.002749953	0.013426959
GALNT2	-5.183180336	0.001512635	0.008476361
PRDM15	-5.183190384	0.010407392	0.037614533
RRAGB	-5.183546293	0.002181877	0.011247632

SMO	-5.184564872	0.010361356	0.03749293
RAB7A	-5.185846899	0.000157541	0.001505031
STXBP4	-5.186916497	0.000468762	0.00341595
FAM72A	-5.189220649	0.032139858	0.088253367
GPR107	-5.189515918	8.83E-05	0.000973601
SMARCE1	-5.189831973	0.021645032	0.065662022
GPRC5D	-5.190317671	0.009823222	0.036117945
NDUFB4	-5.191005198	0.001086752	0.006577023
SELENOS	-5.191288804	0.004614059	0.020161938
GNPDA2	-5.191556448	0.008595219	0.032480964
CNTNAP3B	-5.192632521	0.002649013	0.013039307
BCS1L	-5.192785008	0.003889973	0.017608164
PSMD13	-5.194191258	1.31E-05	0.000212204
ENSG00000273047	-5.196237613	0.002313891	0.011767791
WDR87BP	-5.196516303	0.004464664	0.019686812
FKTN	-5.197163647	0.002593383	0.012853444
NAGS	-5.197951782	0.005551697	0.023277264
KLF3	-5.198373892	0.000647606	0.004418613
MUC20	-5.198702975	0.012505813	0.043385026
SPCS2	-5.199111479	0.000416537	0.003121732
RRN3P1	-5.199805392	0.000377423	0.002890625
CBR4	-5.200091674	0.008611997	0.032513914
FAM86DP	-5.201442053	0.005945561	0.024502918
TMOD2	-5.201451871	0.004403579	0.019488452
SLC26A6	-5.201984382	0.003603965	0.016586461
SEH1L	-5.202337836	1.71E-05	0.000263492
ADGRA2	-5.203054128	0.017143588	0.054924826
PHF6	-5.20343133	0.002970484	0.014307496
RPS4XP6	-5.204399958	0.003449544	0.016045974
AIDAP1	-5.205413248	0.000113519	0.001169353
CABYR	-5.205799231	0.031711916	0.087475167
YIPF4	-5.207393928	0.001530687	0.008557707
GPR176	-5.207616491	0.004545072	0.019918013
ATP6V1B2	-5.21000202	4.88E-05	0.000613241
TMEM37	-5.210039222	0.001724335	0.009331675
ULK4P2	-5.210438804	0.013549954	0.046043976
SMIM8	-5.210816682	0.010946919	0.039132164
VTI1B	-5.210997233	3.58E-05	0.000482114
SFXN4	-5.211039164	0.010710781	0.038424214
MMP19	-5.21112516	0.001280709	0.007470701
IDH3B	-5.211309631	2.60E-05	0.000375053
SMN2	-5.213091411	0.021701348	0.065765563
NGDN	-5.214020425	0.007387864	0.028958128
COPS4	-5.214109176	0.000123524	0.001253257

TAPBP	-5.2152191	1.22E-06	2.72E-05
ENSG00000237493	-5.216904324	0.010276162	0.037284971
TNFSF10	-5.217686776	0.001316507	0.007620758
QRSL1	-5.21809182	0.001541262	0.008605565
HCCS	-5.218973757	0.009769156	0.035940991
HR	-5.21957186	0.009723197	0.035815453
GLT8D1	-5.219907093	0.000363655	0.00281078
UXT	-5.220114183	0.000316908	0.002567505
HLA-DPB1	-5.220567444	0.004491699	0.019770008
OGFOD2	-5.221216476	3.92E-05	0.000515043
CDK19	-5.22179484	0.001414025	0.008073283
ANKRD20A21P	-5.222165209	0.010327379	0.037415535
IDNK	-5.222887794	0.017845901	0.056709579
DDAH1	-5.223330765	0.031769773	0.087594421
RMDN2	-5.223973619	0.01995499	0.061681805
TMEM163	-5.224093701	0.009049147	0.033834066
AGPAT1	-5.22573478	0.000293404	0.002415894
TSSK4	-5.225794384	0.00063167	0.004341703
TRAPPC12	-5.226833392	0.005656412	0.023624987
EAF1	-5.227006739	0.009667614	0.035654114
FUBP3	-5.227785641	0.003773504	0.017157963
CLYBL	-5.22828787	0.010231754	0.037164803
GSTT1	-5.22842002	0.00151343	0.008476891
RAE1	-5.229232345	0.002027969	0.010584966
HILPDA	-5.231968264	0.000907793	0.005725858
CDKN1A	-5.232734416	1.03E-06	2.36E-05
CFB	-5.232968145	0.010717268	0.038436092
RPP30	-5.232996524	0.002787906	0.013601295
WDR47	-5.23364703	0.001389089	0.007972276
IQCC	-5.23383945	0.008292236	0.031662216
IGHV4-34	-5.234208134	0.033620168	0.091160062
UBE2A	-5.234454661	0.000363282	0.002810753
NAGPA	-5.234533591	0.008514572	0.032268153
WDR53	-5.234752463	0.010136985	0.036946122
CYP7B1	-5.234862121	0.016950666	0.054498714
CEP89	-5.237027523	0.001758407	0.009488593
PPP3CC	-5.23965243	0.000154335	0.001486135
KRT86	-5.240631159	0.002961609	0.014276111
WDR3	-5.241382749	0.00149883	0.00843022
KCNQ3	-5.241384387	0.000353321	0.002757314
RFNG	-5.241425681	0.000889295	0.005636997
TRUB2	-5.24146305	0.007255898	0.028597607
MYRIP	-5.243503261	0.016902689	0.054426748
DCAF11	-5.244325008	1.04E-05	0.00017367

PRR29	-5.244874269	0.007258675	0.028597607
RGS14	-5.245573085	0.002977594	0.014330339
RAB29	-5.245650307	0.000275483	0.002302994
RPL13AP25	-5.246168826	0.000663642	0.004502644
MRPS34	-5.246278345	0.01608353	0.052402153
ATP5PD	-5.247680328	0.000153711	0.001483582
MUC20P1	-5.247964325	0.002499527	0.012491444
RPS19BP1	-5.248917201	0.001473474	0.008326298
CD9	-5.251455766	0.021955902	0.066280267
MFN1	-5.252105374	3.27E-06	6.37E-05
ACTG1P10	-5.253458405	0.003611685	0.016609361
RAB43	-5.253787956	8.22E-05	0.000924414
CPSF1P1	-5.253846349	0.001442523	0.008197213
DUSP23	-5.253949162	0.016898242	0.054426748
CGN	-5.25440351	0.015812156	0.051839277
RXRB	-5.254436326	0.005442957	0.022995307
VPS72	-5.255309228	0.004114423	0.018383526
POLR2B	-5.255653107	1.70E-05	0.000262186
B4GALT7	-5.260267742	0.001931967	0.010233932
GCSHP5	-5.260306569	0.014400141	0.04818082
KDM8	-5.260321591	0.01015633	0.03697988
TRMT44	-5.260438322	0.000627283	0.004313998
DCAF10	-5.262156783	0.000980467	0.006084019
PARD6G	-5.262169498	0.015847805	0.051899846
MYSM1	-5.262319327	9.90E-05	0.001053003
NLE1	-5.262622052	8.41E-05	0.000938058
PCYT2	-5.263043938	0.00675398	0.027042729
ARHGAP31	-5.264800955	0.009244524	0.034426698
MID2	-5.265636035	0.001312444	0.007606273
COX15	-5.266767204	0.000180682	0.001664365
CDIP1	-5.266946065	0.004437198	0.019601404
UBE2D4	-5.270138376	0.002611149	0.012919084
KIAA1755	-5.270391408	0.00500406	0.021531867
LARP1B	-5.270407913	0.000155163	0.001490585
ENTPD5	-5.270546412	0.001672901	0.009135508
SERPINB1	-5.270835413	0.00050733	0.00362927
JAM2	-5.270850853	0.004606573	0.02015006
ABHD14B	-5.271636703	0.000298312	0.002444668
UTP4	-5.273225145	0.001830203	0.009776157
CCDC59	-5.273900889	0.015599317	0.051263154
AJUBA	-5.2765146	0.000209942	0.001875379
THUMPD1	-5.27652072	0.000925746	0.005814855
UNG	-5.27676124	0.006081351	0.024918305
CATSPER2	-5.277195214	0.000208828	0.001866804

ABHD17C	-5.277313053	0.031337352	0.086738465
FCGR2C	-5.277569132	0.004528218	0.01986573
TMEM167B	-5.277583077	0.015302355	0.050551228
ITPRIP	-5.27788947	3.71E-06	7.10E-05
NKAPD1	-5.278548844	5.28E-05	0.000652539
SERINC2	-5.280606383	0.000838036	0.005367897
GUSBP2	-5.280755439	0.000190347	0.001734909
FAIM	-5.280877534	0.003482404	0.016162067
SCOC	-5.28277369	0.001256614	0.007375924
MBTPS1	-5.284456319	4.13E-05	0.000536661
DDIT3	-5.284550248	0.00046745	0.003408441
TMED4	-5.28522003	2.97E-07	7.99E-06
KMT5C	-5.285659168	0.000456808	0.003346977
RALGPS2	-5.287768855	0.002450069	0.012300138
PAICS	-5.287994817	3.14E-06	6.15E-05
RHPN2	-5.288825159	0.019849218	0.061472535
MGLL	-5.289176049	9.41E-05	0.00101587
RPL24P8	-5.289973837	0.009843942	0.036172153
DAPL1	-5.290321365	0.030791403	0.085703245
TRNAU1AP	-5.291826601	0.000829154	0.005322208
LRRC37A	-5.292321834	0.006297948	0.02558066
ZIM2	-5.293201208	0.031776859	0.087594421
SRI	-5.293690889	0.000277922	0.002319982
FBXL2	-5.298188976	0.002745458	0.013415836
UBXN11	-5.298644635	7.96E-05	0.00090428
FGFR1OP2	-5.300054161	0.000696561	0.004673703
RNF216P1	-5.300124634	0.001586261	0.008787232
DEDD	-5.301976985	4.07E-05	0.00053046
TAF9B	-5.303870283	0.007056211	0.027941525
SUSD6	-5.305045624	0.00610435	0.024978721
PMS1	-5.305430606	0.00206344	0.010723861
SAMD1	-5.306117359	0.000197873	0.001784691
IL2RG	-5.306249429	0.001974044	0.010417097
SOX5	-5.306926888	0.017698788	0.056345642
SNAP29	-5.307532639	0.002381932	0.012048017
GMPPA	-5.308142937	0.007642681	0.029697274
TGM2	-5.30842741	0.0005233	0.003725895
ARHGEF28	-5.309192065	5.49E-06	0.000100896
GTF2F1	-5.309253962	1.19E-05	0.000195366
PPP4R1L	-5.309452714	0.003534301	0.01634897
WDHD1	-5.310138761	0.013546391	0.046043976
CASZ1	-5.310222256	0.008000393	0.030790701
PABPN1P1	-5.310483246	0.000503729	0.003612046
PA2G4P6	-5.311203756	0.00087118	0.005546958

SLC15A2	-5.311260222	0.007401902	0.02898722
ZNF607	-5.312168016	0.000482409	0.003494365
CHMP4BP1	-5.312512755	0.003436576	0.016014585
METTL2B	-5.312855749	2.92E-05	0.000411979
SLC66A3	-5.313331145	0.009252617	0.034426698
SLC49A4	-5.313750169	8.79E-05	0.000971336
PRDM10	-5.313760626	0.001998923	0.010486583
RSPH4A	-5.314433097	0.015340271	0.050621221
TMEM204	-5.314484997	0.000584667	0.004071176
UBE2V1	-5.317648215	0.000697264	0.004673703
DACT1	-5.317701226	0.000103859	0.001088798
ADHFE1	-5.318253526	0.002219029	0.011395471
ETNK1	-5.31875522	0.001489199	0.008391648
PRSS8	-5.319376073	0.0065374	0.026332342
SLC35C1	-5.319415586	0.006870143	0.02738247
EMC1	-5.319540373	7.59E-05	0.000870441
TM6SF1	-5.320988448	0.005769325	0.023939632
P2RY10	-5.321048356	0.029360069	0.082733676
SLC11A1	-5.321332449	0.005409841	0.022879357
TPGS2	-5.322574184	2.16E-05	0.000321985
ZNF16	-5.322883125	0.001219083	0.00721554
GCA	-5.323116644	0.000493224	0.003555679
ARHGAP42	-5.325946873	0.000149659	0.001453826
RBM38	-5.326747472	0.000924214	0.005811265
MORF4L1P1	-5.327235308	0.005220133	0.02227566
DPH5	-5.327505404	0.014351482	0.048054671
MAP4K1	-5.327629073	0.004939081	0.021335939
SMARCB1	-5.327828013	0.031016562	0.086106528
DENND1C	-5.328689031	0.000289511	0.00239524
COA1	-5.3288918	0.000224659	0.001977655
RTCB	-5.329321356	0.003009211	0.014453764
GTF3C6	-5.32933799	0.00809821	0.031068379
ADA	-5.329666284	0.013110546	0.045071215
QDPR	-5.330232081	0.001300853	0.007566323
PHKA2	-5.332508954	0.000370647	0.002848969
HAUS6	-5.332512493	0.007863966	0.030391264
PTS	-5.33489716	0.001746374	0.009436654
IGHV4-61	-5.335714152	0.00832491	0.031756922
ZNF674	-5.335961907	0.000133663	0.001329377
BOD1	-5.336214271	0.002695993	0.013216809
GBF1	-5.337762229	0.001993941	0.010478705
DDX39A	-5.337797193	0.000254977	0.002174108
BRMS1L	-5.337868435	0.000950359	0.005938642
GFRA2	-5.338049262	0.002284444	0.011632695

BCLAF3	-5.341654143	0.000560507	0.003935256
KBTBD11-OT1	-5.34231362	0.007263598	0.028597607
CASTOR2	-5.34311321	0.027475104	0.078861433
ZKSCAN2	-5.34416057	0.000729829	0.004840467
MRPL18	-5.344937364	0.014318246	0.047967908
SHQ1	-5.347160438	8.95E-05	0.000983103
FANK1	-5.348388203	0.001309376	0.007597643
U2AF1L4	-5.348903028	6.06E-05	0.000732619
AGK	-5.349534814	0.013508285	0.045966059
SLC43A2	-5.349854079	8.37E-05	0.000934989
ENSG00000284292	-5.350752949	0.00725823	0.028597607
WASHC3	-5.350853601	0.000404351	0.003049384
ENSG00000275495	-5.350913932	0.006939999	0.027623067
ZSCAN32	-5.350994332	0.001511417	0.008475041
ANKDD1A	-5.351603298	0.008935823	0.033506567
HSPB7	-5.351965846	0.001239333	0.007303258
AGPAT1	-5.352071386	0.000254731	0.002174108
AGPAT1	-5.352071386	0.000254731	0.002174108
TAF8	-5.352212143	0.00160168	0.008836249
ANO9	-5.353540487	0.000981666	0.006084019
PTPN22	-5.353619198	0.029527829	0.08309808
C1GALT1	-5.354145514	0.014835309	0.049372171
TPST1	-5.354525634	0.007170629	0.028326794
RCL1	-5.356479537	0.002368436	0.011989774
FBXO9	-5.357977602	2.67E-07	7.30E-06
PARP2	-5.35798964	0.013241069	0.045338018
RPS7P11	-5.358342311	0.002197204	0.011312191
ARHGAP10	-5.35959358	0.001434923	0.008173323
RBIS	-5.359680357	0.002907275	0.014044409
IFT74	-5.360258	0.000805738	0.005218112
THAP9	-5.360342431	0.000434097	0.003217578
IQCK	-5.360918344	6.08E-05	0.000734593
AFMID	-5.362431089	5.49E-06	0.000100896
UFM1	-5.362540362	0.000567586	0.00397573
DNASE1	-5.366161754	0.000163529	0.001542754
CEP170P1	-5.3665607	0.003804162	0.017284377
TSPAN5	-5.366615156	0.002380967	0.012048017
PSMB2	-5.367423965	0.007407102	0.028995976
SMURF1	-5.367900703	0.005789224	0.024008324
GHR	-5.370023619	0.004489118	0.019765838
SNAPIN	-5.371579798	0.003177335	0.015052235
CDH16	-5.372020485	0.007275177	0.028618375
CSKMT	-5.372382396	0.000648535	0.004419968
ANKEF1	-5.37262656	0.002047276	0.010662748

IFNAR2	-5.37371531	0.000155649	0.001494035
NAPEPLD	-5.373860021	0.000395625	0.002992903
NID2	-5.374145886	0.000451135	0.003311422
OR2A7	-5.374348542	0.002491417	0.012468412
NABP2	-5.37527086	0.001312748	0.007606273
CWF19L1	-5.3763322	3.41E-05	0.000464755
MRPL45	-5.376912437	0.006887886	0.027442718
EIF4A3	-5.378763792	0.003115829	0.01481886
CTU2	-5.379744091	0.000406082	0.003058628
CXCL12	-5.381833722	0.003095451	0.01475092
PUS3	-5.382664657	0.002534817	0.012610534
PMS2P7	-5.383216958	0.028038077	0.080021901
LTO1	-5.383769283	3.27E-05	0.000447521
SPTLC2	-5.384270791	7.81E-05	0.000891369
RAB10	-5.38510417	4.73E-05	0.000598711
KIF21B	-5.385512123	0.005721244	0.023764564
APOBEC3B	-5.385619638	0.026450475	0.076492189
ENSG00000271793	-5.385925236	0.013620538	0.04618011
RBMS1P1	-5.385986821	0.011738851	0.04134043
TTC8	-5.38713187	0.000339564	0.002687386
FTH1P23	-5.387474648	3.24E-05	0.000444277
POM121L9P	-5.38821091	0.003912125	0.017681986
IFITM1	-5.388331887	0.003002627	0.014445073
CACNB1	-5.389168415	7.47E-07	1.80E-05
ACRBP	-5.390121547	0.002160729	0.011148109
IDE	-5.390229988	7.82E-06	0.00013657
ECSIT	-5.390371539	0.000554155	0.003899706
PDIA3P1	-5.391732268	0.000312604	0.002539437
CTNS	-5.396532052	0.02889036	0.081760327
XPC	-5.396657321	5.87E-05	0.000716198
GOPC	-5.396874176	0.014855568	0.04941242
TRAF6	-5.39689907	0.005298268	0.022528397
KIAA0895	-5.397609641	0.001617579	0.008883476
MED8	-5.39773603	0.006296994	0.02558066
ENSG00000276393	-5.400063897	0.003335408	0.015635856
PLEK2	-5.401744915	0.001561458	0.008689603
ARHGAP19	-5.401842976	0.001022454	0.006274295
KLHL15	-5.4038828	0.000156536	0.001498363
ENSG00000270149	-5.404164775	0.014645722	0.048862132
GOLGA8J	-5.404481446	0.008674734	0.032679421
ZBED8	-5.404543133	0.003141242	0.014916279
TDRD10	-5.405227509	0.000747563	0.004927238
PARVG	-5.405685791	0.003107865	0.014798426
FILIP1	-5.406004444	0.000119086	0.001216386

MAP1LC3A	-5.408214454	0.006450496	0.026069049
TCFL5	-5.40941169	0.001436552	0.008178753
RAB3GAP1	-5.410551813	1.95E-05	0.000296871
HDDC2	-5.411067856	1.36E-05	0.000218869
RPSAP6	-5.412100324	0.00176698	0.00951826
ING5	-5.412625828	0.00050254	0.003607797
PFAS	-5.416010741	0.000763829	0.00500562
ENSG00000253882	-5.416333063	0.020101144	0.061940999
ZNF585A	-5.416565116	0.000586314	0.004078589
BTRC	-5.416908374	0.000220704	0.001949927
ARID3A	-5.417699812	0.00302007	0.014488677
UXS1	-5.418135384	0.000352854	0.002757229
IGLV3-19	-5.41933671	0.028139448	0.080235542
MANF	-5.419894978	0.001620043	0.00889297
CRISPLD1	-5.422340173	0.013753106	0.04655134
ENSG00000276005	-5.423246511	0.0127539	0.044051076
MAP2K3	-5.423421159	0.00013975	0.001378942
AR	-5.424343638	0.000692594	0.004659901
FAM219B	-5.425957482	0.000147256	0.00143611
PEPD	-5.426004676	0.00108423	0.006565042
ZNF48	-5.427383676	0.001180101	0.007029502
FBXW11	-5.427693012	0.000130518	0.001309946
ZSCAN25	-5.432659196	0.000292882	0.002413239
RBBP4P1	-5.434273398	0.007695893	0.029866313
PLAGL1	-5.435574512	0.002514032	0.012548389
PRKAR2A	-5.436130137	7.72E-05	0.000882752
TM2D3	-5.440676518	2.92E-05	0.000411979
DHPS	-5.440751733	0.000668689	0.004529271
CPEB2	-5.440839307	0.000592566	0.004117349
PDZK1P1	-5.443291319	0.026706833	0.077095041
ZNF343	-5.445272935	1.23E-06	2.74E-05
MRTO4	-5.447806498	0.025637096	0.074576161
FER1L6	-5.447833526	0.01522071	0.050391541
SELPLG	-5.447900272	0.007698521	0.029866313
ARHGEF10	-5.448024668	0.000139785	0.001378942
NUDT16L1	-5.448482712	0.00214625	0.011082853
GBP3	-5.449178335	9.75E-05	0.001041603
TPRA1	-5.449397731	0.00154138	0.008605565
DDX60L	-5.449632355	8.89E-05	0.000976857
NEXN	-5.451573195	0.000127595	0.001288079
CTAGE7P	-5.452778841	0.005482441	0.023105664
PRICKLE2	-5.454244324	6.49E-06	0.000116364
MYO7A	-5.455525093	0.000988225	0.006111755
FMO4	-5.456098634	0.007249588	0.028597607

HIRA	-5.456330491	2.73E-05	0.000392531
THBS2	-5.457064276	0.006009897	0.024714715
ZNF641	-5.457880661	0.00157923	0.008764326
AKAP7	-5.458557793	0.003345977	0.015679327
IGHG3	-5.459162968	0.000243782	0.002109175
RELT	-5.459355726	0.000802595	0.005206115
ZNF658	-5.459506724	0.016869414	0.05436299
CLOCK	-5.459574173	0.000265148	0.002244302
USP19	-5.460539907	0.002738974	0.013389559
KANK1	-5.461877488	8.65E-05	0.000958062
PRICKLE4	-5.461976446	0.001474363	0.008327436
KIAA1958	-5.46321149	2.58E-06	5.19E-05
BICDL2	-5.464440305	0.000745787	0.00491935
SUPT16HP1	-5.464669497	0.000510502	0.003647645
GPAM	-5.465027551	0.000117328	0.00120249
SH3BGRL	-5.465194284	0.007995153	0.030780321
PCDHGA10	-5.465310647	0.007917343	0.030568266
PUS10	-5.466550914	0.001779005	0.009566005
FAM76A	-5.467361463	0.000709276	0.004735288
PPP2R3A	-5.468246523	0.003708386	0.01698309
MSL3	-5.468327867	0.000818708	0.005274307
ERLIN1	-5.468577049	0.000574851	0.004017316
LRRC34	-5.469372646	0.007794526	0.030195306
INSIG1	-5.470667293	0.002846177	0.013818275
TMEM39B	-5.47127244	0.000131805	0.001318484
MCRIP1	-5.471404279	0.000148247	0.001441272
CDH26	-5.472283609	0.02625409	0.076047984
IGFBP2	-5.473657139	0.000601757	0.004171629
VRK2	-5.474635796	5.02E-05	0.000625043
FLAD1	-5.474935374	0.00011648	0.001194808
ELOF1	-5.475331458	0.003082369	0.014700155
C8orf44-SGK3	-5.475529282	0.012117514	0.042314118
TMEM25	-5.476215879	0.00063467	0.004352433
ATXN3	-5.476793334	0.000348204	0.002731472
BRSK1	-5.477108182	0.002414616	0.012147343
ARPIN-AP3S2	-5.478386103	0.000420785	0.003145881
ABHD16A	-5.478694447	0.004963659	0.021418942
GALNT12	-5.478712544	0.003201757	0.015156071
ENSG00000270099	-5.481268476	0.015259099	0.050463423
SEPTIN7P2	-5.482508863	0.000393785	0.002980847
MDH2	-5.482557326	0.000329701	0.002637776
CFAP97	-5.482596281	0.002507667	0.012526949
KLHL28	-5.484289471	0.000120668	0.001230473
SOCS2	-5.484314658	0.002338765	0.01185941

SYBU	-5.485988394	0.003218589	0.015206009
DHDDS	-5.48675937	0.000229938	0.002015325
CPSF4	-5.487138164	0.000776377	0.005079603
PGGT1B	-5.487254326	0.000347929	0.002731085
NFATC1	-5.489012834	0.000160485	0.001524042
C9orf72	-5.489158545	0.002485902	0.012454206
SLC4A7	-5.489841628	1.96E-05	0.000297347
ZNF681	-5.490246786	0.000102643	0.001079403
PSMC1P1	-5.490865796	0.002721581	0.013320668
GSS	-5.492618725	0.011514499	0.040739987
TNK1	-5.494153951	0.013323522	0.04550449
ARNTL2	-5.495567973	0.002872909	0.013915044
RGPD3	-5.496822058	0.000340209	0.002687917
EDF1	-5.498147868	0.003011434	0.014458707
TRMT12	-5.498192045	0.002620035	0.012933483
NOC2L	-5.498234295	0.002100868	0.010890323
IPCEF1	-5.498241046	9.56E-05	0.001026303
ZNF530	-5.498751817	0.004432027	0.019585708
TTF2	-5.499060377	0.003294112	0.015478234
SMTN	-5.499433825	7.55E-07	1.81E-05
TANK	-5.499519762	2.83E-05	0.000402922
LPCAT1	-5.500617357	0.001616959	0.008883476
DONSON	-5.500694713	0.000449922	0.003308543
KICS2	-5.501190525	0.00640497	0.025928346
TCAF1	-5.501621324	0.000286753	0.002377304
LCP1	-5.501693926	0.001549549	0.008635238
LIMS3	-5.50261843	0.013174953	0.045239612
ADGRE2	-5.502751754	0.003311985	0.015538086
TMEM205	-5.502788785	3.32E-05	0.0004534
TRIM36	-5.503469321	0.000487574	0.003523338
PANK4	-5.50485098	0.000444496	0.003282599
PRR5L	-5.506366395	0.009489388	0.035114508
PITHD1	-5.506564854	0.007456626	0.02915213
NOSTRIN	-5.508128474	0.008171316	0.031299244
GOLGA6L10	-5.509184267	0.000923907	0.005811265
USP46	-5.510681249	0.001278571	0.007465421
HFM1	-5.510747249	0.002402355	0.012115876
WASF3	-5.511512558	0.025310883	0.073820468
C1QTNF1	-5.512246828	0.0011714	0.006991435
OLFM2	-5.512465072	0.001406372	0.008056186
MCTP1	-5.512559203	0.001246018	0.007333985
NECAP2	-5.512977015	0.000188109	0.001718213
CCDC43	-5.514031843	0.003111242	0.014805816
PDZD8	-5.514191198	0.004358703	0.019339347

BTBD8	-5.516196071	0.013269879	0.045410975
CASTOR3	-5.51700172	0.000552347	0.003893774
CBX3	-5.517123951	0.000160413	0.001524042
RAB31	-5.517319409	0.000260913	0.002217764
EPSTI1	-5.51787937	0.007704536	0.029880073
AP1S2	-5.518015569	0.005790582	0.024008324
ATPAF1	-5.519159692	2.90E-05	0.000411097
PIP4P1	-5.519297429	0.001786152	0.009593938
HLA-DMA	-5.519450964	0.000269453	0.002268052
RAB9A	-5.521859734	0.004495995	0.019776246
SALL1	-5.523066854	0.000449798	0.003308543
ZBTB34	-5.524454409	0.000481033	0.003486482
ZNF209P	-5.524683031	0.001720569	0.009318018
TMEM134	-5.524801945	3.89E-05	0.000512103
CHCHD7	-5.524847143	2.83E-05	0.000402922
PGAP6	-5.526683437	3.17E-05	0.000438339
GSTO1	-5.527009396	0.001935182	0.01023752
SKIDA1	-5.527537817	0.00166654	0.009106907
MAFK	-5.527649879	0.00263708	0.013007014
SMN2	-5.529452426	0.000606086	0.004194432
HAUS1	-5.530030874	0.005597841	0.023420762
MBD3	-5.530557135	6.60E-05	0.000785307
BNIPL	-5.530657136	0.000312226	0.002538288
ASPN	-5.533492063	0.001703963	0.009249341
MBNL3	-5.533824429	0.000799182	0.005192326
SEMA4F	-5.534663297	1.30E-05	0.000211321
UPK3B	-5.535686667	0.001410985	0.008063531
MYH11	-5.537692593	0.002404557	0.012121931
MRPL33	-5.537732429	0.002799825	0.013653942
ITGBL1	-5.538935863	0.01285792	0.044352314
POU5F1P4	-5.54014634	0.005496481	0.023148714
QTRT2	-5.540850628	8.69E-06	0.00014977
ZNF675	-5.542518479	0.000244119	0.002109676
MAP2K5	-5.542692752	0.001067927	0.006492311
RASSF5	-5.543321543	0.000101777	0.001073091
MTERF1	-5.543356892	0.000239013	0.002075062
NOTCH4	-5.543996862	0.017141363	0.054924826
H2AX	-5.545498666	0.00584077	0.024145039
VCAM1	-5.547212954	0.007181988	0.028362408
VWA5A	-5.549409884	3.57E-05	0.00048057
CRAMP1	-5.549761208	8.10E-05	0.000914009
RIMBP2	-5.551550775	0.007202417	0.028424536
KCTD13	-5.552402266	0.000127957	0.001290659
OR2A14	-5.552440041	0.002242006	0.011479374

MRPS21	-5.55254306	0.02313847	0.069033286
ZDHHC18	-5.553054452	0.000137526	0.001363325
TMEM232	-5.554546495	0.005018949	0.021572927
WARS2	-5.555556974	0.01193064	0.041853024
ELP6	-5.557616755	4.18E-05	0.000541911
DTX3L	-5.55804611	0.000434054	0.003217578
STAT5A	-5.558683958	6.42E-05	0.000765858
AIF1L	-5.561573214	0.00109899	0.006627889
JMJD7	-5.562457103	0.000957209	0.005975272
LSM12P1	-5.563569623	0.000339886	0.002687386
TMED5	-5.563782635	1.00E-04	0.001058744
ARPP19	-5.563934396	0.001191311	0.007085814
EIF4E3	-5.564302308	3.15E-05	0.000438182
POU2F3	-5.56460695	0.004774986	0.020737866
PLPP1	-5.564988935	0.003468453	0.016109804
ARMC9	-5.566779833	0.003079943	0.014695035
PMS2CL	-5.568345114	0.001042044	0.006363724
ATG12	-5.568376301	0.000417039	0.003121732
CD200	-5.568704276	7.30E-06	0.000129047
WDR37	-5.569915846	0.001989767	0.010462266
IGDCC4	-5.57063767	8.40E-05	0.000937759
ENSG00000235036	-5.570760279	0.011982434	0.041978403
GJA4	-5.571297585	0.000336596	0.002675089
ITPA	-5.57136393	0.00014296	0.001402263
KIF21A	-5.572036009	6.86E-06	0.000122228
ENSG00000258150	-5.572345582	0.005306859	0.022538322
ZNF18	-5.572827331	0.001588872	0.008793645
STK16	-5.573070741	0.000254626	0.002174108
SGTA	-5.573443816	0.002257161	0.011517992
ANP32AP1	-5.575573899	0.013538107	0.046029562
CD320	-5.577259055	0.00199959	0.010486583
LY75-CD302	-5.578290214	3.48E-05	0.000472038
LYZ	-5.578655512	0.003794711	0.017247909
NUDT9	-5.579203211	0.006659969	0.026745939
RNF24	-5.579535356	0.000138487	0.001371722
APOBEC3F	-5.580329231	0.012219189	0.042598231
SRR	-5.580980053	0.0049909	0.021482879
ZNF100	-5.58514542	0.00074562	0.00491935
ZNF461	-5.58633054	0.000158992	0.001516497
ACTR6	-5.587106975	0.000127246	0.001285634
ATAD5	-5.587174987	0.006162413	0.025144949
SIAH1	-5.588137093	0.001162985	0.006958362
ACBD6	-5.589037425	0.001789144	0.009603459
TFF3	-5.589614056	0.001780592	0.009570286

ARHGAP36	-5.590600123	0.002140075	0.011055685
USP39	-5.591098742	0.000275507	0.002302994
DYRK2	-5.595319311	0.001975607	0.010417097
THOC7	-5.596915773	0.003719118	0.017025798
MICU1	-5.597953412	6.70E-06	0.000119877
GIPR	-5.598967417	0.003159984	0.014981767
LRRC42	-5.599867241	0.003561292	0.01644978
NSMCE3	-5.600066617	0.001817532	0.009720687
AGA	-5.600583996	0.010066486	0.036766669
GSK3A	-5.600995319	1.24E-05	0.000203703
NSL1	-5.60132154	0.005710912	0.023746094
EIF4E	-5.602127157	5.58E-08	1.73E-06
CINP	-5.604123465	0.001343962	0.007757422
TMEM129	-5.605314054	1.73E-05	0.000266063
ANO10	-5.606215562	8.15E-05	0.000918352
MLF1	-5.607042302	0.006779943	0.027119773
TOP3B	-5.607602082	0.001545018	0.008617923
HSPA1B	-5.607954747	0.006783729	0.027125952
SIGMAR1	-5.609670285	0.007431273	0.029062402
COLQ	-5.611376278	0.002318195	0.011784725
ENSG00000267952	-5.611548517	0.00300577	0.014446959
CFB	-5.613156501	0.010093224	0.036812797
TMEM258	-5.613202727	0.000666053	0.004516473
SNIP1	-5.617102641	0.000489996	0.003536617
ZNF605	-5.617730889	1.25E-05	0.000204927
EYA2	-5.617735809	0.000322251	0.002598617
ITGA2B	-5.617813308	0.005856627	0.024202325
SLU7	-5.620588213	0.000357999	0.002782007
GMFG	-5.622423909	0.005064838	0.021731582
RIMS2	-5.623376149	1.48E-05	0.000233461
RAF1	-5.62350949	0.002673412	0.013122053
OSTC	-5.623560187	0.009765979	0.035940229
SAAL1	-5.625507497	0.002235754	0.011466766
CYP2D6	-5.625636346	0.001040432	0.006357089
POP4	-5.626785025	0.001315237	0.007617048
MSMO1	-5.627330299	0.000398214	0.003010608
TTC38	-5.627917638	0.000353126	0.002757314
CYP2U1	-5.629722408	0.005905256	0.024370004
THRA	-5.630535614	3.45E-05	0.000468057
PGPEP1	-5.631062171	0.001038331	0.006350661
VPS33A	-5.63130661	0.011002451	0.039272683
TGFBR3	-5.63312392	0.000142871	0.001402263
ZNF880	-5.633359559	0.005104003	0.021853148
MLXIPL	-5.634174744	3.67E-05	0.000488599

LAPTM5	-5.634519157	0.000503432	0.003612046
MRPL46	-5.634564811	0.006774457	0.027106786
HSD17B8	-5.635267051	0.000450892	0.003311422
SERPINH1	-5.636055975	0.000427902	0.003189242
SREBF2	-5.636262318	0.000114459	0.001176828
SNRPB2	-5.638180335	0.000345867	0.002725502
UBAC1	-5.640251036	1.92E-05	0.00029291
NPR2	-5.641085351	0.007924405	0.030585778
KATNAL1	-5.641291173	0.002338007	0.01185941
TM9SF4	-5.641452051	0.004601015	0.020134016
PDE1B	-5.642806581	0.013199271	0.04525743
ATP2C2	-5.644419569	0.004750882	0.020662839
PRELID3B	-5.645212121	0.000986237	0.006102972
NANOGP4	-5.645649015	0.002429629	0.01220765
LY6E	-5.648527583	0.002801226	0.013655273
KAT6A	-5.651803171	1.00E-05	0.000168133
PCDHB3	-5.652544256	0.013205236	0.04525743
ADAMDEC1	-5.652973865	0.034389061	0.092724637
HLA-F	-5.655538615	0.001449177	0.008218422
CDC42SE1	-5.656847535	1.28E-05	0.000209211
ZNF398	-5.659475082	0.000325822	0.002622173
IMP4	-5.661320768	0.00202424	0.010570061
CEP57L1	-5.662636126	0.000930465	0.005838437
PIGV	-5.662770633	0.031259726	0.086602821
CERS6	-5.664099535	0.002269038	0.011563972
YIF1A	-5.665012578	2.52E-05	0.000367646
PPCDC	-5.667690959	0.004197275	0.018726064
ISY1	-5.66903281	5.61E-05	0.000689681
ZNF121	-5.669629478	0.000327799	0.00262865
LIAS	-5.670039841	0.000113612	0.001169353
GNG5	-5.670624106	0.020953652	0.064030045
LEF1	-5.671456779	0.000749403	0.004929768
MRPL21	-5.672353654	0.006517713	0.026270529
FN3K	-5.672448456	3.51E-05	0.000474745
SESTD1	-5.673954085	0.000974335	0.006060304
OSBP2	-5.674310315	0.003437367	0.016014585
PLEKHA8	-5.675102172	0.000358094	0.002782007
ECHDC3	-5.675287005	0.003989672	0.017989865
ATP2A1	-5.675485699	0.005287939	0.02249744
NUDT4P2	-5.676201747	0.000717801	0.004776396
AGFG2	-5.676675587	0.011723432	0.041298143
PRCP	-5.676751708	0.000371185	0.002848969
ATF7IP2	-5.678525907	0.000171582	0.001601252
TRIM23	-5.679008674	6.88E-05	0.000808191

CELF6	-5.679379207	0.003965382	0.017895969
SLC22A31	-5.680060931	0.00252232	0.01256947
TNFAIP8	-5.680407376	0.001018962	0.006257494
NUTM2G	-5.681209246	0.002974426	0.014320783
PLCH2	-5.68364049	0.004069708	0.018241795
BTN2A2	-5.684938683	0.001760176	0.009490054
LDAF1	-5.687654336	0.020867834	0.063832264
MAPK13	-5.687974706	0.000163474	0.001542754
FTH1P7	-5.68850003	0.000382729	0.002922774
RECQL	-5.688527459	0.000175466	0.001627721
PCDHAC1	-5.691974557	0.000354632	0.002763982
ELAPOR1	-5.693762146	1.13E-06	2.55E-05
CRADD	-5.693855639	0.000285937	0.002373788
CCDC18	-5.697954571	0.004189937	0.018700222
PLCG2	-5.698086897	9.90E-05	0.001053003
WDR75	-5.698558777	0.000965413	0.006014078
PDE6B	-5.699954073	0.001240558	0.007306914
ABCF2-H2BE1	-5.701479067	0.000272344	0.002287287
DNAJA4	-5.702485841	0.000315266	0.002557598
IKZF5	-5.70439178	9.60E-05	0.001030365
SH2D4A	-5.705336522	0.000819486	0.005276096
IL1RL1	-5.705417435	0.005476885	0.023097508
ESF1	-5.706761632	0.005705947	0.023743574
LRRC37BP1	-5.707093111	0.000148079	0.001440799
PIGB	-5.707571343	9.38E-05	0.00101587
GNAO1	-5.707770587	6.62E-05	0.000786742
STMN3	-5.707819894	0.001582941	0.008776877
ZNF317	-5.709614182	0.00015432	0.001486135
NDUFS2	-5.710382147	0.000469243	0.003417396
SARS2	-5.710950595	0.000382247	0.00292275
HLA-F	-5.711605889	0.000161636	0.001532058
TRAPPC4	-5.712424454	5.63E-06	0.000102992
SSX2IP	-5.712511468	0.000765409	0.005013263
PDCD10	-5.712569412	8.76E-06	0.000150855
UBE2B	-5.713072293	0.000251268	0.00215145
ATRNL1	-5.713504929	0.003368708	0.015761439
WDR77	-5.713609447	0.000493999	0.003559148
BRMS1	-5.715790698	7.94E-05	0.00090428
MED28	-5.716579335	0.001089767	0.006588681
ETNK2	-5.716679144	0.000384002	0.00293062
DLG2	-5.718126484	0.000150308	0.001458963
TMEM9B	-5.719267948	0.000335703	0.002673256
ENSG00000264187	-5.71937795	0.000799128	0.005192326
ABCA12	-5.719554046	0.010589317	0.038124059

FER1L4	-5.721927059	9.73E-05	0.001040309
PPIL6	-5.722445594	4.80E-06	8.97E-05
RELL1	-5.723622435	0.001987127	0.010452925
FAM86EP	-5.72487705	0.005464317	0.023077491
ALG12	-5.727529328	0.004411094	0.019507445
MPC2	-5.728293853	0.005795528	0.024015431
DBF4	-5.729262003	0.001739182	0.009401991
UBXN2A	-5.72935795	0.000644278	0.004403351
TIRAP	-5.729390215	0.000718942	0.004781362
AQP4	-5.729577886	0.02043522	0.062794594
MSH5	-5.733153482	0.002619048	0.012933483
AMPD2	-5.733158598	0.000196907	0.001778626
GAS7	-5.733266915	4.71E-05	0.00059682
ZBTB7C	-5.73329162	2.79E-05	0.000398257
ENSG00000255508	-5.734583731	0.001007949	0.006202445
ENSG00000262302	-5.735498446	0.019365339	0.060334123
ENSG00000229180	-5.738467713	4.30E-08	1.36E-06
SNRNP40	-5.740664024	0.000334889	0.002670291
DKK3	-5.741654176	0.000225559	0.001984133
CRYGN	-5.743451525	0.0015431	0.008611196
ASAH2	-5.743535707	0.008689535	0.032714817
CLN6	-5.74402619	3.51E-05	0.000475231
IQCG	-5.744276528	0.002136501	0.011041933
SYNPO	-5.744326106	5.24E-06	9.72E-05
SUPT3H	-5.744461358	0.000207389	0.001856688
PLCD4	-5.744574221	0.001298888	0.00756111
GNRHR2	-5.745891793	0.000868627	0.005534148
CCDC91	-5.746251015	6.82E-05	0.000804835
HNRNPCP7	-5.746968154	0.000403637	0.003047693
GOLT1B	-5.747914661	0.0053121	0.022552668
ESR1	-5.749049228	0.00488229	0.021150766
SPINDOC	-5.749370826	0.001273923	0.007445466
PDE7B	-5.749380837	0.001481925	0.008354552
ALAD	-5.74955003	0.001409897	0.008063531
RUBCNL	-5.750843859	0.003738979	0.01706485
TMEM106A	-5.751014987	5.94E-05	0.000721565
HADH	-5.751425802	0.005376168	0.022776736
HOMEZ	-5.751883954	6.37E-05	0.000761617
PPP2CB	-5.752061791	2.57E-05	0.000372677
PPP3CA	-5.753688823	3.59E-05	0.000482658
FN3KRP	-5.753940912	0.003728634	0.017042129
IGKV2D-29	-5.754209726	0.019525927	0.060678261
PNPLA4	-5.754906494	0.001717851	0.009307462
TP53INP1	-5.756288748	4.30E-06	8.11E-05

PCDHA10	-5.757318414	0.00094423	0.005906441
ARL16	-5.757393247	6.15E-05	0.000740657
ZNF600	-5.757552459	0.000336218	0.002675089
SCAPER	-5.757650314	5.39E-06	9.94E-05
CEP290	-5.75903998	0.019966621	0.06168521
MPZL1	-5.759416996	0.00043155	0.003206431
VWDE	-5.760285513	7.09E-05	0.000826794
ENSG00000268400	-5.76113752	0.004269537	0.019006429
DUSP10	-5.761626521	0.004922073	0.021285018
EML5	-5.761637605	0.000202391	0.001818666
JAML	-5.762021027	0.019115685	0.059681755
PDLIM4	-5.76214719	9.61E-06	0.000162279
HSPA9	-5.76327645	3.24E-05	0.000444277
ALDH3B1	-5.763544923	0.001198163	0.007116079
ENSG00000254509	-5.76420905	0.001093945	0.006604047
TENT4B	-5.764264993	0.003155395	0.014971353
SELENOI	-5.764412249	0.003292728	0.015477739
GCOM1	-5.765264866	0.004762381	0.020690547
ZP3	-5.767006851	0.002240244	0.011475209
PEG10	-5.767179633	0.001442089	0.008197213
METTL5	-5.768178878	0.001187811	0.007071944
TMEM184C	-5.770263967	0.000152015	0.001471893
CSNK1G2	-5.773291198	1.36E-06	2.97E-05
FAM13C	-5.773303895	0.000810325	0.005236611
RABEP2	-5.773687025	0.000747797	0.004927238
AOX1	-5.77540175	0.008696518	0.032730924
DCAF12	-5.777617038	0.000464541	0.003397468
ARMH3	-5.778233676	0.009271581	0.03446659
C2orf88	-5.778298351	0.002056483	0.010701494
ZFHX4	-5.778634478	0.001907524	0.010138374
CAB39L	-5.779441992	0.001457475	0.008247444
LSP1	-5.782584984	0.002186938	0.011268924
MTMR7	-5.782928678	0.003998078	0.017989865
SLC9A3R1	-5.783152278	0.0015117	0.008475041
ENO2	-5.785653404	2.00E-05	0.000301839
TMEM170A	-5.787455396	3.04E-05	0.000425693
TLK2P2	-5.787568472	4.12E-07	1.07E-05
ZNF473	-5.787873581	0.000663295	0.004502644
SLC9A7	-5.788523312	8.02E-05	0.000908315
PPT1	-5.789005184	0.008561996	0.032395875
YPEL3	-5.789487684	8.55E-05	0.000949284
SYS1	-5.79045043	0.001080719	0.006547059
SLC25A14	-5.79631356	0.001415493	0.008077851
ARPC4-TTLL3	-5.797114102	0.004444521	0.019619431

CEP164P1	-5.799985072	0.003208148	0.015180384
OLFM1	-5.800743009	0.010327507	0.037415535
FAM90A25P	-5.800763455	0.002135076	0.011041933
COPS3	-5.800841565	0.000399862	0.00302118
LGI3	-5.800942853	0.001231139	0.007269127
RBM27	-5.801034829	0.00860817	0.032513805
PLA2G4B	-5.802308505	0.000574459	0.004016898
TAMM41	-5.802877835	0.000189248	0.001726195
ATP1A2	-5.803770649	0.008422114	0.032016731
PTBP3	-5.804196022	8.26E-08	2.45E-06
PSMD3	-5.807263989	1.88E-05	0.000286903
GFER	-5.8074481	0.000424396	0.003167012
RNF103	-5.807597715	0.00078693	0.005137541
DZIP1	-5.807802015	0.001614361	0.008880007
PPA1	-5.808342884	6.10E-05	0.000735842
UGGT2	-5.808548275	1.16E-06	2.62E-05
STN1	-5.808752143	0.000977197	0.006071863
PGM1	-5.809659743	0.000175707	0.001627721
TREX1	-5.809799171	0.018497691	0.058321452
EHMT2	-5.811268872	0.002389788	0.012077658
STRC	-5.811405389	0.001212418	0.007179604
SLC35B4	-5.813383179	0.003354522	0.015707207
MRPS33	-5.814686877	0.000163063	0.001541967
TRDC	-5.814762547	0.00349531	0.016203458
TTBK2	-5.814889767	0.001666232	0.009106907
STC1	-5.815221562	0.000191091	0.001739068
SWT1	-5.81539607	0.003220034	0.015206902
CCND3	-5.815442856	2.07E-05	0.000310908
DPAGT1	-5.815508144	8.32E-05	0.00093206
BABAM2	-5.815607676	0.000154949	0.001490585
ZNF124	-5.81574277	6.03E-05	0.000730367
USP20	-5.820807147	2.00E-05	0.000301839
SFMBT2	-5.821504496	7.34E-05	0.000851051
ABI1	-5.82217425	0.000756365	0.004970165
ZNF385A	-5.822423581	1.15E-05	0.000189135
SLC40A1	-5.823561285	0.000662875	0.004502494
NMD3	-5.824311758	0.000489282	0.00353357
POLR1G	-5.825627836	0.001493462	0.008411753
DHRS4	-5.825951874	0.010184132	0.037018869
TUFT1	-5.827596959	0.000878373	0.005579402
ZC3HC1	-5.828864947	0.000284687	0.002365029
GPX8	-5.829020585	0.005137313	0.021980219
TMOD1	-5.829530881	0.00825225	0.031549348
MOB1B	-5.829565397	0.000558422	0.003925167

CNIH1	-5.83159474	0.000804541	0.005213151
ZBTB41	-5.831838088	1.31E-05	0.000213167
XPA	-5.832235661	8.06E-05	0.000911037
ENSG00000285155	-5.832494389	0.005960598	0.024556529
IGLV3-1	-5.834211498	0.009473307	0.035076449
MGST1	-5.835880959	5.68E-06	0.000103617
PLCB1	-5.837046013	9.42E-05	0.00101587
ATP8B1	-5.837862486	0.001983378	0.010443554
FCGBP	-5.839278803	0.008609282	0.032513805
RAVER1	-5.840057482	4.11E-08	1.31E-06
IGLC2	-5.840845768	0.000370106	0.002847908
RNF34	-5.841615497	0.000534899	0.003799004
ENSG00000276805	-5.84161919	8.95E-07	2.07E-05
DGCR6	-5.841732696	0.00491872	0.021278122
MRPS35	-5.841993143	0.00063806	0.004371298
CR1	-5.842315626	0.009154004	0.03413434
ASCC1	-5.842933814	8.87E-05	0.000975603
CLCN4	-5.844533581	0.000745335	0.00491935
ZNF862	-5.847548743	0.003181159	0.015064456
RYKP1	-5.849242654	2.29E-06	4.66E-05
SLC2A1	-5.850308548	3.63E-05	0.000486148
ADSS2	-5.850561863	0.000207579	0.001857013
COQ9	-5.851535598	0.000270937	0.002277374
PFDN1	-5.851860694	2.57E-05	0.000372677
MRPS11	-5.853350203	0.002903256	0.01403395
KCNMB3	-5.853445002	0.003741747	0.01706485
AVIL	-5.853497619	0.000638143	0.004371298
BEX2	-5.85380163	0.003004082	0.014446329
CPED1	-5.854932296	0.004427893	0.019574588
PRPF31	-5.855555503	0.047045993	0.116977135
TBC1D3B	-5.859227431	0.000214534	0.00190655
IL17RE	-5.860488548	0.000420411	0.003145028
ZNF85	-5.861231768	0.003501543	0.016226141
PDXDC2P	-5.861992957	0.000247358	0.002130973
FGD2	-5.862519243	0.000250399	0.002146482
BOLA2B	-5.865562486	0.007847077	0.030345374
PIH1D1	-5.868027469	0.000176397	0.00163235
CDC40	-5.86821443	0.000732011	0.004849623
CCL3	-5.869082669	0.009019651	0.033742516
SNX12	-5.872134889	3.16E-05	0.000438339
FRRS1	-5.877089209	0.00012129	0.001234731
MYL9	-5.877126156	0.000108661	0.001128953
JADE2	-5.878757692	2.30E-05	0.000341799
ENSG00000256029	-5.880199374	0.009211098	0.034336657

TRIM27	-5.880859947	0.000323465	0.00260667
NCKAP1L	-5.88105785	0.00110249	0.006645689
MAN2B2	-5.882897458	3.25E-06	6.35E-05
FAM98B	-5.885600407	6.10E-06	0.000110921
MDM1	-5.885840314	0.000430932	0.003203933
NRF1	-5.88647268	0.000104151	0.001090526
ENSG00000267228	-5.887150935	0.003261318	0.015353945
WRAP73	-5.887159502	0.005073439	0.021760773
CDKN2AIP	-5.887180591	0.000237655	0.002067855
PYGL	-5.888138268	0.00124771	0.007338331
LMNB1	-5.888173602	0.003997403	0.017989865
LMBRD2	-5.888762788	0.001646379	0.00900894
HLA-DRB3	-5.888782714	0.018419351	0.058135027
OXCT2P1	-5.889656425	0.008383596	0.031929099
NAA20	-5.889830944	6.59E-05	0.000784981
ARHGAP24	-5.892340071	7.10E-06	0.000125868
VAMP8	-5.895525353	0.006474919	0.026141569
FAM234A	-5.896880156	5.27E-05	0.000652077
GTF2A2	-5.898284983	0.000304523	0.002487142
GJA1P1	-5.898410348	0.000170607	0.001598317
RPUSD3	-5.900136291	9.90E-07	2.27E-05
PTGES2	-5.90021009	0.000245112	0.002116144
ENSG00000267022	-5.901108277	0.000932218	0.005846407
PRPF38B	-5.901973103	0.000501072	0.003599393
TMEM50A	-5.905188914	0.000368235	0.002837117
TMEM199	-5.906308372	0.003111863	0.014805816
LYSMD3	-5.907285973	0.000171278	0.001601252
ODF2	-5.907338576	0.000266538	0.00225292
BACE2	-5.90768061	2.33E-05	0.000343272
JPT2	-5.909491718	7.12E-05	0.000828792
SDSL	-5.909685902	1.54E-05	0.00024183
DCK	-5.909801893	0.002254263	0.011508055
ARL14EP	-5.912075677	0.001903619	0.01012809
AATK	-5.912318639	0.000517034	0.003685123
PPM1D	-5.912790141	0.000632682	0.004346186
IGKV3-20	-5.913173606	0.002327604	0.011822627
ENSG00000267645	-5.913610845	0.003697985	0.016948279
CLPB	-5.913617087	6.35E-05	0.000761257
C11orf58	-5.91436107	1.52E-06	3.29E-05
ADAMTSL1	-5.914492805	0.000347881	0.002731085
RSU1	-5.914806625	0.000786616	0.005137541
WDFY4	-5.91591479	0.00126168	0.007391758
RB1	-5.916888527	0.000100336	0.001060674
KIAA1191	-5.919024034	4.18E-11	1.90E-09

PGM2	-5.920415524	0.002511629	0.012541568
METTL25	-5.920660116	0.000748612	0.004927243
NFASC	-5.922498634	2.96E-05	0.000416749
LRP12	-5.923305873	1.44E-05	0.000228072
VAMP4	-5.923444062	3.36E-05	0.000458753
ARHGAP26	-5.923582401	7.41E-06	0.000130517
LRGUK	-5.92497018	0.008219772	0.031454984
IKZF3	-5.926591658	0.008062751	0.030991278
THAP3	-5.927762805	0.0013019	0.007568778
KRI1	-5.928277087	5.92E-06	0.000107679
PCDHGB2	-5.928503711	0.001319952	0.007637044
TSPOAP1	-5.929172666	0.000392114	0.002971916
VARS1	-5.933654421	9.18E-05	0.001001142
RTL8C	-5.934425234	0.000297634	0.002440759
AGER	-5.93515299	5.06E-06	9.39E-05
ZMAT2	-5.936109247	0.002005453	0.010506709
TAS2R30	-5.937919019	0.006843478	0.027310733
EGFLAM	-5.938641532	0.001150944	0.006889727
FAM50A	-5.939649469	0.000898119	0.005674182
DNAJC8	-5.939689064	8.40E-06	0.000145063
ZBTB22	-5.940140187	0.000958341	0.005979256
TAMALIN	-5.940503172	0.004388625	0.019436485
SOCS6	-5.94098244	0.000265113	0.002244302
SLIRP	-5.942753086	0.000300021	0.002455342
SPOUT1	-5.943156444	0.000272494	0.002287287
RAMP3	-5.944763536	0.00733731	0.028815966
ERH	-5.945259623	0.000139497	0.001378348
SRPX2	-5.945801894	0.001511359	0.008475041
ENSG00000255330	-5.945931092	0.001759121	0.009488593
TSG101	-5.946279625	0.001168488	0.006984387
PRKCE	-5.947524731	0.001022735	0.006274295
XAF1	-5.947746582	0.00010958	0.001134602
TAF4	-5.949089516	0.000660283	0.004489925
TUFM	-5.949466501	0.000387395	0.002946535
HEXD	-5.95015678	0.000258524	0.002199002
MOSPD2	-5.950403852	0.000243734	0.002109175
FBXO16	-5.95109269	0.003055944	0.014620217
VASP	-5.951380836	3.85E-05	0.00050883
SFT2D2	-5.952409136	0.000212559	0.001894563
FLOT1	-5.963398904	0.000941712	0.005893735
JPH1	-5.965464928	0.00082917	0.005322208
CD82	-5.967293117	2.08E-06	4.32E-05
ATP5MJ	-5.96765066	6.88E-05	0.000808191
CNKSR3	-5.968493427	0.000892035	0.005647066

DIPK2A	-5.968642519	0.000332351	0.0026553
ZNF550	-5.968705076	1.58E-05	0.000247231
DNMT3B	-5.970607233	0.000324575	0.002613878
ENY2	-5.970832781	0.000639298	0.00437673
ABHD16A	-5.97371037	0.000806835	0.005219656
GSDME	-5.973824731	0.003533779	0.01634897
SP140	-5.97459237	0.001613458	0.008880007
RPS6KL1	-5.975076299	0.002409568	0.012132034
APH1B	-5.975914323	2.75E-05	0.000393897
ERMAP	-5.978325476	0.003162669	0.014988622
SEC22A	-5.978924494	0.000981436	0.006084019
SLC22A17	-5.980506086	0.000175563	0.001627721
BCAT2	-5.98096618	4.04E-05	0.000527553
FGR	-5.982498958	0.003487245	0.016172265
GOLGA8Q	-5.985401972	0.002008991	0.010513099
OSTM1	-5.988984255	0.000414295	0.003106954
KLHDC1	-5.989202591	0.00086073	0.005491976
USP30	-5.989804428	0.002222713	0.011404712
KCTD18	-5.989955703	3.00E-05	0.000420067
FAM118A	-5.99084507	0.000616629	0.004255229
RRAS2	-5.991994636	0.000616368	0.004255229
INPP5F	-5.993113838	0.008509982	0.032268153
TRAP1	-5.994398618	7.83E-07	1.87E-05
BCL6	-5.997570219	0.000267838	0.002259172
ITGA4	-5.998555331	0.000705923	0.004715506
ADAMTS3	-5.999323264	0.0013652	0.007852952
SV2A	-6.000073965	0.0010395	0.006354601
PYGM	-6.000542715	0.004308568	0.019166082
COX6C	-6.000852926	4.24E-05	0.000547641
NDUFS6	-6.004037653	0.000200367	0.001805842
BOD1L1	-6.004329294	0.000194287	0.001759615
TRIM44	-6.004802835	0.002482017	0.012439887
ADAM28	-6.006046621	0.003105613	0.01479352
PCOLCE	-6.006948225	6.83E-09	2.53E-07
CTDP1	-6.007262713	0.001235124	0.007285543
HLA-DQB1	-6.009693636	0.00374982	0.017082356
ENSG00000254704	-6.010067663	0.000969091	0.00603389
STX7	-6.013988205	1.68E-05	0.000260355
SLC16A9	-6.013991573	1.39E-05	0.000222235
PIGP	-6.015391216	7.31E-07	1.77E-05
TANC2	-6.017469868	2.40E-06	4.87E-05
PYROXD1	-6.018542106	0.000369357	0.002843958
TRMT112	-6.018668107	2.08E-05	0.000312218
PKIG	-6.019399271	1.81E-05	0.000277786

TFB1M	-6.020149243	0.000471128	0.00342906
MRRF	-6.020181257	0.001017141	0.00624976
ZNF772	-6.020240174	9.35E-06	0.000158949
ELOVL7	-6.023296893	0.001418455	0.008090942
MTRF1L	-6.024247704	0.000245473	0.002117751
PIGA	-6.024271241	8.05E-07	1.89E-05
SELE	-6.024700441	0.008514911	0.032268153
RPL12	-6.025189755	2.24E-06	4.59E-05
RAB8B	-6.025203496	0.000643099	0.004400267
SERPINB8	-6.025652462	4.86E-05	0.000611539
DHX36	-6.026837326	0.006218406	0.025325566
TAF6L	-6.027434961	0.003147914	0.014942099
DBF4B	-6.028221626	0.001343141	0.007756381
DNAJC7	-6.02931791	0.003691376	0.016924398
ARHGEF16	-6.029953218	1.96E-05	0.000297347
TCF3	-6.03119494	0.000307019	0.00250415
TTC5	-6.031894417	0.000390798	0.002967513
QPCT	-6.032713304	0.002863713	0.013881611
S100A14	-6.033093057	4.12E-07	1.07E-05
HERC2P4	-6.035043162	0.002950966	0.014230475
GPN2	-6.040452092	0.001017187	0.00624976
TOMM40	-6.042609377	0.000193591	0.001755229
CEP170	-6.042955618	0.00617902	0.025199076
GABRE	-6.044311758	1.50E-05	0.000236267
ENSG00000250848	-6.044557893	0.000889683	0.005636997
WDR17	-6.044563186	3.09E-05	0.000430693
DPP6	-6.046523175	0.003407436	0.015911885
DAGLB	-6.047809773	0.000576382	0.004023374
IMPA1	-6.047827834	0.00016749	0.001575216
SLC5A6	-6.049258157	0.000769473	0.005037155
H2BP1	-6.049516901	0.008244573	0.031529957
LRRC51	-6.049816697	5.95E-05	0.000722275
ANK2	-6.050684139	0.002020817	0.010560407
XYLT2	-6.05096229	1.72E-05	0.000264453
NPAS3	-6.05119447	0.00071158	0.004745432
AP3M2	-6.051466604	0.000276238	0.002307509
NTRK3	-6.053469925	0.000350094	0.002742742
IL7R	-6.053746631	0.006870503	0.02738247
PDZD11	-6.055874736	0.002401661	0.012115876
BACH2	-6.05647105	0.003140975	0.014916279
EMC8	-6.057589995	4.30E-05	0.000554157
ZNF763	-6.05780791	0.00339449	0.015857546
MRPL49	-6.057903808	0.001046827	0.006377474
RDH14	-6.058320091	0.00071262	0.004749752

ZBTB37	-6.059543401	9.05E-06	0.000154487
ENSG00000206356	-6.064420804	0.000634404	0.004352433
CYB5D2	-6.067463848	8.83E-05	0.000973601
POLR1D	-6.068352747	9.08E-05	0.000993261
MS4A7	-6.068825482	0.000160652	0.001524042
VPS50	-6.070168309	3.60E-05	0.000483127
DNAJB5	-6.07034128	7.15E-05	0.000831871
CBFB	-6.071478004	0.000257474	0.002193151
ZNF257	-6.073049135	0.002521838	0.01256947
TP53I11	-6.0745199	0.000232635	0.002033079
EXOC1	-6.07453526	5.13E-08	1.60E-06
FKBP11	-6.075885807	1.24E-07	3.58E-06
GASK1B	-6.07597445	0.000761647	0.004994029
ENGASE	-6.076609833	1.06E-05	0.000176691
EDEM2	-6.076898413	0.001143279	0.00685401
ENSG00000274559	-6.080101087	0.001981237	0.010440094
DHRS7B	-6.080316256	4.65E-05	0.000591128
JRK	-6.083444353	1.67E-05	0.000259918
AP3S1	-6.084608219	9.20E-05	0.001002452
SQLE	-6.084995072	0.000191007	0.001739068
GALM	-6.085995829	0.000615797	0.004254342
FBXW4	-6.087578664	6.50E-05	0.000774578
B4GALT3	-6.089004044	0.000104366	0.001091764
RASA4B	-6.091502971	0.000304795	0.00248769
OR7E13P	-6.092046232	0.002597981	0.012866594
ABCB7	-6.092904987	1.10E-05	0.000181766
TIMM21	-6.094248045	0.000695081	0.004668845
MXRA7P1	-6.096387148	1.42E-05	0.000227521
ZSWIM4	-6.09932109	6.32E-05	0.000759003
CAMK2G	-6.100137947	4.78E-05	0.000603903
SCN3A	-6.100455375	0.000378335	0.00289467
MPV17L	-6.101363531	0.000333088	0.002657677
ARMCX6	-6.101474165	4.82E-05	0.000607941
DUS2	-6.1037624	4.65E-05	0.000591128
FOXP1	-6.10456089	6.10E-05	0.000735994
GATAD1	-6.105886813	0.000462857	0.003387193
KATNB1	-6.10608138	0.002474404	0.012406869
NBPF3	-6.111078516	0.001104291	0.006653229
RPS6KA4	-6.111882857	2.92E-05	0.000411979
APOLD1	-6.112880798	0.007358339	0.028861093
MRPL9	-6.113038507	3.00E-05	0.000420067
GRB7	-6.113886242	0.000651364	0.004436753
PPP2R2D	-6.113918831	0.000119785	0.001222498
POLR2M	-6.114977342	0.00023786	0.002067855

SDHAF2	-6.115086658	0.016956469	0.054498714
NEDD8	-6.116566272	0.00017132	0.001601252
IGLC1	-6.11727111	0.000539799	0.003825368
GLI3	-6.120366757	9.94E-05	0.001054911
TPH1	-6.124417005	2.57E-05	0.000372677
PLEKHH3	-6.125815324	9.18E-07	2.11E-05
SPTLC1	-6.126437385	3.55E-05	0.000479091
ZNF286A	-6.126690103	0.000101718	0.001073091
CAPZA1P2	-6.127253103	0.000214717	0.001906772
NDFIP2	-6.128583007	0.006007102	0.024714715
PTAR1	-6.128974186	4.36E-05	0.000559509
TMEM164	-6.129456048	0.00399263	0.017989865
DDX19B	-6.130193848	3.95E-05	0.00051805
TMEM132B	-6.131252502	0.000231374	0.002023521
ALG11	-6.132951841	2.96E-05	0.000416749
PIGK	-6.134952532	8.34E-06	0.000144154
TSTD2	-6.135594216	0.00028896	0.002392318
CLIP3	-6.13676277	0.002097997	0.010880102
VPS11	-6.137307236	0.001507376	0.008462559
UFSP2	-6.139148542	8.17E-05	0.000920174
DNPEP	-6.140898727	1.95E-05	0.000296871
FYCO1	-6.144197781	0.005703254	0.023743574
PLVAP	-6.144865129	1.37E-05	0.000219865
PCYT1A	-6.14589255	1.15E-07	3.36E-06
CDK2	-6.146335845	1.42E-07	4.02E-06
COMMD6	-6.148681421	1.67E-06	3.57E-05
TMEM183A	-6.150684657	0.00012985	0.001305403
PARVB	-6.151006308	0.000159846	0.001522244
PLCL2	-6.151131009	0.000193273	0.001753824
RGS1	-6.151783629	0.006570566	0.02644833
CAPN5	-6.15248385	0.000366261	0.002825505
DDX54	-6.152518041	1.17E-06	2.63E-05
TUBG1P	-6.152524318	0.000109315	0.001133808
PDE4B	-6.15309524	1.13E-06	2.55E-05
PRPF40A	-6.154431324	0.000162805	0.001540731
ALOX15B	-6.156449782	0.000182027	0.001674203
GTF2E1	-6.160491395	0.00067655	0.004572283
TM4SF1	-6.163751026	5.88E-05	0.000716198
AGPAT1	-6.164930988	1.84E-07	5.16E-06
SDHD	-6.169649761	0.000236061	0.002057434
ARMC2	-6.170253014	0.001057662	0.006433133
P4HA1	-6.171668241	0.00010445	0.001091764
B3GALNT1	-6.173585423	4.65E-05	0.000591128
GMEB1	-6.175294272	8.98E-05	0.000985148

ATP6V0A2	-6.176712118	8.25E-07	1.92E-05
GKAP1	-6.180400169	0.000514763	0.00367159
LYN	-6.180760739	0.001257758	0.007375924
STK38L	-6.182671276	1.55E-08	5.40E-07
ENSG00000255730	-6.183233028	0.005917028	0.024410263
SPOCK2	-6.187835018	0.000212816	0.001895455
CRISPLD2	-6.188376214	2.31E-05	0.000342208
AMACR	-6.189644345	0.00014076	0.001387421
FBXL6	-6.190309727	0.000553716	0.003898885
ZNF500	-6.191778762	4.88E-05	0.000613241
NCAPG2	-6.19182603	0.000251743	0.002153427
TOPBP1	-6.193040396	0.002709869	0.01327408
ZNF267	-6.193101998	0.000694839	0.004668845
MAGT1	-6.193760586	1.39E-05	0.00022228
RPRD1B	-6.193824695	0.00013321	0.001328153
CSTF1	-6.194537129	9.79E-05	0.00104454
NUDCD1	-6.194734805	0.000146783	0.001433953
ARMC10	-6.196421042	5.21E-05	0.0006467
TMEM62	-6.199001343	0.000676061	0.004571531
ECM2	-6.199536227	9.44E-05	0.001016584
NEB	-6.199711978	0.000736723	0.004878172
SLC7A7	-6.201100336	2.27E-06	4.63E-05
WDFY1	-6.201998898	0.000102181	0.001076413
LCA5	-6.204343175	0.000257919	0.002195392
DHRS12	-6.20502947	0.002877849	0.013933392
FRS3	-6.206040176	8.04E-05	0.000909011
RWDD2B	-6.206110313	0.000886994	0.005629874
ZNF559-ZNF177	-6.207365025	0.000742509	0.004911109
SH3BP1	-6.207393954	0.002839127	0.013790044
TXNRD2	-6.207579848	0.000797366	0.005186094
NDUFAF4	-6.210611811	0.00195616	0.010339457
TSPAN12	-6.212387742	0.000584912	0.004071176
SNRPF	-6.213046869	0.000863358	0.005505843
HARS1	-6.215707884	4.30E-05	0.000554052
CDK5RAP1	-6.217609349	7.44E-05	0.000858919
SLBP	-6.218634666	2.55E-05	0.000370721
NEU1	-6.220687182	3.83E-06	7.31E-05
GABPB2	-6.223717	0.000403875	0.003047693
PLEKHF2	-6.224871882	8.33E-05	0.000932201
HMGN3	-6.225126758	4.49E-05	0.000573885
BIVM	-6.226324591	0.000172	0.001603178
PEX3	-6.227658174	0.000548754	0.003870865
ZNF92	-6.22904776	0.000321338	0.002594717
AGAP7P	-6.2316903	0.000102936	0.001081541

POMT1	-6.23254901	9.87E-05	0.001051287
PDZRN4	-6.232680324	0.015820959	0.051843286
MALLP1	-6.233498747	0.002021519	0.010560407
HSD17B10	-6.234845255	0.000482935	0.003496082
ZFPM2	-6.234900203	6.77E-05	0.000799074
FLCN	-6.238877528	2.57E-08	8.72E-07
WTIP	-6.239770797	0.000690326	0.004647222
C5orf51	-6.239964285	0.005552004	0.023277264
ST20-MTHFS	-6.240409288	0.000705748	0.004715506
TDP1	-6.242114453	0.001684254	0.009170585
SMNDC1	-6.24257458	0.000144225	0.001413517
KCTD9	-6.247059532	9.40E-06	0.00015953
IQUB	-6.248110996	3.17E-05	0.000438339
ACADSB	-6.248808873	0.001865454	0.009946894
PLSCR4	-6.250172793	0.000265803	0.002248272
SERPINE2	-6.25109641	5.85E-05	0.00071471
ZNF256	-6.252766794	0.005024163	0.021587671
RAB25	-6.254383315	0.00093602	0.005861141
MICOS10	-6.254440955	2.58E-05	0.000373564
NAIP	-6.258444113	1.24E-07	3.58E-06
TMEM184A	-6.260006582	0.000109548	0.001134602
ZNF737	-6.260969003	2.38E-05	0.000349893
LDAH	-6.262491021	0.000156677	0.001498363
ZNF548	-6.263578802	7.39E-05	0.000855072
CFB	-6.263869139	0.00012933	0.001301258
DNAJB12	-6.269681348	4.95E-05	0.000617444
RALA	-6.271566193	3.57E-06	6.87E-05
CIBAR1	-6.273312846	6.46E-06	0.000116239
COA8	-6.275628426	4.03E-05	0.000525626
P2RX4	-6.278680265	8.00E-09	2.94E-07
TENT5A	-6.280134195	0.000347609	0.002731085
DDOST	-6.280497635	0.001437739	0.008181664
SLC25A11	-6.284946522	0.00054632	0.003862536
MAPRE2	-6.28751404	9.73E-06	0.000163904
RAD54L	-6.290359243	0.001806573	0.009671278
CYP2D6	-6.292350812	0.00616367	0.025144949
PLXNA4	-6.293136356	9.61E-05	0.001030585
SH3BP4	-6.294625036	8.11E-06	0.000140882
MST1L	-6.298873882	0.001029215	0.006304463
EMG1	-6.299102869	0.000175763	0.001627721
ZNF799	-6.300997106	0.000429727	0.003196936
OLFML1	-6.302419138	0.00182814	0.009769449
GNPDA1	-6.303095692	5.85E-05	0.00071471
CLPP	-6.303530534	0.000694159	0.004667831

ARSG	-6.30473807	0.000465771	0.003403809
B2M	-6.305504007	3.05E-08	1.01E-06
HMGN2P20	-6.30869497	0.000534731	0.003799004
ALKBH6	-6.309426625	0.000362492	0.002807168
SGPL1	-6.311358729	0.000177433	0.001639845
APOC1	-6.313175738	0.000367419	0.002832637
TRIM27	-6.316977932	0.001547288	0.00862661
HLA-DRA	-6.318001083	0.002261256	0.011534025
RNF6	-6.322211609	8.48E-07	1.97E-05
SETD7	-6.323515175	1.54E-05	0.00024183
NT5C	-6.325924227	3.91E-06	7.42E-05
SEM1	-6.327026837	0.000448787	0.003306297
ZFYVE21	-6.328336057	4.08E-06	7.73E-05
CCDC191	-6.329762455	5.94E-05	0.000721565
EEF1A1P4	-6.331183027	7.95E-05	0.00090428
RCC1	-6.33229854	0.000174176	0.001619688
HSD17B7	-6.332368498	0.001269013	0.007431126
FIS1	-6.334902163	0.000219253	0.001941359
CCNT1	-6.335615406	1.56E-05	0.000245025
MPHOSPH9	-6.335887666	2.89E-09	1.13E-07
SEC63P1	-6.336584585	0.000244188	0.002109676
DCAF4	-6.338267173	9.39E-05	0.00101587
BMPR2	-6.339263938	0.003723043	0.017026525
MRFAP1	-6.341392736	2.62E-07	7.18E-06
GARNL3	-6.341747384	2.31E-05	0.000342208
ZNF74	-6.344648256	1.07E-05	0.000178246
DNAAF1	-6.345502921	1.35E-05	0.000218284
UQCRH	-6.346090731	1.45E-06	3.15E-05
SPAG7	-6.352376567	1.52E-05	0.000239099
MDFIC	-6.352789774	0.00147957	0.008345162
PTPN9	-6.355906635	0.000346936	0.002730376
FAM169A	-6.356223247	0.00011007	0.001138707
SLC29A2	-6.357878425	8.12E-06	0.000140894
CYB5A	-6.360326743	3.62E-05	0.000484826
NTAN1	-6.36661681	4.23E-07	1.09E-05
COQ7	-6.368174592	0.001621589	0.008897423
AAAS	-6.368676121	7.00E-06	0.000124217
ENSG00000280518	-6.36873073	0.000294832	0.00242271
SMIM29	-6.371613372	3.07E-05	0.000428103
DHODH	-6.374055561	7.24E-05	0.000840454
HLA-DPB1	-6.375041275	0.000984661	0.006096334
NSDHL	-6.375780659	0.000506155	0.003625954
HYAL2	-6.377037045	0.000182895	0.001679636
SYVN1	-6.377319257	2.36E-08	8.08E-07

SMC1A	-6.377560098	0.000106087	0.001106011
SURF1	-6.378384872	0.001614695	0.008880007
AKAP17A	-6.38131518	9.57E-06	0.000161954
HAT1	-6.381432335	0.000456496	0.003346719
PDGFRA	-6.381928332	0.00225359	0.011508055
DERL1	-6.386673107	7.80E-05	0.000891369
STAG3L5P	-6.387084156	2.45E-06	4.94E-05
BCL7B	-6.389155054	9.17E-07	2.11E-05
TAF4B	-6.389467415	0.000213779	0.001901231
TRPM2	-6.390069427	0.00012366	0.001253587
RING1	-6.390363627	0.004236344	0.018879493
AFAP1L1	-6.392759567	1.52E-06	3.28E-05
FAM114A2	-6.392820297	4.83E-08	1.51E-06
ENSG00000271741	-6.393406053	6.05E-05	0.000732619
COG2	-6.395983363	1.43E-05	0.000227833
ADAM22	-6.39672071	0.000431913	0.003206431
ATG4C	-6.39679457	0.00013362	0.001329377
ZNF555	-6.397698479	0.000386027	0.002944219
PLAAT4	-6.398359837	0.00157137	0.008728824
RGPD2	-6.401392843	0.001933184	0.010235897
C11orf80	-6.405131905	0.000140979	0.001388455
FHOD3	-6.406259502	0.001476381	0.008334944
ENSG00000215493	-6.408218524	0.001278333	0.007465421
MCU	-6.408523812	0.000123808	0.001254039
ENSG00000284431	-6.409994903	0.004526825	0.01986573
ENSG00000286185	-6.412734509	2.01E-05	0.00030243
COX10	-6.418427153	0.001786578	0.009593938
CLMN	-6.418464828	1.08E-07	3.17E-06
KRTCAP3	-6.418854476	0.001629472	0.008924495
KIRREL1	-6.421104536	0.000193292	0.001753824
MRPS18B	-6.42186219	9.99E-06	0.000167662
CATSPERG	-6.427131622	5.72E-05	0.000702401
DHX33	-6.428315925	4.95E-05	0.000617444
EBF4	-6.431271685	0.000165394	0.001559135
SCAND2P	-6.431935588	0.000221018	0.001951279
KMT2B	-6.434157429	0.002612195	0.012919084
MRPL37	-6.437754355	2.47E-06	4.98E-05
SLC10A3	-6.438869108	0.000353576	0.002757524
SNX11	-6.441112776	0.000382733	0.002922774
DRG1	-6.443143619	8.61E-05	0.000954459
ITK	-6.445031063	0.000748418	0.004927243
DCTN6	-6.445191954	7.54E-05	0.000868649
ABHD3	-6.445766152	7.58E-05	0.000870441
ACAA2	-6.4474061	4.35E-07	1.11E-05

DPYD	-6.447614754	0.000235648	0.002056438
GNA13	-6.448385928	8.05E-06	0.000139969
JHY	-6.453544276	0.001256675	0.007375924
ZNF563	-6.455198276	0.004522244	0.019861117
SMIM30	-6.456052628	0.00233354	0.011842837
KCTD2	-6.460829003	0.000633093	0.004346546
DTWD1	-6.462639602	1.71E-06	3.64E-05
EDN3	-6.464249566	0.004165842	0.01859954
ZC2HC1A	-6.466314993	9.41E-05	0.00101587
IGKV4-1	-6.469495314	0.001757707	0.009488593
ZNF569	-6.471492165	3.57E-08	1.15E-06
RAB24	-6.472672002	0.000236102	0.002057434
CTNNBL1	-6.473047471	6.36E-05	0.000761537
EHD1	-6.476085282	1.48E-07	4.18E-06
ENSG00000250424	-6.477531485	0.004104695	0.018360393
ZFP64	-6.479222088	0.001769205	0.009526006
ULK4	-6.481371592	4.86E-06	9.07E-05
MTIF3	-6.483258682	4.25E-05	0.000549014
NFKB2	-6.487830352	5.74E-07	1.43E-05
IGHV3-21	-6.488067256	0.004987658	0.021481465
MINDY3	-6.489709353	2.74E-08	9.22E-07
MED6	-6.489943715	1.59E-06	3.41E-05
CC2D1B	-6.490151554	3.25E-08	1.05E-06
LDLR	-6.494842087	1.64E-09	6.58E-08
TOMM70	-6.496992313	3.17E-05	0.000438339
MIIP	-6.502909719	0.000847225	0.005414369
WDR76	-6.503768918	3.64E-05	0.000486171
GPR171	-6.515448318	0.001614747	0.008880007
IL3RA	-6.5160614	0.00049446	0.003560344
GLDN	-6.516109745	0.001628453	0.008924495
CCDC25	-6.518022532	0.000548778	0.003870865
IARS1	-6.519256317	0.000834986	0.00535311
NCBP2	-6.520188646	1.32E-05	0.000213186
TMEM150A	-6.522035613	2.20E-05	0.000326825
ACTL6A	-6.523285386	5.06E-06	9.39E-05
RSRC1	-6.524993261	6.21E-06	0.000112615
TSGA10	-6.527475246	8.27E-08	2.45E-06
OR7E38P	-6.529742744	0.000182563	0.001677862
MSANTD2	-6.530553794	0.000196047	0.00177218
TSPAN9	-6.533741501	0.000267195	0.00225689
MGAT4A	-6.534260298	9.30E-09	3.33E-07
ZNF25	-6.53490095	6.49E-06	0.000116364
SMN1	-6.540271319	0.000409069	0.003077301
CYP1B1	-6.541264262	0.000319403	0.002582534

TEX10	-6.54279163	4.17E-07	1.07E-05
EMP3	-6.54486494	6.72E-05	0.000796263
STAT4	-6.545322204	0.000684908	0.004615883
PIK3CG	-6.553710013	0.000514449	0.003671515
LTF	-6.553849877	0.001003803	0.006180076
ARHGAP9	-6.564132118	2.68E-06	5.36E-05
EOLA2	-6.564634034	0.000156053	0.001496487
GXYLT1	-6.566420064	6.97E-07	1.70E-05
EEF2KMT	-6.567716574	0.000531902	0.003784918
TMEM127	-6.568627863	4.55E-07	1.16E-05
NEU1	-6.570875098	0.000793401	0.005167592
DCTD	-6.571545107	0.000191879	0.001744931
WDR91	-6.575003253	4.14E-05	0.000537223
SIRT3	-6.577010732	2.07E-05	0.00031027
RAB23	-6.578918784	3.66E-06	7.03E-05
TYW5	-6.579026378	1.03E-08	3.68E-07
RCOR1	-6.585985245	0.000507315	0.00362927
NDUFB8	-6.586414148	8.89E-06	0.000152364
PPP1R7	-6.59145662	2.50E-07	6.88E-06
FOXJ2	-6.596383459	2.23E-06	4.58E-05
YIPF1	-6.597867603	5.05E-05	0.000626556
ZNF704	-6.599846858	9.26E-06	0.000157811
PDZK1	-6.603951935	0.000387315	0.002946535
TBP	-6.606951711	1.24E-07	3.58E-06
CD47	-6.607547458	3.03E-07	8.06E-06
THAP6	-6.611335114	0.000133878	0.001330429
SYCP3	-6.614168011	6.53E-07	1.61E-05
ZC3H15	-6.614977345	3.94E-05	0.000516303
ADAM19	-6.616194909	4.19E-05	0.000542417
CCDC7	-6.616338015	1.64E-06	3.50E-05
INPP5D	-6.617170866	8.27E-06	0.000143224
KLHL18	-6.617206392	5.77E-05	0.000708061
SDHA	-6.618986992	4.13E-08	1.31E-06
PLPBP	-6.620159535	7.48E-06	0.000131341
C19orf12	-6.625646061	6.44E-06	0.000115972
TXN2	-6.626999624	1.58E-06	3.39E-05
FADS3	-6.628339223	1.64E-06	3.50E-05
ARHGAP18	-6.630246162	0.000370938	0.002848969
GFM2	-6.631604868	3.72E-05	0.000494653
ANKRD49	-6.631713283	4.77E-05	0.000603758
GCDH	-6.632737892	3.85E-05	0.00050883
CWC22	-6.633507779	0.001179393	0.007029502
HACD3	-6.637220411	1.31E-08	4.60E-07
PSMD6	-6.638705366	2.04E-06	4.25E-05

CA2	-6.640091802	5.32E-05	0.000656262
CYP4B1	-6.640632597	4.68E-05	0.000593828
AP1M2	-6.642188536	0.000363419	0.002810753
SLC27A6	-6.642239372	0.00356486	0.016450274
PLOD3	-6.642583553	5.43E-10	2.30E-08
DEPDC5	-6.643116726	6.86E-05	0.000807857
RXRB	-6.644271619	7.84E-06	0.00013658
LDLRAP1	-6.646608349	8.66E-08	2.56E-06
TLR3	-6.646753566	7.45E-06	0.000131086
POLD3	-6.64691557	0.000315474	0.002557598
DRAM2	-6.647416089	0.000344586	0.00271895
TECTA	-6.648469977	6.86E-05	0.000807857
TTC23	-6.649229511	1.56E-06	3.36E-05
RNF14	-6.650722403	2.61E-06	5.24E-05
POP1	-6.65133047	0.000355758	0.002767413
NEK8	-6.651734646	4.54E-05	0.000579016
NR2F1	-6.651841527	0.000229161	0.002009977
GIPC1	-6.652706875	2.10E-09	8.38E-08
ZNF322	-6.654449181	3.06E-05	0.000427258
EPC2	-6.654630572	3.20E-06	6.26E-05
C6orf136	-6.654806073	2.28E-06	4.66E-05
PLIN3	-6.661495461	9.76E-09	3.49E-07
ENSG00000212664	-6.66371024	5.33E-06	9.87E-05
TFAM	-6.66613479	9.93E-05	0.001054296
ACADM	-6.668225903	1.08E-06	2.46E-05
TVP23B	-6.672505425	2.71E-07	7.38E-06
H2BC18	-6.67506939	0.002214896	0.011379076
OSBPL3	-6.678374728	8.09E-07	1.89E-05
CCS	-6.679522432	1.39E-05	0.000222522
COPS2	-6.682535373	4.07E-10	1.75E-08
CDK18	-6.685319634	4.52E-06	8.50E-05
ZNF589	-6.685481885	0.000162493	0.001538981
B3GNT5	-6.686436456	3.90E-05	0.000513309
TEDC1	-6.687503044	3.51E-06	6.77E-05
LRMDA	-6.687511883	0.00015374	0.001483582
MAP3K11	-6.691932325	3.02E-09	1.18E-07
UBE2L6	-6.694325419	5.38E-06	9.94E-05
HNRNPAB	-6.695230604	4.99E-06	9.28E-05
CAPZA2	-6.702367606	1.50E-08	5.26E-07
MRPL1	-6.705481737	3.67E-05	0.000488599
ARMCX5	-6.707594341	1.91E-07	5.32E-06
BMS1P2	-6.709357323	0.001272337	0.007443389
CENPO	-6.710764008	5.04E-05	0.000626037
LNPK	-6.716031283	0.001093179	0.006602715

ZDHHC13	-6.717027698	1.80E-06	3.80E-05
KLF7	-6.718043361	3.64E-05	0.000486171
IKZF1	-6.718256218	0.00021958	0.001941414
LAMTOR2	-6.720162004	0.00112961	0.006778782
ADAL	-6.72814464	0.000371018	0.002848969
GOLGA6L3	-6.733764878	9.23E-05	0.001003427
CEP85L	-6.736657199	1.54E-05	0.00024183
CSRNP3	-6.738230993	0.000626576	0.004311584
TPCN2	-6.741130279	0.000131186	0.001313375
ANKRD36BP2	-6.741497517	7.41E-05	0.000856945
DHTKD1	-6.744876481	2.21E-06	4.53E-05
RPL17P3	-6.747458176	1.79E-08	6.16E-07
DNAJC5	-6.749352647	0.000789656	0.005152558
ENSG00000244716	-6.753223063	1.82E-06	3.84E-05
SP1	-6.758726144	8.08E-07	1.89E-05
POLR2C	-6.758903114	3.67E-05	0.00048915
CDK14	-6.760561089	1.56E-06	3.35E-05
CHCHD2	-6.761485206	0.000876703	0.005575981
IL32	-6.762505478	4.82E-05	0.000607944
DCUN1D4	-6.762940709	1.73E-06	3.68E-05
ANAPC7	-6.774449465	1.27E-07	3.64E-06
RCE1	-6.778310734	4.22E-07	1.08E-05
INMT-MINDY4	-6.780967986	0.000197248	0.00178038
ZNF732	-6.782398146	0.000290704	0.002403473
PRMT3	-6.78321972	3.03E-06	5.99E-05
UBE2L3	-6.784466162	6.77E-07	1.66E-05
OGDHL	-6.788957946	5.84E-05	0.00071471
PEX26	-6.789076756	4.57E-10	1.95E-08
PIM2	-6.790040374	0.000359029	0.002787481
MTMR2	-6.791188124	0.00026152	0.002221361
UTP25	-6.792237993	5.81E-05	0.000711827
ZNF28	-6.792695521	0.000126981	0.00128403
PREB	-6.796490909	1.14E-07	3.32E-06
IPO13	-6.79662635	2.16E-05	0.000322224
PIGO	-6.797314784	1.35E-05	0.000217425
RAB1B	-6.802549136	4.00E-05	0.000522544
VPS26A	-6.805744519	1.07E-06	2.45E-05
FANCD2	-6.806093579	0.000178625	0.00164918
FRG1	-6.807137728	9.58E-06	0.000161974
CYP2C8	-6.81588168	3.78E-05	0.000500953
PGM3	-6.816874929	1.76E-06	3.72E-05
TTLL7	-6.8189133	2.92E-08	9.70E-07
SPCS1	-6.819866224	0.000540363	0.003826409
KNSTRN	-6.822177615	9.17E-05	0.00100059

ANKRD27	-6.823099325	0.000160042	0.001522912
ACSS2	-6.823675634	0.000270888	0.002277374
ALDH5A1	-6.823918837	3.81E-05	0.000503916
HDAC9	-6.824086755	1.84E-06	3.87E-05
DCAF13	-6.825557692	7.05E-08	2.13E-06
FCGR2B	-6.825567915	0.001205248	0.007147632
PTPRQ	-6.826228682	0.000106811	0.001112603
VARS1	-6.828586725	1.37E-06	2.99E-05
CAAP1	-6.834989869	4.90E-05	0.00061479
ABHD2	-6.835107919	7.28E-08	2.19E-06
GBP1	-6.836329758	0.000796893	0.005185801
MAP9	-6.839637995	7.15E-06	0.000126485
ALK	-6.840505354	0.000298846	0.002447383
MS4A6A	-6.841750816	2.41E-05	0.000354825
TTLL4	-6.84645991	1.47E-05	0.000232801
FANCC	-6.848465084	0.000391337	0.002969745
FAM247A	-6.850448246	0.006737814	0.027004802
MAN1B1	-6.850689995	4.45E-05	0.000569431
TEK	-6.852667604	0.000171495	0.001601252
DENND11	-6.853146066	6.66E-09	2.47E-07
PEDS1	-6.856054992	0.000338325	0.002681162
PAPSS1	-6.858269983	4.90E-05	0.00061479
ENSG00000258461	-6.860097218	0.001292344	0.007527687
MTMR9	-6.862156755	2.36E-06	4.78E-05
COX4I1	-6.862456793	3.28E-06	6.38E-05
PEF1	-6.86320757	7.22E-07	1.75E-05
SLC66A2	-6.863363335	2.91E-06	5.78E-05
TEX2	-6.864194481	1.96E-06	4.11E-05
UBXN2B	-6.865174991	5.78E-05	0.000709294
BBS2	-6.865304032	0.000426836	0.003183255
DMPK	-6.865808515	3.73E-08	1.19E-06
NME7	-6.867790044	2.05E-06	4.27E-05
RGPD1	-6.868944008	0.000179326	0.001654392
SCARB1	-6.86894556	1.86E-05	0.000285357
EFNA1	-6.870669212	2.32E-06	4.71E-05
PHYHD1	-6.878901726	0.000157879	0.001507068
YME1L1	-6.879550508	1.31E-06	2.89E-05
DDR2	-6.880537051	0.000213159	0.001897118
ENSG00000234742	-6.882458747	8.85E-06	0.000152138
CNTLN	-6.88470605	3.45E-05	0.000468057
NMT1	-6.885818994	0.000288036	0.002386304
SLC35B3	-6.886198367	3.90E-08	1.25E-06
CHCHD10	-6.887244821	1.06E-08	3.76E-07
MACROD2	-6.890873301	2.45E-05	0.000359378

BCL2L11	-6.8967171	5.42E-07	1.36E-05
LHPP	-6.899463812	3.29E-08	1.07E-06
NUP42	-6.899680975	7.47E-08	2.24E-06
ST3GAL3	-6.903053564	0.001079772	0.006544595
LETM1	-6.90408596	2.82E-05	0.000402203
BET1L	-6.904675363	2.83E-05	0.000402922
ENSG00000274944	-6.906649773	0.000932718	0.005846513
CTTNBP2	-6.908370369	3.32E-06	6.45E-05
CFB	-6.908576375	2.72E-06	5.43E-05
GPT	-6.909476864	0.000269418	0.002268052
EVC2	-6.913558307	0.000228152	0.002002577
ARHGAP33	-6.92056721	6.77E-06	0.000120829
ITGB6	-6.922384602	1.56E-05	0.000244149
NUDT5	-6.923407636	0.000107932	0.001122339
ENSG00000239969	-6.928547246	1.30E-06	2.87E-05
NMRK1	-6.929361169	1.30E-05	0.000211321
TAS2R19	-6.930523563	0.000187104	0.001711798
TYW3	-6.932405657	5.53E-09	2.08E-07
NEMP1	-6.939270181	3.19E-05	0.000439352
MARK4	-6.939291634	0.000204077	0.00182974
USP24	-6.944960682	1.83E-09	7.32E-08
STX3	-6.945532899	1.82E-08	6.25E-07
GPR89B	-6.95958776	0.001477096	0.008335091
SART3	-6.960122763	4.25E-11	1.91E-09
ANAPC15	-6.96136017	1.34E-06	2.95E-05
INCENP	-6.961402382	3.58E-09	1.39E-07
MID1	-6.972240358	7.52E-05	0.000867182
GMFB	-6.972662089	3.76E-06	7.20E-05
MYO3B	-6.973229715	4.19E-06	7.93E-05
KIAA1143	-6.977463941	5.59E-06	0.000102516
USO1	-6.98508345	0.000361526	0.002801482
ZMYM1	-6.989667358	5.19E-07	1.31E-05
LANCL2	-6.990505268	1.38E-06	3.01E-05
NBPF1	-6.991374619	1.38E-07	3.92E-06
ARMC6	-6.991875707	2.13E-05	0.000318904
STX5	-6.995807697	1.61E-05	0.000250607
GATD3B	-6.996769318	8.84E-05	0.000973601
IRAG2	-6.997944815	6.74E-05	0.000797375
EIF2B3	-7.002073497	2.93E-07	7.91E-06
CACNB3	-7.00598883	1.71E-05	0.000263492
EIF4H	-7.007040811	7.93E-10	3.32E-08
HEBP2	-7.011058562	1.07E-09	4.43E-08
SPRED2	-7.012225892	1.45E-08	5.09E-07
EMC2	-7.014611778	0.000338468	0.002681162

ACVR2B	-7.015000323	5.02E-07	1.27E-05
NRG1	-7.016260625	0.00083818	0.005367897
BPNT1	-7.016624107	6.91E-05	0.000809946
NPIPB6	-7.021678831	0.001030701	0.006310374
UAP1	-7.025296398	5.31E-13	2.98E-11
DPM1	-7.030265645	2.46E-05	0.000360759
GPM6B	-7.032492653	3.30E-07	8.75E-06
TBC1D3B	-7.033912062	2.85E-05	0.000403853
RNF180	-7.03473725	2.55E-06	5.14E-05
CRABP2	-7.03489282	2.34E-12	1.16E-10
DGAT2	-7.036432401	6.40E-06	0.000115416
SMPDL3A	-7.03906106	2.48E-05	0.000362743
VMA21	-7.040301941	0.000239153	0.002075062
ZNF630	-7.040311355	8.18E-07	1.91E-05
DPP3	-7.041976387	1.06E-06	2.42E-05
PRTG	-7.044625959	0.000281634	0.00234611
PIP4K2B	-7.044872235	1.90E-07	5.30E-06
SNRPA1	-7.045061854	5.63E-06	0.000102992
EXD2	-7.047243309	6.01E-08	1.84E-06
TRMT1L	-7.048946162	2.51E-05	0.000366534
IFI27	-7.049231544	3.89E-05	0.000512103
COX7A2	-7.049398425	3.14E-06	6.15E-05
TMEM167A	-7.050601516	0.003575544	0.016487005
IGLV1-40	-7.050874342	0.001515892	0.008486426
TWSG1	-7.052094348	9.31E-06	0.000158477
PA2G4P4	-7.052277	2.49E-05	0.000363382
RAB13	-7.053310151	3.40E-05	0.000463738
OXSR1	-7.055874736	6.45E-07	1.59E-05
DIABLO	-7.059558953	0.000584274	0.004071176
SOAT1	-7.060986577	1.30E-06	2.87E-05
TBC1D3L	-7.061834005	0.000898002	0.005674182
TUBE1	-7.063157512	0.000166147	0.001565011
PTDSS2	-7.066174762	7.59E-07	1.81E-05
ZNF480	-7.073103596	7.41E-08	2.23E-06
CARF	-7.073343964	1.40E-11	6.56E-10
COPS6	-7.073850417	3.01E-07	8.03E-06
INO80B	-7.074280557	6.22E-05	0.000747229
SNRPA	-7.078057769	8.06E-10	3.37E-08
ITGAL	-7.084328027	0.00098361	0.006092945
UQCC2	-7.085339528	6.16E-05	0.000740657
OXNAD1	-7.087865459	2.67E-08	9.00E-07
RAB5IF	-7.087926335	1.30E-06	2.87E-05
RNF2	-7.088375712	3.64E-06	7.01E-05
RALB	-7.088941186	8.01E-08	2.39E-06

MAP2K7	-7.092171052	2.15E-06	4.45E-05
ZNF774	-7.096712562	4.97E-05	0.000618468
E2F5	-7.099207678	1.16E-06	2.62E-05
SERF1B	-7.099416426	0.000262563	0.002228651
SLC38A5	-7.104409595	2.85E-06	5.69E-05
PRODH	-7.104734288	0.002453201	0.012310756
PUS1	-7.106813255	2.21E-06	4.53E-05
ING3	-7.10990909	0.000666903	0.004519707
RALGAPA1P1	-7.111623267	4.29E-06	8.10E-05
RBMS1	-7.112726192	1.03E-07	3.04E-06
TBC1D8	-7.115082994	2.96E-08	9.78E-07
FAM135A	-7.11617106	3.68E-09	1.42E-07
PNP	-7.119399744	5.93E-05	0.000721565
DAGLA	-7.120348152	0.000303804	0.002482947
CHN2	-7.122800736	1.05E-06	2.40E-05
ACTR1A	-7.12326292	3.09E-08	1.02E-06
FMNL2	-7.132861727	4.52E-05	0.000576749
CAD	-7.132885464	1.28E-07	3.66E-06
G3BP1	-7.133026573	0.000107641	0.001120279
RBM34	-7.135572014	4.46E-08	1.40E-06
FAM149B1	-7.136835794	2.63E-08	8.89E-07
PNRC2	-7.13949472	1.48E-11	6.93E-10
TAPBP	-7.140983983	3.82E-10	1.65E-08
RPAP1	-7.150723516	1.04E-05	0.00017367
EFCAB2	-7.152602338	3.51E-06	6.77E-05
INPP5B	-7.155794068	5.65E-08	1.75E-06
CYP51A1	-7.165410978	6.26E-08	1.91E-06
RPA1	-7.165506514	1.08E-06	2.45E-05
CEP68	-7.175788445	5.48E-09	2.07E-07
HLF	-7.177741651	0.000103896	0.001088798
TTC26	-7.177919179	1.09E-09	4.48E-08
CYRIA	-7.178412943	7.92E-09	2.91E-07
MLYCD	-7.181297052	1.25E-07	3.58E-06
CCDC93	-7.182942945	1.72E-08	5.97E-07
BANF1	-7.188840538	8.09E-11	3.63E-09
PLCXD1	-7.189124912	1.21E-06	2.71E-05
ATG4A	-7.189579205	0.000117467	0.001202899
CLCNKA	-7.192129884	0.001453412	0.008232146
FANCG	-7.194170053	4.41E-05	0.000564497
ZNF496	-7.19738906	9.58E-10	3.97E-08
ABCE1	-7.201992251	9.29E-09	3.33E-07
IL1RAP	-7.203474168	4.96E-09	1.89E-07
COPS7A	-7.204136583	3.47E-06	6.71E-05
NCK1	-7.20420627	8.09E-09	2.96E-07

RAB5B	-7.212363262	4.03E-11	1.83E-09
MMP9	-7.213840374	0.000327599	0.00262865
ENSG00000278019	-7.215860419	0.002365318	0.011978998
CALU	-7.223286932	9.17E-05	0.00100059
PWWP3A	-7.223818554	8.16E-11	3.65E-09
TRPC6	-7.224687539	7.83E-06	0.00013658
SEC61A2	-7.233241367	1.11E-05	0.000183422
RPL36A-HNRNPH2	-7.236232261	0.000148067	0.001440799
ZNF326	-7.236335342	1.14E-07	3.32E-06
DHCR7	-7.23893493	4.65E-06	8.72E-05
SLC41A3	-7.243000532	2.95E-08	9.78E-07
FER	-7.246968324	3.69E-08	1.18E-06
SPECC1L-ADORA2A	-7.24780107	0.000346711	0.002730376
CASP9	-7.262232391	8.04E-07	1.89E-05
MRPL20	-7.271229439	1.26E-07	3.60E-06
CERCAM	-7.271855635	7.45E-07	1.80E-05
NDUFV3	-7.273176573	5.43E-08	1.68E-06
IPO8	-7.277294843	4.23E-11	1.91E-09
ZNF568	-7.282498135	2.09E-08	7.18E-07
TMEM14B	-7.287941202	8.27E-05	0.000928212
SLC16A2	-7.302949884	7.16E-07	1.74E-05
BCKDHB	-7.303183795	7.96E-05	0.00090428
NCAM1	-7.303853216	8.68E-07	2.01E-05
MMAB	-7.304147621	0.000181114	0.001667078
STON2	-7.304257247	3.17E-08	1.04E-06
TADA2A	-7.304273372	5.59E-07	1.40E-05
CCT4	-7.306764839	4.86E-10	2.07E-08
FGGY	-7.309986316	5.49E-06	0.000100896
CDKL5	-7.32389072	6.28E-08	1.91E-06
GOLM1	-7.33202778	6.90E-06	0.00012268
LMBR1L	-7.332848255	3.53E-07	9.32E-06
FAM76B	-7.333493439	3.11E-06	6.14E-05
IL6R	-7.334906455	4.92E-05	0.000615975
RSPRY1	-7.339011464	2.79E-11	1.28E-09
SUMO1	-7.34422306	6.53E-09	2.43E-07
DCBLD1	-7.347694458	6.22E-06	0.000112615
MRPS15	-7.348741162	0.005358882	0.022727368
PIP4K2C	-7.35178863	1.27E-05	0.000207625
CAPN3	-7.353572172	0.000349772	0.002741991
MEMO1	-7.357103735	8.01E-07	1.89E-05
GOLGA6L9	-7.364489277	0.000436982	0.003233023
BIRC3	-7.365844261	0.000386363	0.002944922
PARP1	-7.372754186	2.80E-08	9.37E-07
ZNF808	-7.377754146	1.76E-05	0.000269986

ENSG00000283515	-7.382051935	1.40E-09	5.67E-08
BCO2	-7.383236933	8.96E-06	0.000153365
ENSG00000205236	-7.38412297	0.000506268	0.003625954
PAFAH2	-7.384766499	1.24E-07	3.58E-06
DIP2C	-7.385672744	5.71E-09	2.14E-07
TMEM185A	-7.394031197	9.06E-09	3.26E-07
DCLK2	-7.399938414	4.34E-06	8.16E-05
MCM2	-7.410073925	9.28E-05	0.001007633
ENPP2	-7.410778295	4.15E-07	1.07E-05
COMMD10	-7.413352634	5.97E-07	1.48E-05
SRGAP2D	-7.41847055	8.70E-05	0.000962919
KPNA2P3	-7.42123607	2.93E-06	5.81E-05
TRIP4	-7.421809259	5.91E-07	1.47E-05
HSP90AB1	-7.430709572	0.003769211	0.017144882
PARG	-7.433348003	2.06E-06	4.27E-05
TRIB2	-7.438360826	1.13E-06	2.55E-05
EFR3B	-7.440534698	7.32E-06	0.000129236
BRCA1	-7.440910462	4.33E-09	1.66E-07
AIMP1	-7.448937017	7.49E-07	1.80E-05
GPATCH2L	-7.450764702	2.66E-09	1.05E-07
FAM153A	-7.451344259	0.000429681	0.003196936
NSFP1	-7.455895976	9.64E-05	0.00103234
PRUNE1	-7.464472397	2.81E-07	7.60E-06
GOLGA6L17P	-7.469646911	2.45E-07	6.74E-06
IGHV3-30	-7.470957073	0.000411458	0.003091427
UVRAG	-7.474451649	3.94E-07	1.03E-05
FBXO42	-7.483424912	5.45E-13	3.04E-11
CAMSAP3	-7.485767032	1.06E-05	0.000176777
IGHGP	-7.49589977	0.000307995	0.002508727
EML6	-7.498927761	3.27E-09	1.27E-07
CEP83	-7.511966137	1.60E-11	7.44E-10
MAOA	-7.517696788	6.59E-07	1.62E-05
PICK1	-7.5180897	2.94E-05	0.000414951
TRNT1	-7.520116792	1.43E-06	3.10E-05
HFE	-7.522753985	5.20E-07	1.31E-05
RAB11A	-7.524110216	1.10E-09	4.51E-08
DCXR	-7.525855899	4.36E-05	0.000559509
RPGR	-7.526966828	9.46E-08	2.79E-06
PHLPP1	-7.531375954	1.91E-06	4.02E-05
INPP4B	-7.540552722	3.20E-08	1.04E-06
TRAK2	-7.540602865	1.89E-05	0.000288937
CCDC144A	-7.541718711	0.000466248	0.003403809
METTL15	-7.542395793	4.13E-08	1.31E-06
VANGL1	-7.545479959	1.11E-05	0.000183422

MTRR	-7.54731958	5.89E-08	1.81E-06
MOCS2	-7.547958907	2.45E-08	8.34E-07
CAMK1D	-7.553126582	3.60E-07	9.49E-06
ZBTB43	-7.553175548	1.47E-09	5.95E-08
HLA-DQA1	-7.556844164	0.001495903	0.008417673
SHISA5P2	-7.558052738	4.67E-07	1.19E-05
WWOX	-7.559836693	3.13E-06	6.15E-05
MTERF2	-7.560690146	1.95E-12	9.94E-11
KCNMA1	-7.561371818	3.97E-07	1.04E-05
SRPRB	-7.561720464	6.32E-08	1.92E-06
CHI3L2	-7.568777757	0.000136144	0.001350833
GPS1	-7.582482106	3.72E-11	1.70E-09
NAIP	-7.583887084	1.84E-06	3.87E-05
APPBP2	-7.587127314	2.15E-07	5.98E-06
NPHP3-ACAD11	-7.589292853	4.83E-06	9.01E-05
NSFP1	-7.595971165	1.10E-05	0.000181766
PCDHB14	-7.610353423	4.10E-05	0.000533109
ARIH1	-7.622923901	2.32E-09	9.23E-08
NUDT4	-7.623527688	6.75E-08	2.04E-06
CAP2	-7.631392466	6.34E-06	0.000114545
LMLN	-7.632698686	3.12E-06	6.14E-05
AKR7A2	-7.632920831	2.06E-05	0.000309758
TECRP1	-7.636089714	0.000969864	0.006035594
GABBR1	-7.636503819	9.02E-06	0.000154149
ATG7	-7.641891226	2.13E-11	9.86E-10
DAB2	-7.653470551	2.64E-11	1.22E-09
FAM204A	-7.660501557	2.87E-07	7.76E-06
UBE2G2	-7.663576022	3.10E-08	1.02E-06
SYK	-7.676792117	1.54E-10	6.81E-09
VPS26B	-7.677979737	2.98E-07	7.99E-06
ENSG00000250461	-7.679297734	1.49E-06	3.23E-05
IGKV1D-39	-7.680801214	0.008993981	0.033698278
SDCBP	-7.687118523	1.61E-14	1.19E-12
METTL13	-7.688796477	2.01E-06	4.19E-05
MKKS	-7.700139341	5.75E-08	1.77E-06
RYBP	-7.701134232	4.42E-08	1.39E-06
ENSG00000262633	-7.704915865	8.49E-09	3.09E-07
COLGALT1	-7.706243893	1.27E-06	2.83E-05
FCGR2A	-7.706766369	2.74E-06	5.48E-05
EPB41L3	-7.708201661	1.17E-06	2.62E-05
DNAJC2	-7.711958813	1.49E-09	6.03E-08
MTA2	-7.717173754	5.46E-12	2.61E-10
USP14	-7.72584661	5.61E-10	2.37E-08
MLLT3	-7.739489503	4.98E-09	1.89E-07

SPATA6	-7.742043905	2.46E-10	1.08E-08
AHSA1	-7.751240298	1.31E-08	4.60E-07
RRN3P2	-7.768262086	7.10E-07	1.73E-05
RAB3GAP2	-7.780928104	4.57E-09	1.74E-07
VHL	-7.782227311	7.61E-07	1.82E-05
IGLV3-21	-7.782748414	6.70E-05	0.000793732
ZNF440	-7.786627636	1.44E-05	0.000228831
ADAMTS13	-7.790999626	3.62E-08	1.16E-06
RAB11FIP4	-7.791869065	1.13E-12	5.96E-11
BTN3A2	-7.795229151	6.76E-07	1.66E-05
MTAP	-7.796122943	2.19E-07	6.07E-06
CLCN5	-7.807062361	3.16E-05	0.000438339
PRKRA	-7.816474784	1.35E-06	2.97E-05
RBM8A	-7.819435503	1.30E-06	2.87E-05
RAI14	-7.82580119	1.15E-07	3.35E-06
TAF10	-7.835609511	7.50E-07	1.80E-05
MPHOSPH10	-7.84130289	0.000219472	0.001941414
ENSG00000241057	-7.841656248	0.000479619	0.003478316
UBA6	-7.843325413	2.54E-12	1.25E-10
CD59	-7.844015265	6.00E-05	0.000727567
EBAG9	-7.844028219	5.23E-08	1.63E-06
ARL8B	-7.848217446	1.81E-10	7.96E-09
ENSG00000277802	-7.86386914	0.005708344	0.023743574
ACOX2	-7.864183966	5.05E-09	1.91E-07
ENSG00000234353	-7.868423407	3.19E-06	6.24E-05
CD163L1	-7.870439879	0.000920268	0.005792474
ZNF826P	-7.879114881	4.36E-07	1.11E-05
RNASET2	-7.882957288	2.34E-07	6.48E-06
UTP14A	-7.88460868	2.98E-05	0.000418554
RAB18	-7.889801722	2.57E-07	7.04E-06
ZNF135	-7.890545356	2.64E-10	1.15E-08
GTF2H2C_2	-7.894725595	1.27E-08	4.48E-07
NPTN	-7.894959903	2.70E-07	7.35E-06
CASP8	-7.900461224	1.86E-13	1.16E-11
CDH6	-7.902957106	1.36E-07	3.87E-06
PHBP19	-7.903001322	3.03E-07	8.06E-06
ZNF98	-7.94791889	8.66E-07	2.01E-05
NGLY1	-7.949184583	1.74E-16	1.73E-14
SP3	-7.962851565	3.87E-06	7.36E-05
TAS2R31	-7.969336744	4.16E-07	1.07E-05
GGTLC3	-7.970393544	0.000360919	0.002800365
TXNDC5	-7.970810966	2.03E-12	1.03E-10
ACTR3C	-7.971811452	0.000230768	0.002020754
C17orf49	-7.979100695	5.72E-07	1.43E-05

IGFBP6	-7.980751115	2.44E-07	6.74E-06
IFT43	-7.98100132	3.13E-08	1.02E-06
BTN2A3P	-7.982863622	6.87E-06	0.00012233
TRAF3	-7.984337788	3.21E-07	8.52E-06
INTS13	-7.98822107	9.40E-11	4.18E-09
DLAT	-7.992423873	1.37E-05	0.000220275
ALG9	-7.995168269	8.88E-09	3.21E-07
RABGGTB	-8.001020891	3.02E-12	1.47E-10
PCDHA12	-8.002662602	2.62E-05	0.000378503
CLN8	-8.012852926	7.08E-07	1.73E-05
FANCI	-8.016535381	1.21E-11	5.70E-10
TMCO4	-8.018997324	6.84E-10	2.87E-08
RNASEH2B	-8.02812328	2.45E-05	0.000359278
ALDH6A1	-8.028880602	2.80E-08	9.37E-07
NUP153	-8.037414678	3.90E-06	7.41E-05
IGHV3-48	-8.043049342	0.000129042	0.001299435
HLA-DPB1	-8.046365159	0.000219169	0.001941359
MS4A1	-8.064776151	0.000151219	0.001465455
NEK1	-8.072962091	3.42E-13	2.01E-11
CAMK2D	-8.07629257	1.22E-11	5.75E-10
FOXRED1	-8.077700087	4.03E-07	1.05E-05
SMAD1	-8.07920639	6.09E-07	1.51E-05
PPIAP40	-8.082845422	5.59E-07	1.40E-05
LRSAM1	-8.089753535	3.86E-07	1.01E-05
GK3P	-8.094062032	1.46E-05	0.000230765
FBXO25	-8.104301076	7.63E-09	2.82E-07
ZNF208	-8.121755063	6.39E-05	0.000763938
PDLIM3	-8.13363127	7.98E-07	1.89E-05
PTPRC	-8.140667962	1.30E-05	0.000211398
NUS1P1	-8.143360892	2.34E-09	9.30E-08
FLI1	-8.169013983	9.27E-10	3.85E-08
CACHD1	-8.176171618	3.25E-05	0.000445775
PTP4A3	-8.185929115	8.66E-09	3.14E-07
TMEM106C	-8.211839103	3.44E-10	1.49E-08
FAN1	-8.223042451	0.000466533	0.003403809
NOTCH2NLA	-8.260298044	6.05E-08	1.85E-06
FGF7P6	-8.295635256	2.83E-08	9.45E-07
MDH1	-8.318483195	4.14E-14	2.98E-12
FAH	-8.320907894	8.01E-07	1.89E-05
KIF2A	-8.3259142	2.26E-11	1.04E-09
RYR1	-8.32833745	1.07E-05	0.000177394
BCKDHA	-8.337016519	1.51E-07	4.27E-06
NADK	-8.371933767	1.58E-07	4.45E-06
RUNX2	-8.387934973	5.18E-10	2.20E-08

IGLV1-51	-8.428135026	7.97E-07	1.89E-05
ZNF69	-8.450847929	0.001147448	0.006872198
ZNF248	-8.452245995	3.20E-12	1.54E-10
GSR	-8.458821751	4.19E-09	1.61E-07
CHRNA7	-8.459863529	4.09E-07	1.06E-05
ZNF44	-8.46568408	8.16E-09	2.98E-07
DGKH	-8.475608491	2.93E-15	2.27E-13
TPTEP2-CSNK1E	-8.536869698	0.00066958	0.00453277
CENPJ	-8.557147779	0.000221384	0.001953086
BCL2L2-PABPN1	-8.563563972	7.72E-06	0.000134969
ENSG00000282034	-8.578630714	0.000476041	0.003458587
ANTXR1	-8.596848417	6.83E-12	3.24E-10
SLC37A1	-8.599851685	1.25E-09	5.12E-08
JAM3	-8.615213638	2.67E-09	1.05E-07
GABBR2	-8.634705003	4.56E-10	1.95E-08
BTN2A1	-8.641436976	1.61E-10	7.10E-09
JCHAIN	-8.655856033	7.92E-07	1.88E-05
IGHV3-23	-8.656492021	0.000268578	0.002263835
GPI	-8.665405034	0.000237981	0.002067855
C4B	-8.673523042	0.000387548	0.002946535
WASH9P	-8.70286082	1.72E-08	5.97E-07
IGHV3-11	-8.716515686	2.19E-06	4.51E-05
TRAM1	-8.722302157	9.79E-25	3.20E-22
TULP4	-8.778220354	4.20E-13	2.41E-11
WDR70	-8.805524919	1.29E-09	5.26E-08
HNRNPA1P48	-8.813801381	2.58E-08	8.76E-07
RPP14	-8.828541424	2.78E-07	7.54E-06
FMNL3	-8.843945211	4.21E-15	3.22E-13
ENAH	-9.003434719	1.04E-13	6.88E-12
BMS1P15	-9.006438162	2.32E-05	0.000343212
IGKV3-11	-9.049497839	0.000172053	0.001603178
NPIPA1	-9.165596075	2.65E-16	2.43E-14
PMS2P6	-9.169864735	7.74E-08	2.31E-06
NUP85	-9.19949465	4.29E-14	3.08E-12
RNF128	-9.263528857	3.34E-05	0.000456368
GTF2IP12	-9.283603834	5.80E-14	4.10E-12
GOLGA6L9	-9.354835389	3.28E-06	6.38E-05
RPE	-9.478416894	1.96E-12	9.98E-11
RPL9	-9.509708446	5.96E-09	2.22E-07
AGAP12P	-9.583822291	3.63E-07	9.56E-06
TBC1D3I	-9.769616107	0.00022645	0.001989075
IGHV3-7	-9.850375515	2.00E-05	0.000301839
FAM86B1	-10.06050365	3.77E-06	7.20E-05
WASH7P	-10.54106027	5.12E-08	1.60E-06

AGAP10P	-10.84845473	3.69E-09	1.42E-07
IGKV1-16	-10.97410754	5.30E-07	1.34E-05
IGLC3	-11.37656039	2.39E-12	1.18E-10
C4B	-11.40561144	1.73E-08	6.00E-07
TEAD1	-11.51472184	6.03E-12	2.87E-10
IGKV1-5	-12.89978581	2.38E-09	9.41E-08
ABHD16A	-18.64551523	2.58E-10	1.13E-08
FAM86JP	-19.31214319	2.97E-12	1.45E-10
MGMT	-19.91809952	6.04E-13	3.34E-11
PHGDH	-20.22964612	3.47E-13	2.02E-11
IGHV1-46	-20.57593069	3.03E-12	1.47E-10
DENND10P1	-20.64470202	2.56E-12	1.26E-10
TBC1D3I	-20.64934927	2.53E-12	1.25E-10
GOLGA8Q	-20.68855227	2.30E-12	1.15E-10
IGKV1-8	-20.69949017	2.24E-12	1.13E-10
IGHV7-4-1	-20.72186452	2.12E-12	1.07E-10
LRP1B	-20.78972374	1.78E-12	9.16E-11
FAM86GP	-20.79032461	1.79E-12	9.19E-11
PPIAP19	-20.82123629	1.66E-12	8.59E-11
MSN	-20.85698592	1.52E-12	7.89E-11
IGKV2-28	-20.86831583	1.48E-12	7.71E-11
FLG2	-20.95367226	1.20E-12	6.27E-11
SRGAP2C	-20.95459747	1.19E-12	6.27E-11
ENSG00000278066	-21.02953815	9.92E-13	5.27E-11
SPDYE2B	-21.03726588	9.57E-13	5.10E-11
IGKV2D-30	-21.04365351	9.57E-13	5.10E-11
USP17L3	-21.08275617	8.69E-13	4.67E-11
C2	-21.0870009	8.59E-13	4.64E-11
ENSG00000260537	-21.08752599	8.57E-13	4.64E-11
SFRP1	-21.09571931	8.41E-13	4.59E-11
GGTLC5P	-21.14052215	7.52E-13	4.12E-11
IGHV4-34	-21.19802181	6.51E-13	3.58E-11
NCF1C	-21.21532589	9.71E-15	7.26E-13
NOL4	-21.24738991	5.76E-13	3.20E-11
HLA-DPB1	-21.31797427	4.82E-13	2.71E-11
STRIP2	-21.33518498	4.62E-13	2.61E-11
IGKV1D-12	-21.34750332	4.48E-13	2.54E-11
CNTNAP3P2	-21.35872637	4.35E-13	2.48E-11
CYP21A1P	-21.42638529	3.67E-13	2.11E-11
ZNF804B	-21.42792326	3.65E-13	2.11E-11
ALOX5	-21.45286654	3.43E-13	2.01E-11
TNXA	-21.45563051	3.41E-13	2.01E-11
DDT	-21.45723399	3.39E-13	2.01E-11
PKP2	-21.47524942	3.21E-13	1.92E-11

CETP	-21.50062531	3.04E-13	1.82E-11
PSG1	-21.50529186	3.00E-13	1.81E-11
SH3BGR	-21.50644985	2.99E-13	1.81E-11
RGL1	-21.51831026	2.91E-13	1.77E-11
TNFRSF11A	-21.52072791	2.89E-13	1.77E-11
IGHV4-39	-21.55117248	2.67E-13	1.64E-11
ENSG00000284128	-21.57637472	2.51E-13	1.55E-11
SLC7A5	-21.59980356	2.37E-13	1.47E-11
BBOF1	-21.63271449	3.00E-20	5.01E-18
BAG6	-21.65135042	9.02E-18	1.11E-15
IGKV1-6	-21.69349466	1.86E-13	1.16E-11
COLEC11	-21.69566722	1.85E-13	1.16E-11
HLA-DRB1	-21.69768065	4.73E-15	3.60E-13
MRPS9	-21.69845674	1.84E-13	1.16E-11
PPIAP26	-21.73841686	1.66E-13	1.06E-11
USP17L1	-21.80288696	1.41E-13	9.05E-12
IGHV2-5	-21.80304539	1.41E-13	9.05E-12
FAM90A1	-21.84716343	1.26E-13	8.17E-12
LRRC37A	-21.89599636	1.11E-13	7.24E-12
HLA-DQA2	-21.89764834	1.10E-13	7.24E-12
DNAH2	-21.92903609	1.30E-15	1.06E-13
CLDN16	-21.95376486	9.54E-14	6.34E-12
ENSG00000255556	-21.9715074	9.11E-14	6.09E-12
PLP1	-21.97721543	8.99E-14	6.04E-12
IGLL5	-21.98593836	8.78E-14	5.97E-12
ENSG00000277125	-22.00881461	8.27E-14	5.66E-12
IGHV3-7	-22.01765955	8.09E-14	5.56E-12
LMOD1	-22.02062446	8.02E-14	5.55E-12
ABCB1	-22.02431652	8.04E-16	6.76E-14
EXO1	-22.0681102	7.09E-14	4.93E-12
C19orf33	-22.07166323	7.03E-14	4.92E-12
IGHV3-20	-22.08384105	6.81E-14	4.79E-12
ANKRD20A4P	-22.12573758	1.30E-15	1.06E-13
NOSTRIN	-22.19291277	1.06E-15	8.80E-14
IGKV1D-13	-22.23511542	1.92E-15	1.54E-13
AKR1E2	-22.26983382	2.23E-18	2.94E-16
PRDX1	-22.29007239	3.98E-14	2.88E-12
MAMLD1	-22.29124242	6.28E-16	5.35E-14
ENSG00000266997	-22.29451734	1.43E-15	1.16E-13
KIF17	-22.3015739	2.44E-16	2.29E-14
CFAP47	-22.30292935	2.39E-16	2.26E-14
CD3E	-22.30407994	7.27E-16	6.15E-14
IGF1	-22.31543401	2.93E-16	2.67E-14
MYO15A	-22.36342152	2.64E-16	2.43E-14

AURKC	-22.37555232	3.02E-16	2.71E-14
TSEN54	-22.39389154	1.79E-16	1.76E-14
IGKV3D-20	-22.4079699	2.92E-14	2.13E-12
PCDHA2	-22.40982766	1.38E-15	1.12E-13
CYP2S1	-22.41909958	1.82E-16	1.78E-14
SAMD8	-22.42429151	3.61E-22	8.91E-20
EEF1B2P1	-22.45994713	2.55E-16	2.38E-14
NCF4	-22.47074839	1.89E-16	1.82E-14
PNMA6B	-22.52183226	8.88E-23	2.34E-20
LSS	-22.53589922	1.74E-19	2.45E-17
IGKV1OR2-1	-22.56941509	1.31E-16	1.38E-14
TSEN34	-22.58575741	2.35E-16	2.24E-14
IGKV2-30	-22.59757733	1.47E-16	1.49E-14
ENSG00000249624	-22.61803549	1.46E-19	2.13E-17
SNX29P2	-22.66183542	5.01E-16	4.33E-14
IGKV1D-16	-22.67378193	8.36E-19	1.12E-16
VAMP5	-22.69824877	5.00E-16	4.33E-14
CDADC1	-22.69985496	9.40E-20	1.41E-17
USP17L4	-22.72422044	1.26E-14	9.39E-13
CD274	-22.74617237	1.85E-16	1.79E-14
ENSG00000254979	-22.74855837	2.01E-19	2.79E-17
AMY1A	-22.75374511	3.68E-16	3.22E-14
ENSG00000256210	-22.75382713	1.38E-16	1.43E-14
GRAP	-22.75400819	8.77E-17	9.74E-15
MIS12	-22.76033032	1.55E-19	2.23E-17
HSPE1-MOB4	-22.80017098	1.05E-16	1.13E-14
FAM90A2P	-22.80620611	1.86E-21	4.09E-19
KDELR3	-22.83465212	7.44E-20	1.13E-17
FAM106A	-22.8347046	7.95E-17	8.99E-15
TMTC1	-22.85830888	8.04E-23	2.21E-20
KLRC4-KLRK1	-22.85928253	1.12E-16	1.19E-14
SRGN	-22.88970173	2.90E-19	3.94E-17
IGHV1-3	-22.91871233	8.70E-23	2.34E-20
FAM90A20P	-22.92923255	9.63E-17	1.06E-14
ACOT2	-22.93273	1.02E-16	1.11E-14
NAMPT	-22.94123311	5.20E-20	8.28E-18
NSFP1	-22.9505605	2.01E-22	5.07E-20
UBASH3B	-22.99177737	1.24E-19	1.83E-17
IKBKG	-23.00080392	2.43E-23	6.84E-21
PGA3	-23.0182236	5.75E-15	4.32E-13
NUTM2E	-23.03816632	8.31E-17	9.32E-15
RPSAP58	-23.04891258	6.31E-20	9.80E-18
TWNK	-23.06194904	3.62E-20	5.84E-18
PGAM1	-23.08963628	1.74E-23	5.02E-21

IGHV5-51	-23.09237488	1.73E-16	1.73E-14
TAS2R31	-23.10418532	2.06E-20	3.56E-18
TUBBP5	-23.12249767	9.90E-17	1.08E-14
CD84	-23.14054129	1.63E-19	2.32E-17
USP17L30	-23.16208398	3.89E-15	3.00E-13
IGHV3-53	-23.18750841	1.01E-15	8.43E-14
ENSG00000249319	-23.23677532	7.26E-21	1.46E-18
STAG3L1	-23.23998406	1.05E-20	1.93E-18
CKMT1A	-23.2470531	5.17E-17	6.02E-15
IGKV1D-33	-23.26792668	2.92E-15	2.27E-13
C1orf21	-23.28629855	1.82E-20	3.20E-18
IGHV3-73	-23.3048948	3.01E-16	2.71E-14
KIFAP3	-23.30764718	7.00E-20	1.07E-17
TBC1D3L	-23.31469111	2.57E-15	2.02E-13
ENSG00000280852	-23.31622273	1.28E-20	2.31E-18
ENSG00000281561	-23.31988264	5.48E-20	8.62E-18
AMY1C	-23.32639059	4.11E-22	9.95E-20
IGHD	-23.35557031	3.56E-20	5.83E-18
MT1X	-23.36283655	1.32E-20	2.34E-18
APOE	-23.38124155	5.72E-24	1.73E-21
GPC6	-23.38414739	2.13E-15	1.69E-13
RAB6B	-23.40014968	3.18E-27	1.84E-24
ENSG00000233170	-23.55209756	8.19E-21	1.60E-18
NOTCH4	-23.57765393	8.55E-21	1.64E-18
ENSG00000161103	-23.62627358	1.63E-16	1.64E-14
RPS26P11	-23.65689296	3.72E-27	2.05E-24
ENSG00000263020	-23.66335064	1.25E-27	7.54E-25
SIGLEC10	-23.6798181	2.35E-28	1.77E-25
NTM	-23.69360332	2.94E-25	1.11E-22
ZNF658B	-23.70979752	4.52E-31	6.07E-28
COL11A1	-23.71409019	1.62E-23	4.77E-21
TYW1B	-23.78663684	1.31E-24	4.18E-22
FADS2	-23.81032213	5.58E-29	5.19E-26
HLA-DRB1	-23.84451033	5.95E-16	5.11E-14
TNXB	-23.8518348	3.02E-20	5.01E-18
SERF1A	-23.86714632	6.68E-27	3.26E-24
ALS2	-23.89531231	2.57E-20	4.37E-18
PPIAP51	-23.92526559	8.84E-25	2.97E-22
KRT87P	-23.95726449	7.92E-26	3.55E-23
CCL4L2	-23.967578	2.34E-24	7.27E-22
TNXB	-24.02448252	2.81E-21	5.96E-19
IGHV1-46	-24.03113863	3.16E-17	3.79E-15
IMPDH1	-24.04931068	3.86E-29	3.89E-26
GOLGA8O	-24.07730722	7.92E-17	8.99E-15

IGHV4-39	-24.08429947	3.04E-16	2.71E-14
SLX4	-24.11866389	4.62E-28	3.11E-25
FRMPD2B	-24.15767457	8.21E-26	3.55E-23
IGHV3-13	-24.18594312	4.04E-21	8.44E-19
CEP63	-24.19137289	1.19E-21	2.73E-19
NT5C3A	-24.22169473	2.81E-25	1.10E-22
IGHV3-66	-24.28226077	1.11E-25	4.63E-23
DPY19L2P2	-24.31382293	6.24E-21	1.28E-18
AGAP4	-24.38360667	4.48E-26	2.09E-23
MUC20	-24.43277044	7.11E-29	5.74E-26
ENSG00000198211	-24.47273062	5.51E-25	1.91E-22
TNXB	-24.59148823	1.23E-25	4.97E-23
ZNF225	-24.62401682	3.53E-31	5.34E-28
CASP4	-24.62759827	1.87E-30	2.06E-27
CLCNKB	-24.82118718	7.90E-21	1.57E-18
SMN2	-24.89478293	2.13E-21	4.60E-19
IGKV3-15	-24.93386947	9.89E-21	1.85E-18
USP17L22	-24.94744487	2.57E-17	3.11E-15
IGHV1-69D	-24.94924749	8.83E-22	2.05E-19
MCCC2	-25.00898867	4.41E-25	1.57E-22
ENSG00000267740	-25.02167051	4.15E-25	1.52E-22
IGLV2-8	-25.03158279	3.18E-31	5.34E-28
GBP2	-25.06461801	2.58E-28	1.84E-25
IGHV1-2	-25.20022657	6.55E-29	5.66E-26
IGHV3-15	-25.21093474	1.65E-21	3.70E-19
GOLGA8N	-25.21425166	7.17E-39	2.89E-35
IGHV1-18	-25.26957996	8.61E-18	1.07E-15
IGHV3-74	-25.32055379	6.73E-27	3.26E-24
GOLGA8M	-25.49279606	5.16E-18	6.57E-16
ENSG00000278082	-25.59073233	4.25E-22	1.01E-19
IGHGP	-25.70975925	2.70E-18	3.51E-16
IGKV1-9	-25.85392793	1.12E-27	7.11E-25
SELENON	-26.01930999	9.42E-35	2.85E-31
IGKV1-33	-26.21564108	1.83E-22	4.70E-20
IGKV1-17	-26.34775493	6.60E-27	3.26E-24
IGLV2-23	-27.22971786	1.41E-33	3.42E-30
IGLV2-14	-27.70033901	1.96E-42	2.38E-38
IGKV1-39	-27.80176336	4.23E-33	8.54E-30
LRRC37A3	-28.00707455	3.73E-40	2.26E-36

Supplementary Table-S5: Differentially expressed genes in *Cutibacterium acne* high versus low sub-group of anaplastic thyroid cancer samples (n=30). Padj is p-values, adjusted for multiple testing with the Benjamini-Hochberg procedure.

Gene name	log2FoldChange	pvalue	padj

POTEE	30	7.15E-19	2.06E-16
MYO5BP1	28.6535	2.45E-17	5.00E-15
FAM90A24P	28.43761	4.25E-17	7.71E-15
TRPM1	27.62053	3.35E-16	4.12E-14
GTF2IP20	27.61689	3.37E-16	4.12E-14
REXO1L1P	26.7711	2.69E-15	2.39E-13
ENSG00000257175	26.59888	4.09E-15	3.52E-13
IGSF8	26.30813	8.41E-15	6.86E-13
SPATA31D5P	26.04483	1.55E-14	1.25E-12
AMPH	25.97162	1.85E-14	1.44E-12
CMTM3	25.94926	1.92E-14	1.47E-12
ENSG00000246203	25.85827	2.41E-14	1.81E-12
AGAP7P	25.63136	4.09E-14	3.04E-12
FAM27E3	25.36934	7.48E-14	5.31E-12
ENSG00000253882	25.22506	1.05E-13	7.34E-12
ENSG00000196656	25.20389	1.11E-13	7.63E-12
LILRB4	25.20196	1.12E-13	7.63E-12
PYROXD1	25.02497	1.69E-13	1.12E-11
UBE2V1P1	25.01316	1.70E-13	1.12E-11
ACAP3	24.96695	1.87E-13	1.22E-11
POM121L9P	24.93659	2.02E-13	1.30E-11
LILRB4	24.90988	2.15E-13	1.37E-11
CCN4	24.78466	2.73E-13	1.72E-11
LILRB4	24.67774	3.62E-13	2.24E-11
ALPL	24.6626	3.79E-13	2.32E-11
CPT1A	24.612	4.30E-13	2.60E-11
PTHLH	24.55325	4.85E-13	2.90E-11
TSC22D2	24.4786	5.61E-13	3.31E-11
H2AZ1	24.44093	5.86E-13	3.42E-11
DIPK2A	24.42619	6.33E-13	3.65E-11
ENSG00000225871	24.34174	7.59E-13	4.33E-11
PLA1A	24.32136	7.90E-13	4.45E-11
PPP2R3A	24.26934	8.83E-13	4.92E-11
RAB15	24.13364	1.20E-12	6.61E-11
EEFSEC	24.13272	1.22E-12	6.63E-11
FAM110B	23.85697	2.19E-12	1.18E-10
GOLGA6L10	14.82497	1.07E-05	0.000568
DUX4L19	12.11562	0.000317	0.014364
OSBPL5	12.01442	0.000353	0.015584
GAPDHP61	11.83179	0.000377	0.016361
PIEZO1	11.80675	0.000358	0.015661
HABP2	11.67335	0.000577	0.023956
FLG2	11.62419	0.000161	0.007641
PKD1P5	11.5426	0.000533	0.022506

FAM205BP	11.39178	0.000757	0.030075
EPS8L1	11.28576	0.000797	0.030902
TRAF2	11.17716	0.000799	0.030902
OR4F7P	10.98756	0.001138	0.041312
ALG2	10.92287	0.000137	0.006706
MCTP2	10.67409	0.001558	0.054517
SUCLA2P1	10.50172	0.001825	0.061245
SUCLA2P1	10.50172	0.001825	0.061245
RPSAP2	10.05529	0.002851	0.086508
SERINC2	10.0405	0.002897	0.086519
PRLR	9.997701	0.002944	0.086519
SHMT1	9.99329	0.001811	0.061245
NOS3	9.972923	0.002804	0.086403
TTC31	9.919204	0.003088	0.089534
DDT	9.808056	0.003355	0.09613
DMBT1	9.786505	0.003693	0.10399
CCDC88B	9.758439	0.002765	0.08574
TFIP11	9.722446	0.004202	0.114999
HAVCR2	9.643832	0.004238	0.114999
SNX29P2	9.640091	0.004078	0.113526
CCNE1	9.637477	0.004257	0.114999
ELP4	9.627316	0.001097	0.040394
ALOX5	9.553998	0.004601	0.121217
ALDH5A1	9.523841	0.004449	0.117833
KLHL29	9.433163	0.004971	0.127527
AHCYL2	9.385253	0.001939	0.06333
ENSG00000286220	9.37374	0.005365	0.136209
MAN1B1	9.366737	0.004861	0.126038
FBXL15	9.360027	0.005517	0.138631
RPL12P4	9.319974	0.005724	0.142045
SYNE4	9.31922	0.005728	0.142045
PPP2R2B	9.229869	0.006109	0.147464
MYO1E	9.220118	0.003235	0.093234
CYP21A1P	9.148513	0.006381	0.148174
TXNDC2	9.111569	0.006915	0.1575
GLI2	9.107992	0.006702	0.154903
KANSL1L	9.072157	0.006358	0.148174
MECR	9.056644	0.007267	0.161837
DNAJC6	9.007113	0.006943	0.1575
WWP1P1	8.997062	0.007667	0.168101
PDPN	8.986976	0.007719	0.168101
FCAR	8.971778	0.007299	0.161837
CHD1L	8.954484	0.007916	0.170506
CEACAM5	8.949587	0.007124	0.159397

WDR81	8.926924	0.00816	0.17087
ENSG00000226268	8.888956	0.008438	0.173007
TFCP2	8.870061	0.006023	0.146102
LYAR	8.861636	0.008145	0.17087
GALNT11	8.838298	0.00883	0.175197
AREG	8.835756	0.007999	0.170506
NETO1	8.818548	0.008977	0.175197
MCMDC2	8.818548	0.008977	0.175197
HDGFL3P1	8.818548	0.008977	0.175197
ZNF75BP	8.818548	0.008977	0.175197
ANKRD20A21P	8.778435	0.009296	0.175197
BBS1	8.777922	0.009286	0.175197
RFC2	8.774425	0.0062	0.148174
WDR17	8.755008	0.009499	0.176308
ENSG00000283963	8.754079	0.006263	0.148174
LRRC66	8.726817	0.009109	0.175197
PSPN	8.716595	0.009809	0.179343
ABHD12	8.71245	0.009955	0.180668
RPL39	8.686652	0.009789	0.179343
SCN11A	8.668882	0.010221	0.181497
CEP131	8.661547	0.010008	0.180948
ADGRB3	8.634772	0.009472	0.176308
SIRPB2	8.616912	0.010682	0.183581
ARID3B	8.614658	0.010715	0.183581
STK31	8.601439	0.010815	0.184641
IQSEC2	8.585444	0.010978	0.185491
ZNF747	8.578195	0.010639	0.183561
PANK4	8.543782	0.008915	0.175197
EVI5	8.535936	0.007939	0.170506
YWHAQP5	8.532824	0.011481	0.18878
SIPA1L2	8.529567	0.011427	0.18878
PRIM1	8.510628	0.011778	0.18908
PEX7	8.499712	0.011808	0.18908
ARMC3	8.499712	0.011808	0.18908
TXN2P1	8.499712	0.011808	0.18908
ENSG00000280171	8.499712	0.011808	0.18908
ADAMTS7	8.498454	0.011172	0.187472
REXO1L6P	8.493668	0.011894	0.189218
GTF2A1	8.479409	0.011996	0.190233
RNASE10	8.477034	0.003418	0.097366
WNT5A	8.462969	0.012148	0.191401
HARS2	8.441385	0.012403	0.194791
COL24A1	8.413605	0.01275	0.195907
ATG12P2	8.404568	0.012794	0.195907

ATRNL1	8.404568	0.012794	0.195907
FOXN4	8.404568	0.012794	0.195907
BCL2L14	8.399181	0.009233	0.175197
ENSG00000218426	8.388633	0.001903	0.062592
CAMK2G	8.350985	0.00221	0.070779
FOXA1	8.336213	0.013547	0.19951
ABO	8.333816	0.013574	0.19951
AKR1B10	8.333816	0.013574	0.19951
USP12P1	8.333816	0.013574	0.19951
BRIP1	8.330021	0.013617	0.19951
DCAF15	8.320631	0.013731	0.19951
PRR23C	8.314945	0.013108	0.19702
ATF6B	8.313077	0.013811	0.19951
CD200R1	8.309887	0.013771	0.19951
FIBCD1	8.300477	0.013956	0.19951
ENSG00000212952	8.300477	0.013956	0.19951
ENSG00000254506	8.300477	0.013956	0.19951
PRAMEF22	8.300477	0.013956	0.19951
PYGO1	8.299263	0.01397	0.19951
CCDC175	8.294355	0.014034	0.19951
RNFT1	8.288543	0.010518	0.183207
ENSG00000278594	8.287634	0.014106	0.199762
TOMM40P2	8.269134	0.014325	0.200754
CDC27P3	8.260306	0.014429	0.200962
TMEM39B	8.259042	0.01434	0.200754
FOXK2	8.256471	0.009643	0.177858
EVC2	8.249417	0.014559	0.200962
TTI2	8.238519	0.01467	0.201349
SHMT2	8.235287	0.014752	0.201705
LRIG3	8.229277	0.009194	0.175197
CFAP20DC	8.218846	0.008692	0.175197
ZNF774	8.208436	0.009046	0.175197
FAM86B1	8.205314	0.014509	0.200962
ALKBH4	8.204518	0.010192	0.181497
CARD9	8.203315	0.015223	NA
POLRMTP1	8.189572	0.015296	0.205344
GOLGA6L10	8.18516	0.01349	0.19951
QSER1	8.17893	0.008006	0.170506
TCAF1	8.156192	0.015734	0.206767
AKR7A2P1	8.154757	0.01574	0.206767
ZRANB3	8.151402	0.012984	0.19702
AMPD3	8.128877	0.006317	0.148174
TESMIN	8.110041	0.016338	0.21123
CCNB3P1	8.085523	0.016656	0.21477

CLUHP3	8.071531	0.016846	NA
GNA13	8.060892	0.016993	NA
HIF3A	8.057124	0.017045	NA
GAD2	8.057124	0.017045	NA
FOXH1	8.057124	0.017045	NA
TTC16	8.057124	0.017045	NA
EIF4A1P5	8.057124	0.017045	NA
ENGASE	8.05697	0.017076	NA
NR4A3	8.047968	0.0169	0.216305
RBM14-RBM4	8.035687	0.017343	NA
SFXN4	8.03567	0.017305	NA
ZC3H12D	8.026732	0.017484	NA
CD74	8.019299	0.015578	0.206527
CCND1	8.017856	0.017593	NA
TRIB3	8.010686	0.017685	0.223338
DNAJC22	7.995348	0.003999	0.111978
KIF24	7.988747	0.015847	0.207072
PPIF	7.987132	0.01804	NA
TDRD10	7.9808	0.018133	NA
IDSP1	7.974683	0.017916	0.225676
TMEM163	7.973707	0.018234	NA
ZHX3	7.969468	0.000691	0.028222
SHC2	7.969173	0.01826	0.228838
ACSS1	7.956702	0.018485	NA
AHI1	7.947117	0.018533	0.230103
LZTR1	7.936474	0.018763	NA
TBXAS1	7.935251	0.018807	0.230103
PPP1R1B	7.935251	0.018807	0.230103
TUBG1	7.932154	0.009194	0.175197
TLE6	7.926265	0.018943	NA
EIF3G	7.923517	0.012658	0.195907
SIGLEC8	7.911583	0.019167	NA
CCK	7.911583	0.019167	NA
BLOC1S3	7.911583	0.019167	NA
RPL17P22	7.911583	0.019167	NA
MFSD6	7.901348	0.015454	0.206527
NDUFAF8	7.897944	0.019379	NA
CPED1	7.888316	0.019501	NA
FAAP100	7.880011	0.019726	NA
CLEC18C	7.878724	0.018418	0.230103
CEACAM22P	7.875767	0.000333	0.014982
ZFAND2B	7.874849	0.002256	0.071323
ENSG00000260404	7.871389	0.012697	0.195907
ABCB5	7.869188	0.019237	0.231606

TBC1D31	7.866154	0.018831	0.230103
MAD2L1P1	7.863015	0.019927	NA
ZNF329	7.846859	0.001633	0.056365
PHF14	7.84497	0.020254	0.236302
HNRNPA1P22	7.839951	0.020293	NA
ERF	7.837736	0.020325	NA
HLA-B	7.834021	0.018699	0.230103
GOLGA8R	7.831779	0.010581	0.183207
HMGB1P39	7.829828	0.020457	NA
RELB	7.823544	0.020529	NA
ADH5	7.820302	0.008038	0.170506
TRAV8-6	7.816598	0.020671	NA
C2CD2	7.816502	0.020638	NA
PRR27	7.814456	0.020706	NA
POLR3H	7.810306	0.020726	NA
HOOK3	7.810032	0.006843	0.157425
AP5S1	7.805478	0.020176	0.236138
ENDOV	7.803188	0.020891	NA
TMPRSS9	7.792917	0.021062	NA
BBS7	7.778993	0.021249	NA
CENPJ	7.776313	0.021342	NA
LILRA6	7.764766	0.021535	NA
AMT	7.759469	0.002949	0.086519
ESRP2	7.74727	0.02211	NA
NOP14	7.744324	0.021844	NA
PDCD6IPP2	7.743906	0.02146	0.245684
ACVRL1	7.742317	0.021663	NA
ENSG00000268434	7.739972	0.021958	NA
PGR	7.738019	0.021522	0.245824
DIAPH3	7.736152	0.022001	NA
GNA12	7.727938	0.022067	NA
ZNF440	7.724855	0.011596	0.18908
PHC1	7.719539	0.02238	NA
MARCHF9	7.707034	0.022655	NA
ZNF554	7.705888	0.000517	0.022036
POLE3	7.698932	0.022563	NA
DNAH5	7.696993	0.022801	0.252768
GASK1B	7.696018	0.023048	NA
ATXN1	7.691339	0.021782	0.247773
BTBD7P1	7.655743	0.023448	NA
LRPAP1	7.643065	0.023672	NA
CCNYL1	7.640812	0.023745	NA
B3GALNT2	7.63678	0.017591	0.222732
KMT5A	7.636762	0.023721	NA

CASTOR1	7.635242	0.023823	NA
DUX4L32	7.635242	0.023823	NA
ATP8B2	7.624142	0.024082	NA
S100A10	7.618374	0.024138	NA
TJP1	7.614536	0.024103	NA
ACADSB	7.61222	0.02448	NA
SEMA4B	7.61082	0.023738	0.259629
ZNF12	7.610186	0.024279	NA
ENSG00000258417	7.610051	0.024292	NA
LRRC61	7.608841	0.024313	0.262632
PARD3B	7.608225	0.022635	0.251856
HNF4A	7.607495	0.024339	0.262632
GPR35	7.607495	0.024339	0.262632
ENSG00000277983	7.607495	0.024339	0.262632
RPF1	7.607495	0.024339	NA
PCGF6	7.607495	0.024339	NA
NLRP4	7.607495	0.024339	NA
EN2	7.607495	0.024339	NA
MSRB1P1	7.607495	0.024339	NA
PTMAP1	7.607495	0.024339	NA
RPS3AP8	7.607495	0.024339	NA
ENSG00000233820	7.607495	0.024339	NA
LONRF2P5	7.607495	0.024339	NA
YBX2	7.599032	0.024499	NA
DRG2	7.596216	0.024504	NA
RASGEF1B	7.571783	0.024045	0.262402
MAT2B	7.569416	0.025204	NA
RWDD4	7.568604	0.025147	NA
CADPS2	7.567446	0.022667	0.251856
BHLHE41	7.567338	0.025123	NA
TRIM27	7.556247	0.025692	NA
PMCHL2	7.553712	0.024514	0.263414
SEMA6C	7.548847	0.024648	0.26428
QPRT	7.542323	0.024893	0.264584
IL31RA	7.542201	0.025553	NA
BAGE2	7.540834	0.02562	NA
LPCAT4	7.53484	0.025689	NA
AXIN2	7.514073	0.023373	0.257411
GLCCI1	7.512508	0.02489	0.264584
SCPEP1	7.50657	0.026208	NA
SELENBP1	7.504147	0.026307	NA
PFKFB4	7.498403	0.026409	NA
RXFP2	7.486563	0.026704	NA
MYO7A	7.486457	0.026665	NA

BTN2A3P	7.481531	0.026716	NA
PTDSS1	7.476569	0.026885	NA
ENSG00000281759	7.475881	0.026923	NA
AIPL1	7.472181	0.008614	0.175197
PMS2	7.471079	0.027001	NA
FRMD8	7.47009	0.027078	NA
ZNF583	7.466483	0.011285	0.188732
DTNA	7.459625	0.027132	NA
PRR19	7.459121	0.000153	0.007339
RARRES2P4	7.455452	0.027344	NA
ST13P5	7.453674	0.026862	NA
SMG1P6	7.45108	0.027435	NA
SNAI2	7.450177	0.027456	NA
COL19A1	7.449825	0.022027	0.24869
HIC1	7.445563	0.027593	NA
PES1	7.444906	0.02765	NA
TXNL1	7.444047	0.027502	NA
MERTK	7.438673	0.02779	NA
TBCK	7.431152	0.027607	NA
ENSG00000233406	7.414881	0.021929	NA
PTTG3P	7.413151	0.028236	NA
NAGS	7.411982	0.027245	0.283437
TIMM13	7.411121	0.027481	0.283885
DOK1	7.410872	0.027417	0.283885
BUD31	7.408109	0.014778	0.201705
RER1	7.406318	0.011395	0.18878
NUBP1	7.406206	0.026973	0.281245
SRGAP3	7.403065	0.028439	NA
POU2F2	7.393979	0.02841	NA
CENPNP1	7.39165	0.028462	NA
C5orf22	7.387465	0.028998	NA
HYLS1	7.385477	0.028832	NA
RNF149	7.378613	0.028956	NA
DCHS2	7.376649	0.029025	NA
GRAMD4P3	7.374052	0.029081	NA
SCRN2	7.372417	0.029137	NA
MRPL2	7.370535	0.029189	NA
CSPG4P13	7.369652	0.029059	NA
NLGN1	7.368058	0.029053	NA
MRPL30	7.365546	0.0293	NA
KCND3	7.362502	0.029452	NA
GPR173	7.358982	0.029409	NA
SMIM29	7.35827	0.029428	NA
CMSS1	7.355985	0.011524	NA

JPT2	7.352789	0.019224	0.231606
ZBTB7A	7.351677	0.029576	NA
CD47	7.348926	0.028689	0.289511
ANKRD29	7.348159	0.029447	NA
CCDC18	7.34569	0.029454	NA
RCN3	7.338882	0.029863	NA
APC2	7.331418	0.023435	NA
CSTB	7.33066	0.030048	0.295994
ITFG2	7.328891	0.029932	NA
TSPOAP1	7.328457	0.030453	NA
TMUB1	7.327498	0.029426	0.292554
HLA-A	7.327035	0.030121	0.295994
SNX22	7.323352	0.010968	0.185491
LUZP2	7.322137	0.030237	0.295994
LTB4R2	7.322137	0.030237	NA
RPL21P134	7.322137	0.030237	NA
ENSG00000249495	7.322137	0.030237	NA
GARRE1	7.322137	0.030237	NA
FBXO5	7.322137	0.030237	NA
TRPM8	7.322137	0.030237	NA
MAP1A	7.322137	0.030237	NA
PHBP8	7.322137	0.030237	NA
BDP1P	7.322137	0.030237	NA
MIX23P3	7.322137	0.030237	NA
LGMNP1	7.322137	0.030237	NA
EEF1DP3	7.322137	0.030237	NA
RPL10P7	7.322137	0.030237	NA
STAT5A	7.31852	0.030446	NA
SNX18	7.318274	0.028103	0.28688
ZSCAN20	7.314276	0.030409	NA
TRABD	7.312947	0.030444	NA
C1orf43	7.30709	0.030546	NA
GAMT	7.306898	0.030685	NA
NSMCE4A	7.305577	0.030714	NA
MROH6	7.303538	0.030661	NA
ERLIN2	7.302089	0.030757	NA
TNFRSF9	7.301806	0.030701	NA
HNRNPA1P52	7.300083	0.03074	NA
ZNF480	7.293681	0.030866	NA
TSPAN32	7.291897	0.030929	NA
HLA-F	7.291122	0.03093	NA
ZNF350	7.290941	0.030952	0.299146
CELP	7.290927	0.015928	0.207577
NAAA	7.289159	0.025337	NA

NR5A2	7.284868	0.030432	NA
MAFF	7.281244	0.03086	NA
RPL7P21	7.280686	0.03119	NA
AKAP6	7.280449	0.020192	0.236138
SAXO1	7.279935	0.031206	NA
GNL3LP1	7.277099	0.031273	NA
GAREM2	7.268795	0.031481	NA
SLC1A7	7.268699	0.031601	NA
ZNF684	7.266708	0.031516	NA
OCEL1	7.264506	0.03158	NA
TRIM44	7.262668	0.03161	NA
INTS14	7.259611	0.030531	0.2967
GPR132	7.257648	0.031712	NA
HTT	7.251017	0.03183	NA
AMZ1	7.24696	0.031982	NA
UPK3BL1	7.243548	0.024047	NA
ZNF844	7.240564	0.032135	NA
AMBP	7.239646	0.032157	NA
LRRC74B	7.236509	0.014961	0.202004
LITAFD	7.235933	0.028196	NA
HEXD	7.235837	0.032351	NA
TCF7L1	7.234468	0.03228	NA
GPSM2	7.230982	0.032408	NA
DHRS2	7.230582	0.008275	0.171876
KBTBD8	7.228172	0.032432	NA
DHCR7	7.226014	0.032466	NA
ZNF317	7.216194	0.032716	NA
CBWD4P	7.208769	0.03253	NA
ENSG00000267640	7.208202	0.032915	NA
C1orf127	7.208044	0.032919	NA
CHCHD4	7.20679	0.03295	NA
ENSG00000218418	7.20529	0.032986	NA
ATL3	7.203066	0.032038	0.303987
IL11	7.201067	0.033091	NA
ANO3	7.197398	0.033179	NA
EEF1A1P27	7.196853	0.033193	NA
PCDHGC3	7.195976	0.033308	NA
GABBR1	7.189012	0.033386	NA
ZNF510	7.184977	0.033485	NA
IGFLR1	7.18457	0.033522	NA
SEMA7A	7.180557	0.033595	NA
C19orf48	7.175598	0.033766	NA
DGKQ	7.172023	0.026784	NA
B4GALNT3	7.163384	0.03242	0.304907

ENSG00000260092	7.16237	0.034048	NA
MYO1G	7.15935	0.03307	0.307706
FBXW9	7.159042	0.034133	NA
IL2RA	7.156965	0.032413	0.304907
LZTFL1	7.153443	0.034378	NA
UTY	7.152792	0.02222	0.249723
MIGA2	7.14992	0.027084	NA
BMPR1AP2	7.146992	0.034436	NA
CNTD1	7.145404	0.034476	NA
BTBD2	7.14378	0.034559	NA
BCL2L15	7.143734	0.034341	NA
PSMA7	7.142884	0.034611	NA
CCDC200	7.141371	0.033578	0.308918
RPL24P4	7.139107	0.034636	NA
MOSPD2	7.138702	0.034706	NA
FGFR2	7.135515	0.0303	NA
SSR4	7.124979	0.034915	NA
YIPF2	7.124945	0.034998	NA
SLC5A6	7.116338	0.033028	0.307706
ANTKMT	7.115089	0.035255	NA
B4GALT4	7.114445	0.033247	0.307961
LRRC14	7.107857	0.035413	NA
SLC23A3	7.106253	0.035481	0.316885
ENSG00000265798	7.102603	0.035575	NA
BCAS1	7.101624	0.034904	NA
XPC	7.099375	0.034272	0.312724
MAP3K6	7.097733	0.035704	NA
COL11A2	7.095508	0.035761	NA
BZW2	7.092972	0.035858	NA
MTRES1	7.088895	0.035936	NA
SFT2D1	7.08657	0.012747	0.195907
PPP1R15A	7.081589	0.036127	NA
COLGALT2	7.079947	0.036171	NA
LCMT1	7.078428	0.036193	NA
RTN2	7.077282	0.036219	NA
TBX3	7.072347	0.014605	0.201021
CTSB	7.065293	0.03656	NA
ASB13	7.063071	0.036134	NA
ENSG00000203262	7.058457	0.036742	0.319112
KANSL1	7.055315	0.016136	0.20972
ENSG00000227948	7.051819	0.036922	NA
ARHGEF4	7.049744	0.037272	0.319112
NADK	7.047072	0.037211	NA
RRAGC	7.046489	0.036831	NA

NUDT4	7.045896	0.031387	NA
PKIG	7.04471	0.037056	NA
ZNF282	7.043185	0.037154	NA
IMP4	7.042176	0.03716	NA
HIRA	7.041443	0.03736	NA
CRABP2	7.041331	0.037264	NA
IL15	7.037836	0.025981	0.274371
ENSG00000255639	7.037421	0.037309	0.319112
ENSG00000213067	7.037421	0.037309	0.319112
ENSG00000225869	7.037421	0.037309	0.319112
MRPS33P3	7.037421	0.037309	0.319112
KRT83	7.037421	0.037309	0.319112
YWHAEP1	7.037421	0.037309	NA
EI24P4	7.037421	0.037309	NA
NPAP1P3	7.037421	0.037309	NA
NECTIN1	7.036698	0.03731	NA
RTEL1-TNFRSF6B	7.034874	0.037378	NA
ENSG00000271355	7.032922	0.037431	NA
CDIPT	7.032734	0.037394	NA
TMEM201	7.029186	0.037661	NA
ELOVL6	7.019275	0.037804	NA
NPHP3	7.017978	0.032151	0.303987
PPP1R18	7.017216	0.037992	NA
CHPF2	7.016698	0.037875	NA
GPR137C	7.016007	0.037422	NA
MST1	7.015817	0.037934	NA
GZF1	7.0149	0.037923	NA
PPP2CA	7.012368	0.028519	NA
BMPR1AP1	7.009443	0.037658	NA
RPS21	7.007754	0.038123	NA
ENSG00000285476	7.006411	0.038159	NA
KRT17P1	7.005674	0.038179	NA
GFI1B	7.005674	0.038179	NA
FBLIM1P1	7.005674	0.038179	NA
TINCR	7.002573	0.014854	0.201743
PDIA3P1	7.00006	0.038333	NA
RIN1	6.998683	0.03833	NA
DAAM2	6.995846	0.005415	0.136765
ENSG00000283399	6.995487	0.038462	NA
SLIRP	6.995325	0.038784	NA
PMEPA1	6.994723	0.038781	NA
MDFI	6.993642	0.038533	NA
OGFOD2	6.993145	0.038539	NA
CKAP2L	6.989144	0.03883	NA

HMMR	6.983951	0.038682	NA
DENND1A	6.983148	0.006381	0.148174
ENSG00000224600	6.981917	0.038841	NA
C2CD5	6.981233	0.014308	0.200754
TRPC6	6.978444	0.038939	NA
CYTH4	6.97707	0.039047	NA
RBAK	6.973675	0.039073	NA
ASL	6.970522	0.039043	NA
NUDT14	6.969072	0.039449	NA
CHERP	6.96874	0.028905	0.289639
ZNF514	6.965111	0.037507	0.319112
STAG3L2	6.963765	0.019107	0.231606
GPR68	6.956111	0.039571	NA
EXOSC4	6.953625	0.039642	NA
INGX	6.952016	0.022517	NA
BAG6	6.944654	0.039909	NA
RBKS	6.936199	0.040143	NA
B3GNT4	6.934122	0.040192	NA
ENSG00000258134	6.932031	0.040263	NA
TMEM132B	6.93043	0.039135	0.327928
FAR1	6.930023	0.038961	0.32746
DEAF1	6.93001	0.040329	NA
HAT1	6.929287	0.016734	NA
ENSG00000260776	6.929222	0.040345	NA
NTNG1	6.927388	0.040529	NA
B3GAT1	6.92729	0.040397	NA
ENSG00000203546	6.926501	0.01342	NA
FYTTD1P1	6.924828	0.040472	NA
NCK2	6.922148	0.040256	NA
OIP5	6.921705	0.040563	NA
RXRB	6.921705	0.040563	NA
SAPCD2P3	6.921705	0.040563	NA
RPS15AP16	6.921705	0.040563	NA
DDX31	6.920613	0.040099	NA
NHSL2	6.920157	0.040692	NA
LRRFIP1P1	6.917371	0.040691	NA
SLC25A51P1	6.917054	0.040699	NA
KCTD1	6.916947	0.04084	NA
LRRC46	6.915601	0.040739	NA
EZH2	6.915538	0.040194	NA
WTAPP1	6.912653	0.040088	NA
POP1	6.91228	0.041166	NA
TCAF2P1	6.911256	0.040868	NA
NEK2	6.910047	0.022649	0.251856

EEF1E1	6.909297	0.040918	NA
MED13	6.907698	0.040937	NA
SHOX	6.905278	0.034059	NA
MED29	6.905216	0.041045	NA
TPTE2P1	6.903984	0.041082	NA
GPRC5C	6.902239	0.040631	NA
YJU2B	6.897498	0.041297	NA
KIAA1755	6.896674	0.041605	NA
CFAP44	6.894996	0.039647	0.330957
GRK6P1	6.891558	0.041449	NA
PCDHA4	6.884914	0.008969	0.175197
FOXK1	6.884223	0.041666	NA
MOCS1	6.882792	0.042006	NA
PIK3R2	6.881467	0.041742	NA
SOGA3	6.87615	0.041909	NA
MICALL2	6.874543	0.017076	0.217897
ENSG00000260537	6.873235	0.042117	NA
TNRC18P2	6.873086	0.042001	NA
FLVCR1	6.872103	0.042223	NA
BRMS1	6.871645	0.042017	NA
NDST2	6.867127	0.04137	NA
PGAM1P7	6.859702	0.042404	NA
SLC9A6	6.859591	0.042332	NA
SDCCAG8	6.855645	0.042503	NA
ENSG00000248401	6.855071	0.042544	NA
NEIL1	6.85086	0.014476	0.200962
SLC17A4	6.850416	0.042685	NA
FAM170A	6.850416	0.042685	NA
NEDD8-MDP1	6.849313	0.042719	NA
ENSG00000254595	6.849119	0.042725	NA
TBL2	6.84619	0.042814	NA
OR7A3P	6.843385	0.0429	NA
PTPN21	6.841337	0.042821	NA
BTNL9	6.841304	0.041292	0.33825
ZSCAN4	6.840218	0.042997	NA
TAS2R20	6.839875	0.043006	NA
KCTD9P5	6.838659	0.042202	NA
MSH5	6.837418	0.02751	0.283885
KLRC1	6.837266	0.043094	NA
IL11RA	6.835271	0.043441	NA
ITPRIPL2	6.834078	0.043186	NA
AIFM1	6.833459	0.043151	NA
DNAH8	6.831965	0.043264	NA
KLRG1	6.83162	0.043261	NA

REEP5	6.831572	0.043364	NA
PPTC7	6.829786	0.043317	NA
CYP2E1	6.829148	0.043337	NA
ENSG00000237846	6.828599	0.041313	0.33825
MICB	6.82837	0.043148	0.344343
CIBAR1P2	6.82619	0.043428	NA
OLFM2	6.824263	0.043753	NA
MTM1	6.822807	0.043532	NA
UBE2L2	6.820782	0.043595	NA
RPS6P20	6.820555	0.001102	0.040394
GEMIN4	6.818967	0.043652	NA
NDUFS3	6.815955	0.043876	0.346305
SNAPC2	6.815391	0.043721	NA
ENSG00000265690	6.814672	0.043785	NA
ENSG00000270400	6.814672	0.043785	NA
MCU	6.812324	0.044026	NA
BCL2L12	6.810775	0.044156	NA
ZKSCAN5	6.808346	0.044037	NA
SNRNP48	6.80712	0.043649	NA
LRFN3	6.806048	0.044053	NA
FHIT	6.806	0.044055	NA
NME3	6.805701	0.044064	NA
NLRP6	6.804015	0.044117	NA
ERICH3	6.803007	0.044115	NA
TTC12	6.799082	0.044475	NA
DTWD1	6.796016	0.044503	NA
MXRA7P1	6.7951	0.0444	NA
SLC43A1	6.793831	0.044719	NA
SYNGR2	6.792885	0.044636	NA
KHDRBS3	6.792487	0.044479	NA
PPP2R3B	6.792449	0.04448	NA
TPMTP1	6.789986	0.044558	NA
TOB2	6.787478	0.044637	NA
PARD6A	6.787151	0.044647	NA
IKZF3	6.787151	0.044647	NA
SERPINA3	6.786398	0.044621	NA
INSIG2	6.784982	0.044721	NA
UBN2	6.784962	0.044716	NA
PIPSL	6.783209	0.039072	NA
UGCG	6.782838	0.042984	0.343749
MRPL16	6.782819	0.04479	NA
GLRX3P2	6.781331	0.04483	NA
ENSG00000213605	6.779014	0.044904	NA
PIMREG	6.778927	0.044908	NA

CCT6P3	6.777771	0.04496	NA
EBLN2	6.776726	0.044978	NA
RPL19P16	6.774825	0.045038	NA
IZUMO1	6.770927	0.045162	NA
EFCAB12	6.769063	0.045529	NA
ENSG00000254851	6.768152	0.045251	NA
RPL4P5	6.7668	0.045294	NA
ZNF85	6.76551	0.043767	NA
DUX4L18	6.764965	0.045351	NA
ADGRF3	6.763031	0.045415	NA
KMO	6.762948	0.045417	NA
ENSG00000229399	6.762948	0.045417	NA
ST6GALNAC5	6.762435	0.045433	NA
KLHL17	6.761838	0.045438	NA
MOSPD1	6.761502	0.044773	NA
DHX9	6.761319	0.040078	0.332287
ITGBL1	6.759984	0.045488	NA
SEMA4G	6.759415	0.045618	NA
PGK1P1	6.758724	0.045552	NA
ARHGAP10	6.758336	0.045739	NA
FAM90A22P	6.758073	0.045573	NA
AMER2	6.757442	0.045594	NA
CDKN2D	6.756799	0.045614	NA
RAB27A	6.756635	0.045649	NA
POC1B-GALNT4	6.756243	0.045632	NA
FN3K	6.756	0.04564	NA
XPOTP1	6.755292	0.045663	NA
RPL6P24	6.755116	0.045668	NA
ENSG00000261457	6.754921	0.045675	NA
RPSAP47	6.754796	0.045679	0.349972
ENSG00000232502	6.754796	0.045679	0.349972
SPATS1	6.754796	0.045679	0.349972
GOLGA6L6	6.754796	0.045679	0.349972
COX6B2	6.754796	0.045679	0.349972
RPL12P40	6.754796	0.045679	NA
MEIOC	6.754796	0.045679	NA
HEPACAM2	6.754796	0.045679	NA
RPS4XP7	6.754796	0.045679	NA
CLCA3P	6.754796	0.045679	NA
RPL12P42	6.754796	0.045679	NA
OR2J4P	6.754796	0.045679	NA
OR2J4P	6.754796	0.045679	NA
ENSG00000248824	6.754796	0.045679	NA
CTNNA1P1	6.754796	0.045679	NA

DUX4L17	6.754796	0.045679	NA
ENSG00000280172	6.754796	0.045679	NA
ENSG00000280569	6.754796	0.045679	NA
SYT2	6.754796	0.045679	NA
SCD5	6.753228	0.044236	NA
ZDHHC12	6.751249	0.045792	NA
PROSER3	6.750962	0.023657	0.259629
CPNE7	6.750425	0.045801	NA
ENSG00000267022	6.750003	0.045833	NA
SINHCAFP1	6.749997	0.045833	NA
POP7	6.748375	0.045887	NA
ENSG00000230026	6.747867	0.045902	NA
C3orf52	6.747733	0.045906	NA
C8orf82	6.747437	0.045991	NA
SMG9	6.746464	0.045992	NA
STKLD1	6.745768	0.04597	NA
NPM1P48	6.745768	0.04597	NA
ENSG00000274775	6.745768	0.04597	NA
OR5C1	6.745257	0.045986	NA
EFCAB7	6.745257	0.045986	NA
VWA7	6.745151	0.04598	NA
RLIMP1	6.742315	0.046082	NA
MORN3	6.739821	0.004406	0.117334
MMP25	6.738985	0.046189	NA
SMC3	6.738985	0.046189	NA
ENSG00000205976	6.738985	0.046189	NA
NOL4L	6.738936	0.046728	NA
APBB3	6.735982	0.046207	NA
LINC00933	6.735827	0.046292	NA
GSTCD	6.735535	0.046545	NA
KRTAP1-1	6.732766	0.046391	NA
PDCL3	6.73271	0.04639	NA
CREBZF	6.731416	0.043425	0.345427
CHKA	6.731336	0.032045	0.303987
ZBTB22	6.730703	0.046458	NA
ERFE	6.730108	0.044559	0.349972
ICMT	6.728987	0.046473	NA
RNF14P1	6.727716	0.046556	NA
SMURF1	6.727695	0.046557	NA
SLCO1A2	6.725411	0.033099	0.307706
ENSG00000261717	6.722636	0.046722	NA
FRMD1	6.721663	0.04491	NA
PRSS55	6.721413	0.046953	NA
CLTB	6.720996	0.046785	NA

KLHDC2	6.719173	0.046714	NA
ZNF441	6.717524	0.04689	NA
METTL25	6.716338	0.046914	NA
ZNF25	6.716181	0.046935	NA
CENPF	6.714964	0.046975	NA
LRATD1	6.714957	0.046975	NA
DIRAS1	6.714957	0.046975	NA
TNFAIP3	6.714318	0.046636	NA
LIPG	6.71395	0.047211	NA
ANKRD65	6.712381	0.04706	NA
SERF1B	6.709901	0.039342	NA
ALDH1B1	6.709797	0.047145	NA
ANO7	6.709797	0.047145	NA
PDIK1L	6.708773	0.047179	NA
MRM3	6.708349	0.047189	NA
FER	6.708215	0.047276	NA
PAICSP1	6.707206	0.04723	NA
BCAS2P1	6.707206	0.04723	NA
SAT2	6.706414	0.047244	NA
FGF22	6.704596	0.047317	NA
MZB1	6.702177	0.047397	NA
ENSG00000233990	6.702177	0.047397	NA
ENSG00000251229	6.701997	0.047403	NA
LMF1	6.699315	0.046011	NA
CBR4	6.698974	0.047627	NA
FAM193B	6.698698	0.046466	NA
FCF1P7	6.698018	0.047535	NA
TBC1D25	6.697677	0.047547	NA
GLIPR1	6.696527	0.047585	NA
GABPAP	6.694293	0.047659	NA
ENSG00000220412	6.694123	0.047665	NA
MYCBPAP	6.692825	0.048224	NA
RPS2P45	6.690754	0.047777	NA
FAF2	6.690259	0.047794	NA
MSTO2P	6.689409	0.047822	NA
KCNS2	6.689098	0.047832	NA
TMC4	6.686173	0.04793	NA
GXYLT2	6.685984	0.047937	NA
GDI2P2	6.684534	0.047985	NA
HYAL3	6.683506	0.04802	NA
PLEKHG3	6.68311	0.047946	NA
ENSG00000205485	6.682372	0.047055	NA
BAALC	6.682028	0.048069	NA
C2orf78	6.680861	0.048111	NA

GPAM	6.68083	0.048109	NA
CARMIL3	6.68083	0.048109	NA
SERPINH1P1	6.68083	0.048109	NA
ENSG00000239392	6.679553	0.048152	NA
PCSK2	6.678368	0.048191	NA
SLC50A1	6.678238	0.019213	0.231606
EFCAB13	6.676677	0.048249	NA
CHORDC1P1	6.676565	0.048253	NA
RPL22P11	6.676565	0.048253	NA
ENSG00000283563	6.675632	0.048285	NA
ATP5PDP4	6.675422	0.048294	NA
POM121L7P	6.6753	0.048296	NA
ENSG00000278633	6.6753	0.048296	NA
CNTNAP3	6.674948	0.038621	0.325873
ENSG00000219273	6.674223	0.048332	NA
CFL1P3	6.672749	0.048382	NA
RPL4P3	6.670038	0.048473	NA
KLC2	6.669267	0.046145	0.35013
ENSG00000285329	6.667318	0.048565	NA
COX5BP6	6.667064	0.048574	NA
TYRO3P	6.66639	0.048597	NA
FUOM	6.665844	0.048719	NA
ZNF287	6.663455	0.048696	NA
FAM217B	6.662015	0.048745	NA
PSG6	6.66185	0.04875	NA
HAMP	6.661502	0.048762	NA
KCNQ4	6.660881	0.046717	0.351097
C1orf122	6.659731	0.048824	NA
RAB4A	6.659581	0.039693	NA
B3GALT4	6.659466	0.048832	NA
B3GALT4	6.659466	0.048832	NA
EFNA5	6.658573	0.048938	NA
ARHGEF6	6.658317	0.048871	NA
ABHD16B	6.655643	0.048963	NA
AGAP3	6.655344	0.049041	NA
PAFAH1B1P2	6.653579	0.049032	NA
TCF19	6.651696	0.049085	NA
DOCK11P1	6.650804	0.049127	NA
HLA-DMB	6.649286	0.048922	NA
HDHD5	6.649049	0.03458	0.314368
HMGXB4	6.648287	0.049086	NA
HSDL1	6.644896	0.049329	NA
SLC46A3	6.643185	0.049388	NA
CDSN	6.64242	0.049414	NA

SH3GL1P3	6.64242	0.049414	NA
ADAMTSL5	6.641647	0.049363	NA
DMC1	6.639037	0.045692	NA
CNOT7	6.637424	0.049518	NA
RPL17P26	6.6361	0.04963	NA
RAPGEF3	6.635945	0.048703	NA
PLXNC1	6.635193	0.049739	NA
STX12	6.63452	0.049688	NA
BEAN1	6.633757	0.049713	NA
NIPSNAP1	6.632294	0.038335	NA
CCNY	6.631893	0.04978	NA
ZFP90	6.631619	0.049786	NA
ATPAF1	6.631406	0.049637	NA
WDR41	6.630558	0.049791	NA
SKIV2L	6.630168	0.049837	NA
HLA-V	6.629699	0.032622	0.305636
TUBGCP5	6.628251	0.049903	NA
SKIV2L	6.627753	0.049923	NA
ADORA2A	6.626694	0.049798	NA
DEAF1	6.625388	0.050002	NA
PLEKHG4B	6.625213	0.044787	NA
ENSG00000242299	6.625125	0.04817	0.354939
METTL25B	6.623978	0.049924	NA
EMG1	6.623753	0.049478	NA
HES6	6.622514	0.050102	NA
HCFC2	6.621254	0.049997	NA
COX7C	6.614118	0.04307	NA
ADAM32	6.610766	0.019552	0.233665
DIPK2B	6.594908	0.049785	NA
MLKL	6.581308	0.034948	0.315366
LAPTM4B	6.581095	0.015037	0.202415
FAM133B	6.570703	0.035504	0.316885
COG4	6.570632	0.045134	0.349972
SPATA21	6.542416	0.043702	NA
ESPL1	6.538013	0.0379	NA
LINC01347	6.51775	0.020856	0.24159
NOCT	6.494241	0.008313	0.171876
CWC25	6.489171	0.007957	0.170506
CCDC120	6.479959	0.037467	0.319112
ZNF513	6.476556	0.049284	0.360971
ZNF273	6.468602	0.001973	0.064019
KCNJ15	6.456679	0.0162	0.209999
LAMP2	6.453023	0.012592	0.195907
MAGIX	6.434512	0.03488	NA

ENSG00000270518	6.429393	0.039764	0.33137
PPP3R1	6.374317	0.013957	0.19951
TMEM130	6.373646	0.041206	0.33825
ENSG00000255330	6.362014	0.035112	NA
ZCCHC4	6.353355	0.048749	NA
KCP	6.336436	0.011476	0.18878
ZC3H7B	6.316859	0.019365	0.232565
TNRC6C	6.308952	0.033949	0.310939
C12orf50	6.306779	0.008119	0.17087
ENSG00000217120	6.30293	0.008368	0.172285
CCDC158	6.29848	0.01294	0.19702
ANKRD36BP2	6.285863	0.034802	0.31489
TMEM266	6.26124	0.00929	0.175197
MRS2	6.259708	0.001244	0.044504
MARK2	6.257656	0.046968	0.351899
UEVLD	6.219918	0.047609	0.353463
HRK	6.170516	0.042449	0.342106
ARID4A	6.144978	0.031596	0.302383
ANXA3	6.124238	0.045491	0.349972
C12orf4	6.120561	0.021148	0.243826
ECD	6.109706	0.031571	0.302383
AKAP1	6.076349	0.036025	0.319112
FCRL2	6.073035	0.035446	0.316885
ACBD4	6.066247	0.018097	0.227369
QRSL1	6.062203	0.029449	0.292554
CNTNAP1	6.060917	0.046697	0.351097
ZNF286A	6.057236	0.044292	NA
ARL2	6.05047	0.002938	0.086519
UBOX5	5.994502	0.030191	0.295994
GRM6	5.993258	0.022206	0.249723
MROH7	5.978249	0.00169	0.0579
PKD1L2	5.971108	0.011864	0.189218
KLC3	5.970267	0.028532	0.288856
VPS52	5.940762	0.046315	NA
RNF207	5.939115	0.034206	0.312708
COMMD4	5.934881	0.015845	0.207072
C9orf85	5.934176	0.031922	NA
STAG3L3	5.906239	0.029012	0.290122
AKAP9	5.896981	0.02779	0.284896
EDRF1	5.886038	0.019934	0.234804
OTUB1	5.881317	0.023264	0.257319
SMG1P7	5.856812	0.013177	NA
EMC4	5.846921	0.00901	0.175197
RPTOR	5.804327	0.036645	0.319112

UGGT2	5.793939	0.017674	NA
IL10	5.762829	0.028774	0.289511
TMEM182	5.750263	0.008287	0.171876
ZDHHC8	5.749939	0.04371	NA
TRANK1	5.737498	0.033605	0.308918
RDH13	5.733148	0.030444	0.2967
DDX27	5.724672	0.002666	0.083195
DCUN1D2	5.721004	0.000565	0.023647
HACD2	5.695773	0.031932	0.303987
RBM17	5.68431	0.044743	0.349972
ETF1P2	5.666872	0.046609	0.351097
GBP5	5.650952	0.038195	NA
SLC35E1	5.645815	0.023377	0.257411
EIF4E2	5.632991	0.027519	0.283885
LINC02210	5.62796	0.047267	NA
CUL4A	5.615505	0.047531	0.353463
ACVR2B	5.61498	0.024875	0.264584
VPS11	5.611004	0.048828	0.358708
MECOM	5.597782	0.00574	0.142045
NAV3	5.563866	0.001542	0.054355
NUCB2	5.55886	0.047423	NA
FAM98C	5.548663	0.007035	0.158153
GPALPP1	5.529947	0.046177	0.35013
ERBB2	5.521294	0.016886	0.216305
MAST1	5.516402	0.033666	0.308918
MZT2A	5.510935	0.03336	0.308194
LRRD1	5.491281	0.047266	0.353053
LUC7L3	5.480248	0.044872	0.349972
AMDHD1	5.479612	0.01902	0.231256
NOPCHAP1	5.44879	0.031524	0.302383
PRRG3	5.447182	0.026238	NA
VWA5B1	5.421713	0.037882	0.321699
SEC14L1	5.42053	0.049795	0.364175
MINDY2	5.411908	0.043004	0.343749
BBS5	5.41148	0.029301	0.292418
IFT81	5.403183	0.038362	0.324655
MCFD2	5.358529	0.013098	0.19702
ENSG00000283384	5.346971	0.026719	0.280955
TAOK2	5.343829	0.04331	0.345074
CHCHD5	5.322163	0.041567	0.33825
HLA-DQA1	5.308903	0.002135	0.06884
ZMAT3	5.308295	0.030578	0.2967
HLA-V	5.27329	8.23E-05	0.004199
PLAAT3	5.236782	0.030264	0.295994

ADPGK	5.213476	0.032102	0.303987
GOT2	5.202143	0.041714	0.33825
ENSG00000229180	5.187778	0.04002	0.332287
RBM47	5.139247	0.024322	0.262632
G3BP2	5.13353	0.025532	0.270791
BORCS8	5.106819	0.041277	0.33825
METTL14	5.091947	0.048095	0.354939
COL28A1	5.071748	0.023925	NA
TOP3A	5.066953	0.04793	0.354772
HLA-A	5.061369	0.044793	0.349972
CDKL5	5.037718	0.035647	0.317579
ZKSCAN1	5.029733	0.028867	0.289639
GPATCH1	5.014273	0.038137	NA
DUOX1	4.990631	0.036523	0.319112
NUB1	4.985747	0.043931	NA
GABRG2	4.985355	0.001601	0.055635
ATXN3	4.922354	0.046816	0.351298
ZNF200	4.913822	0.045012	NA
KANK1	4.862336	0.032198	0.303987
MGST3	4.823912	0.047087	NA
TMEM106B	4.821949	0.044707	NA
CEP43	4.800881	0.026977	0.281245
TSSC2	4.792753	0.036853	NA
TBXA2R	4.763989	0.032174	0.303987
CYB5RL	4.712482	0.024802	0.264584
ATP5MC2	4.709559	0.028199	0.286892
ABHD18	4.705499	0.039361	NA
IRS3P	4.705292	0.044927	0.349972
HLA-F	4.698466	0.026946	0.281245
DPP9	4.663249	0.018772	0.230103
KBTBD12	4.660446	0.007036	0.158153
ENSG00000188897	4.653995	0.030674	0.297039
CRX	4.648538	0.01965	0.233738
PDE6A	4.490844	0.018971	0.231244
MNT	4.473008	0.012073	0.190828
TPGS1	4.461623	0.041763	0.33825
RUNDC3A	4.458015	0.034831	0.31489
E2F3P2	4.437976	0.009273	0.175197
FAM228B	4.416144	0.041508	0.33825
MRNIP	4.384345	0.014965	0.202004
REL	4.362625	0.011124	0.18731
TGFBRAP1	4.360829	0.022292	0.249954
ZNF724	4.340182	0.045702	0.349972
CAMKK1	4.306746	0.040965	0.33825

RBFA	4.264978	0.042053	0.339469
HLA-V	4.23255	0.036557	0.319112
ACTE1P	4.23155	0.028736	0.289511
GOSR1	4.15962	0.021981	0.24869
RASEF	4.114291	0.024387	0.262632
NEDD4	4.073041	0.01088	0.185106
CYP3A5	3.97124	0.047907	0.354772
POLR2J3	3.890211	0.03733	0.319112
EXD1	3.816708	0.039896	0.331899
HLA-F	3.768677	0.046398	0.350848
CFLAR	3.758253	0.036988	0.319112
GTDC1	3.736401	0.035356	0.316885
ZNF850	3.727195	0.045637	0.349972
ATP7B	3.626247	0.006001	0.146102
PLEKHH1	3.530615	0.048147	0.354939
MT-ND2	3.472961	0.036808	0.319112
TBC1D8B	3.46032	0.01752	0.222409
RICTOR	3.39002	0.037424	0.319112
SIAE	3.356105	0.026844	0.281245
BLOC1S6	3.326941	0.013091	0.19702
METTL21A	3.294999	0.036486	0.319112
CCL5	3.272568	0.035981	0.319112
TMEM45A	3.066914	0.041705	0.33825
PGF	3.036034	0.045847	0.349972
NEK7	-30	1.33E-25	6.53E-22
ENSG00000283738	-30	1.77E-19	6.68E-17
DYNC2H1	-29.6974	1.17E-21	1.15E-18
BUB1	-29.6745	7.42E-21	4.04E-18
DKC1	-29.4752	3.89E-21	2.38E-18
CALM2P2	-29.4057	3.55E-21	2.38E-18
ACTA1	-29.3009	1.19E-18	3.23E-16
LRRC37A2	-29.2653	8.79E-22	1.08E-18
PLXNA1	-29.1854	3.80E-25	9.31E-22
PDE4B	-28.8628	3.81E-18	9.83E-16
XPA	-28.8202	4.27E-18	1.05E-15
GUSBP9	-28.5968	1.35E-23	2.20E-20
NPHP1	-28.545	7.47E-20	3.05E-17
FEM1B	-28.5165	9.49E-18	2.21E-15
ABI1	-28.2541	1.88E-17	4.20E-15
RRAS2P1	-28.1808	2.28E-17	4.85E-15
PIK3AP1	-28.1556	1.70E-20	8.34E-18
ANKRD36	-28.0459	2.93E-20	1.30E-17
FOLR2	-28.0359	3.31E-17	6.46E-15
COMMD7	-28.0224	3.43E-17	6.46E-15

LLPH	-27.9228	4.43E-17	7.74E-15
WDR27	-27.9141	3.15E-19	1.03E-16
NPTN	-27.878	4.97E-17	8.39E-15
AKR1C2	-27.8259	5.67E-17	9.23E-15
PHC1P1	-27.8146	5.84E-17	9.23E-15
NCKAP5	-27.7567	6.77E-17	1.04E-14
TNXB	-27.7042	7.73E-17	1.15E-14
VSIG4	-27.6756	8.32E-17	1.20E-14
LSG1	-27.5902	1.03E-16	1.44E-14
SHLD2P3	-27.4834	3.90E-19	1.19E-16
ARMCX5	-27.4593	2.64E-19	9.25E-17
L3MBTL3	-27.3779	2.12E-21	1.73E-18
KATNIP	-27.2913	1.53E-16	2.08E-14
PRKCD	-27.2769	2.28E-16	3.02E-14
STX8P1	-27.0896	3.64E-16	4.35E-14
ENSG00000243554	-27.0513	4.01E-16	4.67E-14
PKD1P3	-26.9755	4.84E-16	5.40E-14
IGKV3D-20	-26.9743	4.85E-16	5.40E-14
KDM8	-26.945	5.22E-16	5.68E-14
MTG1	-26.8871	6.00E-16	6.39E-14
WASHC2C	-26.859	2.77E-16	3.57E-14
COMMD2	-26.662	9.14E-16	9.53E-14
TRPV4	-26.6602	1.05E-15	1.08E-13
DNAH17	-26.6245	1.15E-15	1.15E-13
CHCHD6	-26.5702	1.31E-15	1.29E-13
ENSG00000232702	-26.5298	1.45E-15	1.37E-13
TMEM87B	-26.5291	1.45E-15	1.37E-13
POFUT1	-26.3712	2.14E-15	1.97E-13
TNXA	-26.3286	2.37E-15	2.15E-13
MT1G	-26.1125	4.00E-15	3.50E-13
UBE2SP2	-26.0736	4.39E-15	3.71E-13
ANKRD20A5P	-25.9804	5.49E-15	4.56E-13
RAD21	-25.5314	1.60E-14	1.26E-12
ENSG00000282444	-25.0788	4.63E-14	3.38E-12
CLEC2B	-25.0253	5.23E-14	3.77E-12
SEC23IP	-13.1215	7.77E-05	0.00401
TNNT3	-12.6847	4.47E-05	0.002357
UBE2V1	-12.1759	0.000133	0.006562
NPIPA1	-12.0681	9.16E-05	0.004626
CFAP69	-11.8679	0.000144	0.006997
FAS	-11.5993	6.05E-05	0.003152
EP400P1	-11.3572	0.000628	0.02585
FRMD4A	-11.1047	0.000337	0.014999
CABYR	-10.5483	0.000849	0.032492

BOP1	-10.3435	0.001849	0.061629
ROBO3	-10.2099	0.001164	0.041954
IGKV1-16	-10.1653	0.001105	0.040394
WASH2P	-10.1566	0.001877	0.062148
ZNF776	-9.97382	0.00241	0.075707
BTN3A2	-9.96991	0.000958	0.035836
RHD	-9.90826	0.002858	0.086508
GIGYF2	-9.56281	0.000266	0.012534
EIF2B5	-9.47191	0.00286	0.086508
EXT1	-9.39462	0.002919	0.086519
TBC1D15	-9.25291	0.000739	0.029687
BPNT1	-9.13243	0.000801	0.030902
GNPNAT1	-9.09881	0.003014	0.087899
CUTC	-9.07527	0.004252	0.114999
PUM2	-9.05	0.00536	0.136209
HSP90AA1	-8.93166	0.000127	0.006345
ARL17B	-8.92203	0.004384	0.117334
IWS1	-8.85648	0.00634	0.148174
PRELID2	-8.83874	0.000314	0.014364
NPIPB13	-8.60568	0.000729	0.029515
OXCT1	-8.51266	0.010175	0.181497
ROBO1	-8.43272	0.0005	0.021479
KIF14	-8.38603	0.00492	0.126881
LPAL2	-8.37124	0.010223	0.181497
INTS13	-8.16075	0.011399	0.18878
RPL7P9	-8.14214	0.006895	0.1575
PSMB9	-8.06875	0.014863	0.201743
PRSS23	-8.04653	0.000761	0.030075
GPX8	-7.86957	0.010275	0.181757
ENAH	-7.63136	0.000314	0.014364
TP53I11	-7.51981	0.007706	0.168101
SOD1	-7.50105	0.015637	0.206527
UAP1	-7.44744	0.002256	0.071323
KLC1	-7.35095	0.000269	0.012545
DCAF13	-7.34343	0.003565	0.10098
CPS1	-7.31632	0.010391	0.183151
RPL5	-7.301	0.018803	0.230103
FAP	-7.26769	0.009356	0.175657
CR1	-7.1815	0.009465	0.176308
CBWD5	-7.17685	0.000888	0.033717
DDX3Y	-7.17514	0.021426	0.245684
PIK3C3	-7.15926	0.010204	0.181497
SMAD5	-7.1093	0.004271	0.114999
BUB1B	-6.9884	0.007695	0.168101

EDEM1	-6.98158	0.033105	0.307706
CHPT1	-6.90933	0.037247	0.319112
USF2	-6.88582	0.025723	0.27223
MYO5B	-6.8835	0.021346	0.245524
MAPK9	-6.86547	0.008775	0.175197
PXDN	-6.8565	0.005935	0.145408
TP53	-6.78988	0.010579	0.183207
ABI3BP	-6.73527	0.011691	0.18908
CDH13	-6.70551	0.014047	0.19951
ITGA11	-6.6806	0.028287	0.286967
ZC3H6	-6.65853	0.009867	0.179726
PRKACB	-6.58567	0.018597	0.230103
SMIM4	-6.54913	0.045967	0.35013
DOCK11	-6.44679	0.036155	0.319112
ERO1A	-6.39498	0.0089	0.175197
C4B	-6.35773	0.038214	0.323962
ACOX1	-6.35062	0.016907	0.216305
IDE	-6.31702	0.019693	0.233738
PSAP	-6.31359	0.004145	0.114761
RNF2	-6.28554	0.019529	0.233665
ARHGEF28	-6.26624	0.045039	0.349972
USP14	-6.2322	0.026192	0.275999
EIF5B	-6.19799	0.021015	0.242858
ADAMTS6	-6.18616	0.019903	0.234804
CLEC7A	-6.17365	0.000955	0.035836
AVIL	-6.15561	0.01977	0.233996
TIMP1	-6.10794	0.011556	0.18908
TOP2A	-6.10663	0.046231	0.35013
EEF1A1P6	-6.10557	0.043889	0.346305
TANGO6	-6.07709	0.036354	0.319112
QARS1	-6.03162	0.004842	0.126038
IRF9	-6.02853	0.041433	0.33825
NOMO1	-6.02653	0.020571	0.238854
EIF2A	-6.00106	0.028221	0.286892
NPM1	-5.96455	0.005858	0.144244
SLC39A10	-5.95524	0.029494	0.292554
PTGES3	-5.93979	0.022598	0.251856
HNRNPK	-5.91695	0.001356	0.048131
CD163	-5.91107	0.033398	0.308194
RELA	-5.78948	0.043599	0.345692
EPS15	-5.74042	0.036707	0.319112
DROSHA	-5.71771	0.014555	0.200962
ARHGDIB	-5.71094	0.042776	0.343592
NBPF8	-5.67251	0.009655	0.177858

POLR2B	-5.66607	0.004658	0.122056
NDUFV3	-5.65127	0.013549	0.19951
MIDEAS	-5.63795	0.015637	0.206527
NADK2	-5.63261	0.030464	0.2967
QRICH1	-5.6272	0.034485	0.314083
HNRNPDL	-5.59186	0.035264	0.316885
TUFM	-5.56766	0.01877	0.230103
NFX1	-5.5051	0.020146	0.236138
NRP1	-5.50132	0.006226	0.148174
NBN	-5.49487	0.019701	0.233738
BDP1	-5.4158	0.029605	0.293055
MAT2A	-5.40769	0.02794	0.285817
PGD	-5.3607	0.044262	0.348686
AZIN1	-5.3274	0.015488	0.206527
GNL2	-5.30868	0.013252	0.198581
CLU	-5.30336	0.029896	0.295347
TTC14	-5.2993	0.021794	0.247773
TNFRSF1B	-5.29845	0.035409	0.316885
DDX46	-5.28152	0.023699	0.259629
ARAP1	-5.28106	0.036188	0.319112
TM9SF3	-5.19545	0.037512	0.319112
NDUFA10	-5.19508	0.013103	0.19702
CRYAB	-5.17109	0.045735	0.349972
PARVA	-5.14381	0.033157	0.307706
RGPD5	-5.13548	0.03123	0.301234
ESYT1	-5.07989	0.027638	0.28451
RBM28	-5.06696	0.010515	0.183207
ECT2	-4.99663	0.010506	0.183207
USP34	-4.98439	0.041693	0.33825
ABCE1	-4.97498	0.015589	0.206527
PLS3	-4.96685	0.04488	0.349972
DYNLL1	-4.93838	0.048471	0.356618
PRUNE2	-4.93153	0.042634	0.343032
ELP2	-4.91324	0.049237	0.360971
CEP76	-4.89955	0.041888	0.338695
EIF2AK4	-4.88422	0.020443	0.237937
RAD50	-4.87777	0.04138	0.33825
RBBP6	-4.85283	0.038825	0.326878
NAPG	-4.84444	0.050014	0.364686
TXNDC15	-4.82837	0.038639	0.325873
ENSG00000273807	-4.74557	0.042844	0.343592
SAR1A	-4.73465	0.017222	0.219191
CKAP5	-4.67258	0.046148	0.35013
ULK2	-4.63347	0.046619	0.351097

SIPA1	-4.57044	0.045602	0.349972
P4HA1	-4.50047	0.03259	0.305636
TIMM50	-4.48389	0.031538	0.302383
CCZ1B	-4.35903	0.047134	0.352606
AP4E1	-4.33253	0.034674	0.31463
FLNA	-4.32562	0.027792	0.284896
FAM177A1	-4.28813	0.02196	0.24869
PPP2R1A	-4.26041	0.039625	0.330957
SREBF1	-4.24365	0.039151	0.327928
RYK	-4.23775	0.041135	0.33825
IGFBP7	-4.13518	0.014177	0.200195
HMGN1	-4.0662	0.045853	0.349972
LRCH3	-4.04019	0.036155	0.319112
NASP	-3.96019	0.047567	0.353463
ALMS1	-3.93691	0.047458	0.353463
PRRC2C	-3.93337	0.043803	0.346305
ENTPD1	-3.88059	0.043553	0.345692
GSN	-3.82693	0.049974	0.364686

Supplementary Table S6: Reactome pathway analysis result listing the enriched pathways represented by the up-regulated gene list in the *Cutibacterium*-high subgroup of thyroid cancer tumors.

Pathway identifier	Pathway name	#Entities found	#Entities total	Entities ratio	Entities pValue	Entities FDR	#Reactions found	#Reactions total	Reactions ratio	Species identifier	Species name	Submitted entities found
R-HSA-73780	RNA Polymerase III Chain Elongation	1	19	0.001	0.027	0.164	3	3	0.000	9606	Homo sapiens	POLR3H
R-HSA-909733	Interferon alpha/beta signaling	2	190	0.013	0.031	0.164	2	25	0.002	9606	Homo sapiens	HLA-F
R-HSA-73980	RNA Polymerase III Transcription Termination	1	23	0.002	0.033	0.164	2	2	0.000	9606	Homo sapiens	POLR3H
R-HSA-9609523	Insertion of tail-anchored proteins into the endoplasmic reticulum membrane	1	25	0.002	0.036	0.164	3	7	0.001	9606	Homo sapiens	BAG6
R-HSA-76066	RNA Polymerase III Transcription Initiation From Type 2 Promoter	1	27	0.002	0.039	0.164	2	4	0.000	9606	Homo sapiens	POLR3H
R-HSA-76071	RNA Polymerase III Transcription Initiation From Type 3 Promoter	1	28	0.002	0.040	0.164	2	4	0.000	9606	Homo sapiens	POLR3H
R-HSA-76061	RNA Polymerase III Transcription Initiation From Type 1 Promoter	1	28	0.002	0.040	0.164	2	5	0.000	9606	Homo sapiens	POLR3H
R-HSA-1296059	G protein gated Potassium channels	1	31	0.002	0.044	0.164	3	3	0.000	9606	Homo sapiens	GABBR1

R-HSA-1296041	Activation of G protein gated Potassium channels	1	31	0.002	0.044	0.164	3	3	0.000	9606	Homo sapiens	GABBR1
R-HSA-997272	Inhibition of voltage gated Ca2+ channels via Gbeta/gamma subunits	1	31	0.002	0.044	0.164	3	3	0.000	9606	Homo sapiens	GABBR1
R-HSA-76046	RNA Polymerase III Transcription Initiation	1	36	0.002	0.051	0.164	6	13	0.001	9606	Homo sapiens	POLR3H
R-HSA-877300	Interferon gamma signaling	2	252	0.017	0.051	0.164	2	18	0.001	9606	Homo sapiens	HLA-F
R-HSA-1296065	Inwardly rectifying K+ channels	1	38	0.003	0.054	0.164	3	7	0.001	9606	Homo sapiens	GABBR1
R-HSA-5365859	RA biosynthesis pathway	1	41	0.003	0.058	0.164	1	11	0.001	9606	Homo sapiens	RDH13

Supplementary Table S7: Reactome pathway analysis result listing the downregulated pathways represented by the down-regulated gene list in the *Cutibacterium*-high subgroup of thyroid cancer tumors.

Pathway identifier	Pathway name	#Entities found	#Entities total	Entities ratio	Entities pValue	Entities FDR	#Reactions found	#Reactions total	Reactions ratio	Species identifier	Species name	Submitted entities found
R-HSA-445989	TAK1-dependent IKK and NF-kappa-B activation	4	55	0.004	0.000	0.011	9	17	0.001	9606	Homo sapiens	USP14;UBE2V1;TP53;RELA

R-HSA-6804754	Regulation of TP53 Expression	2	4	0.000	0.000	0.011	5	5	0.000	9606	Homo sapiens	TP53
R-HSA-168138	Toll Like Receptor 9 (TLR9) Cascade	5	121	0.008	0.000	0.022	14	80	0.006	9606	Homo sapiens	USP14;PIK3C3;UBE2V1;TP53;RELA
R-HSA-9020702	Interleukin-1 signaling	5	125	0.008	0.001	0.023	20	59	0.004	9606	Homo sapiens	USP14;UBE2V1;TP53;RELA;PSMB9
R-HSA-8853884	Transcriptional Regulation by VENTX	3	48	0.003	0.002	0.064	4	13	0.001	9606	Homo sapiens	TP53;RELA
R-HSA-2871837	FCERI mediated NF-kB activation	5	175	0.012	0.002	0.064	7	19	0.001	9606	Homo sapiens	IGKV1-16;IGKV3D-20;UBE2V1;RELA;PSMB9
R-HSA-975871	MyD88 cascade initiated on plasma membrane	4	109	0.007	0.003	0.064	12	70	0.005	9606	Homo sapiens	USP14;UBE2V1;TP53;RELA
R-HSA-168176	Toll Like Receptor 5 (TLR5) Cascade	4	109	0.007	0.003	0.064	12	71	0.005	9606	Homo sapiens	USP14;UBE2V1;TP53;RELA
R-HSA-168142	Toll Like Receptor 10 (TLR10) Cascade	4	109	0.007	0.003	0.064	12	71	0.005	9606	Homo sapiens	USP14;UBE2V1;TP53;RELA

R-HSA-446652	Interleukin-1 family signaling	5	183	0.012	0.003	0.064	21	92	0.007	9606	Homo sapiens	USP14;UBE2V1;TP53;RELA;PSMB9
R-HSA-975138	TRAF6 mediated induction of NFkB and MAP kinases upon TLR7/8 or 9 activation	4	116	0.008	0.003	0.064	12	60	0.004	9606	Homo sapiens	USP14;UBE2V1;TP53;RELA
R-HSA-168164	Toll Like Receptor 3 (TLR3) Cascade	4	116	0.008	0.003	0.064	12	73	0.005	9606	Homo sapiens	USP14;UBE2V1;TP53;RELA
R-HSA-975155	MyD88 dependent cascade initiated on endosome	4	117	0.008	0.003	0.064	13	75	0.005	9606	Homo sapiens	USP14;UBE2V1;TP53;RELA
R-HSA-168181	Toll Like Receptor 7/8 (TLR7/8) Cascade	4	118	0.008	0.003	0.064	13	79	0.006	9606	Homo sapiens	USP14;UBE2V1;TP53;RELA
R-HSA-8877330	RUNX1 and FOXP3 control the development of regulatory T lymphocytes (Tregs)	2	17	0.001	0.004	0.064	2	20	0.001	9606	Homo sapiens	CR1
